# Unexpected cross-species contamination in genome sequencing projects

**DOI:** 10.7717/peerj.675

**Published:** 2014-11-20

**Authors:** Samier Merchant, Derrick E. Wood, Steven L. Salzberg

**Affiliations:** 1 Center for Computational Biology, McKusick-Nathans Institute of Genetic Medicine, Johns Hopkins University, Baltimore, MD, USA; 2 Department of Computer Science, Brown University, Providence, RI, USA; 3 Department of Computer Science, Johns Hopkins University, USA; 4 Department of Biomedical Engineering, Johns Hopkins University, USA

**Keywords:** Genomics, Bioinformatics, Genome assembly, Microbiome, Sequence analysis, DNA sequencing

## Abstract

The raw data from a genome sequencing project sometimes contains DNA from contaminating organisms, which may be introduced during sample collection or sequence preparation. In some instances, these contaminants remain in the sequence even after assembly and deposition of the genome into public databases. As a result, searches of these databases may yield erroneous and confusing results. We used efficient microbiome analysis software to scan the draft assembly of domestic cow, *Bos taurus,* and identify 173 small contigs that appeared to derive from microbial contaminants. In the course of verifying these findings, we discovered that one genome, *Neisseria gonorrhoeae* TCDC-NG08107, although putatively a complete genome, contained multiple sequences that actually derived from the cow and sheep genomes. Our findings illustrate the need to carefully validate findings of anomalous DNA that rely on comparisons to either draft or finished genomes.

## Introduction

Genome sequencing projects have dramatically increased in number and complexity in recent years. The first complete bacterial genome, *Haemophilus influenzae*, appeared in 1995, and today the public GenBank database contains over 27,000 prokaryotic and 1,600 eukaryotic genomes. Although many of these are draft genomes that contain gaps in their sequences, over 3,000 of the prokaryotic genomes are listed as complete, meaning that every nucleotide is present with no gaps.

The recent dramatic growth in microbiome research has been driven not only by the falling cost of sequencing, but by this large and growing set of known genomes. The large set of completed genomes makes it possible to identify, usually with high confidence, the species present in a sample of DNA taken from a site on the human body. The accuracy of microbiome analysis is critically dependent on the accuracy of the previously-sequenced microbial genomes. The vast majority of these sequences are accurate, but any errors may be amplified by efforts to search for the presence of unusual or unexpected species. This paper describes the finding of unexpected contaminants in two published genomes and the methods used to identify them.

Each genome sequencing project begins with a DNA source, which varies depending on the species. For animals, blood is a common source, while for smaller organisms such as insects the entire organism or a population of organisms may be required to yield enough DNA for sequencing. Throughout the process of DNA isolation and sequencing, contamination remains a possibility. Computational filters applied to the raw sequencing reads are usually effective at removing common laboratory contaminants such as *E. coli*, but other contaminants may be more difficult to identify. Human DNA is another common contaminant, presumably from the scientists who handle the samples at various times during the process of extraction through sequencing (Longo, O’Neill & O’Neill, 2011).

The current project was initiated when we learned that a microbiome project studying samples collected from domestic cows (*Bos taurus*) had identified the presence of a possible human pathogen that does not infect cows. As we investigated further, we discovered, first, that some of the original *Bos taurus* sequences were actually bacteria, and second, that some sequences from a published genome of *Neisseria gonorrhoeae* were actually cow and sheep DNA.

## Methods

We began by using microbiome sequence analysis software to analyze the genome of the domestic cow, *Bos taurus*, for signs of microbial contamination. The *Bos taurus* genome was originally assembled from 35 million Sanger reads (Zimin et al., 2009). The vast majority of the assembly (version UMD 3.1) was mapped onto chromosomes, but a small fraction remained unmapped, as is common with all draft genomes. When we began our investigation, the UMD 3.1 assembly had 3,286 unmapped contigs containing 9,499,556 nucleotides.

To analyze the unplaced contigs from the *Bos taurus* genome, we used the Kraken system (Wood & Salzberg, 2014) to classify each contig. Kraken is a very fast method for identifying the species represented by a DNA sequence, using exact matching of short subsequences of length *k*, called *k*-mers. The software uses a specialized database of *k*-mers (where *k* = 31 by default) that can be constructed from any set of genomes. For our study, we built a database containing all bacteria, archaea, and viruses. To classify a new sequence *S*, Kraken looks up every *k*-mer in *S* to determine if it exists in any known species. If a *k*-mer occurs in more than one species, Kraken assigns it to the lowest common ancestor (LCA) of those species. After looking up every *k*-mer, Kraken then uses a weighted voting scheme to determine the species or higher-order clade assignment for *S*.

Our Kraken database contained 2,757 bacterial and archaeal genomes and 2,335 viral genomes from the RefSeq database at NCBI (Tatusova et al., 2014). The Kraken software (http://ccb.jhu.edu/software/kraken/) includes an automated program that will download all these genomes directly from NCBI and build a local database. It also includes instructions on how to build a database using a customized set of species.

After using Kraken to process the 3,286 unmapped *Bos taurus* contigs, we ran a second analysis looking at the protein translations of these contigs. For this analysis, we created a database with all protein sequences from the 2,757 complete microbial genomes and used BLASTX (Camacho et al., 2009) to align each contig to the database. As a quality control step, we also ran Kraken on most of the mapped contigs, using all sequences from chromosomes 1 through 10. All experiments were run on a computer with 256 GB of RAM and four 2.1 GHz, 12-core AMD Opteron processors. Kraken processed the 3,286 unplaced contigs (9.5 megabases) in just 3.98 s.

## Results and Discussion

After removing low-complexity contigs (some of which contained nothing other than a series of dinucleotide repeats), 138 contigs from the *Bos taurus* UMD 3.1 assembly were identified as bacterial in origin. The BLASTX search, which was far slower but more sensitive, confirmed these 138 and identified 35 additional contaminants including both bacteria and viruses, for a total of 173 contaminant contigs. Table S1 lists all the contigs with the closest matching microbial species for each one. The most common contaminants found belonged to the genera *Acinetobacter* (29 contigs), *Pseudomonas* (35 contigs), and *Stenotrophomonas* (27 contigs). Note that additional microbial species might still be present but undetectable, if they derive from organisms that are not similar to any sequenced species.

One interesting finding from the unplaced contigs was Bovine herpesvirus 6, isolate Pennsylvania 47, a cattle-specific virus that causes multiple diseases. Because this is a retrovirus, we considered the possibility that it had actually inserted itself into the host genome—i.e., that it was part of the genome and not a contaminant— in which case we would expect parts of the sequence to appear in the chromosomal contigs.

To evaluate this hypothesis, we used the nucmer program from the MUMmer package (Delcher et al., 2002; Kurtz et al., 2004) to align the entire bovine herpesvirus genome against the entire *Bos taurus* assembly. This alignment yielded the same five contigs (Table S1, contigs 149–153) we had found in our original scan, indicating that the virus was not integrated into the chromosomal DNA but rather an infection in the original animal.

To reflect these findings, we created a new release of the *Bos taurus* assembly, numbered 3.1.1, available as Bos_taurus_UMD_3.1.1 at NCBI (Accession GCF_000003055.5) and also available from www.ccb.jhu.edu/bos_taurus_assembly.shtml.

We then used Kraken to search all of the sequences placed on chromosomes 1 through 10, as a quality check on our method. We did not expect any of these contigs to match bacteria, but we unexpectedly found 2,885 small contigs that seemed to align in part to a single bacterial genome, *Neisseria gonorrhoeae*, strain TCDC-NG08107 (Chen et al., 2011). This bacterium is a human-specific pathogen, and it seemed highly unlikely that it had contaminated the original DNA used for sequencing.

Upon further investigation, we found that every contig aligned to one of just four locations on the TCDC-NG08107 strain, shown in Table 1. The aligned regions ranged in length from 200 to 634 bp. When we extracted these sequences and aligned them separately to all sequences in GenBank, all of the matching sequences were from *Bos taurus*.

**Table 1:** Locations of foreign DNA in *Neisseria gonorrhoeae* TCDC-NG08107 genome. E-values in column 4 were computed by the BLAST program in a search against the NCBI comprehensive sequence database.

Genome coordinates	Length	True species	BLAST E-Value
499351–499709	359	Cow	3 × 10^−168^
1267185–1267393	209	Cow	1 × 10^−71^
1371560– 1371932	373	Cow	2 × 10^−130^
1635755–1635954	200	Cow	3 × 10^−93^
2118014–2118647	634	Sheep	0.0

In an effort to determine the source of these foreign sequences in the TCDC-NG08107 genome (Genbank accession CP002440), we examined the original publication (Chen et al., 2011) and the GenBank entry, and found that although the genome was listed as complete in GenBank, Chen et al. (2011) described an assembly that comprised 180 contigs. Neither the publication nor the GenBank entry contained any information that the gaps had been filled. We concluded that sequence was erroneously uploaded as a finished genome, with all contigs simply concatenated together, and that the cow and sheep sequences represented accidental contaminants, presumably inserted computationally.

We then used the nucmer program (Kurtz et al., 2004) to align TCDC-NG08107 to its two closest relatives among the complete bacterial genomes, strains FA1090 and NCCP11945 (GenBank accessions AE004960 and CP001050), which were also used by Chen et al. (2011) to order and orient their original set of 180 contigs. These alignments indicated 181 separate alignments, in close agreement with the publication. We also found 67 small segments that did not align to either of the related strains. Normally, these would represent sequences that are insertions in TCDC-NG08107 as compared to other strains, a common finding when comparing bacterial genomes. However, these small segments included the regions that had matched the cow genome (Table 1). As a further check, we aligned all 67 segments to the NCBI comprehensive nucleotide database. As shown in Table 1, four of these segments matched *Bos taurus*, and a fifth segment aligned to *Ovis aries* (sheep). Not surprisingly, none of these five mammalian DNA fragments matched any other microbial species.

After removing the contaminated contigs, we used our alignments to re-order the remaining contigs using both NCCP11945 and FA1090. We removed 11 contigs that were fully contained within other contigs. This process yielded a reconstructed draft genome of TCDC-NG08107 with a total of 165 contigs, available in the Supplemental Information. However, because we did not have access to the original TCDC-NG08107 data and because the original submitters did not respond to any requests for data, we cannot be confident that these contigs are the best representation of the genome. As a result of our findings, GenBank has temporarily suppressed the entry for this genome.

### Contaminants in other genomes

As a test of whether these findings might apply to other publicly available genomes, we randomly selected eight additional genomes from the NCBI database and ran Kraken on each of them. The eight genomes range in size from 75 to 700 Mbp and include animals, plants, and fungi. We also performed BLAST searches for each of the sequences that Kraken identified as contaminants (Table 2), all of which were confirmed as microbial species. Three of the eight genome assemblies contained just 2–4 contaminant contigs, and one (*C. reinhardtii*) had 227, roughly similar to the number we found in *Bos taurus*.

**Table 2:** Results of screening 8 publicly available draft genomes for microbial contaminants. GenBank accession numbers are shown for each genome along with the number of contigs and the size of the draft assembly. The last column shows the sequencing technology used for each project.

Genome	# of contaminant contigs	Total contaminant length (bp)	Range of E-values	Total # of contigs	Genome size (Mbp)	Technology
*Schistosoma haematobium* (GCA_000699445.1)	4	9,415	4E-71–0.0	49,195	35	Illumina
*Cynoglossus semilaevis* (GCA_000523025.1)	2	904	1E-6–5E-22	62,912	470	Illumina
*Caenorhabditis brenneri* (GCA_000143925.2)	2	19,677	0.0	13,373	190	ABI solid sequencing
*Chlamydomonas reinhardtii* (GCA_000002595.2)	227	254,869	0.0	11,385	120	Sanger
*Citrus clementina* (GCA_000493195.1)	0	0	N/A	8,962	301	Sanger
*Anopheles darlingi* (GCA_000211455.3)	0	0	N/A	13,857	174	454
*Auricularia delicata* TFB-10046 SS5 (GCA_000265015.1)	0	0	N/A	4,884	75	Illumina
*Schmidtea mediterranea* (GCA_000691995.1)	0	0	N/A	118,433	701	Illumina

## Conclusion

These results illustrate the importance of performing a thorough search for contamination before submitting a genome sequence to a public archive. The rapidly growing number of draft genomes represents both a valuable resource and also, as we show here, a cautionary tale. Perhaps most problematic was the presence of foreign DNA in *N*. *gonorrhoeae* TCDC-NG08107, a genome that was submitted to GenBank as complete. If scientists cannot assume that the sequence of a species truly comes from that species, then analyses that use this data may be fundamentally flawed. Contamination from other species may masquerade as lateral gene transfer (Willerslev et al., 2002), an event that is relatively common between some bacteria but extremely rare otherwise. In particular, the transfer of bacterial DNA directly into a mammalian genome has been suggested previously, based on compositional analysis, but never proven (Salzberg et al., 2001). The presence of erroneously labelled DNA causes particular problems for microbiome analysis, in which the primary goal is the identification of which species are present in a sample. These findings highlight the importance of careful screening of DNA sequence data both at the time of release and, in some cases, for many years after publication.

## Supplemental Information

10.7717/peerj.675/supp-1 Table S1 Supplementary Table S1 173 contigs from the *Bos taurus* assembly identified as possible contaminants. The closest matching bacterial, archaeal, or viral species is shown. Sequence ID refers to the original identifier in the *Bos taurus* UMD 3.1 assembly. All contigs belonged to the unmapped set; none were mapped onto chromosomes. The final column shows the BLAST E-value from an alignment of each contig against the comprehensive DNA sequence database “nr” at NCBI.

10.7717/peerj.675/supp-2 Supplemental Information 2 This file contains the sequences, in FASTA format, of the 165 re-ordered contigs that comprise the reconstruction of the *Neisseria gonorrhoeae* genome

10.7717/peerj.675/supp-3 Supplemental Information 3 This file contains the sequences, in FASTA format, of the five segments of DNA from the *N. gonorrhoeae* TCDC-NG08107 genome that were identified as cow and sheep sequences
