# Supplementary material for: Unexpected cross-species contamination in genome sequencing projects
Source: PeerJ. 2014 Nov 20;2:e675. doi: 10.7717/peerj.675 (PMC4243333; doi:10.7717/peerj.675)
Supplement: Table S1 — 173 contigs from the Bos taurus assembly identified as possible contaminants. The closest matching bacterial, archaeal, or viral species is shown. Sequence ID refers to the original identifier in the Bos taurus UMD 3.1 assembly. All contigs belonged to the unmapped set; none were mapped onto chromosomes. The final column shows the BLAST E-value from an alignment of each contig against the comprehensive DNA sequence database “nr” at NCBI. [file peerj-02-675-s001.docx]

| **#** | **Sequence ID** | **Kraken classification** | **Length (bp)** | **BLAST**  **E-value** |
| --- | --- | --- | --- | --- |
| **1** | 7180001691515 | Achromobacter xylosoxidans A8 | 1605 | 0.0 |
| **2** | 7180001699216 | Achromobacter xylosoxidans A8 | 1002 | 0.0 |
| **3** | 7180001694778 | Acidovorax avenae subsp. avenae ATCC 19860 | 1061 | 0.0 |
| **4** | 7180001701923 | Acinetobacter baumannii 1656-2 | 1309 | 0.0 |
| **5** | 7180001701419 | Acinetobacter baumannii ATCC 17978 | 1545 | 1.0E-02 |
| **6** | 7180001701949 | Acinetobacter baumannii ATCC 17978 | 1044 | 6.0E-02 |
| **7** | 7180001712792 | Acinetobacter baumannii ATCC 17978 | 1609 | 0.0 |
| **8** | 7180001697315 | Acinetobacter baumannii AYE | 1201 | 0.0 |
| **9** | 7180001706703 | Acinetobacter baumannii AYE | 1089 | 0.0 |
| **10** | 7180001999774 | Acinetobacter baumannii AYE | 3365 | 1.0E-02 |
| **11** | 7180002004940 | Acinetobacter baumannii AYE | 2113 | 0.0 |
| **12** | 7180001701648 | Acinetobacter baumannii BJAB0715 | 1451 | 0.0 |
| **13** | 7180002004930 | Acinetobacter baumannii BJAB0715 | 2692 | 0.0 |
| **14** | 7180002004148 | Acinetobacter baumannii D1279779 | 3098 | 0.0 |
| **15** | 7180001705700 | Acinetobacter baumannii SDF | 1181 | 3.0E-124 |
| **16** | 7180001714277 | Acinetobacter baumannii SDF | 3163 | 0.0 |
| **17** | 7180001705678 | Acinetobacter baumannii ZW85-1 | 1589 | 0.0 |
| **18** | 7180002006808 | Acinetobacter baumannii ZW85-1 | 2314 | 0.0 |
| **19** | 7180001716509 | Acinetobacter calcoaceticus PHEA-2 | 1592 | 0.0 |
| **20** | 7180001999985 | Bacillus thuringiensis | 2515 | 0.0 |
| **21** | 7180001713704 | Bacillus cereus AH187 | 1398 | 0.0 |
| **22** | 7180001717806 | Bacillus megaterium DSM 319 | 1330 | 3.0E-20 |
| **23** | 7180001702762 | Bacillus thuringiensis konkukian str. 97-27 | 1833 | 4.0E-110 |
| **24** | 7180001708562 | Bacillus thuringiensis thuringiensis str. IS5056 | 1370 | 0.0 |
| **25** | 7180001877879 | Choristoneura occidentalis granulovirus | 1301 | 2.0E-27 |
| **26** | 7180001699285 | Bradyrhizobium diazoefficiens USDA 110 | 1279 | 3.0E-139 |
| **27** | 7180001707983 | Burkholderia ambifaria AMMD | 1326 | 0.0 |
| **28** | 7180001695504 | Burkholderia sp. YI23 | 1361 | 0.0 |
| **29** | 7180001693117 | Acidovorax | 1423 | 0.0 |
| **30** | 7180001700258 | Delftia | 1251 | 3.0E-130 |
| **31** | 7180001691753 | Acidovorax sp. KKS102 | 1241 | 0.0 |
| **32** | 7180001696073 | Acidovorax sp. KKS102 | 1006 | 0.0 |
| **33** | 7180001696863 | Acidovorax sp. KKS102 | 1327 | 0.0 |
| **34** | 7180001702419 | Acidovorax sp. KKS102 | 1311 | 0.0 |
| **35** | 7180001708186 | Acidovorax sp. KKS102 | 1299 | 0.0 |
| **36** | 7180001732036 | Acidovorax sp. KKS102 | 1144 | 0.0 |
| **37** | 7180001691654 | Alicycliphilus denitrificans | 1092 | 1.0E-167 |
| **38** | 7180001691556 | Comamonas testosteroni CNB-2 | 1491 | 0.0 |
| **39** | 7180001691762 | Comamonas testosteroni CNB-2 | 1107 | 0.0 |
| **40** | 7180001691912 | Comamonas testosteroni CNB-2 | 1079 | 0.0 |
| **41** | 7180001692022 | Comamonas testosteroni CNB-2 | 1230 | 0.0 |
| **42** | 7180001700513 | Comamonas testosteroni CNB-2 | 1255 | 0.0 |
| **43** | 7180001708911 | Comamonas testosteroni CNB-2 | 1798 | 0.0 |
| **44** | 7180001695134 | Cronobacter sakazakii SP291 | 1537 | 9.0E-111 |
| **45** | 7180001697043 | Cronobacter sakazakii SP291 | 2524 | 0.0 |
| **46** | 7180001696847 | Cupriavidus metallidurans CH34 | 1052 | 9.0E-55 |
| **47** | 7180001715091 | Delftia acidovorans SPH-1 | 1159 | 7.0E-71 |
| **48** | 7180001695082 | Enterobacter cloacae subsp. cloacae ATCC 13047 | 1831 | 0.0 |
| **49** | 7180001695083 | Enterobacter cloacae subsp. cloacae ATCC 13047 | 3068 | 0.0 |
| **50** | 7180001695087 | Enterobacter cloacae subsp. cloacae ATCC 13047 | 1376 | 0.0 |
| **51** | 7180001695131 | Enterobacter cloacae subsp. cloacae ATCC 13047 | 1125 | 0.0 |
| **52** | 7180001697057 | Enterobacter cloacae subsp. cloacae ATCC 13047 | 1394 | 0.0 |
| **53** | 7180001697087 | Enterobacter cloacae subsp. cloacae ATCC 13047 | 8888 | 0.0 |
| **54** | 7180001999886 | Enterobacter cloacae subsp. cloacae ATCC 13047 | 6157 | 0.0 |
| **55** | 7180002004479 | Enterobacter cloacae subsp. cloacae ATCC 13047 | 3155 | 0.0 |
| **56** | 7180002004855 | Enterobacter cloacae subsp. cloacae ATCC 13047 | 2609 | 0.0 |
| **57** | 7180002006322 | Enterobacter cloacae subsp. cloacae ATCC 13047 | 3393 | 0.0 |
| **58** | 7180002006328 | Enterobacter cloacae subsp. cloacae ATCC 13047 | 3366 | 0.0 |
| **59** | 7180002006330 | Enterobacter cloacae subsp. cloacae ATCC 13047 | 3874 | 0.0 |
| **60** | 7180002006331 | Enterobacter cloacae subsp. cloacae ATCC 13047 | 7695 | 0.0 |
| **61** | 7180002015322 | Enterobacter cloacae subsp. cloacae ATCC 13047 | 5829 | 0.0 |
| **62** | 7180001981944 | Pantoea sp. At-9b | 1149 | 0.0 |
| **63** | 7180001694408 | Herbaspirillum seropedicae SmR1 | 1001 | 0.0 |
| **64** | 7180001702918 | Herbaspirillum seropedicae SmR1 | 1016 | 0.0 |
| **65** | 7180001780930 | Human herpesvirus 7 | 1762 | 1.0E-05 |
| **66** | 7180002004853 | Klebsiella oxytoca E718 | 2981 | 0.0 |
| **67** | 7180001702001 | Lysinibacillus sphaericus C3-41 | 7780 | 0.0 |
| **68** | 7180001712366 | Acinetobacter baumannii | 1297 | 0.0 |
| **69** | 7180001691226 | Novosphingobium aromaticivorans DSM 12444 | 1254 | 0.0 |
| **70** | 7180001713639 | Pantoea vagans C9-1 | 1132 | 0.0 |
| **71** | 7180001704583 | Phenylobacterium zucineum HLK1 | 1126 | 1.0E-48 |
| **72** | 7180001692000 | Pseudomonas protegens | 1109 | 0.0 |
| **73** | 7180001698462 | Pseudomonas protegens | 1011 | 4.0E-157 |
| **74** | 7180001730725 | Pseudomonas protegens | 1105 | 2.0E-66 |
| **75** | 7180001712491 | Pseudomonas | 1242 | 4.0E-99 |
| **76** | 7180001691595 | Pseudomonas entomophila L48 | 1293 | 0.0 |
| **77** | 7180001691572 | Pseudomonas fluorescens A506 | 1197 | 0.0 |
| **78** | 7180001703584 | Pseudomonas fluorescens A506 | 1082 | 0.0 |
| **79** | 7180001710297 | Pseudomonas fluorescens A506 | 1119 | 0.0 |
| **80** | 7180001711155 | Pseudomonas fluorescens A506 | 1080 | 0.0 |
| **81** | 7180001714218 | Pseudomonas fluorescens A506 | 1792 | 0.0 |
| **82** | 7180001715123 | Pseudomonas fluorescens A506 | 1237 | 0.0 |
| **83** | 7180001730883 | Pseudomonas fluorescens A506 | 1080 | 0.0 |
| **84** | 7180001732206 | Pseudomonas fluorescens A506 | 1386 | 0.0 |
| **85** | 7180001733386 | Pseudomonas fluorescens A506 | 1158 | 0.0 |
| **86** | 7180001705442 | Pseudomonas fluorescens Pf0-1 | 1259 | 0.0 |
| **87** | 7180001706267 | Pseudomonas fluorescens Pf0-1 | 1823 | 0.0 |
| **88** | 7180001708341 | Pseudomonas fluorescens Pf0-1 | 1065 | 0.0 |
| **89** | 7180001708532 | Pseudomonas fluorescens Pf0-1 | 1090 | 0.0 |
| **90** | 7180001717386 | Pseudomonas fluorescens Pf0-1 | 1104 | 0.0 |
| **91** | 7180001717512 | Pseudomonas fluorescens Pf0-1 | 1302 | 0.0 |
| **92** | 7180001717741 | Pseudomonas fluorescens Pf0-1 | 1150 | 0.0 |
| **93** | 7180001718925 | Pseudomonas fluorescens Pf0-1 | 1140 | 0.0 |
| **94** | 7180001734233 | Pseudomonas fluorescens Pf0-1 | 1211 | 0.0 |
| **95** | 7180001734398 | Pseudomonas fluorescens Pf0-1 | 1190 | 0.0 |
| **96** | 7180001692018 | Pseudomonas protegens CHA0 | 1065 | 0.0 |
| **97** | 7180001698121 | Pseudomonas protegens CHA0 | 1235 | 0.0 |
| **98** | 7180001711853 | Pseudomonas protegens CHA0 | 1061 | 0.0 |
| **99** | 7180001695497 | Pseudomonas putida DOT-T1E | 1248 | 0.0 |
| **100** | 7180001695492 | Pseudomonas putida GB-1 | 1130 | 0.0 |
| **101** | 7180001695493 | Pseudomonas putida GB-1 | 1098 | 0.0 |
| **102** | 7180001704969 | Pseudomonas resinovorans NBRC 106553 | 1099 | 9.0E-90 |
| **103** | 7180001699625 | Ramlibacter tataouinensis TTB310 | 1040 | 1.0E-38 |
| **104** | 7180001715443 | Rhizobium leguminosarum bv. trifolii WSM1325 | 1195 | 6.0E-02 |
| **105** | 7180001691588 | Albidiferax ferrireducens T118 | 1045 | 4.0E-38 |
| **106** | 7180001698272 | Albidiferax ferrireducens T118 | 1268 | 2.0E-171 |
| **107** | 7180001699066 | Serratia liquefaciens ATCC 27592 | 1080 | 0.0 |
| **108** | 7180001730888 | Serratia proteamaculans 568 | 1300 | 0.0 |
| **109** | 7180001767298 | Shewanella baltica | 1034 | 3.0E-02 |
| **110** | 7180001697146 | Novosphingobium sp. PP1Y | 1047 | 4.0E-78 |
| **111** | 7180001719800 | Stenotrophomonas maltophilia D457 | 1334 | 0.0 |
| **112** | 7180001699804 | Stenotrophomonas maltophilia JV3 | 1115 | 0.0 |
| **113** | 7180001700805 | Stenotrophomonas maltophilia JV3 | 1022 | 0.0 |
| **114** | 7180001713726 | Stenotrophomonas maltophilia JV3 | 1009 | 0.0 |
| **115** | 7180001691601 | Stenotrophomonas maltophilia K279a | 1123 | 0.0 |
| **116** | 7180001699840 | Stenotrophomonas maltophilia K279a | 1376 | 0.0 |
| **117** | 7180001702909 | Stenotrophomonas maltophilia K279a | 1117 | 0.0 |
| **118** | 7180001703469 | Stenotrophomonas maltophilia K279a | 1215 | 0.0 |
| **119** | 7180001713625 | Stenotrophomonas maltophilia K279a | 1139 | 0.0 |
| **120** | 7180001691620 | Stenotrophomonas maltophilia R551-3 | 1134 | 0.0 |
| **121** | 7180001691715 | Stenotrophomonas maltophilia R551-3 | 1078 | 0.0 |
| **122** | 7180001692044 | Stenotrophomonas maltophilia R551-3 | 1544 | 0.0 |
| **123** | 7180001696343 | Stenotrophomonas maltophilia R551-3 | 1174 | 0.0 |
| **124** | 7180001698764 | Stenotrophomonas maltophilia R551-3 | 1112 | 0.0 |
| **125** | 7180001699175 | Stenotrophomonas maltophilia R551-3 | 1318 | 0.0 |
| **126** | 7180001702274 | Stenotrophomonas maltophilia R551-3 | 1882 | 0.0 |
| **127** | 7180001703759 | Stenotrophomonas maltophilia R551-3 | 1230 | 0.0 |
| **128** | 7180001706296 | Stenotrophomonas maltophilia R551-3 | 1652 | 0.0 |
| **129** | 7180001707760 | Stenotrophomonas maltophilia R551-3 | 1042 | 0.0 |
| **130** | 7180001707924 | Stenotrophomonas maltophilia R551-3 | 1375 | 0.0 |
| **131** | 7180001713350 | Stenotrophomonas maltophilia R551-3 | 1364 | 0.0 |
| **132** | 7180001713973 | Stenotrophomonas maltophilia R551-3 | 1389 | 0.0 |
| **133** | 7180001720643 | Stenotrophomonas maltophilia R551-3 | 1067 | 0.0 |
| **134** | 7180001697964 | Verminephrobacter eiseniae EF01-2 | 1363 | 7.0E-47 |
| **135** | 7180001703051 | Verminephrobacter eiseniae EF01-2 | 1229 | 0.0 |
| **136** | 7180001699990 | Xanthobacter autotrophicus Py2 | 1032 | 0.0 |
| **137** | 7180001694788 | Bacillus | 1040 | 2.0E-41 |
| **138** | 7180001694799 | Bacillus | 1319 | 5.0E-69 |
| **139** | 7180001695508 | Bacillus | 1453 | 9.0E-61 |
| **140** | 7180001701798 | Bacillus | 1137 | 2.0E-23 |
| **141** | 7180001704400 | Bacillus | 1450 | 7.0E-47 |
| **142** | 7180001705088 | Bacillus | 1059 | 1.0E-24 |
| **143** | 7180001717872 | Bacillus | 1257 | 2.0E-41 |
| **144** | 7180001753746 | Bacillus | 1846 | 1.0E-32 |
| **145** | 7180001691764 | Acidovorax | 1195 | 9.0E-80 |
| **146** | 7180001691574 | Sideroxydans | 1096 | 1.0E-126 |
| **147** | 7180001758503 | Bovine herpesvirus | 1481 | 0.0 |
| **148** | 7180001773904 | Bovine herpesvirus | 2103 | 0.0 |
| **149** | 7180001827628 | Bovine herpesvirus | 1286 | 0.0 |
| **150** | 7180001876321 | Bovine herpesvirus | 1117 | 0.0 |
| **151** | 7180002000232 | Bovine herpesvirus | 3417 | 0.0 |
| **152** | 7180001701723 | Acinetobacter | 2205 | 3.0E-70 |
| **153** | 7180001701726 | Acinetobacter | 1526 | 6.0E-53 |
| **154** | 7180001702962 | Acinetobacter | 2270 | 3.0E-45 |
| **155** | 7180001702982 | Acinetobacter | 1212 | 0.0 |
| **156** | 7180001705725 | Acinetobacter | 1511 | 6.0E-57 |
| **157** | 7180001706662 | Acinetobacter | 1456 | 1.0E-151 |
| **158** | 7180001707276 | Acinetobacter | 2025 | 0.0 |
| **159** | 7180001707407 | Acinetobacter | 1218 | 3.0E-41 |
| **160** | 7180001712918 | Acinetobacter | 1901 | 9.0E-65 |
| **161** | 7180001715003 | Acinetobacter | 2141 | 0.0 |
| **162** | 7180001715158 | Acinetobacter | 1338 | 2.0E-63 |
| **163** | 7180001731947 | Acinetobacter | 3224 | 1.0E-110 |
| **164** | 7180001691874 | Pseudomonas | 1556 | 0.0 |
| **165** | 7180001696401 | Pseudomonas | 1660 | 1.0E-81 |
| **166** | 7180001700334 | Pseudomonas | 1212 | 4.0E-36 |
| **167** | 7180001721485 | Pseudomonas | 1119 | 2.0E-54 |
| **168** | 7180001697934 | Azospirillum | 1085 | 5.0E-88 |
| **169** | 7180001695500 | Sphingobium | 1177 | 1.0E-61 |
| **170** | 7180001692186 | Strenotrophomonas | 1365 | 6.0E-71 |
| **171** | 7180001701108 | Strenotrophomonas | 1059 | 4.0E-50 |
| **172** | 7180001711200 | Strenotrophomonas | 1159 | 3.0E-41 |
| **173** | 7180001719296 | Strenotrophomonas | 1051 | 2.0E-26 |
