# Supplementary material for: Unexpected cross-species contamination in genome sequencing projects
Source: PeerJ. 2014 Nov 20;2:e675. doi: 10.7717/peerj.675 (PMC4243333; doi:10.7717/peerj.675)
Supplement: Supplemental Information 2 [file peerj-02-675-s002.docx]

>176 |ref|NC_017511.1| Neisseria gonorrhoeae TCDC-NG08107 | Coordinates: 2141271,2154835 | Forward

ATAAATTTTTGCACGGGTTGTGGATAAAATATCGGCGAGTCGGTATAATCGGTTCGCTGCGTTTTGAACCGACGCGTATTCAACAGATTTGTTTTCTTTTTGAAAATATTATATTTTCTTTGTTTTCGATTTCATTTTTACCGATTCGAGCCTATCCATGACATTAGCAGAGTTTTGGCCGCTGTGCCTCCGCCGTCTTCACGATATGTTGCCTCACGGGCAGTTTGCGCAATGGATTGCGCCCCTTACGGTTGGTGAGGAGGGTGGCGTATGGGTGGTGTACGGCAAGAACCAGTTTGCCTGCAATATGCTCAAGAGCCAGTTTGCCGGAAAAATAGAAGCGGTGAGGGAAGAGTTGGCTGCCGGCCGTCCCGCCTTCGTATTCAAACCGGGAGAAGGCGTGCGTTATGAGATGGCGGCGGTTGAAGGTGCTGTCGAACCTGCCGAGCCGTCCTTGCACGCGGGGTCGGAGGAGATGCCCGTGCAGGAGGTTCTGTTGGACGAGCTGCCGTCTGAAAAGCCTGTCAAACCCGCTGCGTCGAAAACGGCGGCGGATATTTTGGCGGAACGTATGAAAAACCTGCCGCACGAGCCGCGTCAGGCTGCCGGGCCTGCTTCCCGGCCGGAATCGGCGGCAGTTGCCAAAGCGCGGACGGATGCGCAGCGTGATGCGGAAGAAGCGCGTTACGAACAAACCAACCTGTCTCCGGATTACACGTTTGATACGTTGGTAGAAGGTAAGGGCAACCGCCTTGCGGCGGCTGCGGCGCAGGCGATTGCGGAAAACCCGGGGCAGAGTTACAACCCGTTCTTCCTGTACGGCAGCACGGGTTTGGGCAAAACCCACCTTGTGCAGGCGGTCGGCAACGAGCTGTTGAAAAACCGTCCCGATGCCAAAGTGCGCTATATGCATTCGGACGACTACATCCGCAGCTTTATGAAGGCGGTTCGCAACAATACCTACGACGTGTTCAAGCAGCAATACAAGCAATACGACCTGCTGATTATCGACGATATTCAGTTCATCAAAGGAAAAGACCGTACGATGGAAGAATTTTTCTATCTGTACAACCATTTTCACAATGAGAAAAAACAGCTCATCCTCACTTGCGATGTTTTACCCGCCAAAATCGAAGGTATGGACGACCGCCTCAAATCCCGCTTTTCGTGGGGGCTGACTTTGGAACTCGAGCCGCCCGAATTGGAAATGCGTATCGCCATTTTGCAGAAAAAGGCGGAAGCGGCGGGCATCAGTATCGAAGACGAAGCCGCGCTGTTCATTGCCAATCTGATCCGTTCCAACGTGCGCGAACTGGAAGGCGCGTTCAACCGTGTCGGAGCGAGCAGCCGCTTTATGAACCGTCCCGTCATCGACATCGATTTGGCGCGTACCGCTTTGCAGGACATTATTGCCGAGAAGCACAAAGTCATCACCGCCGACATCATCATCGATGCGGTGGCGAAATATTACCGCATCAAAATCAGCGACGTACTCGGCAAAAAACGCACGCGCAACATTGCCCGTCCGCGTCAGGTTGCTATGAGCCTGACCAAAGAATTGACCACTTTGAGCCTGCCGTCTATCGGCGATTCGTTCGGCGGACGCGACCATACGACCGTCATGCACGGCATCAGGGCGGTGGCGAAACTGCGCGAGGAAGACCCCGAGTTGGCGCAGGATTACGAGAAACTGCTGATTCTGATTCAAAACTGACCGGACACGCCTTTCAGACGGCATGACATTGACCATGCCGTCCGAAGGGTAGGAAATCCAACCGATTTAAGGAGCGAAAATGTTGATTTTACAAGCCGAGCGCGACAGCCTGCTCAAGCCGCTGCAAGCCGTTACCGGCATCGTCGAACGCCGACACACCCTGCCCATCCTGTCCAATGTGCTGATTGAGGGCAGGGGCGGTCAGACCAAACTCTTGGCAACCGATTTGGAAATCCAAATCGACACCGCGGGTCCCGAGGGAGGTGCGGGCGACTTCCGCATCACTACCAATGCCAAGAAATTTCAGGACATTTTGCGCGCGCTGCCTGCCGGTGCGCTGGTGTCGCTGGATTGGGACGACAACCGTCTGACGCTGAAGGCGGGCAAATCGCGTTTTGCCCTGCAAACCCTGCCTGCCGCCGATTTTCCGATGATGAATGTCGGCGAGGACATCAGCGCGACTTTCTCGCTGGGGCAGGAGCGTTTCAAAACCATGCTGTCGCAAGTGCAGTACAGCATGGCGGTGCAGGACATCCGCTATTATCTCAACGGTCTGCTGATGCAGGTTGAGGGCAGCCAGTTGCGCCTTGTGGCGACCGACGGACACCGCCTTGCCTATGCAGCCTGCGCGATTGATGCGGATTTGCCGCGCGCCGAAGTGATTTTGCCGCGCAAAACGGTGCTGGAACTGTTCAAACTGTTGAACAACCCCGACGATCCGATTCAAATCGAGCTGCTGGACAAGCAGGTGCGTTTCCAATGCAACGGCACGACCATCGTCAGCAAGGTCATCGACGGCAAATTCCCCGATTTCAACCGCGTGATTCCTTTGGACAACGACAAGATTTTCGTGTTGTCCCGTGCCGAACTTTTGGGCGCGCTGGAACGTGTGTCCATTCTTGCCAACGAAAAATTCCGTGGCGCGCGCCTGTTCCTGCAACCCGGCCTGTTGAGCGTCGTGTGCAGCAACAACGAGCAGGAAGAAGCGCGCGAAGAAATCGAAATCGCCTATCAGGGCGGCGAACTCGAAGTCGGTTTCAATATCGGCTATTTGATGGACGTGTTGCGCAACATCCATTCCGACGATATGCAGCTTGCCTTCGGCGACGCCAACCGCTCGACGCTGTTTACCGTGCCGAACAATCCGAATTTCAAATATATTGTGATGCCGATGCGGATTTGACGGTTTTCCGGAACACGATGCCCGTATTGGAGATATGCCCCGAACCGTGCAGACGGATTCGGGGTTTTGTTCGGCTGCCGGAAAGGCAATGCCGTCTGGAATGCGGCGGATTGGGGTTGGGAGCGTATGGGGGAAGTGCTTGTGCGGGTCAGCCTCGGAGCAAATCCCATAAATCGTTTTGCAGGTCGGCTTCGCTTTCGCCTTCGGCGGGCGCGGCGCGGATATAGCCGCCGTCGGGCTGCATCAGCCACGCGTGGGTATTGTCTGCCAGTGCCATTTCCAAACCTTCGCGGATGACGCGTTTTTTGAGTTCGGGCGCGGTAATCGGGGTGGCGGTTTCGATGCGGCGGAAGAAATTGCGCCCCATCCAATCCGCGCTGGAAATAAAGGTGTCGTCCGCGCCGTTGTTGTGGAAACAATATACGCGCGCGTGTTCGAGCTGCCTGCCGACGATGGAGCGGACGCGGATGTTTTCGGACAAGCCTTTTACACCCGGGCGCAAGGTACACATACCGCGCACAATCAAATCGATTTGCACGCCTGCCGCGCTTGCCCGATACAGGGCTTCTATGACAGTCGGTTCGATGAGCGAATTCATCTTGGCGGTAATCCGCGCCGGTTTGCCGGCTTTTGCGTGTTCGGTTTCGCGAGCGATGCGGCCGATAACCATTTTGTGCAGGGTAAACGGACTTTGGTAGAGTTTGTTCAGCCGCCCGGGTTTGCCCAAGCCTGTGATTTCCATAAATAATGTGTTCACGTCGGCGGTGATTTGTTCGTCGGCGGTAATGAGGCCGAAGTCGGTGTAGATGCGCGATGTGCCTTGGTGGTAGTTGCCCGTGCCGAGGTGGGCGTAACGCTTGAGCACGCCGTCTTCGCGGCGGATGACCAGTGCCATTTTGGCGTGGACTTTGTAGCCGAACACGCCGTACACGACGTGCGCGCCCGCCTCTTCGAGCTGCTTCGCCCAGTTGACGTTGTTGGCTTCGTCAAAACGCGCCATCAGTTCGACGACGACGGTTACTTGTTTGCCGGCGAGTGCCGCCTTCATCAGGGCGGGGACGAGTTCGGAGCGCGTGCCGGTGCGGTAAATCGTCATTTTGACGGCAAGAACGGCGGGATCGGCGGCGGCTTCGCGTATCATATCGACCACGGGATCGAAAGATTGGTAGGGGTGGTGCAGCAGGATGGGCGATTGGCGCACCAAATCGAAGATCGGGCTGTTTTTGCCCAAGGCTTTCAGACGGCCCGGCGTGTGCGGCGGAAATTTCAAATCGGGGCGGTTGACTAGGTCGGGGACGGCGTTGAGGCGGACGAGGTTGACCGGGCCTTTGACCTGATAGAGTTCGGCGTCGGTCAGCCTGAATTGCGCGAGCAGAAAGTCGCGGATGTAGGCGGGACAGGTGTCGGCGACTTCGAGCCGCACGCCGTCGCCGTATTCGCGGTCGTGCAGTTCGTTTTGAATGGCGGCGCGGAGGTTTTGTACGTCTTCTTCGTCAACGGTCAAGTCGCTGTCGCGCGTGAGGCGGAACTGGTGGCAGCCTTTGACGTTCATGCCCGGGAAGAGTTTGCCGACGTGGGCGTGGAGGATGGACGACAGGAAGACGAAGCCGTGTCCGCCGCCACACAGTTCGGACGGCAGGGGAACAACGCGCGGCAGGATGCGCGGTGCTTGGACAATCGCCATACCCGAAGGCCTGCCGAACGCGTCCGTGCCGTCGAGTTCTACGGCGAAGTTGAGCGATTTGTTCAGCGGGCGCGGGAAAGGGTGGGAAGGGTCGAGTCCGATGGGGGTCAGGATCGGCAGCAGTTCGCGGTCGAAATAGTCTTCGATCCATTTTTTCTGCGTGCCCGTCCAATTGCGGCGGCGGTAAAAATGGATGCTTTCCCGCGCCAACTCGGGCTGAAGGACGTTGTTGAACAGGTCGTACTGGTGCCGTATCAGGGAGCGCGCCGCTTCGGTAACGTCGGCGATGGTTTCAGACGGCGTTTTGCCGTTGTCCGGCCTGCGCCGGGGGTGCAGCTTGTTTTCACGCTTGAGCCACGCCATGCGGACTTCAAAAAACTCGTCAAGATTGGACGACACGATGCACAGGAAGCGCAGGCGTTCCAAAAGGGGGACGTTTTTGTCTTCCGCCTGTGCCAACACGCGGCGGTTGAATGCCAGCAGGCTCAGTTCGCGGCAGAGGATGCGGTTTTGTTCGGTCATAAGGTTCTCCCAAAGATGGTTGTTGTTCGGTCGGAGCGGGCGGACAATGCCGTCTGAAGGCTTGGCGGCATCTGCGCCGGTTTCAGGCGGCATGACGTGACGGACGGTCTGTTTCAGGCATCGGGGCAATGTGAAAACGGATACCGGTACGTGCGGTATCCGTCTGTTTCAAATCACTTCCAACACAAAATAATGACGCAGTTTTTCGTACACCGCATCGCTGACGTTGATGCAGCCGTTGGTCATAATCCTGTCGGACACGGACGGGGAGGCGATGCGTTCGTTTCGCCTTTCGGACGGTATCTGATTCCAAACGCGGTGCAGGGCGAAAAGAAAATCGCCCTCCTGCTTGAAACCGATGACTTCGCCGCCGTAACCGGGCTTGTCGGTACTGTTCAGCGTCAGTCCGAACGTCCCCTTGGGTGTGGCAGTGCCGATGAGGACGGGGTGGCACTGACGGTCGTCGGCAAAACAGAGTTCCGCCTTGGATGTGTCGACGATGACTTTTTTCTTTTGAATATAGGCACTGACCGCATCCGGCTGCCCCTGTGCGAATACGGGGGCAGTCGTCAGCAGGCAGCACAATATCCCTGAAAGCAGGCGGGGCGGCATAGCGGCTTATTGGCGGATACGTTTCGGTTTTGCCGGCACGATTTCGCGGATGATGACTTGCGGCTCGGCTTTGGGTGCGGGCGGCGGGCAGACGGCATCTTTCGGGAAGACGGGGTTCCAGTAGAAGCTGCGGGCAAATTTGTCTTTATCGAAAATCACTTTGTATTGGCAGGTAGTAACGCCTTCCACGCCGGAAGTGTTTTCAGGGTCGATACCCACGCCCGGGGTGTGGAAGTGGAACAGGTAATCCCATTCGCGCACGCCGTACATACCTTCGTCGTAATGCGGGCGGCCCGGGATTTTGTAGATGTCGTCTTTGGTCAGACCGGGGCGCATCTGATCCAGTTCGTCATAAGTCGGGAATGTGCCGCGCTTGTTGTCGAGCGTTACGGAATAGGGTTCCGGGAAAACCGGATTGTCGGTCGTGCCGTCGGCTTTGACGTTGCTTTATGGTTGCGCAGGCAGACAGAATGCCTGCCGCCAATACTGCCAAGCTCGGTTTGACGATTTGTTTGATTTTCATATGCAGAATCCTTTTTTACCCGATGCCCGTCTGCCTTTTGTTCGGAAGAGCCGCATCAGGAGAGTTTTAAACGTTTGATTTTGGTTCGTAATATTAGCATAAAAAACGTGCGTATCAGTAAACGCGGTGTATTTGTACGGCATACGGAATGATGCGCGTGCGAATTTACGCATCCTGCCGGCAATTTGCCGATTCGCCGACATCGGCAACCTGTTATAATTCCTCCTTTAAATTCCTAACGTTTTCAAGCGAAAAACAGAATGACCATGCAAGAACATTACCAGCCCGCCGCCATCGAGCCTGCGGCGCAGAAAAAATGGGACGACGCCCGTATTTCCAACGTCTCCGAAGACGCTTCCAAACCCAAATATTATTGCCTTTCGATGTTCCCTTACCCTAGCGGCAAGCTGCATATGGGGCATGTACGCAACTACACCATCGGCGACGTATTGAGCCGCTTCAAACTTTTAAACGGCTTCAACGTTATGCAGCCTATGGGTTGGGACGCGTTCGGCATGCCGGCGGAAAATGCGGCGATGAAAAACAACGTCGCCCCCGCCGCTTGGACCTACGACAACATCGAATACATGAAAACCCAGCTCAAAAGCCTGGGTTTTGCGGTTGACTGGGAACGCGAAGTCGCCACCTGCAAACCCGAATACTACCGCTGGGAACAATGGCTGTTTACCAAGCTGTTTGAAAAAGGCATCGTCTATCGCAAAAACGGCACGGTGAACTGGGACCCGGTCGACCAAACCGTCCTTGCCAACGAGCAAGTCATCGACGGACGCGGCTGGCGTTCGGGCGCGTTGATCGAAAAACGCGAAATCCCGATGTATTACTTCAAAATCACGGATTACGCCGAAGAGCTGCTCAACGATTTGGACAAGCTGGAACACTGGCCGGAACAAGTCAAAACCATGCAGCGCAACTGGATCGGCAAATCTCGCGGCATGACCGTGCGCTTCGCCGTTTCAGACGACAGCAAGCAAGGTTTGGAAGGCGATTACGCGAAATTCCTGCAAGTTTATACCACCCGCCCCGACACGCTGATGGGTGCGACTTATGTCGCTGTTGCCGCCGAACATCCGCTGGCAACAGCCGCAGCCGCCGACAAACCCGAATTGCAGGCATTTATCGCCGAATGCAAAGCCGGTTCGGTTGCCGAAGCCGATATGGCGACGATGGAGAAAAAAGGCGTGCCGACCGGCCGCTACGTCGTCAACCCGCTCAACGGCGACAAGCTGGAAGTGTGGATTGCCAACTATGTCTTGTGGGGCTACGGCGACGGCGCGGTGATGGCTGTTCCGGCGCACGACGAACGCGATTTCGAGTTTGCCGCCAAATACAATCTGCCGAAAAAACAAGTCATTGCCGTCGGCGACAACGCATTCGACGCAAACCGATGGCAAGAATGGTACGGCGACAAAGAAAACGGCGTATTGGTCAACAGCGGCGACTTGGACGGCTTGGATTTTCAGACGGCATTTGATGCCGTTGCCGCCAAGCTGCAAAGCCAAGGTGCGGGCGAACCGAAAACCCAATACCGCCTGCGCGACTGGGGCATTTCGCGCCAACGCTACTGGGGCTGCCCGATTCCCATCGTCCATTGCGAAAAATGCGGAGACGTTCCCGTCCCTGCCGACCAACTGCCCGTCGTCCTGCCTGAAAACGTCGTACCCGACGGTATGGGTTCGCCGCTGGCAAAAATGCCCGAGTTTTACGAAACTTCCTGCCCGTGCTGCGGCGGCGCGGCGAAACGCGAAACCGACACCATGGACACCTTCATGGAGTCGAGCTGGTACTTCTTCCGCTATATGTCGCCCAAGTTTTCAGACGGCATGGTATCGGCAGAATCCGCGAAATACTGGGGCGCGGTCGACCAATACATCGGCGGCATCGAACACGCGATTTTGCACCTCCTGTACGCGCGCTTCTTCACCAAACTGATGCGCGACGAAGGTTTGGTCAATGTTGACGAACCGTTTGAACGCCTGCTCACGCAAGGTATGGTCGTCTGCGAAACCTACTACCGCGAAAACGACAAAGGCGGCAAAGACTGGATCAACCCCGCCGATGTCGAGCTGACTTTCGATGACAAAGGCCGCCCCGTTTCCGCCGTCCTCAAAGCCGACGGACTGCCCGTCGTCATCAGCGGCACGGAAAAAATGTCCAAATCCAAAAACAACGGCGTCGATCCGCAAGAACTGATTAACGCCTACGGCGCGGACACCGCCCGCCTGTTCATGATGTTCGCCGCACCGCCCGAACAGTCCCTCGAATGGAGCGACAGCGGCGTCGAAGGTGCACACCGCTTCCTGCGCCGTCTGTGGCGTACCGTTTACGAATACCTGAAGCAAGGCGGCGCGGTCAAAGCATTTGCAGGCAACCAAGACGGTTTGTCTAAAGAACTCAAAGACCTGCGCCACAAACTGCATTCCACCACCGCCAAAGTCAGCGACGACTACGGCCGCCGCCAGCAGTTCAACACCGCCATCGCCGCCGTGATGGAACTGCTCAACCAATACGACAAAACCGACACCGGCAGCGAACAAGGCCGCGCCGTCGCCCAAGAAGTATTGGAAGCCGCCGTACGCCTGTTGTGGCCCATCGTGCCGCACATCTGCGAAACCCTGTGGAGCGAATTGAACGGCGCGAAACTGTGGGAAGCAGGCTGGCCGACAGTCGACGAAGCCGCCCTGGTCAAATCCGAAATCGAAGTGATGGTTCAAGTCAACGGCAAACTGCGCGGCAAAATCACCGTCGCCGCCGACGCCTCCAAAGCCGACCTCGAAGCCGCCGCACTCGCCAACGAAGGCGCAGTGAAATTCATGGAAGGCAAGCCTGCGAAGAAAATCATCGTCGTACCGGGCAGACTGGTGAACATCGTCGTCTAAACCGCTTTTAAGGTTTAGCCATACGGATAAAGGCCGTCTGAAACTTGGAAACAGGGTTTCAGACGGCCTTTTTAAGGCAGATTGGGTTGTCTGCAAGACAGACCTCAAATATAGCGGATTAACTTTAAACCGGTACGGCGTTGCCCCGCCTTGCCCTGCTGTCTGCGGCTTCGTCTCCTTGTCCTGATTTTTGTTAATCTACTAAAAATTAATCTCAAAATCCAAAATATGAATTTATTTTTCGATACCCAATTGGGAAAGCAACAAAATAAAGCAACCCACAAAATCCGTGTAATGAGCGAGGCTTGGCTGGAAAAAAACGGCTATTGCCCCTGTTGCGGAAGCAAGCCGATGCAGAGATTTGCCAATAACAAACCTGTTGCAGACCTCTTTTGCCCAAATTGCCACGAGCAATATGAATTAAAGAGTAAAAATCAAAAAACCATAGGCAACAGCGTGCCTGACGGTGCATATCGCACTATGTTGGAGCGCATCCGGTCAGATACCAACCCCAACTTTTTCTTTCTTGCATATAAAAAAGCGGATTACTCCATACGGCAATTGGTGCTTGTACCCAAACATTTCATCACGCCGGATATGATTATTCCCAGAAATAAAGGCATTAAAAACCGACCGCACCACATTATGTGTTCCATCAATCTCGCCCCTTTGCCTGAAAGCGGAAAAATATTCTTAATAGACGATTCCCGCATTATCGAACCCGAAACCGTTCTGAAAAAATGGCAATCCAACCTGTTTTTACGCAACCAAAATGCGGAGCGCAAAGACTGGCTTTTGGCTGTTATGAAATGTATCGACCAACTCACCGAAGAATTCACATTGTCGCAAATGTATGAATTTGAAAACAAACTATCCATCCAATTTCCCCAAAACAACCATATCAAAGACAAAATCCGCCAACAGTTGCAAATTTTGCGTGATCAAAATATGATCGAATTCATTGGTCGCGGACTTTACAAAAAAATCGACAAATTGCACCCAACTCCCAAGGCGTTTTGATTTCAAATCATGATACTGAATTTACGAGAAATATTTATTCTTCTGCCATTCTAAAAACGGTTGAAGTACAACGCAATATTGCCGCAAAGGGCAGCAGTCGTAAAAAAGTCGGAGAATTATTGGCAATTTATGATTGATTTTGACAAACCGGCTGAAGAAGCTGCCATTTATCAAAGTCGTTTGAAAAAATCGTTTCAGACGACCTTGTCCTCTACAACGAAAATTCCCTTAACGTCATGCGGAAGATATTGGAAAAACATCCAAACGGCTGTTTTGATATGATTTTCGCAGACCCGCCTTACTTTCTTTCCAATGACGGTTTCAGTTGTCAAAACGGGCAAATGGTTTCCGTCAATAAGGGCAACTGGGATAAATCCAAAGGAATGGCGGCAGATTTGGAATTTTACGAAGAATGGCTCCGACTGTGTTACGCCTTATTAAAACCAAACGGCACAATTTGGGTTTGCGGCACATTTCATAATATCTATTTAATCGGCTACCTGATGCAAACCGTCGGCTACCATATTTTGAACAATATTACTTGGGAAAAACCCAATCCTCCCCCTAATTTGTCCTGCCGTTTCTTTACCCATTCGACAGAAACAATCTTATGGGCAAAGAAAAATAAAAAAGCCAAACATACGTTTCATTATGAAATGATGAAAGCACAAAATAATGGCAAACAGATGAAATGTGTTTGGACATTTCCTCCACCAAATAAAACCGAAAAAACATTCGGCAAACATCCGACACAAAAACCACTCTCCTTACTTGAACGCTGCATACTATCGGCTTCAAATATCGGAGATTTAATTTTTGACCCTTTTATGGGCAGCGGCACAACAGGCGTTGCCGCCTTAAAACATGGTCGGAGATTTTGCGGTTGCGAACTGGAAGAAGATTTTTTGAATTAGCAAAGAAAAGGTTAGAAAAATGATTATTGGCGGAATCGGCGGTGCAAGGACACAAACAGGACTCAGATTCGAAGAACGTACAGACTTACGAAAGTTGTTTGAAGAAATTCCCGGGTACGACTTAAGAAAAACAGATGATAATGCGGGTTATGAAGTTTGGTTTAATGGAGAATTGAAGGCTTATTGTTTCAAAAAATATGAGTTTTACCGATTTTTGGAAAGACTGGAATACAATATTAATTGGAAAGACCATCTGTCTAAAAGATTACTGCCCGATAATGGCTTATTTATCATCATCCGTGATACCTTATTTATTATTGAGATCAAATTCCAACAAACTCCCGGTTCAGTAGATGAGAAATTACAAACTTGCGATTTTAAGAGAAAACAATATACAAAGTTAGTTCACTCTTTAGGTTGGCGGGTTGGATATGTCTATGTTTTGAATGATTGGTTTACAAAACCGGAATATTAAAGATGTTTTAGATTATATTATCAGTGTTAACTGCCATTACCAATTTAACACGATTCCCTTAAGGTGGTTTGGACTGCCTGATGGTGAAACAAATGAATAAATACCATATTTAAAACACAACCGTTCAAGGTCGTCTGAATATCATTTCAGACGACCTTTCACCAATCCCCGCCGTTTTCCATCTTTCTGCTATTGTGATAAAGTAGCCCGACCTTTTGTTCAAAATACGCGGATTCCGCGACACTGAATGCAGCATCTGCCAAAGATTATTTGAAAACGGTCGAGTTAGACAAGTCTGCCGACAATGTCGATACCACATCCAAAACTATCCGCAGGGTTTAGGCGGTATTCCAGAGTACGGTGTCTTATGCTGATGCTGTAAACAATGCCCGTATAGAAAATCTAAAACGGTTGTTGCCGACCGTTATGCCGGTTTATGAGCAAAGTGTCAGAAATAAGGGGCGCGTTAATAAAAAACGTCGGCGTTAAGGGAAGGGGATCGAGAATTTGAGCCGTTGTTTCAAAATGCCGTCTGAAATCGTTTGAGATTCAGACGGCATTTAAACAGTTCTGCGCCCCGCCCGTTTTGCCTTCAAGCCCGCGCCGCTTAAATCCAATCCCCCCCCTGCAACGGGCGCAAAAAATCCCCGTGCCTCGCGGTCGGGGATGTTTCGGGTTTCGCGCCCGTCTGCGCCGTGCCTTGCTGTCGGGCTATGCCGTCTGTACCGCCGCCGTTTCGGCTTGGTTTGATGCGGTCGGGCATTTGTGCGGCTACGCCCTGATATTCAGTTCGGCGGTCTTGCCTGTCTTGGCAGGCATATTCAGCAGCGCGTCTATATGTGAACTTGCTGATTTTGCCGTTTGCCAAGTCGGCTACGCAATGGCTTCGATGCCCTTGTTTTTGACGATGGTCAGCAGCTTAAACGCTGCGCCGCCGGGCTTTTTAACGCCCCGCTCCCAAGCCGAAACGTGATTTTTCCCCACGTTGAGATAGATGGCGAAAGCGGCTTGCGATAGTGCCTCCTTCTCCCTGATTGCCTTGATGTCCCCGCCGCTCAACGGCTTGATTTCGGTCAGGCAGGACTTGTCAAAGCCGCGCATAGTCTTTTTGTCGATTGCGCCGATGTCGTGCAATCCCTGCATGGTTTCGTGTATTGCTGCGAAAATCCCGCTTTTATATTTCATGGCTGTATCTCCGTGAAAGTGCCGTTTGACTTGGCTTGCTCTATGTCGGTTTCTGTCATTGCCAAGATGATGGCGGCGGTTTTCTTTAATTCTGTAAGTTCTTTATCAGTGATGTTTTCGCGGTCGTTCTTGGCAAAGGCGGTCATGAAAAACGCCCGTTCTCCATGCCTGAAAAATATCAGACTGCGATAACCGCCGCTTCTGCCTTGCCCTTGCCTTGCTATACGCTGCTTAATCACGCGCCGCCCAAATCTGCATCTATCAGCCCGTTATCTGCCCGCTCTACCGCCTCTATCAGCTCTGAATCACTGATTTTGTGCTTCTTGGCAAATTTCACTATCCATTGGTTTTTGAATATCCGCATTGTGCAACCGTTTACTTAGTGATACTGGGGATTATACCAAAGACAGGCAACACGGCAAGCCGCCTTTTTATACCCTGCAATTTCTCCGTTTATGCCGCCTGAAACACCGGCGGCTAATTTGATTATTTTTTAATCAATCGCTTTTAATGGCTCAAATTGCGTTTTTAGCGCGTTTCGCTGTCTTCCTATGCCCCGCAATATAACCAACCGGTTTAACCGCCATTCCTGCTCAAAATAGGCGCGGTTCCGTGCGTTTTGGCTTTTCATTCCGCTACGCTCCCACTGCCAAGACGGTAAAAAACCGGAAAACCCCAAAAGTTGTTACCGATTCGTTACCATTTGTTACCGCATTTGTTACCAATTTTTTATCTTTTTCTTATTTTTATAGTGAATTAAATTTAAACCGGTACGGCGTTGCCTCGCCTTGCCGTACTATTTGTACTGTCTGCGGTTCGCCGCCTTGTCCCGATTT

>1 |ref|NC_017511.1| Neisseria gonorrhoeae TCDC-NG08107 | Coordinates: 1,24525 | Forward

AAATTTAATTCACTATATTTCATATACTTATTTATCTAACTTTTCCCCGGTAACAAGGTAACAACTAAGGGTATCGCTTCCTAGTATAGGTTTTTTCCAAAAACCCAAAAAAACCGCTCTGCATCTTGGACGGTTTCCCCCTCGTTCCCTACGGTTCGTCAGTATTCCCCGCAAGCTCTTTTCCGCTTGGCATTCCTGATTTGGTGGTCGTTCAGCTTTATTTTTGCGGTATTTTTCGGATACCGTAAAAAATCAGGTTAGTTCAATTAGGGCGGATTGGACGGGATTGCACAGTCAGGAAGGGGACGGCAGGATAACAATCAGCCTGAAATCCTTGTATTGATTGGGATTGGTTGATGGCATCAGACAGGATTAAACGAAAAAAAACGCCTAGAATTTCTAAGCGTTTTTGTGTGTTGGTGCCGACAGCGAGATTTGAACTCGCACAGCCTACGGCCACTACCCCCTCAAGATAGCGTGTCTACCAATTTCACCATGTCGGCATTTGAAAAACTGTTATTTCTGCTGCTGAGGAACAGGGGCAGAAGGTTCGGTATTGCTTACGGGTTTGGGTGCTTGCTGAGTCTGTCGTATGTTGCTGAAGTCCAAACCGTGTTTTGTCGTGTGGGTGTGAATATACACCATAGCCATGCAGGTTGCGAAGAAAAATGTTGCTGCAACGGCGGTCGAGCGGCTGAGGAAGTTGGCGTTGCCGGCAGAGCCGAATACGCCTTGCGCGCTGCCGCTTCCCGATCCGAAGGTCGCGCCGGCATCCGCGCCTTTGCCGTGTTGGAGCAATACTAACACGATGACGGCCAAAGCGGAAATAATATTAATAATCCAAATTAGGGTTTTGAAGGCTTCCATATTTTTCTACGCATTTTGTGCGGCACTGATGATGGCGGTAAAGGAGTCGTACGACAATGACGCGCCGCCGACGAGTGCGCCGTCCACATAAGGTACTGCGAAGATGTCGGCCGCGTTGTCCGCTTTCACACTTCCGCCGTAAAGGACGCGGATTTTAACATCGCTTCCGCACAAAGACAAGATTTCTTTGTAGATGAATGCGTGCATATCGGCAATCTGTTCGACGGTGGCGACTTTGCCTGTGCCGATTGCCCAGACGGGTTCGTAGGCGACGGCGATGTTTTTGGTATCCAGCCCTTGCAGGATGGAAAGCTGATGGGCGATGACTTCGTGTTCTTTGCCGGTTTCGCGCTCTTCGAGGCTTTCGCCGACGCACAATAATGGGATGAGTCCGACGTTGAGGACGTTTTCCATTTTGCGGCGTTGGATTTCGTTTTTTTCGCCGAAATAAAGGCTGCGTTCGGAGTGTCCGATGAGGACGATGTCTGTGCCGGTGTCGGCGAGCATTTCGGCGGACACTTCGCCGGTGTACGCGCCGTTGTCGGGGAAGCGGCTCACGTCTTGGGCGCAGGTGAGGATGCGGTTGTTTAAGACGATTTGCATGGCGTTGTGCAGTTGCAGCAGGTAAACGGTCGGGGCGGCGAGTCCGATGAGGACGCGTTCGGCGGTGGGGAGGATGCGGAAGCGGTGCATCAGTGCGTTGTTGTTTTGGAGCCGGCCGTTCATTTTCCAGTTGCCGATGACCCATTTTTGATCCCACATTCCGATTTGGCGATACATCTTTTTTGCTCCGTGTCGTGTTTTTCTGTCTGCCGCGTGTGGCGCGTTGCAATGTGAAGTTTAGTGGATATGCGGCGGGTTCGCAACTTGGGGCGGGCGGCTGCGGGGGCGGTTTGGAATGTTGTTTCGGGCAGGATGTTTTATAATGGCTGCCTGATATGTATGCAAATATGGGAGATATGATGCACGCGCTTCATTTTTCGGCTTCGGACAAGGCCGCGCTTTATCGTGAGGTGTTGCCGCAGATTGAGTCTGTGGTGGCGGACGAGGCGGATTGGGTGGCGAATTTGGCGAACACGGCGGCGGTTTTGAAGGAGGCGTTCGGCTGGTTGTGGGTGGGTTTTTATTTTGTCGATACGCGTTCGGACGAATTGGTTTTGGCACCGTTTCAGGGACCTTTGGCGTGTACGCGGATTCCGTTCGGGCGCGGGGTGTGCGGTCAGGCTTGGGCGAAGGGTGAAACGGTGGTTGTTAAGGATGTGAACGCGCATCCCGACCATATTGCCTGTTCGTCTTTGTCGCGTTCGGAGATTGTCGTGCCGCTGTTTTCAGACGGCCGCTGTATCGGCGTGTTGGACGCGGACAGCGAACATTTGGCGCAGTTTGATGAGGCAGATGCTTTGTATTTGGGCGAACTGGCGAAGATTTTGGAGAGGCGGTTTGAGGCTTCGAGTCAGGCGGCTTGAGACTGGCAAAACGGGCGGGCTTCGCGTGCCGAAGTTGGCGCGGCGGTAGTGTGGTTTTATAATGCCTGCCATTGATAAAACAATTATTTGACGGAGCACTAAATGGATTTTGAAAAAGCGCGGTTCAATATGGTCGAACAGCAGATCCGTCCTTGGGATGTATTGGATTTTGACGTGTTGGACGCTTTGGAGGAGATTCCGCGCGAGCTTTTCGCGGATGAGTCTTTGCAGGGTTTGGCGTATGCGGATATGGAGCTGCCGCTTGCCAACGGTCATAAGATGCTCGAGCCGAAAGTCGTGGCGCGGCTGGCGCAGGGCTTGAAGCTGACGAAAAACGATACGGTTTTGGAAATCGGCACGGGTTCGGGCTATGCGACGGCTCTGTTGGCAAAACTGGCTGGCCGTGTGGTTTCGGACGACATCGATGCCGAACGGCAAAAGCGCGCCAAAGCGGTTTTGGACGGCTTGTCTTTGGAAAACATCGATTATGTGCAAAATAACGGGTTGACCGAACTTTCCGCAGGTGCGCCGTTTGATGCGGTTTACGTCGGCGGCGCGGTAACCCTTGTGCCTGAAGTGTTAAAGGAACAGTTGAAGGACGGCGGGCGTATGGCGGTTATTGTGGGACGCAGGCCGGTGCAGCGCGCGCTTTTGATTACGCGCAGGGGCGATGTGTTTGAAGAGAAGGTGCTGTTCGATACTTTGGTGGCGCATTTGGACGACAAGGATGCCCATCCTTTCGACAGTTTTAATTTTTGATGTTCGGATTGTGATGCCGTCTGAAAGCGGGTTTGGGGCTTCAGACGGCATTTTGCTTGGTTTTTTTCGGGGGGTTTGTGATGGATATTGTGCAACTTCCGTCGGCGGCATTGAAGGCGTGGATGGACGAAGGGCGGATGTTTTGTTTGTTGGACGTGCGTACGGATGAAGAAGCGGCGGTTTGTTCGCTGCCAAATGCGCTGCATATCCCGATGAATCTGATTCCGCTGCGGCAAAACGAGTTGCCGGACGATGTGCCGCTTGTGGTGTATTGCCATCACGGTATCCGCAGCCTGCATACGGCGATGTATTTGGCGGAGGCGGGTTTTGAAAACCTGTACAACCTTCAGGGCGGCATCGACGCGTGGGCGGTTGAGGTTGACGCGGAAATGGCGCGGTATTGAAGGCGGCTTCAGACGGCATTCCTTAAATGCGTGTACCTCTGGTGTTCCATAAAGGTCGTCTGAAAGTGCAGCTTCTGCGAAGCTAAAGCGCAGTATCAACGAAGTTAAAATTTGCCTGAACCTTAAAGGCAGCCTGCACCCCAATTCCCTCGCCCCGTGGGAGAAGGCTAGGGAGAGGGCGGCAAACTGCAGGTTTGCTTGGGCGGCATTTTCAATGTGCAGGCTGCTTTTAGCTTCGCAGAAACTCCGTTTTCAGACGACCTTATTTAAACCGATAGGTAAACGCCGCGTTCACTGTCCGCAGTGCGCCGTGGCTGTGGCGGTCGGGCTGGGTGCGGTAGTGTTTGTTGAACAGGTTGTCCACGTTCAGCGACAGTTCGGTGCGCGGATTGAAGCGGTAACGCGCCATGATGTCGGCGACGGCGTAGGCTTTCTGGCGGCTGTTGTCGGCGGCGCGGGCTTTGGCGGCGGGATTGGGGATGCGGAGCGCGGCTGGGTCGGTGTGGGTTTCGCCCTGCCGGCGCACACCCGCACCGATGGTCCGGCCGCTGGGGGCTTCGGGGGCGAAGTGGTAGGCGGTGAAGAGTTTGAAGCTGCGTTCGGGTACGCTGTCGGGGTTCAGGCGGCTGCCGTCTTGGTCGCGGGGTTTGCTTTGGCTGTAGCCTGCCTGTATCTGCCATTCGGGCGTGATGCGGCCGCCGACTTCGATTTCCCAGCCGTGGGTTTTGGCTTGGTTGGCGGCGCGATAGTAGGTGTTGCCGCTCTGGTCGCGTCCTGCTGCGGTGGCGAGGTTGTTTTTACGGGCGCGGTACACGGCGGCGGATGCGTTCAGACGCCCTTCAAGCCATTCGCCTTTGATGTCGGCTTCCAGATTGTTGCCGGTTACGGGTTTCAGGTAGCTGCCGTGTTCGTCTTTTTGCAATTGCGGGACGAACAGGCTGCTGTACGAGCCGTAAAGCGACAGGTTGCCGGTCAGATCGAACACGATGCCTGTGTAGGGGGTGAAACGGTTGGCGGACACATAGGTCATGCCTTGTGTGCGGCTGTTGTAGCTGCCTGCGCGGTAGCGGCTGTATCTGCCGCCGAGTATCAGCGAAAGGTTGTCGGCGGCGCGGAAACGGGTGGCGAGATAGCCGCCGATTTGCCGCCTGGTGTCGTATTGCGGGATGGTTTGGGCAAACGATGATGGCTGCGGATAGGCGCCCGTGCGGGAAAATTCGTAGGCGTTGGGAATGGCGTTGGGAATGATGCTGCGTTCGCCGTATTTGTTGCTGGCGTATTTGTAGCCGTTGATACCCGCGATTAAATCGTGCTCGCGGCCGAACAGGCGGTATTTGCCGGTCAATGACATGCTGGCGCTGTGGGTGCGCGGATCGGCGTGCCAATAACCGGGAATCAGGTCGGTGGCGGCAGTGCTGTGGTCGATGGAAAGTACGCCTGCCACACCGTAGGGCTGGCGGAAGCGGCTACGGGTGTAGTCGTATTCGGCTTTGAGTTTCCAGTCTTGGTTGAAGCGGTGTTCTATGCCGGCGAACAGGTTGAGCGCACGGTTGCGGCTGTTCGACCAATTTGTGGCGGGGTTGTCTTTTGGGCCGAAGGCGGTGGCATAACCTTGGCTGTCGTACACGGCGTAGCTGAGCGGCGCGTCTGCGGTTTCTTTCGCCTGCTGGTAGTCCATGCCTGCGTGGACGCGGGTTTGCGGTGCGATGTCGTATTCCAAAATGCCGTAGAGTTCGGCATCGCGGCTGCGTTCGAGCTGCCGCCACGAGTCGCCGCGTCCGAAGGTGGAAACCAGGCGGCCGCGCAGCGTGCCTTCGGCGTTCAGGCTGCCCGATACGTCCGCGCCCAGCCCGAAATGTTTGCGGTTGCCGGCTTCGGCGCGGACTTCAAACAATGGCTTGCGGGTCGGGTGTTTGCGTACCAGATTGACGGTGGCAGAAGGCTCGCCCGTGCCGTCCGGCAGCCCCGCCACGCCGCGCACGACTTCTACGCGCTCATAGGCGGCGGTGTTGGCATTGCCCGTATCGGCCAGCGCGTCGGCAACGGGGATGCCGTTGATTTGGTAGTTGGCGATGCGGCTGCCGCGCGCGAACAGGTAGTTGTAGCCCGCGCGGTCGGAGCCGTAAATCTGGCGGCTGGTGCCGGTCGCCTGCAACAGGGCGCGGTCGAGCGTTTTGATGTTTTGGTCGCGCATTTGTTGCGATGTGATGACGCTGACGCTCTGCGGGATTTCGCGCAGGGTCATGGGCAGCCCGAACGGGGTGTGCGTGCCGGAAACGGTGTAGCCGTCGTTGGAACTCGCGGTGCGGTCGGCGGTAACGGTGATGGTCGGCAATTCGGTGCTTTCCTGCGGTTTGGGGTCGTCTGAAACAGAAACATCGGCCTGCGCGTACACGGGTAGCAGGGCGGCAAAAAGCAGGGAGTATTTGAAGCGTGTCATGGGGTTTCCTTTGTGTGTTGGTGTGCGGTGGGGTTGTATAGCGGATTAACTTTAAACCGGTACGGCGTTGTCCCGCCCCGGCTCAAAGGGAACGATTCCCTAAGGCGCCCAAGCACCGGGCGAACCGGTTCCGTACCATTTGTACTGCCTGCGGCCCGCCGCCTTGTCCTGATTTTTGTTAATCCTCTATGCTTTTGGCATATTGAAGGTGTAGGCTGCTTTTAGCTTCGCAGAAACTCAGCTTCCTTCGGAAACTTCGTTTTCAGGCGGCCTCGGACGGGTCGGGCAGATCAATCAGCACCACCGAGCAGCCGTTTTCCGAACGCGGGCGGTGCGACACCGACTTCGGCACGACCGCCATCTCGCCCTCGCGTATCGTCATGTTGCCGCCGTCGGCGAAGTCCACCGCCATGTCGCCCTCCACGGCAAACAGCACTTTGTCGCTGTATCCGTGCGTGTGCCAGCCGTAATCGCCCAAAAGTTTGACCAGGCGGAATTGGAACCCGTGGCGGTTGATGATTTCGGGCTGCCAGTATTCTTTGATGGCGGCAAGGTGCTGTTTCAGGTTGATGGTTTCGTTTTGCATGGGGTTCTCCTTGTGGTTTGTTTTGAATAAAACTACTGCGCCGCAACGGGTTCTGCCTTTTCAAACGCCGCCTTCAACTGTTCCGCCGCCTGTATCAACTGCCGCGCGCCGCCCGCGACAATGTAGTTCGCGGCAGGCATGACGATGATTTGCTTGCGCTTCCAAGCGTTCGTGCCGCATACCAGCGCGTTATCCAACACTTCCACGGCAGCCGGCCCTTCCTGCCCGATGGCGGCGGTGCGGTCGATGATGAAAATCCAGCCGGGGTTTTTCTCTTTGATGTATTCGAAGGAAACGGGCTGCCCGTGCCCTTCGTTGCGTAAAGATTCGTCCACGGGCGGCAGGCCGATGTCGCCGTGTATCCAACTTGCCAACCGCGATTGCGTGCCGAAGGCGGACACCTTGTTGCCTGTAACCGACAGCACCAGCCCGCGTCCTTTGCCTTTGGCGGCTTCGCGCTTTTGGGCGAACAGCGCGTCAATCTGCGCATTCAATTCCGCCACGCGCGCTTCCTTACCGAAAATCCGCGACAGGGTCTCCATCTGCTTCTCGCCGCTGGTGCGGATATTGCCGTTGTCCACCGTCAAATCTATGGTGGTCGCGTTTTTCGCCAACTGTTCATACGCTTCCGCACCCGGCCCGCCGGTAATGACAAACTGCGGATTGTGGCGGTGCAGGGATTCGCAATCGGGCTCAAACAGCGTCCCCACCGTTGCCGCCTTGTCAAATGCAGGCTGCAAATAGTCCACGCGCACCGGCGCGGTGGTTGCGCCCACATTCACGCCCGGCTCGGTCAGCGTATCCAACGCCGCCCAGTCGTACACGGCGACGCGTTCGGGATTCTTCGGCACAACGGCATCGCCCCGCGCGGTCGGCACGGTCAGCGTGGCGACAGGTGTGGATGCGGCTTGGGATGCGGCGGATACAGTTTTTTCGGCGGCAGGTTCGGGCGAACAGGCGGCCGGCAGGACGGCGCAGGCTGCCCAATAAAAACGCGGTTTCACGGTGTGCGGTTCCTTTGCATACGAATAAGAATGTTTATTATTAAGCAAACAACCTGCAGAAACCACGCCGCAAAACGCAAAATTCCCGATACGGCAAAACAAATTCCCGAAACGGAAAAGGTCGTCTGAAACACCCTTTCAGACGACCTTTTCCGTTCCAACCAATCCCTGCGCTTGATTATTGGTAATAATTCCTATTTAATTCATTTGTTAGACAACTCGTTCCTATCCAATCATGAACACCGCCGCCATCTACCGCCAGTACCAAACCTATGTCCGCTCCGATAAATCCGGCTGGGCGTTGGACGGCTGTTCCGACAGCGCACTCATTGCGCAGGCAAAACAGCCCGGTTTGCATCTGAAAATGTGCATCAACCGCTTCGACTCGGGCATCACCTTATCACGGATGCGCGGCGGCGGAACGGGTGCGTTTCCCACCGAAATCCACAATTTCAGCCACAACTGCGCCTTGTTCGTCATGGTATCGGGGCAGAACCGGTTACAAATGGGCGGCAGGGAATACCGCCCGTCTGCCGGCGAAATCTGGCTGGTACGCGGCGAACTGGCGGACGTATCCGAAACCCTGCTGCCAGACAGCGGCGGCATGTGTGCGCTGCATTTGGATTTTTGGCTGGAAAAACTGCGCCGCTGGCACGACGAAGGTTTACTGGACGAACGCCTGTTTTCGCCGCAAACGATAGGCCGATTCGCCCTGCAACGGCTGGCGCAAAACGCGGGGACACTGACGGCGGCGGCCTGCCCCCTGCTGCAACGCCCGTTCGAATCGGACGGCTTCGGCCTGCTCGCCGACGAAGCCGACGCGCTGGAACTCTCCGCCCGATTATTACGCTTCACCTTCCGCCGCCACGACAACGGCTACCGCCGCCGGCGCATAGACGAAGCCGCCGATATTTTGAACAGCGAATTCGCCCGCCCGCTGACCATCGCCGAAATCGCCCGCCGCGTCGGGCTGAACGAATGCTACCTGAAACGCTATTTCAAAGCGCAAACCGGCGAAACCGTCGCCGGACGCCTGCGCCGCCTGCGGCTGGAACACGCGCTCGCCCTCATCGAATCGGGCAGCACCATCCAAGCCGCGATGCACTTTTGCGGCTACCGCCACGCGGGACGGTTCAACGAAGCGTTCAGGCGGCATTACGGATTTTTGCCTTCGGATGTGAAAAAGTGCTGATGGGCCGACGGCATAGATATTTTGATTTGAAACAACCATTAAAAAAGATCGTCTGAAAAAGCAATTTCAGACGATCTTTTTTCATTCGCGGCGGCAGTGTCGCCCCCTGCCCTTATCCCAAAAATTCTTTCAAAAACAGCAACACGCAGGCGAGTTTGTCGGCGGATTCGCGTTGGGTGCCGTTGCCGGTATGGCCGCCGCCGTCAGGCGAGTAGAGCCAAGATTGCGGCGAGGTTTCGCGCAGTTTGGCGTAGAATTTGAGCGCGTGGGCGGGATGGACGCGGTCGTCGCTGAGGCTGGTGGTAATGAGTGCGGGCGGATAATCGATGCCGTCTGAAAGATTGTGATACGGCGACAATTCGCCCAGCCGGCGTTTGCAGGCTTCGTATTTCTGCGGATTGCCGTATTCGTCCGTCCAACTTGAACCGGCGGACAGCAGCGGATAACGGATCATATCGGTCAGCGGTACTTCGCACACCAGCGCACCGATGCTTTGCGGTTCGCGCACGAAGGCGGCGGCGGTAATCAGGCCGCCGTTGCTGCCGCCCTGCAAGCCGATGTGTTTGGGCGAACTCATGCCGCGTTCGGACAAATCACGCACGACTGCCAACAAATCATCAACGCTTTTGTGTTTGCTGATTCCCTGCGCCGCCTGATGCCAGCGCGGGCCGAATTCTCCGCCGCCGCGGATGTTTGCCAATACAAAGGCATTGCCCTCTTCCAGCCAATATTTGCCGACGCTGCCCAGATAATGCGGCAATTCAGGAATGCCGAAACCTCCGTAAGCATAGACTAAGGTCGGCGTGTCGGGCGCGGCGTTTTTGCCGACGTGGAAATAAGGAATGCGTTCGCCGTCGGACGACACCGCCCAAAACTGCCGCACTTCGATGCCGTCTGAAACAAACTGCTGCGGCTGGAGGCGCATGACGGTCAGTTCCATCACGTTCAAATCCAGCGCAAACAGCGTCAGCGGCGTGGTGAAATCGCTGGCGGCAAGATAAACCACGTCGCCGCCCCACGGTTGGTCGGTCATTTCCAACGCGCCCGAGGGCAGGTGCGGCAACTCGGCTTCCTGCCATTTGCTGTCGGCAAACCGCCACGCTTTCAGACGGCCTTGTACATTCTCCAGCAGGCTTGCCACCACAAAACGCTTGGTCGTTTCCACGCTTTCCAATGCCTGCGTTTCATCGGGCGCAAACAAAAGCTGCGCCGCCCCGAGTTCGCCCCGATTCAGTTTCACCGCCACCAACGCGCCACTCGGATAGCTTTGGTTCGCGCGGTGCCAGTCCTTGCGCAGCGTCAGCAAAAGATGTCCCGCCAGATAGCCGACCACATCGCAATCATTAGGCAGGTTCAACGGTTTCGCCCCGCCTTCGGACGACACCTGCAAATACGTCTTGGTGTAAAAACCGTCCGACGCTTCAATCAAATCAATCGGCGAACCCTGCGGATCGAGGTAACGCCACGCGTTTACCATCATCGCGCCTTTATCGATTTGGTACGCCGGCAGGCTTTCCTCGAAACTCTTGCCGCGTTCCACCAGCCACACTTCGCGCGGATAGCCCGATTCGGTCAACTGGCGTTCGTCCCAAGCCGGACACACCCACACGCTGTTTTCATCGCGCCACGACACATGGTTTTTGCCTGCCGGAAAGTGAAAACCGCCCTCTACCAATTCCCCTGCTTCCAAATCCACTTCCAGCGTATACGCCGTATCGCCGCCCGATTTGTTCAAAGTCAGCAGCGCGCGGTTGGGCTGCTCCACCAAGTGCGACACGCCGCCCAAATACACATCGTCGCCGAGCAACTCATCGAAATCCGCCACCGAAAACAGGATTTTCCACTCGGGATAGCCGGAACGGTAGGTCGCCGCCGTACACATGCGGTACACGCCCTTCGGATATTCCGCATTCTGATGGAAATGGTACATCCGCGCGCGGTGTTCCTGACAAAACGGAATCTGCCGCGTGTCCTGCATTTGATTCAAAATGCCGTCTGAAAGTGCGCGCGCCTTGTCGTTTTCTAAAAAACGCGCGCGCGTTTCGGCATTCGCTTCAGCAGCGAAGTTTTGCGTTTCGGCGGAATCGAGGTTTTCAAAATGGCGGTAGGGGTCGGGGTAGGATTTCATCGGGGTCCTTGAGGGTCGGGCAGGTCTTTGTCATCGGGGAAATGCCGTCTGAAACGGGGTTCAGGCGGCATTTCTGCGGCGGCTTTCCGTTGCGGTCAGCGGTGCAGGCGACGCACCAGAATATGCGGGTTTCGTCCGTTGCTGCGGTAGTCTTTGCGCCGCGTTTCGGGCAGCTCGTCTTCCGATGCCGTCTGAAAGCCGCGTTCGGCAAACCATTCGCCGGTATTTGTGGACAGTGCGAACAGCCTGCTTATGCCTATGCCGCGCGCCTTATCGATAATGTGGGCAAGCAGGCGTTCGCCGTAGCCGCCGTCCTGTGCCTGCGGCGAGACGGCAAGGCAGGCGATTTCGCCGCAATCGGCTTCGGCAAAGGTTTTGAGTGCGGCACAGCCGTACAGGTTGCCGTCGTGTTCGAGGATGGAAAATTCGGAAATGTGGTTTTCGAGGTATTCGCGGCTGCGGTGCAATAGGATGCCCTGTTCTTCCAGCGGGCGGATGAGGGCGGCGATGTGCGGGATGTCGCCGCTGTGCGCCTGCCGGATGGAGACGAAGGCTTCTTTGGCAATGGACGTGCCGATGCCGTTGCGGGTGAAGAGTTCTTGCAGCAGGCTGCCGTCGGCGGCCCCGTTGAGGATTTGGACGCGATGCACGCCGCCTTCGAGCGCGGCAACGGCGGACGAAATCAGTCGTCGGGTTTCGCTGGCGGCGTGTTCCGCCAGCGATTGCGCTTCCTGTGCCGAGAGGGTTTCGGCGAGCGTGCCGTCGGGGCGGGAAATGCCGTCTGAAAGGGTCAGGTAAACGAGTTTTTCGGCTTGAAGCGAGACGGCGACGGAAGCGGCGGCCTGCACCATATCGAGATTGAAGGTTTTGCCGCCGTAGGAATGCCCGAGCGGCGGCATCCAGACGATATTGCCCGCGTCGAGTTGGAAACGGAGGGCGGCGGTGTCGGTTTTGCGGATAACGCCCGCGTATTCCATATCGGTTCCGTCAATCACGCCTATCGGACGGGCGGTCAGGAAGTTGCCCGATACGAGCGGGACGGAAGGCGCGCGCGCGAATCCTGAAACGCTGCCGCACAATGCGGCTTCAAAACGGCTGCGGACGGTGCCGGCAAACTGCTGCGCCTGTCCGAGCGAGGTTTCGTCGGTAACGCGCAAACCCCGGCAATAATGCGGCGTGCGGCCTTGCGCGGCGGCGAGGCGGTCGAGGAAGTGGTACGCGCCGTGGATGAGGACGAGTCGGATGCCCAGTTGCGACAACAGCCCGATGTCGGCGGCGAGCTTATTTAAGGTGCCGCCTTCGAGCAGGCGGCCGTCTATGCCGGCGACCAGTGTCGTGCCGCGCATTTGGCGGATGTAGGGGGCGGCTTCGCGGAAGTGGGCGACAAAGCTGTCGGGCGCGTTCATAGGATGAAGAGGTAGGAAAGCTGCATAATGAGGACGATAAGTGCGAACAGGGTTGCAATAGTCCAGTTGAGCCCTTCCTGACGGGCGGGCGGTACGGAAACCTGCGGTGCGGCGGCAGGTGCGGCAGCGGCGGTTGCGGGCGGCGTATCGTGCAGGGTTGTACCGCCGTTGAGGATATCGGCGATTTCGTCGCGGGAAATCTGTTTCTTGCTAATGGCGTGCGTGCCGATGCGGTGGACGAGTTTGACATCCGAAACAGCTTCGGGCAAATCGTTGAATATAGGTTCTTTCGTGCTTGCCAGATGGTCTTTGGCTTTAAACAGCCCTTCGCATTTTTGGCAGACGACGAAGCCTTGGGCGACGTTGAGCTGGGTTTCTTTGACCCAGAGGCGGGTTTTGCAGTGCGGACAGAAACAGGCGGGCATGATGTTTTCTCGGTGTGTGTCGGTTTGATGCCGTCTGAAGCGGCAGGTGGTTCGGACGGCATATGCTTTATTCTACGCCGTACTGCCGGCGGTAGGTGCGGACGGGTTCGAGGAACTGTCCGAATTCGGGGTTGTTTTGCAACAGGATAAACAAATCGTTCAGGCTGGCGATGGGGGCGACGGGCAGGCCGTATTGTTTTTCCACTTCCTGAACGGCGGACAATTTACCCGTGCCTTTTTCCATGCGGTCGAGCGCGATGGCGACACCGGCGGGGGTTGCACCCTCCGCTTCAATCAGTTTGATTGATTCGCGTACGGATGTGCCGGCGGAAATCACGTCGTCGATAATCAGCACGCGCCCTTTAAGCGGCGCGCCGACCAACACGCCGCCTTCGCCGCGGTCTTTGGCTTCTTTGCGGTTGTAGGCAAACGGGACGTTCACGCCTTTTTCCGCCAGCATCATCGCGGTTGCCGCCGCCAAAATAATGCCTTTGTAGGCGGGGCCGAACAGCATATCGAATCGGATGCCGCTTTCAATGATGGATTGTGCATAGAATTTTGCCAGTTGCAGCGTGGACGCGCCGTCGTTGAAGAGGCCGGCATTGAAGAAATAGGGCGACCGCCGTCCGGCTTTGGTGGTAAATTCGCCGAATTTCAAAACATTTTGGGCGAGGGAGAATTTGAGGAAATCTTGGCGGAAATCAGTCATTTTGTGCTTTCTGTCAGATATTGGGACGCAGTTGCGATTCTACCGCCCCGTGCGGCGCGCTTCAACTGCCGCCGCCTTGCCGCAAGGCGCGTTTCAACTGTTTCAACACCTGCCAGTTTCGGGCTTTGAGTTCGGGTTGGCGCAACAGCCGGGCGGGATGGTCGATGATGAAGAAGGGACGGCTGCCGCACAAAGTTTCAATCATCGCCTGCCGTTCCAGGTTGACAAACGCCTGTCCGAGGAATAGGACGGCGGGGGCGCGGCAGCCGTCGAGTTCCCCGGCGATTTGACCCAGCGCATCCGCGACGGCCTGTTCAGACGGCATCGGGTTACCGACGGCGGCGGTTTTCACCCAACAGGTTTTGTGGACATAGGCGGCATCCAGTCCTGCGGCTTTGAGGATGTTGTCGAGCAGGATGCCCGCTTTGCCGTGGAACAGTTGCCCGTAAACCGCATCCTCGATCGGCGGACACAGGCTGACGACGGCAAGCTTGGTAATGCCCGAAGCGGCGGGAACGGGGGCGATGCCGTCTGAAAGGCCGGGCGGGGGCGTTTCGGTTTCAGGCGCGGGTTTGCGCGTATGTACGGCGGCGGTTTCCAACGCTTTCATCGTTTCGAGCCGCGCCTGAACGTTATGGGGTTGGGAAGGGCGGATCGTGGCGGCGCGGACGGTTTGGGGACGTGCCTGTGCCGGGGTTGCGGGTGTGTTTTTGGGCGGCAGGACGGCGGCGGCCTGTTTCAGCCACATCGGACCCAAGCCCAAAGCCTCGTGCAGGTGGAGGTAGCGCGCGCTTAACATATTTTCTCCATTAAGACGGCATCTTCGGTTTGCCCGTCGGCGGCGCAGTAATAGTTTTTCCGCCTGCCCGCAATGCTGAAGCCGTGTTTGGTATACAGTGCCTGTGCGGCGGCGTTGCCTGCGCGGACTTCGAGCAGCAGGCGTTGCGTGCCTTCGGGCAGATGTGCGTACCAATATTCGAGCAGGGCGGACGCAACGCCCCGTCGGCGGCATTCGGGCGCGGTGGCAATCAGGTGCAGTTCGGATTCGTCGGGCAGGTTCTGCCAAACGATAAAGGCGGCAATCCTGCCGTCTTTTTCCGTAAGGAAAACCTGTTCGGACGGCGAAACAAGCGCGGACTCAAATTGGCGTTGCGTCCACGCGGACGGGTTGCAGACGGTATCGAGCGCGGCCAGTGCGGCGCAGTCGGACGGTAAGGCTGGGCGGATGTTCATGTTCGTGCCTTCCGTTCCGCCTGTTCTTTGGCAGTCAGGGCGATTTTGTTGCGGACGTAGAGCAGTTCGGCGTGTGCCGCGCCAGTTGCGGGATAGCCGCCGCCGAGGGCGAGCGCGAGAAAATCGGCGGCGGTCGGCATATCGGGTTTGCCTGAGAAGGGCGGACGGTTTTCCAGTGCGAACGCACTGCCGATGCCGTCTGAAAAGACGTACCCCTCGGGGAGGGCAATGTCTGCCGCCCTACCGACTTGATAATCGCTCAAACGGCGGCGGTTCAGCGTGTCGAACCACGCATAAAACACTTCGCCCATACGCGCGTCCGCAGCGGCGAGTATGCAGCTTTGCGGCGGCGGCAGCGAGGCGGCGGCATCGAGCGTGGGGATGCCGATTAAAGGCGTGTCGAACGGCGTTGCCAAACCTTGCGCCACGCCGATGCCGATACGCAGTCCGGTAAACGCGCCGGGGCCTTTCGCATAAACAATCGCCCCCAAATCGGCGGCGGTAATGCCCGCATTTCGGAATAGGGTGCGGATTTCCGGCAGGATCAGTTCGGACTGGCGGATGCCGACTTCCTGATGGAACAGACGGATTTCGCCGTCGGCGCGCAGCGCGAGCGACAAATAGGAAGTTCCGGTATCGACGGCGAGGACGGGGCGGTTGAAATCGGCTTGCATGGTGTGGTTCTCGTTGGTTCAGACGGCATTATATAGTGAAATCGGCTTGCCTGCCGTGCCGTCGTGTCCTAGGGCGGTATGGCGCAAAAATGCCGTCCGAACGGTAAATTATCGTGTTCGGACGGCATTTTTCAAATACTACTGTTTGTCGGCGATGCCGATTTCGTGAACCTTTTCCCCTATCTTCACGGTTGCCGAGCCAGCGATTTCTTGGGCGCGGTCGCCGAAAAGGGCGAGGCGGTAAGTGCCTTTCTCTTCGCCGCCGTAGCGCGTGTCGCCCAAAATGACGGCGTGTGATTTTTCATCTGCTTTGAGTTCGGCGGAGGCAAGCTCAACATTCTGCTCGGGTGTTTTCAGGTGTTCGATTTTGCCGTGTCCCTGTTTGGCGGCGAAATCTATGGTATAGGTCAGTTTTCCGCCGGCATCGTCGGAGCTGAATGCTTTGCCGTGATACTCGGCTTTGCCGTCAGGCAGTTGGTTGAAGGCGGTATGTTCTCCGCCCAAATCGCTGACAAGGAAGGAGCGTTGGTTTATCAGGCTGTCGATTTTGTCGGGGTTGTTGATTTTTTCAATCCGTAGGGCAACGACGGCGGAGTGATCCTGTTTGTATATTTGAAATTCGCCGCTTGCCAGTGTGATGGTTTGTCCGTCCACTTCGATTTTTTGCACGAAGTCGAAGCGGCTGATTTTGTCGTTCTTCAGTTTGCCCGTGTTGAGGCTGTTGTCTTTGCCGCCGGCTTTGAAAGTTTTTTCCGCACCTTGTGCCGACAGGGTCAGTGTTCCGTTTTGGGGAATGGAGGCTTCCAATGTTAGGGATTTCAAACCTTTGTCTTTATGGTCGAGCGGCGCGGTTAATGCATCGGCAAGCCCCGTGCCGATGTCGGCGGCGACACCACCGCCTCCGCCCCTCCGCTGCTGCAGGCGGTCAGAATCAGGGCCGGCGGTCAAAGAAAGGCAGCAGAAGGTAGTTCGGTTCACAGGTTTACTCCTAGTCATACACAGAATAGATAATATATAAACGTTTTGGTTATGGTATCTTTTTTTGCATACTGCATCAATGAGGCAGGTCAGAGAAGCAAAAATCAAATGCCGTCCGAACGGCGTTCAGACGGCATTTTGTTTACAGGCAACCTGTTATTTGACGATTTGGTTCAATTCGCCCTTGGCATAACGGCTTGCCATTTTTTCCAACGAAACAGGTTTGATTTTGCCTGCCTGACCTTCGCAACCGAACGCAAGATAACGGTCGAGGCAGATTTGCTTCATCGCTTCAATGGTTTTGCCCAAGTATTTGCGCGGATCAAAGTCGGACGGGTTTTCGGCAAGGTAGCGGCGTACCGCGCCGGTGGAAGCGAGGCGCAGGTCGGTATCGATGTTGACTTTGCGCACGCCGTGTTTGATGCCTTCGACGATTTCTTCAACCGGCACGCCGTAGGTTTCGCCGATATTGCCGCCGTATTCGTTGATGACTTTCAGCCATTCTTGCGGAACGGAGCTGGAGCCGTGCATCACGATGTGTGTATTGGGCAGGGCTTGGTGGATTTCCTTGATGCGGTCGATACGCAATACGTCGCCTGTGGGCGGACGGGTGAATTTGTATGCGCCGTGGCTGGTGCCGACGGCAATCGCCAATGCGTCAACGCCGGTATCTTTAACGAAACGCACGGCATCTTCAACGCTGGTGAGCATTTGGTCGTGTGAGAGTTTGCCTGCCGCGCCCACTCCGTCTTCTTCGCCTGCTTCGCCGGTTTCGAGGTTGCCCAATACGCCGATTTCGCCTTCGACGGACACGCCGCAGGCGTGGGAGAAGTTGACGACGGTACGGGTGGCGTTGACGTTGTATTCGTAAGAAGAAGGGGTTTTGCCGTCTTCGAGCAAAGAGCCGTCCATCATCACGGAGGAGAAGCCCAGTTGGATGGAGCGTTGGCACACGTCGGGCGATGCGCCGTGGTCTTGGTGCATCACGACGGGGATGTGCGGAAATTCTTCGACTGCCGCCAGAATCAGGTGGCGCAAAAACGGCGCGCCCGCGTATTTGCGCGCACCTGCGCTCGCCTGTACGATGACGGGCGCGTTGACTTGGTCGGCGGCTTCCATAATGGCGCGCATTTGTTCGAGGTTGTTGACGTTGAACGCGGGCAGGCCGTAGCTGTTTTCGGCGGCGTGGTCGAGCAGTTGGCGCATGGATACGAGTGCCATTTGTGTCTCCTTGGGCAATAGGTAAATAAGGCGGATTATAATGTTTTTTACGGCAAAAAACCATAAACGGCTCATTGATTTTATATTAACGATAACGGCGGGCAGATGGGGCGGATTTTGGGCGGTTCGGGATTTTGGTGTGTTGTTTATGATAATGTTTTCACTGGTTTTTTGATATTTGTGTGGGACGGTTATGGTTTTGGACGGGTTTGCGGCGTATTTTGACGCTTATTTGGAAAACATCGTGCGCGAGGGCAAGTCGGAGCACACGGTTGCGGCATACCGGCGCGATTTGGAAGAACTGTTTGCACTGTTGGCACAAATGCCGTCTGAAGATGCAGGCGGCGTGCCGCAGGACTTGTCGCGGCGCGATTTTACGGCGGCGTTGCGGCGGCTGTCGCAGCGCGGTTTGGACGGTCGGACGCTGGCGCGCAAGCTGTCGGCGTGGCGGCAGTATTGCGCCTGGCTGGTCAAACGCGGGCTGATGCGCGCCGACCCGACCGCCGACATCAAACCGCCGAAGCAGCCCGAGCGCGTACCCAAAGCCCTGCCGCAGGAATGGCTGAACCGGATGTTGGATTTGCCCGTGGACGGCGGCGACCCGCTGGCGGTGCGCGACCACGCGCTGTTCGAGCTGATGTACGGCAGCGGTTTGCGCGTGAGCGAGATACACGGCTTGAATGCAGATGATGTATATTTGGACGAAGCGTGGGTACACGTTACCGGCAAAGGGCGCAAGCAGCGTCAGGTTCCGCTGACCGGCAAAAGCGTGGAAGCCTTGAAAAACTATCTGCCGCTGCGTCAGACGGCATCGGACGGCAAAGCCCTGTTTACCGGCAGGAACGGCACGCGCCTGAGCCAACGCCAAATCCAAAAACGCCTCGAATCGTGGGCGGCGCAATACGGCGACGGCAGGCACGTTTCGCCGCATATGATGCGCCACAGCTACGCCGGCCACCTGTTGCAGGCTTCGCGCGACATCAGGGCGGTGCAGGAGCTGCTCGGACACAGCAGCCTTTCGACCACGCAGATTTATACCAAGCTCGATTTCGACCACATCGCCCGCCTCTATGACGAAGCCCACCCGCGCGCCAAGCGGCAGGACGAATGACGTACGGCAAAATCAGCCGTCAGCCGCACGCTCTTGATATATAATTGACCGTTGCACCCGGACGACACATAAAAAAGACACACCATGAACCCAAGCCCCCTACTCGACCTGATTGACAGCCCGCAAGATTTGCGCCGCCTGGACAAAAAACAGCTGCCGCGCCTTGCCGGCGAGTTGCGCGCCTTTCTGCTGGAATCTGTCGGGCAGACCGGCGGGCATTTCGCCAGCAATCTGGGTGCGGTCGAACTGACCATCGCCCTGCACTATGTGTACGACACGCCCGAAGACAAGCTGGTGTGGGATGTCGGACACCAAAGCTACCCGCACAAAATCCTGACAGGCAGGAAAAACCAGATGCACACCATGCGCCAATACGGCGGTTTGGCGGGTTTTCCGAAACGTTGCGAGTCCGAGTACGACGCGTTCGGCGTGGGGCATTCCTCCACCTCCATCGGCGCGGCTTTGGGCATGGCGGCGACGGACAAACTCTTGGGCGGCGACCGCCGCAGCGTCGCCATCATCGGAGACGGCGCGATGACGGCGGGGCAGGCGTTTGAAGCCTTGAATTGCGCGGGCGATATGGATGTGGATTTGCTGGTCGTCCTCAACGACAACGAAATGTCGATTTCCCCCAACGTCGGCGCGTTGCCCAAATATCTTGCCAGCAACGTCGTGCGCGATATGCACGGACTGTTGAGTACCGTCAAAGCGCAAACGGGCAAGGTATTAGACAAAATACCCGGCGCGATGGAGTTTGCCCAAAAAGTCGAACACAAAATCAAAACCCTTGCCGAAGAAGCCGAACACGCCAAACAGTCGCTGTCGCTGTTTGAAAATTTCGGCTTCCGCTACACCGGCCCCGTGGACGGACACAACGTCGAGAATCTGGTGGACGTATTGAAAGACTTGCGCAGCCGCAAAGGCCCTCAGTTGCTGCACGTCATCACCAAAAAGGGCAACGGCTACAAACTCGCCGAAAACGACCCCGTCAAATACCACGCCGTCGCCAACCTGCCTAAAGAAGGCGGGGCGCAAATGCCGTCTGAAAAAGAACCCAAGCCCGCCGCCAAACCGACCTATACCCAAGTATTCGGCAAATGGCTGTGCGACCGGGCGGCGGCAGATTCCCGACTGGTTGCGATTACCCCCGCCATGCGCGAGGGCAGCGGACTGGTGGAGTTTGAACAACGATTCCCCGACCGCTATTTCGATGTCGGCATCGCCGAGCAGCACGCCGTTACCTTTGCCGGCGGTTTGGCGTGCGAAGGCATGAAGCCCGTCGTGGCGATTTATTCCACCTTTTTACAACGCGCCTACGACCAACTGGTGCACGACATCGCCCTGCAAAACCTGCCCGTTTTGTTTGCCGTCGACCGTGCGGGCATCGTCGGCGCGGACGGTCCGACCCATGCCGGCTTGTACGATTTGAGCTTCTTGCGCTGTGTGCCGAACATGATTGTTGCCGCGCCGAGCGATGAAAACGAATGCCGCCTGCTGCTTTCGACCTGCTATCAGGCGGATGCGCCCGCCGCCGTCCGCTATCCGCGCGGCACGGGTACGGGCGCGCCGGTTTCAGACGGCATGGAAACCGTGGAAATCGGCAAGGGCATTATCCGCCGCGAAGGTGAGAAAACCGCCTTCATTGCCTTCGGCAGTATGGTCGCCCCCGCATTGGCGGTTGCCGAAAAACTGAACGCCACCGTCGCCGATATGCGCTTCGTCAAACCGATAGACGAAGAGTTGATTGTCCGCCTTGCCCGAAGCCACGACCGCATCGTTACCCTTGAAGAAAACGCCGAACAGGGCGGCGCAGGCGGCGCGGTCTTGGAAGTGTTGGCGAAACACGGCATCTGCAAACCCGTTTTGCTTTTGGGCGTTGCCGATACCGTAACCGAACACGGCGATCCGAAAAAACTTTTGGACGATTTGGGTTTGAGTGCCGAAGCGGTGGAACGCCGGGTGCGCGAGTGGCTGCCGGACCGTGATGCGGCAAATTAAACCGCTTGACCGCGCCGTCGTTATCGGGCGGCGTTTTTAAACGGCGTTTGTTTCTGCGGTTTTTTTATTGAAACCCCGCAGGCGGCAGGAAGGGTTCGGGCGGCGGCTTTCGGGCGGTGCTTGGTGTGCCGTTGCGCGTTTGGAAATTTATTCCGCTTGTCCGTATAACGGCGGCGGTGCCGTCTGCCGATACAAGGCAAAATGCCGTCTGAAACGCTTCAGGCGGCATTTTTCGGCGTGAGGGTTTTAGGCTTCGACAATTTTGCCGCGCAGGGAAAAGGTGTAGGCTTCGGTGATTTCCAAATCGATCATTTGGTTGATCATGTCGGGCGTGCCGGTAAAGTTGACGACGCGGTTGTTGGCGGTACGGGCTTGGAGCTGGTCGGGGTCTTTTTTGGAGATGCCTTCGACCAGGCAGCGTTGAACCGTGCCGATCATGGTTTGGTTGATGCGGGCGGTTTCGGCTTCGATGACTTCGTTCAAGGCTTCGAGGCGGCGCACTTTTTCTTCGTGCGGCGTGTCGTCCGGCAGGTTGGCGGCAGGCGTGCCGGGGCGCGGGCTGTAAATAAACACGAAGCTCAAGTCGAAGGCAATGTCTTTCACCAGTTTCAAGGTTTGCTCGAACTCGCGTTCGGTCTCGCCGGGGAAACCGACGATGAAGTCGCTGCTCAGGCACAAATCAGGACGGATGGCGCGCAGTTTGCGGATGATGGATTTGTATTCCAAAGCGGTGTAGCCGCGTTTCATCGCGCTCAATACGCGGTCGGAACCGCTTTGAATCGGCAGGTGCAGGTGGGAAACCAGTTTGGGCAGGTCGCGGTAGCACTCGATAATCGAGTCGGTAAACTCGCGCGGGTGGCTGGTGGTGAAGCGCATACGTTCGATGCCGGGGATTTCGTGGACGATACGCAGCAGGGTGGCGAAGTCGCAGATTTCGCCGTCGTCCATTTCGCCGCGATAGGCATTGACGTTTTGTCCCAAGAGGTTGATTTCTTTCACGCCTTGCTGGGCAAGGTTGGCGATTTCGGTCAATACGTCGTTGAGCGGGCGGGAGAATTCTTCGCCGCGCGTGTAGGGGACGACGCAGAAGGAGCAGTATTTGGAACAGCCTTCCATAATCGACACAAATGCCGCGCCGCCTTCGACGCGGGCGGGCGGCAGGTGGTCGAATTTTTCGATTTCGGGGAAGGAAATATCGACTTGCGACAGCCCGCTGGTTTCTTTGTCCACAATCATTTTGGGCAGGCGGTGCAGCGTTTGCGGGCCGAAAACCACGTCAACATAAGGCGCGCGTTTGATGATGTTTTCGCCTTCTTGCGAGGCGACGCAGCCGGCAACGCCGATGATGAGGCCGGGGTTTTTTTCTTTGAGCGGACGGACGCGCCCCAAGTCGGAGAACACTTTTTCCTGTGCTTTTTCGCGCACGGAACAGGTGTTGAACAAGATGATGTCGGCTTCGTCGGCTTGGGTAACCTGTTCGATGCCGCCGTGTTCTTCGGCAAGGACGGACAGCATTTTTTCGCTGTCGTACTCGTTCATCTGGCAGCCGAAGGTGCGGATAAATACTTTTTTCATGGTTTGTGTCTTTCTCGGGCAGCCGTAATCGCGGGGCTGATGGTTGTTGGAATGAAAAAATTTCAGACGGCACGACGATGCCGTCTGAAAATCGGTGCGGATTATAGCACGATGTGGGTTTGGGAGGCAAAATATTGTTTTAAAATATGAATTTAATCGGTCGGAACGGCTGTATAATGTTTGGCTTTAACGGGAGGTGTGTGTATGGGCGGCATTGCTGCTGTGTGCGTGTACCAGCAATTTCGGCGACAGGGAACATCAGTTCCTGCGTTATAGTGGATTAAATTTAAACCAGTACAGCGTTGCCTCGCCTTGCCGTACTATTTGTCTGTCTGCGGCTTCGTCGCCTTGTCCTGATTTTTGTTAATCCACTATATCAGACGGAAGAGGGAATCGCACAAACGGTTGTTAAAGGCAAAACGACCCGGGCGGAGGTAGAGGCACGTTTCGGGAAGCGCAACCCTTTCGGCTGTTATGCCTATCACGAGGTCAGCCTGCCGATTTATAATTTTTTGCCGACCAATTTCATCTATATGAAATCGGAGCGGCGGCATTGGGAATGGTGCGTGGATTACGACGGGGAGGGAGTCGTCCGGGACTACCGCTTTACACATATAAAGAGGAAAAAGACGAGCGTTCCGTCATCCGGGATACGGTTGGCGTAATCCGCAAAGAAGCGGGCAAATCCTTGTCGCAACCTGAAAAATGATAAAATGGGGCTTTCTGCTTCCAAGCCCGAAACCTGCCGTTCAGACGGCATTTGAGGATAAATATGAACCGTAACGAAATTTTATTCGACCGCGCCAAAGCCATCATCCCCGGCGGCGTGAATTCGCCCGTGCGCGCATTCGGCAGCGTCGGCGGCGTGCCGCGCTTCATCAAAAAAGCCGAAGGCGCGTATGTTTGGGACGAAAACGGCACGCGCTACACCGATTATGTCGGCTCTTGGGGGCCTGCGATTGTCGGACACGCGCATCCCGAAGTCGTCGAAGCCGTGCGCGAAGCTGCGTTGGGCGGTTTGTCGTTCGGCGCGCCCACCGAAGGCGAAATCGCCATTGCCGAACAAATTGCCGAAATTATGCCGTCTGTCGAACGGCTGCGCCTCGTCAGCTCCGGCACGGAAGCGACGATGACTGCCATCCGTCTGGCACGCGGTTTTACCGGCCGCGACAAAATCATCAAATTTGAAGGCTGCTACCACGGCCATTCCGACAGCCTGTTGGTGAAAGCAGGCAGCGGTCTGCTTACCTTCGGCAATCCTTCTTCCGCCGGTGTGCCTGCCGACTTTACCAAACATACTTTGGTACTCGAATACAACAACATCGCCCAACTCGAAGAAGCCTTTGCCCAAAGCGGCGACGAAATCGCCTGCGTGATTGTCGAACCCTTCGTCGGCAATATGAACCTCGTCCGCCCGACCGAAGCCTTTGTCAAAGCCTTGCGCGGATTGACCGAAAAACACGGCGCGGTGTTGATTTACGACGAAGTGATGACCGGTTTCCGCGTCGCGCTCGGCGGCGCGCAGTCGCTGCACGGCATCACGCCCGACCTGACCACGATGGGCAAAGTCATCGGCGGCGGTATGCCGCTTGCCGCGTTCGGCGGACGCAAAGACATCATGGAATGTATTTCCCCGTTGGGCGGCGTGTATCAGGCAGGTACATTATCAGGCAACCCGATTGCCGTCGCCGCCGGCTTGAAAACGCTGGAAATCATCCAGCGCGAAGGCTTCTATGAAAACCTGACCGCCTTGACACAACGCCTTGCCAACGGTATTGCCGCCGCCAAAGCGCACGGTATCGAGTTTGCCGCCGACAGCGTGGGCGGTATGTTCGGTCTGTATTTCGCCGCACACGTGCCGCGAAACTATGCCGATATGGCGCGCTCCAATATCGACGCTTTCAAACGCTTCTTCCACGGCATGCTCGACCGCGGCATTGCCTTCGGCCCGTCCGCTTATGAAGCAGGTTTCGTTTCCGCCGCGCATACGCCCGAGCTGATTGACGAAACGGTTGCGGTTGCGGTTGAAGTGTTCAAGGCGATGGCTGCATGATGTTTTGACGGACAGAGTTTCTCTGTTCGATTTGTTTGGCAGATTGAAGTAAGAATGCACACCGCCGTCATTCGTCATTTCCGCGCAGGCGGGAATCCGGACCTTTCAGTTTCTGTAATGATTGAAAATAACGGCAAGCCCGACCTTCCGGATTCCCGCCTGCGCGGGAATGACGGGCGTGTACATTTTTGATTTCAATTTACTGTAAAAATGCCGTCTGAAATATATAGTCAATTAAAATCAAAATAGGACAGTAGCGCATCGTCAAATCGGGCGTAATCAGACAAAACGGTTCG

>2 |ref|NC_017511.1| Neisseria gonorrhoeae TCDC-NG08107 | Coordinates: 24526,34571 | Forward

GCTTTGTTTCTTAAGTCCGCAGAGTATGCCATGGTTAAACCTTCAACGTCGAGTGTTGTACTATTTTGTTTTTAATTGAATATAAATAGCGTTTCAGACGGCATTGTTTGTTTCTTATTGCGCTTCGGCTTTCGGGGCGGAAATCAGGAAGTGTTCGCGGTAGTGGCGCATTTCTTCGATGCTCTCCAAAATGTCGTCCAATGCCTTGTGCGAACCGCGTTTGACGACGCTTTTGGCAATGGGCGGATTCCAGCGTTTGGCGAGTTCTTTCAGCGTGGAAACGTCGAGGTTGCGGTAGTGGAAGTAGTTTTCCAGTTTCGGCATATATTTGACCATAAAACGCCGGTCTTGGTGGATGGAGTTGCCGCACATCGGTGTGGCGCGTCCGGGTATCCATTCCGACATAAAGTCCAGCAGTTTCTGTTCGACTTCGGCTTCGGTATGCGACGATTCGCGCACGCGCTGCGTCAGCCCCGTCCTGCCGTGTGTGGCGGTGTTCCACTCGTCCATATTGTCGAGCAGATCGTCGCTTTGGTGGATGGCGTAAACTTCGGATTGCGCCAACACATTCAAATCCGAGTCGGTAATAATCATCGCGACTTCGATAATGCGGTCGGTTTCGGGATTCAGCCCCGTCATTTCCATATCGAGCCAGCAGAGGTTGTTTTTGTCCTGCACGGTGTTTCCTTTCCGTTTCAGCGTTTTACGCCGTTCAAGCGCGCCCAGCCTTCGTCAGTTTCCGCCGGGTCGAGATCGAACCATTGGCTGTAAATGCCGCCGAGTTCTTCGGCCTGTTCATCCAACAAACCGGACAACACGATGCGTCCGCCCTGTTTGGTGCGGGCTGCAAGCATTTCGCCGAGCATACGCAAAGGGTTGGCGAGGATGTTGGCGACAACTACGTCGAATTGCCCTTGAGGCAGACTGTCGGGCAGGAAGAATTGTGCATCGACGTTGTTCTGTTCGGCGTTGTCCCTGCCGGAGCGGACGGCCTGTTCGTCAATATCCACGCCGACGGCGGAACCTGCACCGAGTTTGAGGGCGGCGATGGTCAGGATGCCCGAACCGCAGCCGTAGTCGAGGACGCTTTCGCCGTTTTTGAGTTGCGTATCCAGCCATTTGAGGCAGAGGCGCGTGGTCGGGTGGCTGCCGGTGCCGAAGGCGAGTCCGGGATCGAGGCGGAGGTTGACGGCGCAGCCTTCGGGGGCTTCGTGCCAAGAGGGGGTAATCCACAGGCGGTCGGAAATTCGGATGGGGTCGAATTGCGATTGCGTGAGGCGCACCCAGTCTTGGTCTTCGATGGTTTCGCCGGTGTATGCCAAGTCTTTTAACCCGCATTCTTGTGCGGCGGCATCGATGACGGCGGCGGCTTCGTCGTGTTCGCCGAACAGGGCGATGACTTTGCTCTGCTGCCAGATTTGTTCGGTGGGCATACCGGGTTCGCCGAAAATCGCCTGTTCGTTTTGCGTGCCGGCGCAGGCATCTTCGATGGCGGCGGAGAGTGCACCGTGTTCCATCAGCGCGTCGGCGAGGCGTTCGGCGACGGCATCGTTGACGTTGACGGTGATTTGTTGGTAGGGCATAACGGGCTTTCTTGGAAATATCGAAATCGGTTTCAGACGGCGGGGCAAGGGAAATGCCGTCTGAACGCGGAAGGGCGGCTTCAGACGGCATATCGGCGGCGGCTTATTTGTCCTGTTTGGTTTTGCGCGCTTCCAGCCAGTGCTCCAGGTAATGGATGCTCACTCCGCCTTCTTGGAAACCCGCATCGGCGAACAGGTCGCGGTGCAACGGCGTATTGGTTTTGATGCCGGTTACCGCCAGCTCGGCGAGTGCGACGCGCATTTTCGCCATTGCCTGTTCACGCGTTTTTCCGTGTACGCAGATTTTGCCGATCAGGCTGTCGTAGTACGGCGGGATGCGGTAGCCTTGGTAGATGTGGCTGTCCACGCGGATGCCGAAGCCGCCGGGCAGGTGGCAGCTTTCAATCGGGCCCGGGCTGGGGATGAAGTTGTACGGGTCTTCGGCATTGATGCGGCACTCGAAGGCGTGGCCTTCGATTTTGATGTCTTTCTGTTTGTATTGCAGCGGCAGGCCGGATGCGATGCGGAGTTGCTCTTGGACGATGTCCACGCCGGTAATGAGTTCGGTAACCGGATGCTCGACCTGAACGCGCGTGTTCATCTCGATAAAGAAAAATTCGCCGTCTTCGTATAAAAATTCAAACGTGCCCGCGCCCCGGTAGCCGATGCGTTTGCACGCGTCGGTACAGGCTTTGCCGATTTTTTTGCGTGCCTCTTCATCGATAAACGGGGCGGGTGCTTCCTCGATGACTTTCTGGTGGCGGCGTTGCAGCGAACAGTCGCGCTCGGCAAGATAGACGGCGTTGCCGTGTTCGTCGGCAAGCACTTGGATTTCGACGTGGCGCGGGCGTTGCAGGTAGCGTTCCATATAAACCATCGGGTTGCCGAACGCCGCGCCCGCTTCAGCCTTGGTCATTTCGACAGACTGGAGGAGGTCTTCTTTTTTCTCGACCACGCGCATACCGCGCCCGCCGCCGCCGCCCGATGCTTTGATAATCACGGGATAACCGACTTTGTCGGCGATTTTGAGGATTTCGGCATCATCGTCGGGCAATGCGCCGTCAGAGCCGGGGACGCAGGGCACGCCTGCCGCTATCATCGCGTGTTTGGCGGAGACTTTGTCGCCCATCAGGCGGATGGTGTCGGGTTTCGGGCCGATGAAGGTAAAGCCGGACTGTTCGACCTGTTCGGCGAAATCGGCGTTTTCGGCAAGGAAGCCGTAGCCCGGGTGGACAGCGTCCGCGCAGGTTACTTCGGCGGCGGCAATGATGGCGGGAATGTTCAGATAACTTTGCGCAGAAGCGGCAGGACCGATGCACACGGATTCGTCGGCGAGTTTGACGTGCAGGCTGCCTTTGTCGGCTTCGGAATGCACGGCAACGGTGGCAATGCCCATTTCGCGGCAGGCACGGAGTACGCGCAAGGCGATTTCGCCCCGGTTGGCGATTAAAACTTTTTTCAGCATGATGACCTTTCCTGCGGTTCCGGCAACCCGTCCGTAAAAAAAGGGTGCGGGAACGCTGAAGGGGGGAAAGCATTTCAGACGGCATCGGAAGTTTATGCCGTCTGAAAACAGGATTATCCGATGATGAAGAGCGGTTCGCCGAATTCGACGGGCGTACCGTTTTCGACCAGAATTTTTTTGACCGTGCCGGATTTTTCGGCTTCGATTTCGTTCATCAGCTTCATCGCTTCGATGATGCACAGCGTGTCGCCGGCTTTGACCTGTTGACCGACTTCGACAAATGCGGCGGCATTCGGGCCGGGCGCGCGGTAGAACGTGCCGACCATAGGCGATTTTTGAGCGTCGGACAAATCGCGGGCGGCCGGCGCGGCGGCGGGCGCGGATGCCGCAACGGGTGCGGCGGCCGGCGTTACGGCCGGCGCGGCGGCAGGTACGGGCGCGGCGTAAACGGGAGCTGCCGCAGCTGCAATGGTACGGGTAATGCGGACTTTTTCTTCGCCTTCGGTTACTTCGATTTCGGCGATACCCGATTCTTCAACCAAATCAATCAGTTTTTTTAATTTGCGCAAATCCATTTCTGTTCCTTTAAAGGCTGCCGGTGTTCCGGCGGGTTGTTGCGTTGGTTTTTCAGAAAACAGCCGGCTTGCGGCAACGGCGCAAACCGGGCGGAATGGTGTTCGGATACGGTTTTCCTGTGGTGGGAAACGACCGTGAAATTGTGTTCATTTTCCCGAAGTTGTCGGTAAATGTCCAGTAAAATATCAAAAAACGGCGGTTTTTGGGGGAAATGTGCAGGAAGTTTGATTTTGCGCACAAGATGCCCCGAGTCAAGAGTGTTTATTCTAATCTGTTGGTTTTTCGGGCAAAGATGCCGTCTGAAAAAGGGCTAAAGTGCGTATAATGGCGGCTTGCCCGAACGAGAGTGTAAAAATGGATATTTCAGATTTTGACTTTACCCTGCCCGAACACCTGATTGCCCAGCATCCGCCCGAGGTGCGCGGCAGCAGCCGGCTTTTGGTCGCGCTGCCCGATATGCCGCTGCAAGACCGGGTGTTTGGCGATTTGCCCGATTATGTCGAGGCAGGCGACGTTTTGGTATTCAACAACACCAAAGTCATGAAGGCGCGGCTGTTTGGGCAGAAAGACAGCGGCGGCAGGATCGAAGCCCTGATTGAGCGTGTGTTGGACAACCATACCGCATTGGCGCACATCCGTTCGTCCAAGTCCCCCAAGCCCGGTATGGGGCTGGTGTTTGAAGGCGGTATCCGTGCCGTGATGGTCGGGCGTGAGGGCGAACTGTTCTGCCTGCGTTTTGAAGGCGGTCAAACCGTTTACGAACTTTTGGAACAGAACGGACACCTGCCCCTGCCGCCTTATATCGAACGTGCCGCCGATGCGGACGACGACAGCCGTTATCAAACTGTTTATGCCAAATATCAGGGCGCGGTCGCCGCGCCGACGGCGGGCCTGCATTTTACGGAAGAACTTTTGCGCCGTCTGAAAGACAAAGGCGCGGTAACCGCAGAAGTAACCCTGCACGTCGGTGCGGGGACATTCCAACCCGTGCGCGTCGATAAAATCGAAGAACACAAAATGCACAGCGAATGGTTTGAAGTGCCGTCTGAAACCGTCGCCGCCGTTGAGGCGGCAAAAGCCCGGGGGAACAAAGCCTGGGCGGTCGGCACGACTTCCATGCGCGCCCTCGAGTCTGCCGCGCGCGCAACGGGATATTTGAAAGACGGACAGGGCGACACCGATATTTTCATCACGCCGGGCTACCGTTTTAATGTTGTCGACAGGCTGGTTACCAATTTTCATCTGCCGAAATCGACGCTGCTGATGTTGGTCGGCGCGTTTTCGGGTATGGGTCATATCCGCGCCGTGTACCGTCATGCGATTGAACGTGAATACCGTTTCTTCAGCTACGGAGATGCGATGGTTTTGGGGCGGAACGAAGGGGGCGGGCTTTAAACTGCTGCCGTCCGTTGCAAGGCAGATGCCGTCTGAACCGTGGTTCGGGCGGTATTTTTATGGATGTCCGGCAGTTGGATAATCCACCGCCCCAAATTAGGGTGCTTAAAGGTCAAAAGAAAGTGAAGGCTATGTGCAACAAAATGCCGTCTGAAACCGCAAAACGGCTTCAGACGGCATTGTTCAACCTGATTCAGGCATCAGTTGCGGTTTTTCAAACGACCGTGCAGCTCTTGAACGCTGTACACACCCAGATAATCCCGGCTTTTCGCGCCTTCGCTCATCGCTTCGCCGCCGGCGGTGGTGGTGTATTGCGGCACGCGTTGCTGCAATGCGCTTTGGCGGATGATGTGGCTGTCGGACACGGATTGCGGATCGCTGGAAACGGTATTTACGACCAGTGCGATTTCGCCGTTTTTCAGCGCGTCGCCGATGTGCGGGCGGCCTTCGGGGACTTTGTTGATGGCCTGCACAATCAGCCCGTGTTCGGTCAGGTATTGCGCCGTGCCGCGCGTGGCGCAGATGCCGTAGCCTAAGGCTTGGAAGTTTTTGGCGGTTTTAATGACGCGTTCTTTGTCTTCTTCGCGCACGGAGAGGAAGATTTTGCCGGTCGGGTTGAGGCGTTCGCCCGCGCCGAGTTGGGCTTTGTAGTAGGCTTCGCCGAAACTTGCGCCCACGCCCATCACTTCGCCGGTGGAGCGCATTTCCGGACCCAAAATCGTATCCACGCCCGGGAATTTGATGAATGGGAACACGGCTTCTTTAACGGCATAGAAATCGGGGACGACTTCTTTTTCCACGCCTTGTTCTTTCAGGGAAATGCCCGCCATACAGCGTGCGCCGACTTTGGCGAGCGGCACGCCGGTGGCTTTGGAGACAAAGGGGACGGTACGGCTGGCGCGCGGGTTCACTTCCAACACGAACACCACGCCGTCCTGCACGGCAAACTGCACGTTCATCAGTCCGACCACGCCCAGCGCGTACGCCATCGCTTTGGTTTGGCGGCGGATTTCGTCTTGGATTTCTTCGCTTAAGGAGTAGGGCGGCAGCGAGCAGCCGGAGTCGCCGGAGTGGATGCCCGCCTGTTCGACGTGCTGCATGATGCCGCCGATAACCACATCTTTGCCGTCTGAAACGCAGTCCACATCGACTTCAATCGCGTTGTTGAGGAAGAAATCGAGCAGCACGGGGCTGTCTTCGGAAACCTGCACGGCTTCGCGCATGTATTTTTGCAAGGCTTCGGCGGAGTGGACAATCTGCATCGCGCGTCCGCCCAAAACATAAGACGGGCGCACGACCAGCGGATAACCGATTTCTTCGGCTTTGACGAGTGCTTCTTCTTCGTTGTGGGCGATGCGGTTGGGCGGTTGGCGCAGGCCTAAGTCGTTCAACACTTTTTGGAAGCGTTCGCGGTCTTCGGCGGCATCGATGCTGTCGGCGGATGTGCCGATGATGTTCACGCCGTTTTCAACCAATGCGTTGGCGAGTTTCAGCGGGGTTTGACCGCCGTAATGAACAATCACGCCCCACGGGTTTTCGGTGCGGACGATTTCCAACACGTCTTCCAATGTCAGCGGCTCGAAATAGAGGCGGTCGCTGGTGTCGAAGTCGGTGGACACGGTTTCGGGGTTGCAGTTGACCATAATCGTTTCAAAGCCCGATTCGCGCAGGGTGAGTGCGGCGTGAACGCAGCAGTAGTCAAACTCGATGCCCTGACCGATGCGGTTCGGGCCGCCGCCGAGAATCATCACTTTTTTACGGTCGGAAGGACGGGATTCGCATTCTTCTTCGTAAGTGGAGTAAAGATAGGCGGTTTCGGTGGCGAACTCGGCGGCGCAGGTATCGACGCGTTTGTAAACCGGATGCAGCTTCAGCGCGTAGCGGTGTTCGCGAACTTCTTTTTCGCTTACGTTCAACAATTGTGCCAAACGTTTGTCGGAGAAGCCTTTGCGTTTCAGACGGCGTAGGGCGGCGTAATCCAAATCTTGCAACTGGCCGTCTGAAACCGATTTTTCTTCCTTCATCAAGTCTTCGATTTGCGCCAAGAACCAAGGGTCGATGGCGCAGATCTCGTGGATTTCTTCCGGCGTGAAGCCCGCGCGGAACGCGTCTGCCACAAACAGCATACGTTCGGGGCCGGGGTTGGCCAGTTCGCGGCGGATTTCCGCTTTGTCTTCGCTGCGCGGATTGAAACCGCACAAGCCGGTTTCCAAACCGCGCAAGGCTTTTTGGAAGCTTTCCTGAATGGTACGGCCCATCGCCATTACTTCGCCCACAGATTTCATCTGCGTGGTCAGGCGGTCGTCTGCGGCGGGGAATTTTTCAAACGCGAAACGCGGGATTTTGGTTACCACATAGTCGATGGAAGGCTCGAACGACGCGGGCGTGCGGCCGCCGGTGATGTCGTTGCGCAACTCGTCCAGCGTAAAGCCGACCGCCAGCTTCGCCGCCACCTTCGCAATCGGGAAGCCCGTTGCTTTGGAAGCCAGCGCGGACGAACGGCTCACGCGCGGGTTCATCTCGATCACAATCATCTCGCCGTTTTCAGGGTTCACCGCAAACTGCACGTTCGAGCCGCCCGTGTCCACGCCGATTTCGCGCAATACCGCCAACGAAGCGTTGCGCATGATTTGGTATTCCTTGTCCGTCAGCGTTTGCGCCGGCGCAACCGTAATCGAGTCGCCCGTATGAACGCCCATCGGGTCGAAGTTTTCAATCGAACAGATGATGATGCAGTTGTCCGCCTTATCGCGCACCACTTCCATCTCGTACTCTTTCCAGCCGAGCACAGACTGCTCAATCAGCAGCTCATGCGTAGGCGACGCATCGAAACCGCGTTCGCAAATCGCCAAAAACTCATCCTTATTGTAGGCAATGCCGCCGCCCGAACCGCCCATCGTGAAAGACGGACGAATCAGCGTCGGAAAGCCGACCTGTTCTTGCGCCGCCAAGGCTTCGTTCATGGTGTGGCAGACAAAAGATTTCGGGCAAGAGAGGCCGATTTTTTCCATCGCTTCTTTAAAGCGGCCGCGGTCTTCCGCCTTGTCGATCGCGTCTTCCGTTGCGCCGATTAACTCGACATTGTATTTCGCCAGCATGCCGTTACGCGCCAAATCCAGCGCACAGTTCAGCGCGGTCTGACCGCCCATCGTGGGCAGAATCGCATCGGGCCGCTCCTTGGCGATAATCTTCTCCACCGTCTGCCACATAATCGGCTCGATGTAGGTAACATCCGCCATTTCGGGGTCGGTCATAATCGTGGCGGGGTTGGAATTCACCAAAATGACTTTATAGCCTTCTTCACGCAAGGCTTTGCAGGCCTGTGCGCCCGAATAGTCAAATTCGCAGGCCTGACCGATAACGATAGGGCCGGCGCCGATGATAAGGATGGATTTTAGGTCGGTACGTTTGGGCATGGGTGGTTACTCTTGAATTAAAGAAGGTTGTTTTGTTACATAGCTTTTAAGAAAAGTATTTCCCTTTTCTAAAATTGAAGCTAAAACATCATCTTCTATGTTAGATAAAATATTCTCTAAATCTGCGCTTGATTTAATTTTAAAGAATTCTTTTGTTTGAGAATGGTATACAAGAGAAGGTTTATTTAGCTGTAAAAGTTCTGCTATATTATTTAATGAACCAATACTCTTACCATCCCATAAAATAAAGCCATAATCAGCAATTTCTGCCATTTTCTTATCTTTTGCTGTATAAAATACCCTTCCAGTTCCTTTGCTGTCTACTTGGACAAACTGCCAATTTCCCACATTATTTCGGTAAATTTTGCCAGAAAAGTAAATATGCACATTAGCATAATCTTGTTCTTGTAGAAATTCTTGGATAGCTTTATCAGCCCCATTAGCATCTCCAATAACAATATCAAAATTATTGCTAAGAATATTGTTAATTCTTTCTCGTATTTGTGGATTTAGTCGTGAAATGCTGCGTGAACCAGAAAAGAAAATAGTGCGCATTTTTATCCTCTAGTCTTTGTCATTGCTAAAACAATTACAGATCTAGCATTTTTTGAAAACAATTTTTCAGTTGATATTTCCAATGTTGCTCCGCTATCAAATAAATCATCGATTACTAAAATATTCTTATTGGATAGATCTATATTGTCAATTGTGATGGAATTATTCAGTATTTCTAGTTTTTCTGATTTTTCTTCAATATTTTTGAGTGGTGTGTGTGAGCTAGATTTTCTTAAAATAGGAGAATATGGAATATTCAAGCGATCACTTAGTTCTTTGGCAATTAATTGAACTGGTTGATTAATACGCTCTGTTGTAAAAGGGGCGGGAACAATTAAATTTATAGATTCTAAACCACTAAATTTTTGTAAAATATAGTCTACCAGCAAAGAAACATTTTGGGTTTGATTACGATATTTTAATTGATAAACCCATTCTCCAATCACACTTCTCTTGCTATCAAAGTGAGGATGCCCTAATTCATCATAACCTAACAAAATACTTTTTTGCATATGATGGTCTAAAGCAAATCCTTTTGTCCAGTTACCATTTAGTTCTATCGCCATTTCATTGACTCCTTGTCAAAATTAAGTATAACGAGCAATCCTAGCAAGCCGTAGCCCGCATGTAGGGTATGTGCGGTACGCACGCACGCGTTCTTCATTTTCCCTGTAACCCCAACCCACCGCATGCGCGCCTTGCGGCACACTCTCTGCCGATGAGTTCAAAGATTGTCTGAAAAAACGCTCGCGGCGTTTGTTCCATCTGCTTGACAATAAAGGCTACCTGAAAAATTCCCGCTGTTGATATTTCGTGCATTCTTTTTCAGACGACCTGAAATCTCTGCCGAAACCCACTCCCTCCCCCGTGGGGGAGGGCTGGGGAGAGGGCATTCTCCGAATGGTGGCAATCTTTCCTAATACCTTCGCCGCCCAAATACAAGCCTTGCGGCTTGTTGCCCTCTCACAGGGAGAGAGGACGGGTCGGCTGTTGGGGTTGAGGTTTGCTGCAAGGAAAACAGGTTGTGCAGGTTGCTTTTTATTTTTCAGACGACCT

>3 |ref|NC_017511.1| Neisseria gonorrhoeae TCDC-NG08107 | Coordinates: 34572,37106 | Forward

TTTTAAACGCTTAATTCAGTTAATTTTACCTTATTTTTTTCAAAATCTAATCAAAATCAACCGTTTGCTACCGTGCTGGCTGCTTTTCCAATTCCTGCAATACGCGCAGGATTTCCGCCAGTACATCGTTTGTCTGCTGCAAAATTTCGTGATTCCAAAAACGCAGCACGGTAAAGCCCAAGCTGTTGAGATATACCGTCCGCGCGTGGTCGTATACGGCTTGTTCCGCGTGCTGCCCGCCGTCTGCTTCGACAATCAGCTTGGGCGTTACGCACATAAAATCAACAATATAATTCCCCATCGGCTGCTGTCGGCGGAATTTATAGCCGTTCAGACGGCCTGCCCGCAGGTGTTGCCACAATTTTGCTTCCGCCTCGCTCATTTCTTGACGCATGGCTTTGGCGCGTTGGCGTAGGGCGGGGTTTTCGGCGGTCAATAGTTTCTCGGGCGGGTTCATTTTTGTTATCGGGTTGCTGTTTGTGTAAGCCTTACAGCTTGTTGCCCTCTCTCTAGCTCTCTCCCACAGGGAGAGAGGACGGGTCGGCTGTTGGGGTTAAAGGGGCTGCAAGGAAAACAGCTTGTGCAGGCTGTTTTTTATTTTGCATAATCCGTCTGAAAATTAATAACTGCTGATTTCCAGCAGGTTGCCATCGGGATCGCGCAGGTAAACCGATTGGATTTTGCCCATTGCGCCTGTGCGCGCTACGACGCCGCTTAAAGGTTTGATGCCGTGTGCGGATAATTCCTGTAAAACCGTTTCCAGTGGGGCGTATCGGTCAGCAGGCATAAATCCGCTGTGCCGCAGGCGGCGTGTTGCGCGTTAGGCTGAATTTCCGCACCGCGCCCGTGTAGGTTGATTTTCTGACTGCCAAACAACAAAGCTTTACGGTTGTTGCCAAATGAAACTTCTTCCATGCCCAAAACTTGTGTGTAAAACGCGATGGTTCGGTCAATGTCGGCAACAGTCAGTACTAGATGGTCGAGTGCGCTAATTTTCATTTCCAGCTTTCTTTTTCAGACGGCAGCAGCATACTGTTGCCGTCTGAAAATCATTATGCTTGTTTTGCAGCCTTCATATTGCCAATGAATTTGTCGAACAAATAGCCGACATCTTGCGGACCCGGGCTGGCTTCGGGGTGTCCTTGGAAACAGAACACGGGTTTGTCGGTCAGCTCGATGCCTTGCAAGGTATTGTCGAACAAGGATTTGTGGGTAATGCGTGCGTTGGCGGGCAGGGTGTCGGCATCGACGGCGAAACCGTGGTTTTGGCTGGTAATGACGACTTTGCCGCTGTCCAAATCTTGCACAGGATGGTTCGCACCGTGGTGGCTGAAGCGCATTTTCAGGGTTTTCGCGCCGATGGCGAGGCTGATGAGCTGGTGTCCCAAGCAGATGCCGAAAATCGGTTTGCCGCTTTCCATCAGTTTTTGCACGGCTTCGATGGCGTAGGTGCAAGGCTCGGGGTCGCCGGGGCCGTTGGACAGGAACACGCCGTCGGGATTGAGTGCCAACACGTCTTCCGCGCTCGTTTGTGCGGGGACGACGGTCAGGCGGCAGCCGCGCGAGGCGAGCATACGCAGGATGTTGGTTTTCACGCCGAAATCGTAGGCGACGACGTGGTAAGGCTGTTTGTCAGGGGTAACGAAACCTTTGCCCAATTCCCATTCGCCTTCCGTCCATTCGTAAGTTTCCGTGCAGGAAACTTCTTTTGCCAAATCTTTGCCGACCATGCTGCCGAATGCGGCGATGAGTTCTTGCGCTTTTTCAACGGTGGCATCCGCACCTGTCAGAATCGCGCCGCCTTGCGCGCCTTTTTCGCGCAACAGCATGGTCAGGCGGCGGGTGTCGATGTCGGCGATGGCGACGGTTTCGTTGCGTACCAAATAGTCGTGCAGGCTTTCGGAGGCGCGGAAGCTGCTGTGCAAGAGCGGCAGGTCGCGGATAATCAGGCCGGCGGCATAAACGCTGCGGCTTTCTTCATCTTCGGCGTTGGTGCCGGTGTTGCCGATGTGGGGGTAGGTGAGGGTAACGATTTGTTTGCAGTAGGACGGATCGGTCAGGATTTCCTGATAGCCGGTCATCGAAGTATTGAACACGACTTCGCCGGAAGCCGAACCTTCGTAACCGATTGATGTGCCGTGGAATACGCTGCCGTCAGCGAGGACGAGGAGGGCGGGGGTGCTCATGATGGGAATCCTGTTTTTAATAAAATTCTTGACAATGCCGTCTGAAGGGACTTCGGCAGGGGGCATCCGGTCGGAAAAATCCGGTCAAAAAAAAACACGCCGCAGAACAAAACCGCGTAACGTGCTTTTCGGGCAAGTGCCTATACCCTGAAAGCAGCATATTTTAAGACGAAACATACCCGATTTCAAGCGCAAACGCGGCAGGCGGGCTGATATGTTCGCGCGAAAACGGAAATCCGGGGCATAAAACCGCCCGCTCCGCGCCCCCTATGCCCGTTACGCATTTCCCCACGTCCGCCCGGCGAAACTATGGGAATACCCGAACCGTCATTCCCGCCTGTGCGGGAAT

>4 |ref|NC_017511.1| Neisseria gonorrhoeae TCDC-NG08107 | Coordinates: 37107,37670 | Forward

GACGGTTTAGAAGTTGCCCGAAACCTCAAAAAAAACCGAAACCGAACAAGCCGGATTCCCGCCTGAGCGGGAATGACGGGCTAAATAATATCAAACCATAAATCCCGCCAAGAAACATTATTTTCTTCAATCAGTTGCAACTTCCAAGCCCTGTTCCATTTCTTCAACTGTTTTTCCCGAGTAATTGCGCTCTCCATCGTAGGATGCAGTTCATACCAAACCGGCATAGTAACGTTGTACCGTGATGTAAATCCTTCAATCAAATGCTCCCTATGTTGGTAAATACGTTGCACCAAATCAGATGTAACGCCAATGTATAACGTGCCATTACGTTGGCTTGCTAAAATATAAACCGCAGGCTGCATATAATACCCTTTTGAATTATTTAAATTTATATTCCCGCGAACACCATCCGGTGATTACTTTAACCCTTCGTTATCCCCATAGCTTTCCATCATTCCCGCAACTCTTCGTCATTCCCGCGAAAGCGGGAATCCAGAATCTCTAAAGCTTCAGCTAACCTTTGAATATTGCTGTTGTCCCACGTTCTAGATTCCCGCCTGA

>5 |ref|NC_017511.1| Neisseria gonorrhoeae TCDC-NG08107 | Coordinates: 37671,39955 | Forward

AAACCAAAAACAGAAACCTAAAATTCCGTCATTCCCACGAAAGTGGGAATCCGGTTTTTTGAGTTTCAGTCATTCCCGATAAATTGCCTTAGTATTGAATGTCTAGATTCCCGCCTGCGCGGGAATGACGCGTTTCATTTGCCGCCCCCTCCCGAAAAACGCAAAAAAATGCCGTCCGAAGACCTTTCGGACGGCATTTGCGGAAAAACCGGCCGCGCAGGCGGGTCAGAAGAAGACTTCGCGCCAGCTTAAGCGTTTGATACCGCACATCGGGCCGGTAATATCCAAGCTGTCCAAATCGTTCATCAGCAGGGTGCGCACACCTTTTCCGGAGAAGCACCGGTTATTGCGGGCGGGTTTTTTACCCTCTTTGAATGTTCCGCTGCCGCCTGCATCACCGTCTGCCGTGACGGGGAAATCGTCTGTTTTCTTTTCGTCCAGGTAGCGCACATTAACCGGTTTGTCGTAAACATATCCGTTCGGGCAGACGGTTTTACCGCCTTTTTCCATACAACCTATAGGGATGGATTTGCCTTTGGAGGTTTTCTTATGGCCGGAATATTGCGCAACCGAATTGTGATCCGGCACAATCGGGCGCGCGCTTCTCGGAGTCAATGCGCCGCCGTCGGCGGTATTGATGCCCAAAATGGCGGTTTCCGCGCCGCAGCCGCCGTCTTTATATTTGCGGATGGTTACGAAGGCGGTACGCAATACCACGGTCGGTTTGACGGTAACGCGTTCTCCTTCCCTCAATTTCACTACCCAGCCTTTATCGGCCGATCCGCCGGATGCCTTATTATTTGTCAGGAACAAGGTTTTATTTTCCTGCGTAAGGTTTTGCTTGAGCAGCCCGTCGCCCATGTGGTCTTTTACCTTTACACTAACATTCGCCTTATCGTCGTCAAAGATACCGTAAATATATTGTTCGTCCGTATTGAATACGTCACTTTCACTCAAATCGCTGCCCGTACCGAAGATAACGACGCGTTTGTCTGCCAGTCGGGAAACGGCGGGCGCGGAGGTAATCGGCCTCTGGCCTTCGAAAATAGTGCTTACAGACCATTTACTAGGATTGGAATCGCTCAAATCAAAGCGGTACATATTCCCGCCCCGGTCGCCGGCATAGGCGATATCGACTGTGCCGTCCAAATCTTTATCCACCAGCGTGGGGGACGAAAGCCCGCCCTTGCCGCTGGGTGCTTCGATTTTTTTAATCAGACTACCACTGCCGTTTTCCAAATCATACACATACAGCGCGGTTTTATTGTCGTTGCTGTTAATGTCTTTAGCCGCATAACCGGAGGCGAGGAAGGCGGCGTATTTGCCGTTTTGGGTTTTGCCGATTTGCGGCGTACCGACGGTGTAGCCTAATTTCACGCGATTATTGTCATTCTTATTGTTATTTTTGTCGTTTTGGACATCAAACATGGAAACGCCGGTCAGGTTGCTGCTGTCGATTTTGCTTAAATCCAAGGCATACGCGCCTCTGCCGCCCAGGCCCATCGCACCAAACATAAAGAAATGTTTTTGCTTGTCTTGGTCATCTGTAATGCGGCGCAAGACAAAGCCGCCGTCCACGCCGTAGCGGTCGCCCACATAGCCTTTTTCGGCAAAGGTGCGCAGCTCTTTGGCGAGGTCGGAGTCATTGCCTTCAATATCCTTACGCTCCATCGTACCGGGGATGTAGCTGAGCTTCAGTTCGTAGCCTCGTTGGTCTGTGCCGTTTCTTTTAAACAGGTGCACCATCCCGTCGTTGGCAGAAGTTGCCAGATACCCGCCGACCGCCGTTATCGGGCTGTTGACGATGTCGCCCAAATCGCGGGGTTTGTCATTGTCATCTTCTTTTGTGCGGATGCGGTATTTTTGGCTGTATTGTTTTTTGCCGTTTTGTGTTTTGCTGTTTGGTTGGTTGAATGTTTTAAATATATCGTCATTACCGTAATACCGGACCGTCCAAGGCAGCAGCACTTTTGCCCACTCGTCGGCATCAGGTGTGACTAACCTTTCCTTGTAGATGCCGAAAGTGTCGTTTTTGCCGTCATTGCCATTCAAACCCACGATCCTGTCGCGAGTCGGGGCGATCCGGTACACGCCGCCCGGCAATCGGATGACGGTCTGCCTTGAGTTGAAATTCGGCTCGCGGGATTTGATATCCTGCGCATTCAAAGCGGCGAGGGAATACCGGCCGGGCCTGCCGGGGTCGGTTTGAGTTTTCAGCTCTTGGAGGAAGATGCGGCTGCTCGAACTGCCGGGGTAGGTAGAAACCGAAGCGGAATACATCAGCA

>149 |ref|NC_017511.1| Neisseria gonorrhoeae TCDC-NG08107 | Coordinates: 1845521,1847240 | Reverse

TATTGGGGCGGTTAAGTTGTTGGGAAAGGTTGCCGCAACTTGGAGAATGCCCTCTCCCCGGCCCGCCATTGCCGCGCAGGCGGCAATGGCGGGCCAAGGAGGCGGTTGCGGATTGCCAAAAACCGCTGTACCATGGATAAGCGCGCAAGGGTAGAATAATGCGGCAACCCTATACATTGCACCCCGTCAGAGGGGCGCGTTACCTTTGCGAACATCCCCCTTTGGCAGCCGGGCGAAGGGGGCTTTGCAACCGGCAATCCGGCGGGCGCGGGATCGGGCGGTTTGCCGAATCCCGCCGTTTGCGGCGCGCCTGCCGCCGACGGTATCCCGCGAAGCAAGATTTAAGGGATAAAATATGTTCCAACACGCAGGGCGGCACATAAGGCGCCGCCCTGATTCGGAAGGGCTTGCACCCCTCCCGGACAAAGCCTGATCCTGCCGCCCCGAAGGGCGGGGGGTTTGACCGAAAAGGAAATACGATGAATAAAACTTTAAAAAGGCGGGTTTTCCGCCATACCGCGCTTTATGCCGCCATCTTGATGTTTTCCCATACCGGCGGGGGGGGGGCGATGGCGCAAACCCATAAATACGCTATTATCATGAACGAGCGAAAGCAGCCCGAGGTAAAGTGGGAGACTCAATATAGTCAATCAGCATTAAAGGACAAAGGCAGGGAGCGGACATTTAGCCATACGAGCCAGAAAGGCAGGTTCAACATCACACACAATTTTATCTCATTCAACAATAACGATACCCTTGTTTCTCAACAAAGCGGTACTGCCGTTTTTGGCACAGCCACCTACCTGCCGCCCTACGGCAAGGTTTCCGGCTTTGATACCGCCGAGCTGAACAAGCGCGGCAATGCCGTCAATTGGATTCGTACCACCCGGGCCGGGCTGGCAGGCTACGCCTACACCGGTATCCGTTGCGGACATGCCCGAGACTGTCCCAAACTTACCTATAAAACCCAATTTTCCTTCGATAATCCCGACTTGGCAAAAACAGGAGACAGGCTGGATAGGCACACAGAGCCAAGCCGCGACAATTCGCCCATTTACAAATTGAAGGATTATCCATGGTTGGGCGTGTCTTTCAATTTGGGCGCCGAGGGTACCGCCAAAGATGGCAGATCATCCAGCAGATTGATATCTTCTTTTAATGAAAAGAATAGTAATAACAACCTCGTCTATACCACGGAAGGCCGCGATATTTCCTTGGGCAACTGGCAGAGCGAAAGTACCGCCGTGGCCTATTATCTGAACGCCAAGCTGCACCTGCTGGACAAAAAAGAGATTAAAGATATCACCGGCAAAACAGTTCGGTTGGGTGTCTTGAAGCCGAGCATCGATGTGAAGACACAAAATACGGGGTTTGCCGGCTTGCTAAATTTTTGGTCTAAGTGGGACATTAAAGATAACGGGCAGATTCCGGTCAAGCTCGGCCTGCCGGAAGTCAAAGCCGGGCGCTGCACCAACAAACCGAACCCCAATCCCAAATCCAAAGCCCCTTCGCCGGCACTGACCGCCCCGTGCTTGGGCGCCTTAGGGAACCGTTCCCTTTGAGCCGGGGCGGGGCAACCCGTACCGGTTTTTGTTAATCCGCTATAAAAGGCGGGCTATAGGGTAGGCTTCATCCCGCCAATCTCACTGAATCCGTCAATTTCCACAATTCAATTAAATACCGTCAAACCGATGCCGTTATTCCCGCGCAGGCGGGAATC

>7 |ref|NC_017511.1| Neisseria gonorrhoeae TCDC-NG08107 | Coordinates: 46923,47109 | Forward

CTGCGCGGCAATGGCGGGCCGGGGAGAGGGCATTCTCCAAGTTGCGGCAACCTTTCCCAACAACTTAACCGCCCCAATACAAGCGCTTGTTGCCCTCTCTCCAGCCCTCTCCCACGGGGAGAGAGGACGGGGAGGCTGTTGGGGTTAAGGGTTTTGTAAACTAATCAGGTTGTGCAGGCTGCTTTTT

>8 |ref|NC_017511.1| Neisseria gonorrhoeae TCDC-NG08107 | Coordinates: 47110,58636 | Forward

CCGCCCCAATACAAGCCTTGCGGCTTGTTGCCCTCTCTCCAGCCCTCTCCCACGGGGAGAGAGGACTATGAAGCCCGCCGGCAGGCGCGGGGCTTGGGACGGCATTGCTGTTGCGGTTTCGGGCGCGGCTTTATTCGACGACAATTTTAGGGAACCGGCTGCTGAAGTCTTTGCCTTTGTCGGCAATGCCCAGGGCGATTTGGAATGCGGCATCGGTGTAGATTCGGGCGATGGCGGGGTGTTCGTCGAACAGTTGCGCCGGTGTGCCGCCGTCCATGGCTTCGCGCACGGGCAGGCTTAGGGGAAGCTGTCCGAGCAGGGGGACGTTGAGGCGGGCCGCCAAATCTTTGCCGCCGTCCGTGCCGAACAGTGCTTCGCTATGCCCGCAGTTGGAGCAGATGTGGACGGACATGTTTTCCAATACGCCCAAAATGGGAATGTTGACCTTGCGGAACATATCCACAGCCTTGCGCGCGTCTATCAGCGCGATGTCCTGCGGCGTGGTTACGATGACGGACCCGGTTACGGGGATGCGTTGGGACAGCGTGAGTTGGATGTCGCCCGTGCCGGGGGGCAGGTCGATGAAAAGATAGTCCACTTCGTCCCACTCGCTTTGGAACATCAGCTGCTGCAAGGCTTGGCTGACCATCGGCCCGCGCCAGACGACGGCTTGGCCGGTATCGACTAGAAAGCCGATGGACATAACCTGTATGCCGTCTGAAGATTCGACGGGAATGAGTTTTTGGTTTTTCTGATCGGGTTTGCGGTCGTGTACGCCCAGCATCGTGGGCTGGCTCGGACCGTAAAGGTCGGCATCGAGCACGCCGACGCGCGCGCCCATGCGCGCCATTGCGGCGGCAAGGTTGGCAGTGGCGGTTGATTTGCCCACGCCGCCTTTTCCCGATGCGACGGCAATGATGTTTTTCACGCCTTTGATGGTGGCGACGCCGGGCCGGACTTTGTGTGTGCCGATTTCGGTGTCTATGGACAGATGGATGTGTGTGTCGCCTGTCAGCGGCATCAGGGTTTCTTGTACGGCATCGGCGAGGGCGGCGGCGATGTGCGCGACAGGGAAGCCGAAATGCAGGGCGATGTGGATGCCGTCTGAACGCTGTCCGACCGAGCGGACGGCCTTTTCGCCGCCGAGCGTGCGTGCCGTATTCGGAACGGCGACGGTGTCGAGGAGGGTGCGGATGTTTTGTATATTCATGATGTGTGCCTCTGTTCGCAAGTCGTGTGAGGGGAAGATAGACGGAAAGTGTACATGATTTCGGGCGGTTTGCTTTTTTCTTGTGCGAACTTTTACACGGTATGCGGCGGATTGTGAAAATCCGTTGTCAAAATATCCCGTGCAAACAGCAGTCTGCAATTAAGCCTGTAAAATTTGCTTGACGGCGGGGAACGGGGCTGTATAATGCACGGCTTATCGGGTCGTTAGCTCAGCCGGTAGAGCAGCGGACTTTTAATCCGTTGGTCGAGCGTTCGAATCGCTCACGACCCACCAGATAACCGGAAGCCAAGTTTCGAGACTTGGCTTTTTTGTTTGCCTGCCGGTTGCGCGGGCGTACCGTTTGCGGGGGTGTATGAAAAAGAAACTGCTTTCGGGCATCAAATTTGCCGTTCGGACGGCATTGGTGTTTTTGCTGGTGTCGCTGTTTTTGGATTGGGTACGCAAGCCCGACGAACCTGCCGGGGCGGCAGGGCGGCCTTTGACCCTGCTGTCGGGGCAGCGGCTGACTTTGGGACAGTTTAGCCGGGATAGGACGGTGCTGGTGTATTTTTGGGGAAGCTGGTGCGGCGTGTGCCGTTATCAGTCGCCGATAATCGATGATTTGGCGGCGGACGGCGTGCCGGTCGTCGGCGTGGCGGTACGTTCCGGCAGCGCGTCCGAAGTGGCGGCATATATGGCAAAACGGGGCTTGGGCTTTCCGACTGTCAGCGATGAGGACGGGGGTTTGGCAAGGTCTTGGCGGATTGCCGCAACGCCTGCCGTCGTTTTGGTCAAAAATGGGAAAATGGTCCGCTATACGACGGGAATCAGCAGTTATTGGGGCTTGCGCGCACGAATTTTTCAGGCGGATTTTTTCGGTTAACTTTTGTTTTTGTCAAACTTTCCGCTATGTAGAGAATCAAACGGCATATGCCTTGCGCGGCGGATGCGGCGGCTTTTGTTTTTTGGGAAAGAGCCGGCGCGCCTGCCGATGTAATCCCTTGTTGTGATTGTGGGAAAAATAGATTAAAATATAACTATTAAAATATTTTCAGATAGGATTATCGGAATTAAAGTCTTTTATACTCGGTCGTCCGATGAGGTTTATAGCGTATTGTTGCTATATGTTCGTTTTGTTATATAACGGTTGCATCAAAAATTACGCCCACAGGCTTTCCCGACGGTTTGAAAGTTTGATTTTCGATAACTTGGAGACTTAAACAATGCCTACCCAATCAAAACATGCGTCTATCAATATCGGTCTGATACAGGCAAGGGAAGCCCTGATGACCCAATTCAGGCCTATTCTGAATCAGGCGAATATTACCGATCAGCAATGGCGGATTATCCGTCTTTTGGCGGAAAACGGCACATTGGATTTTCAGGATTTGGCGAATCAGGCGTGCATTTTGCGCCCCAGCCTGACCGGTATCCTGACCCGCCTTGAAAAAGCGGGGTTGGTCGTCCGCCTGAAACCTTCCAACGACCAACGGCGCGTTTATCTGAAGCTGACTTCCGAGGGCGAGAAGCTGTATGAGGAAATCGGCGAAGAAGTGGACGAGCGTTACGACGCTATCGAGGAAGTGCTGGGCCGCGAGAAAATGCTGCTGCTTAAAGACCTGTTGGCAGAACTTGCCAAAATCGAGGATGCGTTGAACTCGTAATACGCCGTAATGCGCGGAAACGTCCGACCGACGGCTTTTTGAATCAGAACTGCTGCACATGGGGGATGCCTTGTGTGCAGCATTCTTATATAGGGGACGGTTTAAAGGGGAAAAATGGCGGATTTGCAGAAAACTTTTCAAACTTCGTTCCGTGATGCGATGGCATCCTGTGCGGCAGGCGTTCATGTCATCACGACAGACGGTGCGGCAGGGCGTTACGGCATTACAATGACGGCGGTTGCGCCGGTTACCGACGAGCCGCCGACCGTGATGCTGTGCATAAACCGGAGTGCGCGAATCATTCCGATCCTGTCGGAAAACGGCAGCCTCTGCATCAATATGCTGGCGGACGAACATCAGGATGTTGCCGAACATTTTGCCGGGCTGACCGGCCTGTCGCCCGAAGAGCGGTTTGCCTACCATATTTGGCATCGCGGCAAAACGGGACAACTTGAAATAGAAGGCGCGTTGGCGCACCTGCACGGGCATATTGTCGGCAAACATGAAATCGGCACGCATTTTGTGTTTTACGTCAGGCTCGACGAAATCAAAAACTGCGGGTGCAAACGCCCCGCGCTGCTGTATTTCAGACGGCAGTTTAGGCCTTTAGACTGATATTCGGACAGATATATGAAAGCGATGATACTGGCGGCAGGACGCGGCGAGCGTATGCGCCCTTTGACCGACACCACTCCGAAGCCGCTGCTCGATGTGGCGGGTAAGCCTCTAATCGGTTGGCACTTGTGCCGTCTGAAGCAGGCGGGGTTTACCGAAATCGTCATCAACCACGCTTGGCTGGGTCGGCAGATAGAAAATGCTTTGGGCGACGGCTCGGCTTATGGCGTGAACATCGCCTATTCGCCCGAACCCGCAGGCGGTTTGGAAACGGCAGGCGGCATCGCGCAGGCATTGCCGCTGTTGGGTGGGCAGCCGTTTTTGGTGGCCAACGGCGACGTGCTGACCGACATCGATTTTACCGCCGCGTTTCAGACGGCATCGTCCCTGCCCGGGCACATTTCCGCACATTTGTGGCTGGTGGGAAATCCCCCGCACAACCCCGACGGCGATTTTTCCCTGCTGCCCGACGGCAGCGTGCGGCCGGAAGTATCCGGCGGCAACGGACTGACATTCAGCGGCGTGGGTATTTACCGTCCTGAAATGTTTGACGGAATCGAAGCGGGCAGTGTGGCAAAACTCGCGCCCGTATTGCTGAACGAAATGCGGCAAAACCGCGTGAGCGGTCAGAAGCATACGGGCCTGTGGCTGGATGTCGGCACGGTATGCCGTCTGAAGGAAGCGCAAGCCCTTGCAGCGGCTTGGAAGTGAAAACTCGGTTTCAGGCGGTATGGCGGATTCGGTTTAACGTTTCAGTGCCAACGTCAACACGCCGCCGTAACCAGCCCCAAGTCTATCCATTCCTGCGTGTTCGGGCGTTCGTCTAAGAAAATCACCGCCATCAGCGCGACCAAGACCAGGCTGAATTTGTCGACGGGCGCGACTTGCGGGGCTTTGCCCAGTTGCAGGGCTTTGAAGTAGGCGAGCCAAGATGCGCCGGTGGCGAGTCCGGACAAAACCGGAAACGTCCGGTTGCGCCCCGTGAAGCCGTTTACGCCCTGCCATTTGCCGGTGTAGGTCAAAAACAATACCAAGGCGGCGAGGATGACCAAGGTGCGGATAAAGGCGGCGAAATCCGAATCTATGCCCTGCAAGCCCATTTTGGCGAAAACGGCGGTCAATGAGGCGAAGCCTGCCGATGCCAATGCCCAAAACAGCCATGCGTTGCTGCCCATGTTTTCTCCTTTGATTGTGAACAATATGAACGGTATTTTTGTTGCTGCGTCAAAAATTTCACTGCGGGTTTGGTGCGGATAACGTTATAATATGCCTGATATTATTTTCAATCCACCTGTTTGTCGCCTGATGCTTTCAGACGGCATGTCCCTCCTCATTTCTAAAGGAAAATCATGAGCTTCAAAACCGATGCCGAAACCGCCCAATCCTCCACCATGCGCCCGATTGGCGAAATTGCCGCCAAGCTGGGTTTGAACGTTGACAACATTGAGCCTTACGGTCATTACAAAGCCAAAATCAATCCTGCCGAAGCGTTCAAGCTGCCGCAAAAACAAGGCAGGCTGATTTTGGTTACCGCCATCAACCCGACTCCGGCGGGCGAAGGCAAAACCACCGTAACCATCGGTTTGGCGGACGCATTGCGCCATATCGGCAAAGACTCTGTGATTGCTTTGCGCGAGCCTTCTTTGGGTCCGGTGTTCGGCGTGAAAGGCGGCGCGGCAGGCGGCGGCTACGCGCAAGTTTTGCCGATGGAAGACATCAACCTGCACTTCACCGGCGACTTCCACGCCATCGGTGCGGCGAATAACCTCCTCGCCGCCATGCTCGACAACCATATCTACCAAGGTAACGAGTTGAACATCGACCCCAAACGCGTGCTGTGGCGGCGCGTGGTCGATATGAACGACCGCCAGTTGCGCAACATCATCGACGGTATGGGCAAGCCTGTTGACGGCGTGATGCGTCCCGACGGCTTCGACATCACCGTCGCCTCCGAAGTGATGGCGGTATTCTGCCTTGCCAAAGACATCAGCGATTTGAAAGAGCGTTTTGGCAATATTCTCGTCGCCTACGCCAAAGACGGCAGCCCCGTTTACGCCAAAGATTTGAAGGCACACGGCGCGATGGCGGCATTGCTAAAAGATGCGATTAAGCCCAATTTGGTGCAAACCATCGAAGGCACTCCGGCCTTTGTACACGGCGGCCCGTTCGCCAACATCGCCCACGGCTGCAACTCCGTTACCGCAACCCGTCTGGCGAAACACCTTGCCGATTACGCCGTAACCGAAGCAGGCTTCGGCGCGGACTTGGGTGCGGAAAAATTCTGCGACATCAAATGCCGCCTTGCCGGTTTGAAACCTGATGCGGCAGTCGTCGTGGCGACTGTCCGCGCCCTGAAATACAACGGCGGCGTGGAACGCGCCAACCTTGGTGAAGAAAACCTCGAAGCTTTGGCAAAAGGTTTGCCCAACCTGTTGAAACACATTTCCAACCTGAAAAACGTATTCGGACTGCCCGTCGTCGTTGCGCTCAACCGCTTCGTGTCCGACTCCGATGCCGAGTTGGCGATGATTGAAAAAGCCTGTGCCGAACACGGCGTTGAAGTTTCCCTGACCGAAGTGTGGGGCAAAGGCGGCGCGGGCGGCGCGGATTTGGCGCGCAAAGTCGTCAATGCCATCGACAACCAACCTAATAACTTCGGTTTCGCCTACGATGTCGAGTTGGGCATCAAAGACAAAATCCGTGCGATTGCCCAAAAAGTGTACGGCGCGGAAGATGTCGATTTCAGCGCGGAAGCGTCTGCCGAAATCGCCTCGCTGGAAAAACTGGGCTTGGACAAAATGCCGATCTGCATGGCGAAAACCCAATATTCATTGAGCGACAACGCCAAACTCTTGGGCTGCCCCGAAGGCTTCCGCATCACCGTACGCGGTATCACTGTTTCCGCCGGCGCGGGCTTCATCGTTGCGTTGTGCGGCAATATGATGAAAATGCCGGGCCTGCCGAAAGTTCCGGCTGCCGAGAAAATCGATGTGGACGAACACGGCGTGATTCACGGCTTGTTCTGAACGGTTTTTGAAACCGGATGCCGTCTGAAGCCGTTTCAGACGGCATTTTTTCGGAACGCGGGCGGCGGTATGCTATAATTCTCCGTTAAATTTCTCTATTTTCAGGAAAAACCATGAGTTTGAAATGCGGCATCGTCGGTTTGCCCAACGTCGGCAAATCCACCCTTTTTAACGCGCTGACCCAATCGGGCATCGAAGCGGCAAACTATCCCTTCTGCACCATCGAACCCAACGTCGGCATCGTCGAAGTGCCCGACCCGCGTATGGCGGAGCTGGCGAAAATCGTCAATCCGCAAAAAATGCAGCCCGCCATCGTCGAGTTTGTCGATATTGCCGGCTTGGTTGCGGGCGCGAGCAAAGGCGAAGGCTTGGGCAACCGGTTCCTTGCCAACATCCGTGAAACCGATGCCATCGTCAACGTCGTGCGCTGCTTTGACGACGACAACATCGTCCACGTTTCCGGCAAAGTCGATCCGATTGCCGACATCGAAACCATCGGCACCGAATTGGCGCTTGCCGACTTGGCAAGTGTGGAAAAAGCCATCGTCCGCGAAGAAAAACGCGCCCGATCAGGCGACAAAGACGCGCAAAAACTAGTCGATTTGTGCAAAAAACTGCTGCCGCATCTGGACGAAGGCAAACCCGTGCGTTCCTTCGGTTTGGACGCGGAAGAACGCGCGCTGCTGAAGCCGCTGTTCCTGCTGACCGCCAAACCTGCGATGTATGTGGGAAACGTTGCCGAAGACGGTTTTGAAAACAACCCGCACCTCGACCGCCTGAAAGAATTGGCGGCAAAAGAAAACGCCCCCGTCGTTGCCGTCTGCGCCGCGATGGAGAGCGAAATCGCCGAATTGGAAGACGGTGAAAAAGCCGAATTTCTCGCCGAAATGGGCTTGGAAGAACCGGGCTTGAACCGCCTCATCCGCGCGGGTTACGACCTTTTGGGACTGCAAACCTACTTCACCGCCGGCGTGAAAGAAGTCCGCGCGTGGACGATACACAAAGGCGACACCGCGCCGCAAGCCGCCGGCGTGATCCATACGGATTTTGAACGCGGCTTCATCCGCGCCCAAGTGATTGCCTACGATGATTTTGTCTCGCTCGGCGGCGAAGCCAAAGCCAAAGAAGCCGGCAAAATGCGCGTGGAAGGCAAGGAATACGTCGTGCAGGACGGCGACGTGATGCACTTTTTGTTTAACGTGTAACCCAAATGCGGCAGGTTTTCAGACGGCTTTGCCGGAAATGCCGTCTGAAGCCGGTTTTGGTGGTTTTCGACGTTCCCATACCGCCGGAATGCAGCCGCATCAAAATAAAATCCCGCCCGCATTTCCGATTTGCCCTCCCCGATTCCTGCAAAACAAACCGCCTGCCCTGCCGTTACGGGAAGCCGTCCGGTATTCCGAATATCCCGAACCCCGATACAAAATGACCTTTCAGACGGCATTTGCACAGCCCGCCGCGTTTCAAGTAAAAACATTATGAGCCAAGCCTTACCCTACCGCCCGGACATCGACACATTGCGCGCCGCCGCCGTCTTGTCCGTCATCGTGTTCCATATCGAAAAGGATTGGCTGCCGGGCGGGTTTCTCGGTGTCGATATATTCTTTGTGATTTCAGGCTTTTTGATGACGGCGATCCTCCTTCGCGAAATGTCCGGGGGGCGTTTCTTCCTCAAGACATTTTATATCCGCCGCATCAAACGGATTTTGCCCGCATTTTTCGCCGTATTGGCGGCAACGCTGGCAGGCGGCTTCTTTTTATTCACCAAAGATGATTTCTTTCTTTTGTGGAAATCCGCGCTGACCGCCTTGGGTTTCGCCTCCAACCTGTATTTTGCAAGGGGGAAGGATTATTTCGATCCCGCGCAGGAAGAAAAGCCCCTGCTGCACATCTGGTCTTTGTCGGTCGAAGAACAATTTTACTTTGTCTTTCCGATATTGCTGTTGCTTGTCGCCCGCAAAAGCCTGCGCGTACAGTTCGGCTTCCTCGCCGCATTGTGCGCCTTAAGCCTTGCCGCTTCCTTTATGCCTTCCGCGCTCGATAAATATTACCTGCCCCACCTGCGCGCCTGCGAAATGCTGGTCGGATCGCTGACCGCCGTGCGGATGCGGTACCGGCAACAGCGGAATCCCGCCGTCGGGAAACGGTATGCCGCCGTCGGCGCATTGTTTTCCGCGTGCATACTGTCCGCCTGCCTGTTTGCCTATTCGGAACAAACCGCCTATTTCCCGGGCCCCGCCGCCCTGATTCCCTGTCTGGCTGTTGCCGCGCTGATTTATTTCAACCATTACGAACACCCGCTTAAAAAATTTTTCCAATGGAAAATCACCGTTGCCGCCGGTTTGATTTCCTATTCGCTTTATCTGTGGCATTGGCCGATATTGGCCTTTATGCGCTATATCGGCCCGGACAACCTGCCGCCTTATTCGCCGGCGGCAGCGATCGTCCTGACCCTGGCGTTTTCCCTGATTTCTTATCACTGCATCGAAAAGCCGTTTAAAAAATGGAAAGGCTCGTTCGCACAATCCGTTTTATGGATTTATGCCTTGCCTATGCTCGTTTTGGGGGCGGGCTCGTTTTTCGCGATGAGGCTGCCGTTTATGGCGCAATACGACCGCTTGGGGCTGACGCGTTCCAACACCTCCTGCCACAACAATACCGGCAAACAATGCCTGTGGGGGGATACGGAAAAACAGCCGGAACTGCTGGTTTTGGGCGACTCCCACGCCGACCATTACAAAACATTCTTCGATGCCGTGGGCAAAAAAGAAAAATGGTCCGCCACTATGGTTTCCGCCGACGCCTGCGCCTATGTGGAAGGCTACGCGTCCCGTGTGTTCCAAAACTGGGCCGCCTGCCGCGCCGTTTACCGCTATGCCGAAGAACACCTGCCCCGGTATCCGAAAGTGGTTTTGGCGATGCGCTGGGGCAGCCAGATGCCCGAAAACAGCCGCTCCCTTGCCTACGATGCCGGTTTTTTCCAAAAATTCGACCGTATGCTGCACAAACTCTCATCCGAAAAACAAGCCGTTTACCTGATGGCGGACAACTTGGCTTCGTCTTACAACGTCCAGCGCGCCTATATCTTGTCTTCACGCATACCGGGTTGCCGCCAAACACTGCGCCCGGACGACGAAAGCACCCTGAAAGCCAATGCCCGCATCAGGGAATTGGCAGCCAAATACCCCAACGTCTATATTATTGATGCCGCCGCCTATATCCCCGCAGATTTCCAAATCGGCGGATTGCCGGTTTACTCGGACAAAGACCACATCAACCCTTACGGCGGCACAGAATTGGCGAAGCGTTTTTCCGAAAAACAAAGGTTTCTCGATACGCGCCATAACCATTGATTCGCTTAAATTTGTTACAATCGGCGGTTTGCAAAACCGCTAATTTTTTTGAAAGAGACCGATGAGCGTCATCCAAGACCTGCAATCGCGCGGCCTTATCGCGCAAACCACCGACATCGAAGCCTTAGACGCTTTGCTGAACGAACAAAAAATCGCCCTTTACTGCGGTTTCGACCCGACAGCCGACAGCCTGCACATCGGACACCTGCTGCCCGTATTGGCATTGCGCCGCTTCCAACAGGCGGGGCATACGCCGATTGCACTGGTGGGCGGCGCGACCGGTATGATCGGCGACCCCAGCTTCAAAGCCGCCGAACGCAGCTTGAATTCCGCCGAAACCGTTGCCGGCTGGGTAGGAAGCATACGCAGCCAATTAACCCCTTTCTTGAGCTTTGAAGGCGGAAACGCCGCCATTATGGCGAACAATGCCGACTGGTTCGGCAGCATGAACTGCCTCGACTTCCTGCGCGACATCGGCAAGCATTTCTCCGTCAACGCCATGCTGAACAAAGAATCCGTCAAACAGCGCATCGACCGCGACGGCGCAGGCATTTCCTTTACCGAGTTCGCCTATTCCCTGCTGCAAGGCTACGACTTCGCCGAGTTGAACAAACGCCACGGCGCGGTTTTGGAAATCGGCGGTTCCGACCAGTGGGGCAACATCACCGCCGGTATCGACCTGACCCGCCGCCTGAACCAAAAACAAGTGTTCGGTCTGACCCTGCCTTTGGTTACCAAATCCGACGGTACCAAATTCGGCAAAACCGAAGGCGGCGCGGTGTGGCTGAACGCGAAAAAAACCTCGCCGTACCAGTTCTACCAGTTCTGGCTGAAAGTCGCCGATGCCGATGTGTATAAATTCCTGAAATACTTTACCTTCCTGTCCATCGAAGAAATCGGTGTCATCGAAGCCAAAGACAAGGCAAGCGGCAGCAAGCCCGAAGCGCAACGCATCCTCGCCGAAGAAATGACCCGCCTGATTCACGGCGAAGAAGCCCTTGCCGCCGCGCAACGCATTTCCGAAAGCCTGTTTGCCGAAGACCAAAGCCGGCTTACCGAAAGCGACTTCGAGCAGCTCGCCCTCGACGGCCTGCCTGCATTTGAAGTTTCAGACGGCATCAACGCCGTCGAAGCCTTGGTCAAAACCGGCTTGGCAGCGTCCAACAAAGAAGCGCGCGGCTTTGTCAATGCCAAAGCGGTTCTGCTCAACGGCAAACCGGCTGAAGCCAACAACCCCAACCACGCCGCCGAACGCCCCGACGATGCCTATCTGTTGATAGGCGAATACAAACGTTTCGGCAAATACACCATCCTCCGGCGCGGCAAACGCAACCACGCGCTTTTGGTTTGGAAATAATCCGATTGCCGCAGAAATGCCGTCTGAAGCTTTCAGACGGCATTTTTATCAAATGCAAAACACCCTGCGCCTGCCGATATGTCGTCATTTCCATGCAGGCGGGAATTCAAACTTGTCCGCACGGAAACTTATCGGGCAAAACGGTTTCTTCAGTTCTACGTTCTAGATGCCCGCTTTCGTGGGAATGACGAATTTCGGGAAACTTATGAATTGTCATTCCCGTGAAAGTGGGAATCCAGGACTCAAAATCTCAAGAAACCGTTTTGCCCGATAAGTTCCTGCACTGACAGATCTAGATTCCC

>9 |ref|NC_017511.1| Neisseria gonorrhoeae TCDC-NG08107 | Coordinates: 58637,59778 | Forward

CCGATAGATTCCCGCCGCGTCGGGGGTCCGGATTCCCGCCTGCGCGGGAATGACGGGTTTCGAGATTGCGGTGTTGTCGGGAATGATGGAAAATGGCGGGAATTGTGTAAAAAATGCCGTCTGAAACCGTTGGAAGCATCGTAAACGTTGGAGTCGATGAATCGGTGGGCTTCAGTCCGCCATTCCCATCAACCCAACATGTCTACCGTTTTCATCGAATCCATCGAATCCGCCCTTTCGACCACCCGGCCCTACGCAACCGAACCGTCATTCCGAGCATTTGTGGTAACACTTGGCTTAACACCCTGTTCCTTTGGGTAAGTGGTAACAGCAACGGTTTCTTGTTGAACCGAACGAACCTGATGTCTGACGTGTCCGTAGGCGACGCGCATGCCGATATAGGGTTTGAAGCGGGAACCGGTATCGAAATCGTAAATGGTTGACAAGCCGAGAGAAGAAACGGCGTGGAACGTACCGTTTTCCTGATGTTCCGTCTTCAAGGTTTGCTCGTTGCGAAGCCTTTCCGCCGAAGTCGTAGCCGACCGACACCCTGGGGTGGATGGAATGCGTACGGATGTTTCTGAAATAATCGCTTACCGTGCTTATTTTGTTTTTGCCTGTACCGGTTGGTTTCGGATAATCGTGGGTAATGCGTTCGGCGGCGTAGGCTAAATCCGCCTGCACATACGGGCCGCGGCCATTGCCTTCACCCGCCGCCTGCGCTGCGACCGACACCCTGGGGTGGACGGAATGCGTACGGATGTTTCTGAAATAATCGCTTACCGTGCTTATTTTGCCTTTTTTTTGAGCGGTTGGTTCCGGATAATCGTGGGTAATGCGTTCGGCGGCATAGGCTAAATCCGCCTGCACATACGGGCCGCGGCCGCCGTCTTCACCGGTTTGACCGGTTAAAAAAAAGATTTTCACTGATGCTTCAAAGGCGGATTATATCGGGTTCCGGGCGGTGTTTCAATACATAGCACCGCGCCTGCTGCGCGTTTTATGCGTTTGGCGCGTTCGGCGGCGGGAAATTTGCCTACTTTTCCCGCGTCGGGCGGGCGGAACGGGCGGCACACTGTCTATAAACCGCAATACCGTTTACAATGACCGCCTGTTTCACCACATACCCGAACGCAACAATG

>10 |ref|NC_017511.1| Neisseria gonorrhoeae TCDC-NG08107 | Coordinates: 59779,63891 | Forward

TTTCAACACACGGGACGGCACATCAAGCACCGCCCTATGTGTCGTCCTGATTTGGAAGGGGTTGCACCCCTCCCGAATAAAGTCTGATCCTGCCGCCCCGAAGGACGGATGTCCGAGTGGCGGGGTTTCAACCGAAAAGGAAATACAATGAAAATCAGGCCGGGGCGGCACAACGCGCCCGACTTTCCGCACGGGGCCGCCGTAACCATAGGCAATTTCGACGGCGTACACCTCGGACACAAACACATCCTCCAAAAACTCCGCCTCGAAGCCGATACGCGCGGATTGCCCGTCGTGGCCGTCGTTTTCGAACCCCAACCCAAAGAATTTTTCGCACTCCGTACCGGCAAAACCCCGCCCTGCCGTATCAGCCCCCTGCGTACCAAACTGGAATTGCTGGAAGGGACGGGTTGCGTCGATGCCGCCTGGGTTTTGCGTTTCGATCGGAATTTTTCCGAAATATCCGCGCAAGCATTTATCGACCGCCTGCTGCGTCAAACCTTGAATACGCGCTATTTGCTCGTCGGCGATGATTTTCGTTTCGGCGCGGGGCGCGAAGGCTGTTTTGAACTTTTGGCACAACAGCCCGATATGCAAACCGAGCGCACGCCTTCCGTCATCGTCGAAGACATCCGCACCAGCAGCACCGCCGTCCGCCAAGCCCTTTCAGACGGCAACCTTGCCTATGCGAAAAAACTTTTGGGACACGACTACGTTTTGGGGGGCAGGGTGGTGCACGGCAGAAAACTCGGGCGCACCTTAAACGCCCCGACCGCCAACATCCGACTGCCCGGCCACCGTTATGCACTCGGCGGCGTGTTCGTCGTCGAAGCGGACGGCGCATTCGGCACGCGGCGCGGCGTGGCGAGCTTCGGCTTCAATCCCACCGTTGATGGCGGCTGTTCTCAAAAGCTTGAAGTCCACCTGTTCGACTTTCAAGGCGACCTGTACGGACAACGGTTGAACGTCCGCTTCCTGCACAAACTGCGCGACGAGGAAAAGTTTGACGGTATGGAAGAACTGAAAAGGCGGATTGAAGCCGATATGGAAGCCGCAAAGTGTTGGTAGAAAAACCTTATACAAACCATCCGATTGGGCTACAATCAGCCTTTTAACTGTTCGGACGGCACAGGGGTTTCCCGTTGTGAAATACTGTTTGAGGCGCAATGCCGTCTGAAACCGAAATATTGTAACAATAGAGATTAAAAAATGACCGATTACAGTAAAACCGTCAACCTGCTCGAAAGCCCGTTTCCGATGCGCGGCAATCTTGCCAAGTGCGAACCTGCGTGGCTGAAAAGCTGGTACGAGCAAAAACGTTACCAAAAACTGCGCGAAATCGCCAAAGGCCGTCCGAAATTCATCCTGCACGACGGCCCGCCGTATGCCAACGGCGACATCCATATCGGTCATGCCGTCAATAAAATTCTTAAAGACATTATTATCCGCAGCAAAACCCAAGCCGGTTTTGACGCGCCTTATGTACCGGGTTGGGACTGCCACGGCCTGCCCATCGAAGTGATGGTGGAAAAACTGCACGGCAAAGATATGCCTAAAGCCCGTTTCCGCGAATTGTGCCGCGAATATGCCGCCGAACAGATTGCCCGTCAGAAAAAAGACTTTATCCGCTTGGGCGTGTTGGGCGACTGGGACAATCCTTACTTGACCATGGATTTCAAAACCGAAGCCGATACCGTGCGTATGCTCGGCGAAATCTACAAATCCGGCTATCTCTACCGGGGCGCGAAACCGGTTCAGTTTTGCTTGGATTGCGGCTCTTCGCTGGCGGAAGCGGAAGTGGAATACAAAGACAAAGTATCGCCTGCGATTGATGTTGCCTATCCGTTTAAAGACACCGTCGCGCTTGCCGCCGCATTCGGCTTGGCAGGTATCGAAGGCAAAGCGTTTGCCGTCATTTGGACGACCACGCCTTGGACTCTGCCTGCGAGCCAGGCCGTGTCTGCCGGCGCGGACGTGGTGTATCAATTAATCGATACGCCCAAAGGCAAATTGGTGCTGGCGAAAGATTTGGCGGAAGGCGCTTTGAAACGCTACGGCTTTTCAGACGGCATCGCCATCCTTGCCGAAACCACCGGCGACAAGCTGGAAAACCTGCACATGAATCATCCGTTCCTCGAACGCGATATTCCCATGCTCAACGGCGAACACGTTACCACCGATGCCGGTACCGGCTTGGTGCATACTGCGCCTGCGCACGGTTTGGAAGACTACGCCGTCTGCAATAAATACGGCATCGAGCTTTACAACCCTGTCAACGCCGAAGGCAAATACATAAGCGAAACGCCTCGTGTCGCAGGCATGAGCGTTTGGGAGGCGAATCCCGTCATCCTGCAATGGCCGGAAGAAACCGGCAACCTCTTGGCAAGCAGCAAAATCGAACACAGCTACGCCCACTGCTGGCGCCACAAAACCCCGCTGATTTACCGAGCGACAGGTCAGTGGTTTGTCGGCATGGACAAAGCCGGCAGCGACGGTAAAACCCTGCGCGACAAAGCCATCAAAGCCGTGGACGACACCGAATTCTTCCCGCCATGGGGTCGTGCGCGTTTGGAATCCATGATTGAAGGCCGTCCTGACTGGGTGGTTTCACGCCAACGCTATTGGGGCACGCCGATGACTTTCTTTGTTCACAAAGAAACGGGCGAGCTGCATCCGAACTCTGCCGAACTTTTGGAAAAAGTCGCGCAACGCATCGAAGAAAAAGGCATCGAGGCTTGGTTCTCCCTCGATAAAAGCGAATTATTAAGCGCCGAAGATTGCGAACATTACGACAAACTCCCCGATACCATGGACGTATGGTTCGACTCAGGCTCGACGCATTATTCCGTTGTAAAACAACGCGAAGAATTGGAATGGCCGGCTGACTTGTACCTCGAAGGCAGCGACCAACACCGCGGCTGGTTCCAATCCTCTATGCTGACCGGTTGCGCCTCATCCATGGGACGCGCACCGTATAAACAGCTGCTGACCCACGGTTTCGTGGTTGACCAAAACGGCCGCAAAATGTCGAAATCCATCGGCAACGTCGTCGCGCCGCAGGAAGTCTATAACGAGTTCGGCGCGGACATCCTGCGCCTGTGGGCGGCATCCACCGATTACAGCGGCGAATTGGCGATTTCCAAAGAAATCCTCAAACGCGTAACCGAAAGCTACCGCCGTATCCGCAATACCTTGAGCTTCCTGTTTGCCAACTTGAGCGATTTCAACCCGATTGAAGATGCCGTGCAACAGGCGGATATGGTGGAAATCGACCGCTACGCCTTGGTATTGGCGCGGCGGCTGCAAGAGCGTTTGGCAGGCGGTTACTATCCGCGCTATGCCTTCCACTTCGCCGTGAAAGACATTGTTTCTTTTTGCTCGGAAGACTTGGGCGCGTTCTACCTCGACATCCTGAAAGACCGCCTCTACACCACCAAAGCCGACAGCCGCGCCCGCCGCAGCGCGCAAACCGCCCTGTACCACATCACGCGCAGCCTGGTTCTCTTGATTGCACCGATTTTGTGCTTCACCGGCGAAGAAGCGTGGGACATCATCGGCGGCGGCGAAGAAGACAGCGTCCTCTTCCATACTTGGCACGAGTTCCCGGCCATCAACGAAAAAGCCGAAGCCGAACTGGTGAAAAAATGGACGGCAATCCGCGAAGCGCGCGAAGCAGTAACCGCCGCCATCGAGCCTTTGCGCGCCGACAAAACCGTCGGTTCGTCCTTGCAGGCGGAAGCCGAAATCACCGCGCCGGAAGAAATGGCCGGCTATCTGAATGCTTTGGGCGAAGAATTGCGCTTTGCCCTGCTGGTGTCTAAAGCAGAAGTGAAAGTCGGTGATGAACTTGCCGTTGCCGCCAAAGCCGGCGACGGCGAAAAATGCGAACGCTGCTGGCACTACACCCGCGATGTGGGCGCGGTTGCAGGCTATGAAACCGTCTGCAAACGCTGTGCGGAGAATGTCGGCGGAGAAGGCGAAACGCGCCATTACGCCTGATAAAGTTTGAGCAAATGCCGTCTGAAACCGCCAAACAGTATTTCAGACGGCATTTTTTGTGCCGCGATTTGTCTTCATAATGGCGGAGGGGTTTTAAGATTACGGTATTGTCGG

>50 |ref|NC_017511.1| Neisseria gonorrhoeae TCDC-NG08107 | Coordinates: 587305,588181 | Reverse

CAGCGCAGGCGGCGGGTGAAGGCAATGGCCGCGGCCCGTATGTGCAGGCGGATTTAGCCTACGCCGCCGAACGCATTACCCACGATTATCCGAAACCAACCGGTACAGGCAAAAACAAAATAAGCACGGTAAGCGATTATTTCAGAAACATCCGTACGCATTCCATCCACCCCAGGGTGTCGGTCGGCTACGACTTCGGCGGGTGAAGGCAATGGCCGCGGCCCGTATGTGCAGGCGGATTTAGCCTACGCCTACGAACACATTACCCACGATTATCCGGAACCAACCGGTACAAAAAAAGACAAAATAAGCACGGTAAGCGATTATTTCAGAAACATCCGTACGCATTCCGTCCACCCCAGGGTGTCGGTCGGCTACGACTTCGGCGGCTGGAGGATAGCGGCAGATTATGCCCGTTACAGAAAATGGAACAACAATAAATATTCCGTCAACATAGAAAATGTGCGGATACGTAAAGACAATGGCAACAGGCAAGATCTGAAGACGGAAAATCAGGAAAACGGTACGTTCCACGCCGTCTCCTCGCTCGGTTTGTCAGCCGTTTACGACTTCAAACTCAACGATAAATTCAAACCCTATATCGGCGCGCGCGTCAGCTACGGACACGTCAGACACAGCATCGATTCGACCAAAAAAACAATAAAGGTTACTACCGTCCCCAGCACTGCTCCTAACGGAGCAGTTACAACTTATAATACTGATCCAAAGACGCAAAACGATTACCAAAGCAACAGCATCCGCCGCGTGGGTCTCGGTGTCATCGCCGGTATCGGTTTCGACATCACGCCCAACCTGACCCTGGACGCCGGCTACCGCTACCACAACTGGGGACGCTTGGAAAACACCCGCTTCAAAA

>12 |ref|NC_017511.1| Neisseria gonorrhoeae TCDC-NG08107 | Coordinates: 64847,64993 | Forward

TCGGCTCCTTATTCGGTTTAACCGGTTAAAAAAAGATTTTCACTGATGTTGAAGGGCGGATTATATCGGGTTCCGGGCGGTGTTTCAACACAATATGGCGGATGAACAAAAACCGGTACGGGTTGCCCCGCCCCGGCTCAAAGGGAA

>14 |ref|NC_017511.1| Neisseria gonorrhoeae TCDC-NG08107 | Coordinates: 65111,68003 | Forward

CCGATAGATTCCCGCCGCGTCGGGGGTCCGGATTCCCGCCTGCGCGGGAATGACGGGTTTCGAGATTGCGGTGTTGTCGGGAATGATGGAAAATGGCGGGAATTGTGTAAAAAATGCCGTCTGAAACCGTTGGAAGCATCGTAAACGTTGGAGTCGATGAATCGGTGGGCTTCAGTCCGCCATTCCCATCAACCCAACATGTCTACCGTTTTCATCGAATCCATCGAATCCGCCCTTTCGACCACCCGGCCCTACGCAACCGAACCGTCATTCCTCGTGGGAATGACGGGATGTAGGTTCGTAGGAATGACGTGGTGCAGGTTTCCGTGCGGATGGATTCGTCATTCCCGCGCAGGCGGGAATCCGGTCCGTTCGGTTTCAGTTATTTCCGATAAATTCCTGCTGCTTTTTATTTCTAGATTCCCACTTCCGTGGGAATGACGGCGGAGGGGATAAGTTCTTGCAATCTAAAATTTCGTCATTTCTATAAAATAGCAAACCGAAACAGAAACTTAAAAACAGAAACCTGAAACAGCAACCTGAAACCCCGTCATTCCCGCGCAGGCGGAAATCCGGCGGGCCGTAGGGTGTGCTTCGGCCCGCCATTTCCATCAATCCAACATGTCTACCGTTTTCATCGAATCCATCGAATCCGCCTTTTCGACCACCCGGCCCTACGCAACCGAACCGTCATTCCGGAATCTAGGACGCGGGGTTTGGGCAACCGTTTTATCCGATAAGTTTCCGTGCGGACAGGTCCGGATTCCCGCCTGCGCGGGAATGACGGGTTTCAAGATTACGGCATTTGCCGTTTCGGGTACAGGAAAGGGGGTTTTCGGGTAGAATGGCACTCTTTTATCCGGCTGTTGAAAAATATGTCTTCATCTGTTTCAAGTAAAACGCGCTATTGGGTATTGGCACTTGCCGCCATCGTGCTGGACCAGTGGTCGAAGTGGGCGGTGCTGTCGTCGTTTCAGTATCGGGAACGCGTCAACGTCATCCCTTCGTTTTTCGATCTGACGCTGGTGTACAACCCGGGCGCGGCATTCAGCTTCCTTGCCGATCAGGGCGGCTGGCAGAAATACTTTTTTTTGGTGCTGGCGGTGGCGGTGAGCGCGTATTTGGTACGCGCCATCTTGCGCGACGAGTTTGCAGCCCTCGGCAAAATCGGGGCGGCAATGATTATCGGCGGTGCGTCGGGCAATGTCATCGATCGCCTGATACACGGTCATGTCGTCGATTTCTTATTGTTTTATTGGCAAAATTGGTTTTATCCCGCCTTTAATATTGCCGACAGCTTTATCTGCGTCGGTGCGGTGTTGGCGGTGCTTGACAATATCGTCCATCGCAAAGATAGCAAAAAAACGTGAATGCCGTCTGAACACGGAATGCAAAACTTATGAACGGAAAAACCATCATCCTTGCCAATCCGCGCGGCTTCTGCGCCGGTGTGGATCGGGCAATCAGTATTGTCGAACGTGCTTTGGAAGAATTCGGCGCGCCGGTTTATGTGCGCCACGAAGTCGTCCACAACAAATTCGTCGTGGACAACCTGCGCGAAAAAGGCGCGGTGTTTATCGAAGACTTGGCGGAAGTGCCGCCGGGCGCGACACTGGTTTATTCGGCACACGGCGTATCGAAGGCGGTGCAGCAGGAAGCGGCGGAGCGCGGTTTCCGGGTATTTGATGCGACTTGCCCGCTGGTGACGAAAGTGCATAAGGAAGTCGCCCGACTGGATGCCCAAAACTGTGAAATCATCATGATCGGGCATAAGGGGCACGCCGAGGTCGAAGGCACGATGGGGCAGCTTGCACCGGGCAAAATGCTTTTGGTCGAAACGGTCGGAGATGTGGCAAAACTCGAAGTCAGAAACCCCGACAAACTCGCCTATGTCAGCCAAACCACGCTCTCGGTCGATGAAACCAAAGACATCATCGCCGCGCTGAACGCGCGTTTCCCCAATATCCGCAATCCGCACAAGGAAGACATCTGCTATGCGACGACCAACCGGCAAACCGCCGTCAAAGAGCTGGCGGAACAGTGCGACATCGTGATTGTGGTCGGTTCGCCCAATTCGTCCAACAGCAACCGCCTGCGCGAAGTGGCGGCATCGCGCGGAATCGATGCGTATATGGTGGACAACGCGTCCTACCTGCAACGCACGTGGTTTGAAGGCAAAAGCAAAGTCGGCGTAACGGCAGGCGCGTCCGCGCCCGAAGTGTTGGTGCGGGAAGTATTGGCAGCCATACGCGGATGGGGGCATGAAACCGTGCGCGAAGGGGGGGGCGCGGAAGAAAGCATCGTGTTCGTCCTGCCCAAGGAGCTGCGCCGCGAGGGCGAAACCAAACCCGATTTGTGCAAACGTTGACGCAGGCGTTAACGCAGGCATCGGATGTTTGGGCAACACAAATGCCGAGACCTTTGCAAAATTCCCCTAAAATCCCCTAAATTCCCACCAAGGCATTTAGGGGATTTCCCATGAGCACCTTCTTCCGGCAAACCGCCAAGCCATGACTGCCAAACACATCGGCCGCTTCCCGCTATCGGAGTTGGACCAGGTGATTGATTGGCAGCCGATCGAACAATACCTGATCCGTCAAAAAACCCGTTACCTCCGAGACCGCCGCGGCCGTCCCGCCCATCCCCTGTCGTCCATGTTCAAAGCCGTCCTGCCCGGACAATGGCACAGCCTCTCCGATCCCGAACTCGAACACAGCCTCATCACCCGCATCGGTTTCAACCTGTTTTGCCGTTTTGACGAACCGGGCATCCCCGGTTGCAGCACCTTATGCCGCTACCGTAAATTCCGCTATGCGCGGGCAGCCTATTTCGGGCTGCTCAAAGTGGGTGCGCAAAGCCACCTGAAGGCGATGTGTTTGAACCTGTTGAAAGC

>15 |ref|NC_017511.1| Neisseria gonorrhoeae TCDC-NG08107 | Coordinates: 68004,82794 | Forward

GTGTTTGGGTTTCGGATGTCGAAGGAAGGGCTTTTTTGCAAAGGCCTCATGCCGTCTGAACAGGCTTCAGACGGCATTTTTGCCGCGTGCCGGATGCGGAAACCAATCAGGCGTAATGTCGTGCAAGAAAACCGGGCAGTTCGGACAAACCGTCCAATACGGCGAGATGCGGTGCGCCAAGGAGCTGTTCGCGCGAATGTGCGCCGGTGGCAACGCCGACTGCCGCCGCGCCTGCGTTTGCCGCCATATGCAGGTCGTGCGCCGTATCGCCGACAACCAATGCCTCTTTCGGGTCGAGTCCCAGTTCGCCGCAGAGTCCGAATACCATTTCGGGCGAGGGTTTGGAGGGGTATTCCCCCGCGCAGGCGGTGGCCAGCCAATAGCCGCCGGTGGCGGTTTGGCTGATGGCGTTGTCCAAACCCGCCCGCCCTTTGCCCGTGGCGACGGCAAGCCGGAAGCCTTGCGCTTTGAGCTTGTCCAGACAGGGCAGGGCATCGGGAAAGAGTGTCATATTGCGGTTGTTGGGATTGAGGTAATGCGCGGAATAAGTGCGCGCGATGTCGGCAACGGCAGCTTCAGAAGGCATTTCGAGCAGGGCGCGGATGATTTCGGGCAGGCTGTATCCGATCAGGCTGCGGACGCGCTCCGCTTCGGGCGGCGGAAAACCGCATTCGGCGAAGCTGCGGCGCATGGTGTCGATGATGGGCTGGGTCGTATCGGCAAGCGTGCCGTCCCAGTCGAAGATGATGAGTTTGGGCGGGGTCATGGCAGGTTGGTTGCAGTAAAAAAGCAGATTTTATGCGGAAAACGCAGACGTGTCGCATTTTCGACAAAATTTGTCGGCTGAGCGATATGTTTTTCCGAACAAGCCGCGTTGTGCTTTATTAAAATAGAACCATTATCATTTATACAGATGGGACAGTTTATGTCAGTTTTCCGCATCAATATGGCCGCCGCCACGGTTTTGGCAGCACTCTCGTCTCCGGTTTTTGCCGCACAAACGGCGGATTTGGAAACCGTCCATATCAAAGGGCAGCGTTCGTACAACGCGATTGCCACCGAGAAAAACGGCGATTACAGCTCGTTAAAGGCAGCTATATGGATGACCGCCTCAATACCCGCGTCTCCCCCTGCCGCCTGAAAGACAAAAACGCCGCCGAACCCGAACAACCGCAACACCCGTTACGCCGCATTGGGCAAACGCGTGATGGAAGGCGTTGAGACCGAAATCAGCGGTGCGATTACACCGAAATGGCAAATCCATGCAGGTTACAGCTATCTGCACAGCCAAATCAAAACCGCCGCCAATCCACGCGACGACGGCATCTTCCTGCTGGTGCCCAAACACAGCGCAAACCTGTGGACGACTTACCAAGTTACGCCCGGGCTGACCGTCGGCGGCGGCGTGAACGCGATGAGCGGCATTACTTCATCTGCAGGGATGCATGCAGGCGGTTATGCCACGTTCGATGCGATGGCGGCATACCGCTTCACGCCCAAGCTGAAGCTGCAAATCAATGCCGACAACATCTTCAACCGCCATTACTACGCCCGCGTCGGCGGCACGAACACCTTTAACATTCCCGGTTCGGAGCGCAGCCTGACGGCAAACCTGCGTTACAGTTTTTAAAGACCAATATGCCGTCTGAAACGGCAGCCGCAGCATAATCAAACCACAACAAGCTGCGCGGCATACCCTATGCGCTCACAACCGGAGTATGGCATTGCGAAGGAAAACAGACCGAACCGGCAGGCAGACCGCTTTGCCGGTTCGGTTTTACCGCTTGCCGCCAGTCTGACCCACAAGCCGAACATCATGAAACCCATACCGACCGACACATTCCAACCTGCCATACTGCCCCAAGCCTTTGAAACCGAAATCAAATCCACCTGCACGGGGCGAATCTATCGGATTCAGACGGCAACACTCGGCGAAATGCAGTCTGAAGGCTATCCCGTCCTCTTTGTCCTCGACGGCGAAGCCTTTTTCCCGCGCTGTACAACATCATGCAGTCGCTGATGAACAACCCCGTTACCCGAAGCAACGCCCCCTGCCTGATTGTCGGTATCGGCTACACGACAGGCAGTGTGCGCGACCTGGCGCAGCGTGCCGCCGACTACACGCCGCCGCTTGGAGACAACGCCACAGCAGACGAACGGCGGCAGTTCGGACAGGCAGACCGCTTCGCCGACTTTATCGACAGCGAACTGACCGCCTTTTTAGAAAGCCGCTACACCCTTAACCGTAATGAAACCGCCGTATTCGGACACTCGTTCGGCGCACTGTTCGGACTGTATTCCCTGCTTTCCCACCGCTGTTTCAGACGGCATTGGCTCGTATCCCCCTCGATTTGGTGGCACAACAGGCGGATACTCGACTTTATGCCGTCTGAAAACCGGCTGGACGGCATCGATGCCTGCCTCAACATCGGCGCACTCGAGCGGAGCAGCGGTTGCAAACGCAGGGAAGAACGCGACATGGCAGGGCAGGCCGAACAAATGGCGGCAGAGTTGGACAGGCGCGGGACCGCCGTATTTTTCCGGGAATATCCGAATGCCGACCACGGCAATGTCCCGTTTTACTCGCTGACCGACTGCGTCGAATATTTGAGGGAAGCGTGGCAACGGTAGGGGGAATCAAATATATGACTGCTTTGTTTTGCATCGGAAAATATAGCGGATTAACAAAAACCGGTACGGCGTTGCCCCGCCTTAGCTCAAAGAGAACGATTCTCTAAGGTGCTGAAGAGTGAATCGGTTCCGTACTATTTGTACTGTCTGCGGCTTCGTCGCCTTGTCCTGATTTTCGTTAATCCGCTATAACAAGAGCTACCTAAGGGTTATTGCTCCCGTTCTCATTTCGCAGTGCTACAATTTTTGATAGTTCGCGCCGACTTTGCCCGTGGTTGTCGCTCCCGTTCTCATTTTATCTTGATATAAAAATCCCTGTTTCAGGCCGTCTGAAACAGGGATAGATTAATTTAACGGACGGGCGGGCGTTTTTTCAGGCGGCACGGTCTGATTTCTTTGCCCGGTTCGTCGATTTGGTATTTTTGGAATGAAAGGGCGGTTTTGACACCGATACTTTTGAAAATGCCGTTTTTCCCTTTGGCGCTGTCTGTGTCATGTACACGGATATCAATCGCCCCGGTTGATTTATTTAGGCTGGCGAAATAACCAAAAAATACGTTTTTCTTGGCAGTTACTTTTATTCAATCATTCGAATAAAGTACGAATGAAACCGGTTTTGGGCTTCAGACGGCATTTGTATTTTTGGTTACCAATTCACGCGCACCGACCGGCTGCCGAGCAATGTTTCCAGTTCGCCGAACAATGCGGAACTCGGTGTAACCGTCCATTTCGGAGGCACTTGAAGCCTGCCCGACGCTTTTTCGTTGGCATACGACAATTGAAGCGGGATGCGCGGCGTGTCGGGCAGTTGGTGGGCGGCGAGCAGCCGTACCAGTCCGCCGATGTCGTGATGCGGGGCAAGGGCGAGGCTGAGGCTGCGGGCGTAGCGTTCGCGCGCCGTTTGCAGGGTCATGACTTGGTTTGCCATGATGCGCAGCCCGTCGCCGCCGCCGTAATCGTCGCGGCTGACTTTGGATTCGATAATCAGCACTTGGTCGGCTTTGAGGCAGTCGGCGCAGTTTTCCAGCGTTTGTCCGCCGACCATGATTTCAGCCTGCCCGCTCGAATCTTCGAGGCTGACGAAGGCGATTTTGCCGCGTTTGCCCATCATGGTGCGCACGGCGGTAACGAATCCGGCGAGGCGCACGCTGTCTTGCGGTTTCAGACGGCCTAATTTGGTCGGGGCGATTTGGCGGACTTCTTGGGCATACGGGCCGAACGGGTGGCCGGACAGGTAAAAGCCGATGACGGTTTTTTCTTCGGCGAGTTTTTCCGATTCGCTCCACATGGGTGCGTCGATGAGCCGCACCGGTTCGATGGCGTCTTCCATCATGTCAAACAGCCCGCCCTGATTGGCGTTGGCGGCTTTTTGGTCGGCGTTGTCCATGGCAAGGTCGATGTTCGCCAAGAGCATGGCGCGGTTGGGTTCGATGCTGTCGAACGCGCCGCCGCGTATCAGGGCTTCGAGGGTGCGGCGGTTCATGTGTTCTTTGCCGACGCGCTCGCAGAAGTCCAACAGGCCGGTAAATTTGCCGCCGCTTTGCCGCGCGGCGATGATGGATTCGACGGCGGCTTCGCCCGTGCCTTTAATCGCGCCGAGTGCGTAGCGGATTTTCATGTTCGGATACGGCGTGAAGCGGTAGTCGGATTCGTTAATGTCGGGCGGCAGGAACTCGATGCCGTTGGCGCGGCAGTCGTCGTAGAAATGTTTGAGCTGGTCGGTGTTGTCCAATTCGGACGACATCGTCGCCGCCATAAATTCGGCGGGATAGTGGGCTTTGAGCCATGCGGTCTGGTAGGAAATCAGGGCGTAGGCGGCGGCGTGGGATTTGTTGAAACCGTAGCCGGCGAATTTTTCCATGTAGTTGAAGATTTCGTCGGATTTTTCGCGCGAAATGCCTTGTTTTGCCGCACCTTCGGCGAAGATTTCGCGGTGTTTCACCATCTCTTCGGGCTTTTTCTTACCCATGGCGCGGCGCAGCAGGTCCGCGCCGCCGAGCGAGTAGCCGCCGATAATCTGCGCCGCCTGCATCACTTGTTCCTGATACACCATAATGCCGTAGGTCGGCGCGAGGATGCCTTCCAGCAGCGGGTGGATGTATTGGAATTCCTGTCCCTTCATGCGTGCGACGAAGTCGGGAATGTTGTCCATCGGGCCGGGTCGGTAGAGCGATACGAAGGCGATGAGTTCTTCAAACTTGGTCGTATGCGCCGTTTTCAGCATTTTTTTCATGCCGGTCGACTCGAACTGGAAGACGGCGGTGGTGTTCGCATCGCGGAAGATTTGGTAGGCAGTCTGGTCGTCAAGCGGAATCTTGCCGACATCGACGATATCGCCGGCGGTGTTTTTGATGTTGTTCTGCGCCATTTCGATAATGGTCAGGTTGCGCAGGCCCAAAAAGTCGAACTTCACCAAACCCACGTCTTCCACGTCGCCCTTGTCGTACATGGATACGGGCGAGGCGGATTCGTCCGCCTGATACACGGGGCTGTAATCGGAAATCTTGCCCGGCGCAATCAACACGCCGCCTGCGTGCATACCCAAACCGCGCGTCAGGTCTTCCAGTTTTTTCGCCAGCGTAATCAGTTCGTCCGCTTCTTCCGCTTCAAGCAATTCCTGAATCTGCGGCTGCGCCTTCATCGCGTCGTCCAAACTCAGGGGTTTGTTGGCTTCCAACGGAATCAGCTTGGACAGTTTGTCGCACAGCATAAACGGCAGTTCCAGCACACGCCCCACGTCGCGGATGACCGCTTTGGACGACATCGTGCCGAAAGTCACAATCTGGCTGACCGCCTGAGCGCCGTATTTTTCGCGCACGTATTCAATCACGCGGCCGCGGTTTGCCTGACAGAAGTCCACGTCAAAGTCGGGCATGGAAACGCGTTCGGGGTTTAGGAAACGCTCGAACAGCAGCGCGTATTTCAGCGGGTCGAGGTCGGTAATCTTCAGCGAATACGCCACCAGCGAACCCGCGCCCGAACCGCGTCCCGGCCCGACCGGACAGCCGTGCGTTTTCGCCCAGTTGATAAAGTCTTGTACGATAAGGAAATAGCCGGGGAATTTCATTTGGATGATGATGTTCAGCTCAAAGTCCAGCCGCTCCTGATATTCCGGCATTTTTGCCGCCCGTTCCGCCTCGTCGGGATAAAGCTGAACCATGCGTTCCTGCAAACCCTCGTTGGAAAGTTTGATGAGACAGTCATCGAGTGATAAACCGTCGGGCGTGGGGAAAAGGGGTAGGAAGTTTTTGCCCAATGTGATGTGCAAGTTGCAGCGTTTGGCGATTTCCACCGTGTTTTCCAAGGCTTCGGGCAAGTCGGCGAAACGCTCCAGCATCGTTTCCGGCGGAATGAAAAACTGGCTCGGCGTGAAATCGCGCGGACGTTTCTTGTCCGTCAGCACCCAGCCGCCCGCGATGCACACCCGCGCCTCGTGCGCGTTGAAATCGTCGCGGTTCATAAACTGTGTCGGATGCGTTGCTACCACCGGCAAACCCAGTTCCTCCGCCAGCTTCACGCTGCCGGAAACGCAAGCCTCCCATTCGGGGCGTTCGGGCAGGCGTTGCAGCTCTAAATAAAACGTATCGGGAAACCATGCCGCATATTTCAACGCCGCCGCACGCGCCGCGTCTTCATTGCCGTTCAACAGATTCACGCCCACTTCGCCGTAATGCGCGCCGCTCAAACAAATCAAGCCGCTGTTGTCGCCGTTTTCCAGCCATTCGGGATTGAGTTCCGCATGATGGATATTGCGGTCTTGGCCGACATAAGCCTCCGTCAAAAGCTCGCTCAAGCGCAGATAACCCGCATCGTTGCGGATAACCAGCATGGCGCGGAACGGCTTGTCGGGCGCATTCGGATTGCCTATCCACACGTCCGCCGCGCCGACGGGCTTAATCCCTGCGCCGCGGCAGGCTTTATAGAATTTCACCAAACCGAATTCGTTCATCAAATCGCTGATGCCCAAAGCAGGCAAACCGTATTCTTGCGCTTTGGCAATCAGTTTTTTAATCCGCACCATACCGTCGGTAATGGAGAATTCGGTATGCAGGCGCAGGGGGATGTAGGTCGGCTCGGTCATGGCAAAATCGGCGTGGACAATAAAAGGCGTATTGTAGCAGGGTTGTCTTTAGATGGCGGTGCAGGTAATGCCGTTTCGGATTCAGACGGCATGACCTGCAAATGTTTTTTGAGCTTTTACGACGGCAAAAAAATGCCTCCTGCCGTGTGGCGGAGGCTTCCCAAGGAGTATTGATAGATATAAAGGACTATCAAACTAGTTATAAGGAACTATATACCTTATTCGGACGGACGGCAAGCAGTTAAATAGATTTTACGTTCAAACAGGTTTTTGATTTCGTTTTGATGCCGATTGCCGGTGTATCGGGCAGTCCGCGTTTGAGGATGTGCATCAGCGTCAATGCGGATTCGTCGGGGAACAGGATTTGCAGCCTGCCGTTGGGCAGGATTTCTTTGGACGGATGGCGGCATACGGGGTTTCAGACGGCATTTTTACGGAACAGGCAGTGCGGACACATCGCCGGTTTTGCGGCAATTTTGGGTGCGGCGGCGGCAGGTGCTACAATAACGCCCTCTTTCTAAAAGGGGACATTATGGAAGCCACCGTCTATCTCGAAGACAACGAATACATCGCCTTGTGCGACCTCTTGAAATTGGCCGGACTTGCCGAAAGCGGCGGACAGGCAAAAGCATTTATCGCCGAAGGGCTGGTGTTGCGCAACGGCGGAACCGAAATCCGCAAAACGGCCAAAATACGCGGCGGAGAAGCCATCGAGTTTGACGGCGCGCGCTTGGAAATCGCCGATGGATACGACCCTGAAGCATAAAGCCGAAGCCCTTTTGGGCGAGCCGCTTTTGGACGAACCCGTCCGCCCCGAGTCGTGGGAATGCTGCGGCAGCGACTGCGGCGAGGCGTGCATTCAGACGATTTACTGGGCGGACAAGGCACGCTACGATGCGCAACGGAAAAAACTGAAGGAAGCGGGTTGGCCGGACGATGCCGTCTGAAAACGGTTGGGCTTGATAAAACCACGTTTTCAGACGGTCTTTTATAGTAGATTAAAATTGAAAACGTTCATATCGCCATTCCCTCGAAAGCAGGAATCCGGAAGTTTGAAATAGCGGTTAACCTTAAACATTTCCAATAAATCAAAGTCTGGATTCTCGCCTGCGCGGGAATGACGGCATAGCGGCTTTTCCTTTGCATTTGCCATACACCTGTTTTTTAAAATGACCGCCGCCGCCACACTGTCCGACAACATTGAAACATCCCGTCCTTCATTCACCACCACATAACACCCCATGATAGACCTGCACTGCCATTCCACCGTTTCCGACGGTATGCTCCCCCCCGCCGAAGTCGTCCGCCTCGCACATCAAAACGGCTGCACGCTGCTGGCGTTGACCGACCACGACCACACGGGCGGCATCTCCGAAGCGCGTGCCGAAGCCGACAGGCTCGGTTTGCGCCTGATTAACGGCGTGGAAATTTCGGTAACGTGGCGCGGGCGTACCATACACGTTGTCGGTTTGGATTTCGACGAGCAGGACGAAAACCTGCAAAACCTGTTGGCAGATGTCCGCAAAGGCCGTCTGAAACGTCTTGAAGCCATCGCCGCCAAGCTCGAAAAGAAAGGCATCGGCGGCGCATACGACGGCGCGCTGGCGCTGGCGGCAAACAAAGAAATGGTCAGCCGCACCCACGTCGCCGAGTTCCTCATCAAAAACGGACACGTCAAAAACAAGCAGCAGGCGTTCACCAAATACTTGGGCGACGGCAAATCCTGCGCGGTACGCCACGAATGGGCGACGCTGGCAGACTGCGTCTCCGCCGTCAACGGCGCGGGCGGCATGGCGGTGATTGCCCATCCGATGCGTTATGATTTGTCGGCAACCGCCAAGCGCAACCTGTTTGAAGAATTTAAAAACCTCGGCGGCGCGGGCATCGAAGTCCACAGCGGCAACTGCTGCAAAAACGACCGCCTCAACTACGCGCTTTTGGCAGACCGCTTCGGATTGTTGGCAAGCGCGGGCAGCGACTTCCACCGTTTAGACGACTTCAGCGGCGGCATCCTCGGCGCGTGTCCCGATCTTCCGGAAAACTGCAAACCGGTGCGGGCGCATTTTTCCCGACATTGAATGCGGATGAAAATGCCGTCTGAAAGGTCTTCAGACGGCATTTTTTGCGTTTTAAACGTTGTCGTACGGTTTTCGGACGGTTTTGCCGATGGCGGCGATGCCTTTTTCCAGCGTTTGAGCGTCCTGTGCGATACTCATACGGATGCACTCGCCCGCGTGCGGGTAGTCTTGCGTGTCGATGCCGACGAAGAAATGTTCGCCCGGAATAATCAGCGTACCTTCGGCTTTGAGCATTTCGTACAGGGTTTGCGACGAAACGGGCAGGTTTTCAAACCAGAGCCACAGGAAAATCGCGCCTTCGGGTTTGTGGATTTTCATCGGGTACGCGCCCAGCTCGCGCTTGAGCAGCGAAACGGCGGTTTGCGCCTGATTGCGGTAAAACGGCCGGATGACTTGGTCGGCAAGCCGTTTCATCTCGCCGCTTTCCAGCAGCGGGGCGGCGATGGCCGCGCCGAAGCGCGTGGGGGCAAGGTTCACAATCGCGTTCAGGCTGCTGACGGCTTTGACGGCTTCGGGCGCGGCGACGATGATGCCGGTGCGAACGCCCGGCAGGCCGACTTTGGAAAGGCTGAAGCAGAGGATGATGTTTTCGTGCCAATTCAGCGTTACGCCGCTGTCAATAATGTTGGGGAACGGCATTCCGTAGGCGTTGTCGATAATCGGCGGAATGCCGTGTTCACGCGCCAAAGCGTCCAAACGCGCCATTTCGCCGTCGGTCAACACGTTGCCGGTCGGGTTGGTCGGGCGCGAACAGCAAATCGCGCCGATTTTGCCCGCTTTGAGTTCGGGCAGGTTTTCCAGTGCGTCAAAGTCCACGCGGTATTTGAAGAAGCCTGCTTCGCCTTCGTGTTCGACGTTTTCGATTTTGGGTTTGACGGAAACGAAGTGCCGCCCTTCGACATGCACGTCGGCATAGCCGATGTATTCGGGCGCGAGCGGCAACAAAATGGCTTTTTCTGCGGATGTGCCGTCTGAAAGCTTGAATTTGCCGCCGAAGAGGTTGAATAAATAGAAAAACGCGTTTTGCGAACCGTTGGTCAGCGCGATATTGCCGACGGTCAGGTTCCAGCCGTATTCGCGGTTGGGGAAGGCGGTCAGCGCGTCAATCAGCGCGGCATCGCCTTGGGGATTGGAGTAGTTGCCGATGTTCTCGACGGCGTGTTCCGCCGCCAGCTTGGAAAATATGTCGGCGAACGCCCGATCGATTTCGGGAATGCGCGCCGGGTTGCCGCCGCCGAGCATATTGACGGGCTTGTCGCTTTTGAGCGCGTCGCCGAGGTCGTCCATCAGTTGCAGGATGCCGCTGTGTTGCGTGAATTTTTCGCCGAATGCCGAGAACTGCATGTCAAACTCAGTGTGTGTAAGGCAGATTGGTTTGTACGGTATGCCGTCTGAAGGTTCAGACGGCATTTTTTCTGTGTGTTTCGCATACCCAAAGCAGGTCGCAGATGCCGCCGGTCGGGGTAAAGCCGGTCGGGGCGTTGACGAGCAGGTCGCGGATTGCCTGCTGGTCGTAACGGTCGCAGGCGGTGCGGATGCGGTCGAGCAGGGCGGAGAGTTCGTGCCACGGCAGCATGGTCTCGTCGGCGGTCATGATGCGCGGATGGCCGGTTTTGCGGACGTTGTCGCCGATGAGCAACTCTTCGTAGAGTTTTTCGCCGGGGCGCAGCCCGGTAATGAGGATTTCGATGTCGCCGTCGGGCTGTTCGGGCGTTTTGGGTTTGAGTCCGCTTAAGGTAATCATTTGGCGGGCAAGGTCGGTGATTCTGACGGATTCGCCCATATCGAGGACGAACACGTCGCCGCCTCTGCCCATCGCGCCGGCTTGGATAACCAGTTGCGCCGCTTCGGGTATGGTCATGAAATAGCGCGTGATGTCGGGGTGGGTCAGGGTGATGGGGCCGCCTTCGGCAATCTGTTTTTCAAACAGCGGGACGACGGAGCCGGACGAGCCTAAAACATTGCCGAAACGTACCATGCTGAAGCGGGTTTTTTGTCCGGGTTCGGCGGCGAGTGCCTGAAGGCAGAGTTCCGCCATGCGTTTGCCCGCACCCATCGTGTTGGCGGGGCGGACGGCTTTGTCGGTGGAGATGAGGACGAAGGTTTCCACGCCCGAAGCCGTGGCGGCAAGCGCGCATTCGAGTGTGCCGAAGATGTTGTTGCGTATGCCCTCGACGGTGTTGAACTCGACCATAGGGACGTGTTTGTAGGCGGCGGCGTGATAGACGGTCGCAACGGAAAAGGCGGTCATAATGTGCGTGAGCAGCGTGCGGTTTTGCACCGAACCCAAAAAGGGAAGGATTTCGGCGGCGATGCCGTTTCGGGCGCAGTATTCGCACAATTCTTTTTCAACGGTGTACAGGGCGAATTCGGACAGCTCGAACAGCAGCAGCCTTTCGGGGCGGCGGCGGATAATCTGGCGGCAGAGTTCCGAACCGATGGAGCCGCCCGCGCCGGTTACCATGACGGTTTTGCCTTCGGTGTCGGCATTCATGAGGCGGTCGTCGGGTGCGACGGAATCGCGCCCGAGCAGGTCGGACACAGAGATTTTTTTGAGCGTGCCGATGCTGATTTTCCCGTCCATCAGGTCTTTCATTCCGGGAATGGTCAACACTTCGCACGGATAGGCTTTCAGTTTGCTGATGATTCGGCGGCGTTGTTCCTGAGTCGCGCTGGGAATGGCGAGCAGGATTTTTTCCACGCCGTAGCGTTCGATGAGGAAGGCGATGGCATCGGGCTGGTAAACGGCAAGGTCGTAGATGACGGTGTGCCAAAGCTTGGGGTTGTCGTCCACAAAGGCGGCGGCGGAATATTCGCGCATCTGTTTGACGGCTTCAAGGAGCTGCCGACCCGAACGCCCCGCGCCGTAAATGATGACGGGGTCATGTGTTTTTTTCTGGTGGTCGGACAGGAGTCCGCGCAAAACCATACGCGAGCCGGTCACGGAAACAAACAGCAGTAAGAAATAGACAATCGGCAGGGCGAGGCGCAGCCTTTCTTCAAAAATCAATGTGTTGAGGAAGAACAATACGGCGGAGGCGAGGCTGCCGGCAAAGGCGGTGGTCAGGACGTGGAAACTGACAAAGCGTGTAACGGCGCGGTAAAGCCCCATTCGGATAAATAATGTGATGGTCAGCAAGGCAGTCAGCAAAAAAGACTGCCAGTTGGCAAAATCGAACCATTCGTCCGAGTAGTCGGCCTTCAGGCTTTGGGTGAACCAAAAGGCAATGAAAATCATCAGAAAATCGTGTATGAGGAAACAGATTTTCTTGATGTTGCGCGGCAGGGCGATCAGGGTTTCCAGATTCATATCGTGGGGCGGTATGTGCTTTCAGGCGGCATATGCCGTCTGAAGGGTTATCGTGCGGCTTCGGTCAGGACGGCTTCGATGTGTTTTTTGCAGAACGCGATTTCGTCGTCGGTCAGCGTCGGGTGCACCAAGAACGTCAGGGCGGTGCCGCCCAGTTCGACGGCGTTTTTCAGCCGCTCTTTGGGCCGCCACGGCGTGTTGTCGAAGGCTTTTTCCAAATACACTTCGGAGCAGCCGCCTTGATAGCAGGGGACGTTGCGCGCGTTCAGTTCGCTGACGATGCGGTCGCGCGTCCAGTCGTCTTTCAGGTGTTCGGGTTTGACGAAGGCGTAGAACTTGTATTGCGCGTGTCCGATGTAGCCGGCGACTTCAATCAGGCGGATGCTTTTGAATTTGCGCAAACTTTCTGCCAGCTTGGCGGCGTTTTCTTGGCGGCGCGCCGTCCATTCGGGCAGGTGTTTGAGCTGGATGCGGCCGATGACAGCCTGCATTTCCATCATGCGCCAGTTGGTGCCGAAACTTTCGTGCAGCCAGCGGAAACCGGGCGCGTGTTCGCGGTGGTACACGGCATCGTAGCTTTTGCCGTGGTCTTTGTACGCCCACATTTTTTCCCACAGGGTTTTGTCGTTGGTCGTAACCATACCGCCCTCGCCGCCGGTGGTGATGATTTTGTCTTGACAGAACGACCACGCGCCGACGTGCCCGATAGAGCCGACGGATTTGCCTTTGTAGGTCGCGCCGTGCGCTTGGGCGCAGTCTTCAATCACCCACAAATCATGTTCTTTTGCCAAAGCCATGATGCCGTCCATTTCGGCAGGCATACCGGCAAGGTGGACGACAATGACGGCTTTGGTATTCGGGGTCAGCACGGCTTTGACGGTTTCCGCGCTGATGTTTTGGCTGTTCAAATCCACGTCGGCAAACACGGGGTTTGCGCCCGCGTTCACAATGCAGGAGGCGGAGGCGAGGAAGGTGCGCGAGGTAACAATCACATCGTCGCCCGCGCCTATGCCGATTGCTTTGAGTGCGGCATCGAGCGCAAGCGTGCCGTTGGAAAGGGCGACGGCGTACCGCGTGCCGGCAAAGGCGGCAAATTCTTTTTCAAATTCGCGGCATTCGCTGCCCGTCCAGTAGTTGACTTTGTTGGACAGCAGGACTTTGGAAACGGCATCGGCTTCTTCTCGGGTGAAGCTCGGCCATGGGGAAAGGGCGGTGTTCAGCATGGTGTTTGGTCCGTCGGGTTCGGACGGCATTTCCGACCCTATGCCGTCTGAAGGGGGCGTGTTCCGAAGAATCGGGCGCGCGCCGCAGGTGTTGTCAAAATCGGTTTGTACGGAAGTTTATTTTAATCGCTTATGCCGTCCCGGTCTTGGGGTTTTTGCCCGTAAGGGGCTTTGCCGGGTTGCCCGCGACGGTCATGCCGTCCGGGATGTCGCATACGATAACCGCCCCTGCACCGGCGGTAACCCCGCTGCCGACGGTTGTCTGCTGGCGGCTGCACGCGCCCGTGCCTATCCGGCTTTCTTCGCCGATACGCGTGTTGCCTGACAGGTGCGCGCCCGGGCTGATGTGGACGAAAGCGTCAAGCAGGCAGTCGTGATCGACGGTGGCGGCAGTGTTCACAATCACGCCGTCTTTCAATACGCTGCCGGCCTGTACGACGGCTTTCGCCATTACGACGCTGCCTTGTCCGATTATTGCAGAAGGCGAGACGGTCGCGTCGGGATGAATCAGAACGGGCAGTTTGAAGCCGAGCGCGGCGGCTTTTTCGGTGATTTGGCGGCGGATGCGGTTGTTGCCGACGGCGACGGTGATGTCGAATTGTTCGGGCGATAAACTGTTTTCAAGCAGCAGCGTCGTGCCGATGACGGGGAAGCCGTTGACGCTGCCTTGGGTGCGGTCGTCCAGAAAAACGATTTCGCCGTATGTGCCGAGTGCGGCGGCAAGCTCGGCAACGACTTTGCCGTGTCCGCCCGCGCCGATAACGGCGAGTTTGCGATTCCCCGCGAAAGGGGGCATGGTGGCTTCCCCTTGCGCCGAAATGCCTTCTTTAATCAAGACTTTTTTGACTGTCAGAAACAGGATTTTCATATCCAGCCAAAAGCTGAAATTGTCGGTGTACCAAACATCGCAGGAGAACTTTTCGTCCCACGAAAGCGCGTTGCGCCCGTTGACCTGCGCCCAGCCGGTAATGCCCGGTTTCATTTCGTGGCGGCGGTTTTGAAATTTGTTGTAAAGCGGCAGATACTGCATCAAAAGCGGGCGGGGGCCGACCAGGCTCATCTCGCCTTTGAGGACGTTCCACAGTTCAGGCAGTTCGTCCAAACTGGCGGTGCGCAGTTTTTTGCCGAAGGGCGTGAGCCGCTCGCTGTCGGGCAGCGGAATGCCGTCTGAATCAAGCGCGTCGCGCATGGAACGGAATTTGACCATTTTAAAAGGTTTTCCGTCCTTTCCGGGGCGTTCCCGAATGAAGAAGACGGGCGAACCTAAGTTTTTGCGGATGAGGTATATTAAAACCAAAAACACGGGCGACAGGGCAATCAGCCCCGATGCGGAGGCGACAATGTCGATGAGGCGTTTGAAAAATTTATTCATCAGCTAATCTTTCAATCAGGTTGACGATTTTCCGATAGGAAATGTCGCGCTTGAAGCGGCGGACGATTTCTTCGGACTGAACAGGATCGTTTTTGC

>16 |ref|NC_017511.1| Neisseria gonorrhoeae TCDC-NG08107 | Coordinates: 82795,89530 | Forward

GCTTCAAAATATCTTTGGCGGCTCGAACGAAACCGTCCACATCGCCGGAACGGTAGTTCGCATGCGGCAGCAGGGTAAGGACTTCGGCAACTTCGTCGTTGACCTGGCTGTTCAGAATCGGTTTTTGCAAAGCCATATAGTCGGAGAGTTTGTTGGTAATCGACTGCATGGCGTAAGAGTGGATGGCGTTGACGGCAATGTCGCAGCCCTTGGCGACCGACATCATTTCGGCGTAAGGAATGTAGCCGTAAAACTTGATGCCGTCGCAGGCATATTGTTTGAGCCTGTCCAAATCGGGGCCGCCGCCCATGATGTGCAGCTCTACATTTTCGCCGTCGTCCAAAAGTTTCCGAACGCCTTTGCACACGGTTTCCACGTCATAGCTGTAACTGAGCGTGCCCAAGTAGAAAAAGCGGGTTTTGTCGTCGCCAAAATCTTTGGCAGGTGCGGCATCGAGTTTGGGAAAGTCAGCACCAATATAGATGACTTCGCCGGGTACGTTCGGATTGGTTTCTTTGGCGCGGTCGAGATAAGTCTGCGATACGGCAACCAGCGCGTCGGCGTAGCGGTAGGCCCGGTTGGCGCGTGAAGCAAAGGGCAGCAGCTTGTGCGGTACTTTTTTCAAAAACGGTACGACCGAGGAGAAAGACTCCGGCCATACGTCCTGCACATCGACAATCAGTTTGTAGCCCAAACGCGCTTTGTGTTTGCCCAACAGCAGGTTGGTGGCAATCAGCGGATAGGCGGAATAGACGACGTCTTGTTCGCCCGGACGGCAGTTTTCCAACCATTTTTCAAAATGTTTGACGAAGCGGTGATGGCTGGTCACGCGTCCCAAAGATACGTTTTTGCTATATCCGCTTTCTTCCAACAGTATGACTTTCAGACGGCCTTGTGAGGCGGCCTCGGCATCTTCGGGCCGTCTGAAAGATTTGTCGTAGTGCTTGAAGTTGCTGGTAATCAGCAACACGTCGTGCGATTGCGACAATAGTTCTGCCAGATACCAAAAGCGGTTGAAATGCGGTTCGGACGGCAGCGAGCAGTAGGGGGCGGCTATGGTAATGTTCATGTTTCAGACGGCCTTACATTTCGTACGCAGCCATGGTGGTGCGGTAGATTTCGTCGTCGGAGCATAAGGTTTCGACATGTTTGTGCAGGGCTTTGCCCATTTGGCTGCGCAACTCTGGATGCTTGATGAGTGTATCGACGGCTTCGATAAACGCTTCGTCATCGCCGAAAGGAATGCAGTAGCCGGTTTGGCCGGTAATGACCATTTCGGAAATGCCCGCCATGTTGTAGGTCACGACAGGCGTGTCGTAAAGGCCGGCTTCCAAAATATTGTTGCCGACACCTGCGCCGTGGTCACCTACGCAATGCGGCGTGTTCACTAAAATATCGACTTCTTTGAAGTAGCCGGTCAAATCGCGGACGCCGCCGAGGAAGGTCACTTTGTCTTCAATACCCAAACGTTTGGCTTGGGCTTTGAGGTTGTCCATTTCTTCTCCTATGCCCGCCATGTTCAGGCGTACGGGTATATTGCGGCCAACCATTTTCTTCAAAATATCCAACATCAGATGCACGGCGCGGACGGTGTCCAAACGGGAAAGTGTGCCGAGTACGGCGCATTCTTTTTCGGTTTTCCGGAAATGAAATTCCGGGGGGTGTTGTAGGCGTAGGCAATCCGGCCGGCGGGGAAGCCGTGGCGGATGAGCTTTTCGCGTTCGTGTTTGCAGTTGCCGATGATGTACGCGCCCAGCTTGTCGAAGAGTTTGGCGGTTTTGGGGTAGGTTGCCGCGTCCAAACCGTGCGAGTGGCAGATGATTTTGGTTTTCGGTGAAACGATTTTGGCGGCAAGGGCGCAGGCCGGGACGACGCGCGCCATTTGGCAGTCGATGATGTCGGGTTGTTCGCGGCGCAGCATCCGGGCGTAGGCAAAGGTGCTTTTAAGGTAGCCGGCAAGCCCGCCCCGGTAAAAATCAACCGGCTGCCAACGGATGCCCGATGCTTGCGCCTCTCCGACAAAAGGGCCGTCCGAGGAGGCGAGGATGATGTCGTGCCCGTGCCGTTTGAGCAGCCGCCCGAGGCGGACGGTGGCGGTTTCTGTGCCGCCCAGACCCGACATAGAAGTAGTCAGGATGATTTTCATAATGGGAAACCTTGTTTTTTCAAATAATGAAACAGTTTGTGCAAATTTTTCCGGTGGCGCAGGATGCAGCCTGCCAGATATGCCGCCCATACGCCGGCAAACAGGGGGTAGTTTGCCGGCGTGCCGAAGCAGGTGTAAGCCGCCGAGGAGGCCAGGCAGAACAATGTGTGCATATAAAGCGGCAGGCGTTTGAGCGGCTGCCACAGGCGGCAGGAGCTTTCTGTCTTGAAAACAAAAAACAACCAGAATGAGGCGGCACAGGCAACCGCCGCGCCGCGCGTGCCGCCGGACGGTACGGCAAGCCCCAGCAGCAGCAGGTTTGCCGCCAGCGCGCCCAAGGTGGCAAGCGCGATCGGACGCGTTTTGCGGACGACGTTCAAACCGATGCCGCTGATTTCGGTCAGCGTGTAAAACAGCGGCGGCAACATGCACGATACGACGGTAAACCGGACGGCGGCGTAGTTTTCCGGCAGTAGGAGGGAGGCGAGGGGCGAGAAAATTCCGGTCAGGCAGAGGGCGGCGGCAAGCAGGGCGGCGGCAGATTCTGCCGTTGCCGAGAGGCGGGTGGGCGGGGCGTTTTTTTCGATTGCGCGGAAAATATACGGTGTCCAAACCGTTGAAAAGATGCTTTGGAACAATAATGCCGCCCCGCCGAACGAAATACCCATCGAATAAATGCCGAGCTGTTCCAGGCCGGCATATTTTTTCAGGAACAAACGGTCGGCGGATGCCAGCCCCCAATAGGCAAGGCTGCTCAGTGCGAGCGGTATGCCGTAGCGCAGCCCCCGGTGCAGGACGGCGGGCGAAAACGGCGCGCGCCGGACGGCCTTCAGACGGCATCGGTTTTGAAACAGCAAAAAGGCGGCGGCGGCAAGGTTTGCCAGCGCGTAAACGGCGGTCAGGACGGCGGTGTTCGCCGGAAAGTGCAGCAGCCCGACCGTCAGCGGCAACAGCAGCAGAATGGCGAGTTTGGGCACGAGTTGCGCGGACGAAAAGGCAAGGGCGCGCCCTTCCATACGCAAAACCAGTAAGAGAAAGCGGATGGGCAGGAAGCTCAGTTCAAACAGCACCAGCCCGATGCCGGCGGCGGCATCGTCGAGCGAAAACAGGATTTCAGACGGCAGGGACGGGCGGGAAAGCAGCAGGGCGGCTATCGCGGCGGAAAACAGCAGCGGCGGCAGGAACAGGGTTTTGAACAAAGTGTCTTTGTCGGCGGCGGCATAGTATTCGCGGACGTATGCCTGATCCAGCCCGAGGCACAATACCGACACCGTCAGTCCCGCCGCCGTCTGCATCAGCACGATGCGCCCGATGTCGTCGGCGGGGAAATACCACGACAGCAGCGGCAGGATGATGACGGCTAAAACCGCGCTGCCGATCGAGCCTGCCGCGTAGCCGAGGATTTCTTTTGTGTCCATTTTTGATGTCCGGTCGGCGGCGGGATGCTGCCTGTGCCGTCTGAAGCCTTTCTTGATCGGAATTTGACGGCTTTCAGGCCGTCGCGGCTGCCGGCGGGGTGCGGCAGCCCGGGTTGCGCTTTTCCGGGCGGGCGGCGGTCTGAACGGGCTGTTTTTTATCGGCGTTATTATATAGTGAAACGGCGGCAAACCCTTTAAAAGGCGTTGCCGTTTTTCCGGAACACGGTTTTGATGTCGTGTCCGAGGATTTCGGTGGAAACGGGTGTCCATAATGGCGGCGCGGAAAGGGCGGCGCGGTTTTCGGGCAGGGAAAACAGGTCTTTGCCGCCGCCGAGGATTTTGGGCGAACGGTACAGCACGATTTCGTCCGCCAAATTTTCCGCCAAAAATGCGGATGTGAGTTCGGAGCCTGCTTCGACCATGATTTCGCCGAAACCTTCGTCGGCAAGGAGGCGCATCAGATGGTGCAGGTCGATTTTGCCGTCTGCCGTTTCAGACGGCATCAGGATGCGGATGTGTGCGTGTTCCCGATAGGGGCGGAGTTTGTCTTCATCGCGTTCCAAGGTGGCGATGTAGGTCGGAGATTGTCCGTCGGTAACCAAATGGCTGTTCGGGGGCAGGCGCAGGCGGCTGTCTAAAACGATGCGTGCGGGTTGGCGCAAAGTTGGAAAAGCGCGGACGTTGAGCCGGGGATTGTCTGCCAACACCGTGCCGATGCCGGTCAGCACCGCGCAGCTTTCGGCACGCAAAACCTGTACGTCGGCACGCGCTTCCTCGCCGGTAATCCAAAAGCTGCTGCCGTCTGAAAGGGCGGTTTTGCCGTCCAGCGAAACGGCGCATTTGAGGCGGACAAAGGGGCGGCGGCGTTCGATGCGCGATAAAAATCCCCGGTTGAGTTCGCGTGCTTTGTTTTCAAGTAGTCCGCATTCCGTCTTGATGCCCGCTGCTTTGAGCAGGACAAGCCCTTTGCCTGCAACCGGCGGGTTGGGGTCGCGCATGGCGGCGACGACGCGTGTTACGCCGGAACGGAGCAGGGCTTCGGCGCAGGGCGGTGTGCGTCCGTAATGGCTGCACGGTTCGAGGGTAACAAAGGCGGTCGCGCCTTTTGCCATTTCCCCCGCCTGATGCAGGGCGTGGACTTCGGCATGGGGTTCGCCCGCTTTGACGTGGAAGCCCTGTCCGACAATTTGTCTGCCGTGTGCGATAACGCAGCCGACGCGCGGGTTGGGTGAAGTGGAAAAACGCCCCAAAGCGGCAAGTCGGAGGGCGTTTTCCATCATGGATATATTTGTGTCCGAAAACATAGGGATACCGTATCAGTATGGGTTGGGGGAATCAGGTTTTGCCGCCTGTTTTGACGGCTTGCGCCAGCCACGAGGCGAAATGTTCCGGGCTGTCGAAGCGTTTGTGCAGGGCGGCGAAACGGACGGCGGCTTCCGTGTTTTGCCCGAACAGCTCCTCCAGCACGATTTCGACAAGTTCGGATGAGGATATGTCGCGCTGACCCGAAGTGTAGAGCCTGTGTTCCGTCAGGCGGACGGTTTCGTCAATCTGTTCGGGTGTCAGGGCGGATTTTCGGGCGGCGGCGGTCAGGTCGTTGCGGAGGCGTTGTGCATTGAAGGGCGAACGTTTCTTGTCCGGACCGATGACGGCGGGCATTTTGAGTTCGACGGTTTCGAGCGTGCCGAAGCGTTTGCCGCAGCCGGGGCAGTGGCGGCGGCGGCGGACGGTATTGCGCTCTTCCATCAGGCGGGAATCGGCAACTCGGGTGTCTGGGTGGCTGCAAAACGGGCATTTCATGGTGTTTCTTCCTGATGCCGTCTGAACGTCAAACCGATACGCCGGCGGCGCGGGCGATTTCCAGGCCTTCTTCGGCACTCATATAGACGGGATTTTCGGGACGGTCGTGCCGGACGATGTTGCCTTCGCGGAACATGACCAGTTCGTTCACGGCAAGTTGGGACCACGATTCATCGCGAGTCAGCGGCAGGGTGGAGATAACGGCGACGCGGTCGTCCGGCGTGGTTACTTCGGCAAAATCGACCATTACATCGTCGTCGAGCAGGCGCGCTTTGCCGAACGGGGCTTGGCGGACGATGTAGTGCAGCAGCGTGCTGGCGTGGGCAAACAGGGAAATGCCGTCTGAAAGCATGAAGTTAAACAGCCCGAACTTGCGGATTTCGTGCGTCAGCCCCGCAATCGCGTCAAACAGCGTGTCGTCGTCGGGACGGGCGGCAAAGCGGGTGCGCAGGCGGTTGAGGATGTGGCAGAACGCGCGTTCGGAATCGGTTGTGCCGACGGGGTGGAAAAATTCTCCTTGTTCGGGGAAAAAATCGACCAAATGCCCGTTGTGGGCAAACAGCCAGTAGCCGCCCCACATTTCGCGCATAAAGGGATGGGTGTTCGCCAGCGAGGTTTGACCTTGCGAGGCTTTGCGGATGTGGGCGACGACGTTTTCCGATTTAATCTGGTAGGCGCGCACGAGGTCGGCGACGGGGGAATTTGCACTCGGCTTGTCGTCATGGAACAGGCGCACGCCTTTGCCTTCGAAAAAGCCGATGCCGAAACCGTCGGCGTGATGGTCGGTAATGCCGCCCCTGCGGCGGAAGCCTTCAAAGGAAAACATAATATCGGTCGGCGTATTGCAGTTCATGCCCAGCAGTTGACACATAGTTTGTCCAAATGATTCAGATGGTCGCAAGTATTCGGATTATACCCCGAACTGAAAATGCCGTCTGAAATACGGCTTGTTCCCCATTATTCCCGCGAAAACAGAAAACAAAGACGGAAACTTAAGATTCCGTCATTCCCGCGCAGGCGGGAATCCGACTTGTCCGGTTTCGGTTGTTTTTCGTTCCGTAACTTTTGAGCCGTCATTAATTTCGGGAAACTTATGAATCGTCATTCCCATGCAGGCGGGAATCTGGAATTTCAATGCCTCAAGAATTTATCGGAAAAAACCAAAACCCTTCCGCCGTCATTCCCACTTTCGTGGGAATGACGAATCTAGAAATGAAAAGCAACAGGAATTTATCGGAAATGACCGAAACTGAACGGACTGGATTCCCGCTTTCGCGGGAATGACGGCGACAGGGTTGCTGTTATAGCGGATTAACAAAAACCGGTACGGCGTTGCCCCGCCCCGGCTCAAAGGGAACGGTTCCCTAAGGCG

>107 |ref|NC_017511.1| Neisseria gonorrhoeae TCDC-NG08107 | Coordinates: 1401116,1403446 | Forward

ATTCCGGATAAATTCCTGTGATTTTGAATTTCCAAATTTCCATCTGCGCGGAAATGACGGCGGACAGGTTGTCGTTATTCCGGATAAATCCCTGCAATATGGAATAACCGGATGCCCGCTTTCGCGGAGAATGATGATGGAAAGTCATCATCGTGCGTCAAACCCGCGCGGCAACACATCGGCAGAGGAAATCAGAGATACGGTTGCAGGGTGCGCCGGAGGATGTCCGTGTCGGTAATTTCGGTGATGACGGCTTCGCCCAGCCGGTTCAGACCGATAAAGCGCATGATGCCGCCGCTGACTTTTTTGTCGTGGCTCATATGTGCCAGCCATTTTTCAAAGGCAAACACGGGCGGTGCGGACGGCAGTCCGGCGGCTTCGAGCAGGGCGGCGAGCCGCGCGGTATCTGCGGCGGAGGTTTTGCCCAGTTGTTCGGACAAACGCGCCGCCAACACGCAGCCGGCGGCAACGGCCTCGCCGTGCAGCCATACGCCGTAACCCATTTCCGCTTCGACGGCGTGTCCGAAGGTGTGTCCGAGGTTGAGCCATGCGCGTATGCCCTGTTCGGTTTCGTCTTGGGCGACGATGTCTGCCTTCATTTGGCAGCAGCGGTACACGGCTTGGGTGAGGGGCGCGCGTTCGAGTGCCATCAGTTCGGGCATATGCTGTTCCAGCCATTCAAAAAAGCCGATGTCGCCGAGCGCGCCGTATTTGATGACTTCCGCCATACCGGCGGACAGTTCGCGGGCGGGCAGGGTGTGCAGCGTGTCCAAATCTGCAAGCACCGCCTGCGGCTGGTAAAACGCGCCGATCATATTTTTGCCGAGCGGGTGGTTGATGGCGGTTTTTCCGCCCACCGATGAGTCGACCTGACTCAACAGCGTGGTCGGTATTTGGATGAACGGCGCGCCGCGCTGGTAGGTGGCGGCGGCAAAGCCGACCATGTCGCCGATCACGCCGCCGCCCAGCGCAATCAGCGTGGTTTTACGTTCGGCGCGGTTTTGCATCAGCCCGTCAAAGATGAGGTTGAGCGTCTGCCAGTTTTTGTGCGCCTCGCCGTCGGGCAGGATGATGCTGAAATGGGATACGCCTGCCGCATCCAATGCCGTCTGAAGCGTGCCGAGGTAGAGCGGGGCGACGGTTTCGTTGGTGATGATGGCGGCGCGTTTGCCCAAATGCGGTTTGAGCAGGCTTCCCGCCTGCGGCAGCAGCCCGTTGCCGATAAAGATGGGGTAGCTGTGCGAAGGCGTGTGTACGGTCAGTGTTTTCATTGTTGTTCCTTAAAGTTTGAACCGCCGGCCCGCCGGGCGGGGTGCGGTTTGGTTTTCGGGGAGCGGCGCATATGCCGGTTTATCGGGATAAGCGTTTGAGCAGGGTTTGCACGGTTTCCCGGCAGTTTGCCGATTCTACGGTAAAGTCGGCGGTTTGGCGGTAAACGGGGTCGCGTGCGGCGTAGAGTTCGCGCAATTTCGCCAAAGGATCGGCAACTTGCAGCAAAGGGCGGCTGTTGTCGCAGCGCGTGCGTTCGAGCAGGGTTTCGGGCGGGGCGTGCAGATAAACGACCGTGCCGCTTTTGCGGATAAGGGCGCGGTTTTCTTCTTTTAACACCGCGCCGCCGCCGGTGGACAGGACGATATGCGGCAGGACAATCAGTTTTTTGAGTATGGCGGTTTCGCGCGAACGGAATCCCTGTTCGCCTTCCATTTCAAATATGGTGGGGATGGGAACGCCCGCCGCTGCGGCGATTTCGTGATCGCTGTCGTAAAAACGGTAATCCAGCCGCTGCGCCATTTGCCGGCCCAGCGTAGTTTTGCCCGCGCCCATCAGTCCGATGAGGATGAGTTTGCCGTTAAAGTTTTTCATCACGGTTCCTTAATGTTTGACCCCCCCCGCCTTTCGGGGCGGCAGAGTTCGGGCTTGTCGGGTTACGGCGGGATTTTATACGAAATCGGCAGGGCGGCGGTACGTTTGGAAAAATAACCCGACCATCCCGAACTTTTCTGATTTTAAGGAAAAATAAAAGAAATCAGGGAGGTTTTTTATTTCAGGCTGTGTTTTGACAATCCGTTGATTTCACTTAATTTGTCAGGAAAAGGCAATTATCTTTGCTTAGGTAAACAATTATCCAATTGAATATATTGAAGATAATATGTTTATCAATACTATAGCGGATGAACAAAAACCGGTACGGCGTTGCCCCGCCTTAGCTCAAAGGGAACGATTCCCTAAGGTGATGTCCCTAAGGCGCCCAAGCACCGGGCGAACCGGTTCCGTACCATTTGTACTGCCTGCGGCCCGCCGCCTTGTCCTGATCTTTGTTAATCCGCTATA

>17 |ref|NC_017511.1| Neisseria gonorrhoeae TCDC-NG08107 | Coordinates: 89531,142807 | Forward

ATCCAGTCCGTTCGGTTTCAGCCGTATCCGACAGATTCCTGCCGCGTTGCGGTTCTAGATTCCCGCCTGTGCGGGAATGACGGCATATCGGCAGGCGAAGGATAAATGCGTAAGGCGGATGCGTAAGATGGGTTGTAGGGTGGACTGTAGGGTGTGCTTCAGCCCGCCGATTCCAACCAACTTCGCCAAAAACCGAAACCGCCGAGTTACGCCTATTCCCCAAAACCCTTGATGCGGTGAAATTAGTGGGCTGAAGCCCACCCTACGGCCCGATGAATCGTCAGTAATACCCGAATCGTCATTCCCGCGCAGGCGGAAATCCGAACACGTCCGCACGGAAACCCATATCCCGTCATTCCCGCGAAAGCGGGAATCTAGAATTTCAATGAGGCAAGAATTTATCGGAAAAAAACCGAAGTTTAAAGACCTAGATTCCCGCCTGCTCGGGAATGACGGGGTGTTTCGGGTTGCTGTTTTTTGTGGAAATGACGAGGCTTTGGATTGCGAGGATTTATCCCTTCCGCCGTTATTCCCACGAAAGTGGGAATCTAGAAATGAAAAGCAACAGGAATTTATCGGAAATGACCGAAACTGAACGGACTGGATTTCCGATTTCGCGGGAATGACGGCTCAAAAGTTACGAGACGAAAAACAACCGAAACCGGACAAGTTGGATTCCCGCCTGCGCGGGAATGACGGCATTTCGGTCGCGGCAAAAAGCATAAAGAAAGGGCATATGCTGTAAAACATATGCCCTTATTTTGACGCATCAATAGCGCAGGCTGTTGCCGGCCGTATCTATAATCCTCGGGGTAATGAAAATCAGCAGTTCGCGGCGGTCGGTTTTTTTCCCGCGTGTTTTAAAGAGGTTGCCGATAACGGGGATGTCGCCCAACAGGGGGACTTTGGTCAGCGTATTGCCGTTGTTTTCTTCATAAATACCGCCGACAATCAAAGTGCCGCCGTTTTCAACCATAGCCTGCGTATTCAGGCTTTTGGTCGAAATACATAGGATTGTGTTGCTGCCTGAAGCACATTGTGCAGGCGAGTCTTTGTTGATTTTGACGGTCATGATGATTTGTCCGTCGGGCGTGATGTTCGGCGTAACGGTCAGCCCCAAGACGGCTTTTTTGAGTTCCGTGTTGGTAGAGTTGCCGCCGCCCGAGGCTGTAGTTACGGTAAAAGGAATTTCGTAACCGGATTCGATTTTGGCCTCTTTGCGGTTTTGGGTCAGCACGCGCGGATTGGCAAGCGTTTTGGTTTTTGAAAGCGACTCGGATGCGGACAATTCCAAATTCAACGCGCCGGAGGAAATCGCGCGCACCAGCGAAATGCTGTTTGCGGCAGCGGCAACCGGCAGGTTGATTTTGGTTTGGGCCTCCCATTTATCGCCGCCCCCGAAGCCGGAGTTCACGCCCCAGCCGAATGCGCTCGTCTCATTTTTCAGTTTTTTCCTGCCTGTCGCGCCGAACTTAACGCCCAAATCGCGCGAGAAGCCGTCTGCCGCTTCGACGATACGCGCCTCAATCATCACTTGTTGCGCGGGTACGTCCAATTCGTCAATCAGTTTGCGGAATTTTTCGATGACGCTGCGGGTATCGGTAACAATCAGGGTGTTGGTGGCGGGATCGATCAGCACGCTGCCCCTGCCGCTGACAAGCGTGTTGCGGTTTCCGGTCGTGTCGGCATTGTCCAAACGCAGGATGCTGCGGAATTCTTCCACATTTTTGTATTTCAATTGGAAGTTTTGGGAATACAGCGCGCCCAGATCGGCAATGTCTTTTTCCGCTTGTAAGAAGGCTTTGTCTTTGGCAAGCAGCTCGTCGCGGGGCGCGATGTTGACGATGTTCCCTTGCTGGCGCATATCGAGGTTGCGCGCCTGCATAACCAAATCCAAAGCCTGATCCCAAGGCACATCTTTGAGGGAGAGGGTCATTTTGCCGTTGACGGAGTCGCTGGCAACAATGTTCATCCCGGATTCTTTTGCCAAAATCTGCAGGATGGTGCGGATTTCGACATCTTGGAAGTCAAGGGAGATTTTCCGGCCTGTGAAGGTTTTGGGCGCATTGTTCACGCCGCCTGACTCGAGGTTTTGTTTTTTCGGCAGGACTTGGAAGGTAAAGTATCCGGGCGCGGCGGATTTGTTGACGAGTTCCCAGTTGCCGGTTGTTGTGATAATCAGCTGGGTGTCGTTATTGAGGCGTTTCAGCGTAACCTTTTGAACCGGTGTTTTGAAGTCTGCCACATCCAAACTGCGTTGGAGCGCGGTCGGCAGGGTATGGTTTTTCAGCGTAACGATGATGTGGTCGTGCTGTTGGCTGATGTCGGGCTGCCCGGCAAAGCCCAATGCCGCCAATTCGATAATGCCGGCATTTTTGCCGTCTTTGCGGAAATCGATATTGGTTTGTTTTGCCGGTGTCGCCGCCTGTTGTTTTGCCGATGCCGCTGCCTGTTGTTTTGCCGGGCTGAACGGTGCGGATACGGATACTACGGACTCGGTAAACGGTGCGGCAGCCTGTTGTTTTGCCGGTGCGGCAGGCGCGGCTTTTACGGCTGGGCGGGCGGGGGCGGACACGGTATCGTCCGATTCGTTAATGAATATCCAAACTTTGTTCCCGCGTACTTCGGTATTGTATTGGCCCGGTTTGTTCAAATTCAGAACCAGACGCGCACGGCTGCTGTTTTGTGCGGCACTGATTTTGCTCAACAGAGGATCGGCATATTCGAGTACCTGTTGATCCATGGAAATGCCGGTTTGTTCAAAGTCCAAGGCGATGCGGGCCGGTGAGGAGGTTACGAAGCCGGTCGGGTTGACAATCTCTTTGTCAAAGCTGACTTTGACGATTTTCTGTTTGTTGGGCAGGGAGGAAACTTTGATGTCTGTAATGTTTCCTGCCGATGCCGTCTGAAAGGCGGCGGTTGCGACAAAGAGACCGGAAATGATTTTTGTCAGTTTGGTATTCATAATGGAGTAATCCTCTTCTTAATTTTGTTCTGCGGCAGGTGCTGCCGCTTGTTCGGTGTTTTTGTCGGAAGAATTCAACAGCAGTTCTGCTTTACGGGAAACCCAGTTGCCCGTGCTGTCTTCTATCAGCTCGTTCAGGATGATGCTGTCGTCGGTAATGCTTTCGATTCTACCGTAGTTTTGTCCCAAATAGTTGCCGACACCGACAGTGTAGACATAACCTTCAGCCTCGATGAAGCCGGAGACTTTCTGTCCGGACTTCAAAATGCCGACATAACGCATATTTTCCAAACTGAATTTTTCCAGCGTTTCTTTAATACGCTTGGTGTCGGGGGCATTTTCCCCTTTTTTGGCGGTTTCCATGCGGCGGAAGTCGAATGCGTTCGGCCCTGTAAGCTGCGGCGGGCTGTATACCGGCGCAACCGGCAGGGTAGGTGCTTGGAAAGGTATGATTTCTGCTTTGGCTTCGCGTCGCGTTTGTGCCATCCATTCGTTTAGGTCTTCAGAACTTTGGGAACACGCGGAGAGAGCCAGAAAGCTGATGAGTAAGGCATAGTGTTTCATGGTTTCCCTAACGTAAGTTATTTTTGCTCGGCATTTTGTGCCGCTTCTGCGGCAAGCTCTTCTACGGATTTTGCTTGGTAGGTGGTGGCAATGGCGCTGAGGTTCAGGATACTGCTCTTGCCGTCAGGATTGCCGCCGTTTTCCGGAGATTGGGCGATTTTCAGCGACTCAAGGGTAATGATTCGGGAGAGACTGCCGACATCGCGGGTAAATTGGCTGATCTGTTCGTAATTTCCGGTAATGGAAATGGAATAGGGTAATTTTTTGATGGGACCGTCATCTACGGGAGGTTGGGGCATAACGCTGTCCAAGCGCAGACCGTTGCTCGAACCCGCCTGATGAAGCTCTTGAACCAAATTGGGAATTTCTGCATCTGTCGGCAGCTGTTTCAACATGATATCGAAGGCAGAGCGGATTGAGGCAAGTTCGTCCCTAAGGTTGTTCAGGCTGGCCGCGTCGATACTTTTCTGTTTGTAGGTGTTTTTCAGTTCGGTTTCTTTTGCTTCATATTCCTCAAGGGATTCCATCTGGCTTTTGAACAATCCGGCATAACCGAGCCCCAGCACGGCGGCAACGACCAGCAGGGCGATAAAAAGCCTGGCAGGAAGGTTGAGCAGGTGAAGGTTGTTGAGATCCAAGTTGGTTTTAGATGATTTAGAAGCCATTCAGTTTGCCTCCTGTGCGTTTCCCGAAGCCGGATTCTCTTTGGATTCGGCCGCCTTTACGATAGGTTGTAATGTTGCCTGAAGGGTAAATTCTTGATGCGAATTGTTTTTCTTGATGCTTAACAATTCGGGTTGCTTGAATATGCCGGTATTGGGCATCGCCCTCATCATGGCGGCAACGCGGTTGTCGCTGGATGTCCTGCCGCTGAGCCGATAAGAGTCGGCGGTAACGGCATCCAGCGAGGTTAGGTAGGTGCTTCCGGGGACGGCCTCATTCAGGCTGTCGAGGATTTTTGCGGCTTGGAGGCGTTTGATCTGGAGCTCCCCGATTTTGTTTTTCTTAATCAGGAAGGCATCTTTTTCCTGTTTGAGCTTTTGTATTTCCGACAGCTCGGTATCCAAGTGTGCGATGGAGATTTCCAGCAGCGTGTTTTTTTCCGACTGTTTATTGATCATATTGTCGATAAACAGGTAGGTTGCCGCAACGGCGGCAACGCCCGTCAGCACGGCGCCGTACATCAGCGTTTTAAACTGCTGCTGTTTGCGTTTGTTCATCTCTTCCCTGTAGGGGAGGAGGTTGATTTTGATTAAATTGTTCATAATTATAATCCCCGTACCGCCAAACCGAACGCCTTGGTCAGTGTCGGCGCATCAAGTTCGAATTGTTGTTCGTCTGTTTTGAGGTCGTTCGCAAAATAACGCGCGGGATGGACGCATTGTACATCCGCATTGGTTTGCGAGGCGACGGTTTGGGCGATGCCTTTCTGGCGCACCGCTTCCCCCGTCAGCAGGATATGCTTGATGTCGGTCATATCGTCTGCGGTCTGCGTGGTGTAATAAAACTGCAAGACCCTTTGTATTTCTTGGGTAATCTGCTGGTTGAAATAGTTTGCCACGCTTTCTTGGTAATCGGAAGGTTTTTGCGGGGAGTTGATGATCTCTTCCGCTTTTTCTGCTGTTACCTGATAGGTGCGCTGGATGAGTTGGTTGAGCTGTTCTGCGCTGACGGAGGTTTCCTGTTTGTATAGGATTTTTCCGTCTTGGATGACCAGGGCGTAGGTCTGTGCGGCATATACGCCGAAAATGGCCACTTTTTCGTCCGCAAGCTCGGGGGCAAAATGGTTTATCCACAGCGCGTAGGCGTTGTATTGTCCGAAAATGTCCACATCAAGCGCGGATAATTTCATACCGGCTGCGTTGAATGCGTCAATCAGGGGTTCGATTTCATCCTTTCTCGATGCGACGGACAAAACAGCTTCGCCGACGGCCGATTGGGACAAGACCTGATAGTCGTAATTGGCTTCTTCGAGCGATATCGAGCTGGCTTCGGAGATGGAGGACTCCACGAACCCCTGCAGGTCCAATTCTGCATCTTTGGCCGTGTAGGTCAATTGTTCGATGGTTGCCAGATTTTGCGGGACGGACGCGACGATGTTTTTGCACGAAGTACCCAGTTTGGCATAGGCTTGTTGCAAATATGTAACAAGTTGATCGTAATTTTGGACTTTATTGCCTTGAATGATATTCTTTGGTAATTTGGCAATGACGTATTTTTCCAATTGAATTTGGTTTAAACTACGTCCTGACAATTGTACCATTTTGATGGAATGCTGGTCGATATCGATGCCGATTGCCGCGCGGTTATTGAGTCCCGAAGATTTTTTAGGGAGCTTGGCATCTGTTTTTTTAGGGTTTTTCAAGCTTTTAAACAAGCGCATGATGAAAGTTCCTGCTTTATTTGTACAGTGAGTAACCGTTTCGGTATCCGTAATGGATTCCTTGTTCTTTGCACATTGAAACCGTGCTTTGTAGAAATCGGTTGCTATTTTACTTTATTTAATACCAATAATGGTAAATTATTATTCAGCTATGATTAAAAAGATTTTAACTACTTGTTTTGGTTTGTTTTTTGGTTTTTGTGTATTTGGAGTGGGTCTGGTTGCCATTGCTATTTTGGTAACGTATCCGAAACTGCCGTCTTTGGATTCTTTGCAGCATTACCAGCCTAAAATGCCGTTGACTATTTATTCGGCGGATGGAGAAGTCATCGGTATGTATGGGGAGCAGCGGCGCGAATTTACAAAAATCGGCGATTTCCCCGAGGTGTTGCGGAATGCGGTTATTGCCGCCGAGGATAAACGCTTTTACCGGCATTGGGGGGTGGATGTTTGGGGTGTTGCCCGCGCTGCCGTCGGCAATGTCGTGTCCGGCAGCGTGCAGTCGGGTGCGAGTACGATTACACAGCAGGTGGCGAAAAATTTTTATTTGAGCAGTGAAAAAACGTTCACACGCAAATTCAATGAGGTGTTGCTTGCCTATAAAATCGAGCAGTCTTTAAGCAAAGACAAAATCCTTGAGTTGTATTTCAATCAGATTTACCTCGGTCAGCGCGCCTATGGTTTTGCATCTGCCGCGCAAATCTATTTCAATAAGAATGTCCGAGATTTGACTTTGGCGGAAGCCGCCATGCTTGCGGGACTGCCCAAGGCTCCGTCTGCCTATAATCCGATTGTTAATCCGGAGCGTGCCAAGTTGCGCCAGAAGTATATTTTGAACAATATGCTCGAGGAGAAGATGATTACCGTGCAACAGCGCGATCAGGCATTGAATGAGGAACTGCATTATGAGCGGTTTGTTCGGAAAATCGATCAGAGTGCTTTATATGTGGCGGAAATGGTGCGTCGGGAACTGTATGAGAAATATGGTGAAGATGCCTATACGCAGGGTTTTAAGGTTTATACCACGGTCCGCACCGATCATCAGAAGGCGGCAACCGAGGCATTGCGCAAGGCTCTACGGAATTTCGATCGCGGCAGCAGCTACCGCGGTGCGGAAAACTATATCGATTTGAGTAAGAGTGAAGATGTCGAGGAGACTGTCAGCCAGTATCTGTCGGGACTCTATACCGTCGATAAAATGGTTCCCGCCGTTGTGTTGGATGTTACTAAAAAGAAAAATGTCGTCATACAGCTGCCCGGCGGCAGGCGGGTTGCGCTTGACAGGCGCGCCTTGGGTTTTGCGGCCCGAGCGGTCGATAATGAGAAAATGGGGGAGGACCGTATCCGCAGGGGCGCGGTCATCCGTGTCAAAAACAACGGCGGGCGTTGGGCGGTGGTTCAAGAGCCGTTGCCGCAGGGGGCTTTGGTTTCGCTGGATGCAAAAACCGGAGCTGTGCGCGCGCTGGTCGGCGGTTATGATTTTCACAGCAAAACATTCAATCGTGCCGTTCAGGCAATGCGGCAGCCGGGTTCGACCTTTAAGCCGTTTGTCTATTCGGCGGCATTATCTAAGGGGATGACCGCGTCCACAGTGGTTAACGATGCGCCGATTTCCCTGCCGGGGAAAGGGCCGAACGGTTCGGTTTGGACACCTAAAAATTCAGACGGCAGATATTCCGGCTACATTACTTTGAGACAGGCTCTGACGGCTTCCAAGAATATGGTTTCCATCCGTATTTTGATGTCTATCGGTGTCGGTTACGCGCAACAGTATATCCGGCGTTTCGGCTTCAGGCCGTCCGAGCTGCCGGCAAGCCTGTCTATGGCTTTAGGTACGGGCGAGACGACGCCGTTGAAAGTGGCGGAGGCATATAGTGTATTTGCGAACGGCGGATATAGGGTTTCTTCGCACGTGATCGATAAGATTTATGACAGAGACGGCAGGTTGCGCGCCCAAATGCAACCTTTGGTGGCAGGGCAAAATGCGCCTCAGGCAATCGATCCGCGCAATGCCTATATTATGTATAAGATTATGCAGGATGTGGTCCGTGTCGGTACGGCAAGGGGGGCAGCTGCGTTGGGAAGAACGGATATTGCCGGTAAAACGGGTACGACCAACGACAATAAAGATGCGTGGTTTGTCGGTTTTAACCCTGATGTGGTTACTGCCGTATATATCGGCTTCGACAAACCTAAGAGTATGGGGCGTGCCGGCTACGGCGGTACGATTGCGGTGCCGGTTTGGGTGGACTATATGCGTTTTGCGTTGAAAGGAAAGCAGGGCAAAGGGATGAAAATGCCTGAAGGTGTGGTCAGCAGCAATGGCGAATACTATATGAAGGAACGTATGGTAACCGATCCGGGCTTGATGCTGGACAACAGCGGTATTGCGCCGCAACCTTCCCGACGGGCAAAAGAAGATGATGAAGCGGCAGTAGAAAACGAACAGCAGGGAAGGTCTGACGAAACGCGTCAGGACGTACAGGAAACGCCGGTGCTTCCGAGCAATACGGATTCCAAACAGCAGCAGTTGGATTCCCTGTTTTAAAGACTCCGCAAAATGCCGTCTGAAAAGTCTTTCAGACGGCATTTTAGATTTGGCAGTGGCAATTTTTTAAATGTTTGCGGCCGGTCAAGTGGGGGGAATACGGTTTCCGTATAATTGAGGTCAGTTTTCCTCTGGAGAGGAAGCGGCGGCATCTGCTGCGTCAAACCAGCTTCCGACAGTTCGGTTGGCCTCGTCAATACCTTGTTTTTTCAGGCTGGAAAACAGCTGTACGCTGATGTTTTGCCTGTCGGAATAAGGTTTGAGCAGTTTTTTGACTTGGGACAGGGTTTTTATCTGTTCGTTTTTGGATAATTTGTCGGCTTTTGACAGCAGGATGTGAACCGGTCTGCCGGTCGTGTGGAAAAAGTCCAGCATACGGATGTCGAGTTCTTTTAAAGGATGGCGGGCATCCATAATCAAAACCAGCCCGATAAGCTGTTTCCGATGGCGGAGGTAGTCGCCGAGCAGATTGACCCAATGTGCGCGTACCGCTTCGGGGACTTGGGCATAACCGTAGCCGGGCAAATCGACCATAAAATTGCCGTTCTGCAGCTCGAAGAAGTTGATATGCTGTGTCCGTCCCGGTGTTTTTGAAACGTAGGCAAGACGGACATGGTTGGTCAGGGTATTGATGGCACTGGATTTTCCGGCATTGCTCCTGCCGACAAAGGCAATTTCGAGCGGTGTGTCCGGCAGGTCTTTGAGATGGTTTACCGTCGTGAAGAATTTGGCGTTTTGAAAAAGGTTCATGGGCATATCCTTGTTTTCCGCCGCCGTTTGTCCGACAGCAAAAATATGCGGTTGGTTTTATGTGAAACACAGTGGTAATTTAATGTAAATTTAGTATAGAATAACACGTTTACAGAATCATCGGTTTTAATCGGGTCAAAAATCCCATATTTGAATATAAAAAAGAGCATTGTTGCGTTATCCAATGCTGTAATCAGGAGCACTCCATGAGACGATTGACTTTATTGGCCTTTGTTTTGGCTGCCGGTGCGGTTTCCGCATCTCCCAAAGCAGACGTGGAAAAAGGCAAACAGGTTGCCGCAACGGTTTGTGCGGCTTGCCATGCAGCAGACGGTAACAGCGGCATTGCGATGTATCCGCGTTTGGCGGCACAGCATACTGCTTACATCTATCATCAAACTATCGGCATCCGCGACGGCAAACGCACCCACGGTTCGGCAGCTGTGATGAAACCGGTGGTAATGAATTTGAGCGATCAGGATATTTTGAACGTATCCGCATTCTATGCCAAACAGCAGCCCAAATCCGGCGAAGCCAATCCTAAGGAAAATCCCGAATTGGGCGCGAAAATCTATCGCGGCGGTTTGAGCGATAAAAAAGTGCCGGCGTGTATGTCCTGCCACGGTCCGAGCGGTGCGGGTATGCCGGGAGGCGGAAGCGAAATTCAGGCTTATCCGCGTTTGGGCGGCCAGCACCAGGCATATATTGTTGAACAGATGAATGCCTACAAGTCCGGTCAGCGTAAAAATACCATCATGGAAGATATTGCAAACCGTATGTCCGAAGAAGATTTAAAAGCAGTTGCTAACTTTATCCAGGGTTTGCGTTAATCATCCAAGGGTCTGCTTCAGAAGCCGTCTGAAAAGGTTTTCAGACGGCTTCTGAAAATTTTGCGATAAGTTTTTTCAATCGCAACCGTTGGAATCGATGCAGGCTGTCTTCATTGTCTTGAAATAAAAAGCATCAAGACAGTAGAATCGGGACGTTGTTTTCTGTCTGCCCAATTCTGCTTTCCCATATTCCTGATGGCGGAATAAACACACAATGAGTAAATCCCGTATATCCCCCACACTTCTTTCCCGTCCGTGGTTCGCTTTTTTCAGCTCCATGCGCTTTGCGGTCGCTTTGCTCAGTCTGCTGGGTATTGCATCGGTTATCGGCACGGTGTTACAGCAAAACCAGCCGCAGACGGATTATTTGGTCAAATTCGGACCGTTTTGGACTCGGATTTTTGATTTTTTGGGTTTGTACGATGTCTATGCTTCGGCATGGTTTGTCGTTATCATGATGTTTCTGGTGGTTTCTACCAGTTTGTGTTTAATCCGTAACGTTCCGCCGTTTTGGCGCGAAATGAAGTCTTTCCGGGAAAAGGTTAAAGAAAAATCTCTGGCGGCGATGCGCCATTCTTCGCTGTTGGATGTAAAAATTGCCCCCGAAGTTGCCAAACGTTATCTGGAGGTGCGGGGTTTTCAGGGAAAAACCGTCAGCCGTGAGGACGGGTCGGTTCTGATTGCCGCCAAAAAAGGCACGATGAACAAATGGGGCTATATCTTTGCCCATGTTGCTTTGATTGTCATTTGCCTGGGCGGGTTGATAGACAGTAACCTGCTGCTGAAGCTGGGTATGCTGGCCGGTCGGATTGTTCCGGACAATCAGGCGGTTTATGCCAAGGATTTCAAGCCCGAAAGTATTTTGGGTGCGTCCAATCTCTCATTTAGGGGCAACGTCAATATTTCCGAGGGGCAAAGTGCGGATGTGGTTTTCCTGAATGCCGACAACGGGATGTTGGTTCAGGACTTGCCTTTTGAAGTCAAACTGAAAAAATTCCATATCGATTTTTACAATACGGGTATGCCGCGCGATTTTGCCAGCGATATTGAAGTAACGGACAAGGCAACCGGTGAGAAACTCGAGCGCACCATCCGCGTGAACCATCCTTTGACCTTGCACGGCATCACGATTTATCAGGCGAGTTTTGCCGACGGCGGTTCGGATTTGACATTCAAGGCGTGGAATTTGAGGGATGCTTCGCGCGAACCTGTCGTGTTGAAGGCAACCTCCATACACCAGTTTCCGTTGGAAATCGGCAAACACAAATATCGTCTTGAGTTCGATCAGTTCACTTCTATGAATGTGGAGGACATGAGCGAGGGTGCGGAACGGGAAAAAAGCCTGAAATCCACTCTGAACGATGTCCGCGCCGTTACTCAGGAAGGTAAAAAATACACCAATATCGGCCCTTCCATCGTGTACCGCATCCGTGATGCGGCAGGGCAGGCGGTCGAATATAAAAACTATATGCTGCCGATTTTGCAGGACAAAGATTATTTTTGGCTGACCGGCACGCGCAGCGGCTTGCAGCAGCAATACCGCTGGCTGCGTATCCCCTTGGACAAGCAGTTGAAAGCGGACACCTTTATGGCATTGCGTGAGTTTTTGAAAGATGGGGAAGGGCGCAAACGTCTGGTTGCCGACGCAACCAAAGACGCACCTGCCGAAATCCGCGAACAATTCATGCTGGCTGCGGAAAACACGCTGAATATCTTTGCGCAAAAAGGCTATTTGGGATTGGACGAATTTATTACGTCCAATATCCCGAAAGGGCAGCAGGATAAGATGCAGGGCTATTTCTACGAAATGCTTTACGGCGTGATGAACGCTGCTTTGGATGAAACCATACGCCGGTACGGCTTGCCCGAATGGCGGCAGGATGAAGCGCGGAACCGTTTCCTGCTGCACAGTATGGATGCCTATACGGGGCTGACGGAATATCCCGCGCCTATGCTGCTCCAGCTTGACGGGTTTTCCGAGGTGCGTTCCTCAGGTTTGCAGATGACCCGTTCGCCGGGTGCGCTTTTGGTCTATCTCGGCTCGGTATTGTTGGTTTTGGGTACGGTATTTATGTTTTATGTGCGCGAAAAACGGGCGTGGGTATTGTTTTCAGACGGCAAAATCCGTTTTGCTATGTCTTCGGCCCGCAGCGAACGGGATTTGCAGAAGGAATTTCCAAAACACGTCGAGAGCCTGCAACGGCTCGGCAAGGACTTGAATCATGACTGAACACTATAAAACCCTTCCGGAACACGAGCTGCTGATTCAGAAATCTTTGATCCGCAATCTGAATCTTTGGGATTGGGTATTTGCCGTGCTGGTTTTTGCGGCTACGGTTTTCGTGCAGACCCGTTCCGGTATGCATATGGACATTTACGAAACGGTCATGTTGTGGGCGAGTGCCGGTATTGCCGTGTTTTTGGGTTGGTTTTTCAAACCGATGCGCTGGTTTGTTCCTTTAAGCGTATTGCTTGCCTATGCCGCCGTCGGTCTGTATGGCGGCAACATTAAATCGGCAGAGATTTTCCTGTTGCGGTATTTCCTCAGCAGCCAATCGGCGATCATGTGGCAGTGTGCTTTTGTCTTCTTCGCCCTGTTCGCCTATATTTCGGGCGCGGTTTTGGCAAGCGTGAAAAATGTGCCGACCAACACGCTGTTGGGTATGGGAACCGTGTTTGCATGGGTGTCTGCCGTAGCAGGCTTTACCGGTCTGCTGGTACGTTGGCACGAAAGCTATCTGCTCCGTCCCGATGCAGGGCATATCCCCGTGTCCAACCTTTATGAAGTGTTCATTCTGTTTTTGGTCATTACCGCGCTGATGTATTTGTATTACGAGGGTAAATTTGCCGTGCAGAAATTGGGCGGCTTCGTGTTCGGCTTTATGGCGGTCGTGGTCGGCTTTGTGTTGTGGTACAGCGTGTCGCGTGAGGCGCATACCATCCAGCCGCTGATTCCCGCGCTCCAGTCCTGGTGGATGAAAATCCACGTTCCGGCAAACTTTATCGGTTACGGCGCGTTCTGCATTTCCGCGATGCTCGGTATTGCCGAACTGGTTTCCCTGCGTGCGGAAGAAAAAGGCGGAAAACTGTGGCTGCCGCCGTCGGCATTGATCGACGAGGTGATGTATAAGGCGATTGCCGTCGGCTTTTTGTTCTTTACCATTGCCACCATTTTGGGCGCGTTGTGGGCGGCGGACGCTTGGGGACGCTATTGGAGCTGGGATCCGAAAGAGACTTGGGCGTTCATCGTTTGGTTGAATTACGCCGTGTGGCTGCATTTGCGGCTGGTGGCGGGCTGGCGCGGCAAAGTGCTGGCGTGGTGGGCGATTATCGGTTTGTTCGTAACCGCATTCGCCTTTATCGGCGTGAATATGTTTTTGAGCGGGCTGCATTCTTACGGAACGCTTTGATACGGTGCGACGATGCCGTCTGAAGGGCTTCAGACGGCATGTTCCGTTTTGGGGATACGGCAGTCGTGCCGAAATTCGCTAGAATACGTTTTTCAGTTTTTAACGGCATCAGACCATGTTGGTATTAGGAATCGAATCTTCTTGCGACGAAACCGGCGTTGCGCTGTACGACACCGAACGAGGATTGCGGTCGCACTGCCTGCACACTCAAATGGCAATGCACGCGGAATACGGCGGGGTCGTGCCGGAATTGGCGAGCCGCGACCATATCCGCCGCCTTGTTCCGTTGACTGAAGGCTGTTTGGCGCAGGCAGGCGCATCGTATGGCGATATTGACGCGGTTGCCTTTACGCAGGGGCCCGGTTTGGGCGGTGCGCTGCTGGCGGGTTCGAGCTACGCCAACGCGCTGGCTTTAGCGTTGGATAAGCCCGTCATCCCCGTCCATCATTTGGAAGGACATCTGCTGTCGCCGCTGTTGGCGGAGGAAAAACCCGACTTTCCTTTTGTCGCGCTGTTGGTTTCGGGCGGGCATACGCAGATTATGGCGGTCAGGGGCATAGGCGACTACGAGCTTTTGGGCGAGAGCGTCGATGATGCGGCGGGCGAGGCATTCGACAAAACGGCGAAACTGCTGGGTTTGCCGTATCCGGGCGGTGCGAAACTGTCGGAACTTGCGGAATCGGGCAGGCCCGAAGCGTTTGTTTTTCCGCGCCCGATGATTCATTCCGACGATTTGCAGATGAGTTTTTCGGGATTGAAAACCGCTGTTTTGACTGCCGTTGAAAAAGTGCGTGAGGCAAACGGTTCGGAAACCATACCCGAGCAAACCCGCAACAATATTTGCCGTGCGTTTCAAGATGCGGTAGTAGAAGTGTTGGAGGCAAAAGTGAAAAAAGCCCTGTTGCAGACAGGGTTCAGAACCGTAGTGGTCGCCGGCGGGGTCGGTGCAAACCGCAAACTCCGTGAAACTTTCGGCAACATGACGGTACAAATCCCGACCCCCAAAGGCAAGCCGAAACATCCGTCCGAAAAAGTCAGCGTGTTTTTCCCGCCGATGGCATACTGCACGGACAACGGTGCCATGATTGCCTTTGCCGGTGCGATGCACCTGGGCAAGGGCAGGGAGGTCGGTGCGTTCAACGTCCGTCCGCGTTGGTCGTTGTCCGAAATCGTCAAATGACAAGATGCCGTCTGAAACCTGTTCAGACGGCATTTTTATTTTCGTTACGGCGTTTTATAGCGGTTGTACATAAACAGATACTGCGTCGGAAAACGGCGTATCCAATATTCGGTATTGCGGTTGAACACGGCGGCATCGTGGGCTTTGTTGCCGTTCAATTCCCCTTGGACGGGGCGGATGTGCAACACGAAGCCTTGTCCGTCGGGCAGGCGTTCGCAGCAGAAAAACAGGGTTTTCACGCCTTTGACGTGTGCCAATTTTGCCGCCAGTGTCATGGTGTATGCAGGTTTGCCGAAAAAATCCGCCCACACGCCGCCGCCTTCCTGCGGAGAAGGGACGTGGTCGGGCAGGATGATGGTTGCCTCGCCCGCGCGCAGGGCCTTGATGATTTGTTTGACCCCTTGTATGCCGGTGGGCGCGGTTTTGCCTTTGCCGCGCACCCTGCCCGCCTGCATGATTTTGTCTATCGCTTTGATTTTCGGCGGCTTGTACATGGCGGTCAGGTGGAACGGAAGCTGCTGGCTGATGTAGCGTCCGCCCAAATCGTAGCTGCCGATGTGCGGCGTGATGAACAGCAGCCCTTCGCCCTTGTCCAAAGCCTGCTGCACGTGTTCCCAGCCGTGTACCGCTTTGAACATTGTTTCGATGTCTTCCGGTTTTTTGAAAAACGCGGGGGCAAGTTCCAAACCGCATTTTGCCGTTTCCGCAAAAACGGCTTTGACCGTCTGCGTGTCGGGGTTCAAACCCGCCTGCCGCATATTGGCGACGATGCGCGCGCGGTCTTCCTTTAAAAGGTAAAACGCCAGATGTCCGAGCCGGTTTCCCAGCGTGTGCAGACAGGAAAGCGACAGCAGGGAGAGGCATTTGAGCAGGGCGGTCAACAGGATGTGCATGGCGGTTCGCAAAGGGGGAAACAGCCTGAATTGTAAACGAAACATGCCGTCTGAAAAAGGGAAGTATTGCGGCAATATGCCTTTTCTGCTACGATGCGCGCTGCATTAAGAGTTGGGAATTCCATGCCAACCTGCTTTTCAAACGGAAAGGTAAGGTGGACGGTTGAAAAACCGATGTGGCTCGCCGGAGCAATCCAAACCCGCTTGATGCGGGAATTTTTTTGCCTGTACGAAACGTACGGACAGAGATTCCAAAGCGCCGTTTAAATAGGAATATTTCTCAACTGAATGGCACGAATAGGGAAATTTTGCTATATTTCCCGCTGTCGACATTATGTTCATACAACATGCTGTCTGAAGAAGATGGTTTGTTTTTCAAGGAAAATTTCAATGAGCGAATATCTGTTTACTTCCGAATCGGTTTCCGAAGGCCATCCGGACAAAGTAGCCGACCAAGTATCCGATGCGATTTTGGATGCCATCTTGGCGCAAGACCCCAAAGCGCGTGTCGCGGCGGAAACTTTGGTCAACACAGGCTTGTGCGTATTGGCGGGCGAAATCACCACCACCGCCCAAGTGGACTACATCAAAGTCGCACGCGAAACCATCAAACGCATCGGCTACAACTTCTCCGAGCTGGGCTTTGACGCCAACGGCTGCGCGGTCGGCGTGTACTACGACCAACAATCCCCCGACATCGCCCAAGGCGTGAACGAAGGCGAAGGCATCGACCTGAACCAAGGCGCGGGCGACCAAGGCTTGATGTTCGGCTACGCCTGCGACGAAACCCCGACCCTGATGCCGTTTGCCATCTATTACAGCCACCGCCTGATGCAGCGTCAAAGCGAATTGCGCAAAGACGGCCGCCTGCCTTGGCTGCGCCCTGACGCAAAAGCCCAACTGACCGTGGTTTACGACAGCGAAACCGGCAAGGTGAAACGCATTGATACCGTCGTCCTGTCCACCCAGCACGATCCTGCCATCAGCCATGAAGAACTGAGTAAAGCCGTGATTGAGCAGATTATCAAGCCCGTTTTGCCGCCCGAACTGCTGACCGACGAAACAAAATACCTGATCAACCCGACCGGCCGCTTCGTCATCGGCGGCCCGCAAGGCGACTGCGGTTTGACCGGCCGCAAAATCATTGTCGATACCTACGGCGGCGCGGCTCCGCACGGCGGCGGCGCATTCTCCGGCAAAGACCCGTCCAAAGTGGACCGTTCCGCCGCTTACGCCTGCCGTTATGTGGCGAAAAACATCGTCGCCGCAGGCTTGGCAACCCAATGCCAAATCCAAGTTTCCTACGCCATCGGCGTTGCCGAACCGACTTCGATTTCCATCGATACTTTCGGTACGGGTAAAATCAGCGAGGAAAAACTGATTGCGCTGGTTTGCGAACATTTCGACCTGCGTCCCAAAGGCATCGTCCAAATGCTCGACCTCTTGCGCCCGATTTACGGAAAATCTGCCGCCTACGGACATTTCGGCCGCGAAGAACCTGAGTTTACTTGGGAGCGCACCGACAAAGCGGCCTCATTGAAAGCGGCAGCGGGGCTGTAATTCCGGTTTGAAAATCAAAAATGCCGTCCGAACAGTTCAGACGGCATTTTTATATTTTCCCGATTCAGGCGCGGCGTTCTTTGCACATCAGTTTCGCATCCAGCCAGCCGTCGCCGCCGGAGATGATGTTTTTGGCAACGAAGTTGTCCAAGTCGGGCAGCAGGGAAACGGCGCGGCCGCTGTTGATGATGACGACCACCGCCATCGGTTTGTCGCCCAGCCAATAACCTGCAAGGGCGCGGACATTGTTGAGCGTGCCGGTTTTTAAGCGCAACAGCCCGCCGCTTTGTTTGAAGCGGTTGCGTAAAGTCCCGTCTGTGCCGGCGATGGGCAGCGTGTCGATGAAATCTTGTGCAAACGGGCTGAAATAAGCCGTTTCCAACATTTGCGCCATCATTCTCGCCGTTACCCTTTCTTTTCTGGACAGACCCGAACCGTTTTCCAAAACCAAATCCGCAACATCGATGCCCGATACGGCAAGTTCTCGCCGGACGGCAGACGCCGCCTGTTCGGAAACGGCGGGCAGTTTGCCGTCGCCGCCGAGTTTGAGGAAGACGGAACGCGCAATCAGATTGTCCGAACGCTTGTTCATGTCCGTCAAAATTTCCTTCATCGGTTTGGAGTGTGCAACGGCAAGCGTCTGCGCGCCTTCCGGTGTGTCGGCTATGCCGATGCCGTCTGAAATCCGTCCGCCGCCGAGCAGCCAGCGGTTGGTAAAACTTTGGCGGATCAGTTCGTCAAGCGCGAACATCCGGACACCGACAGGCTTGCCCAAACAGCTTTCGGGAATATTGCCGCGCAATTTCAGCGTATTGCCCGAAAAAGATGCGCGCATCAGTTTTTTGACCGAAGGGCAGGCAGCTTGGGAGGCGGTAATTTTCAAGTTGTTTTGGGCAAAAATATGCGGCAAAGGCGGATCGGTGAGGATGTCGGTACTGCCGGCGGCATTGCGTTCGGCGCGCACCATAACCATACCGGCAGACAGCATAGTCGGATTTGGGGGCGTCATAAACGGCGAACCGCTGTCGGCTTCAAAATGGTCGGGACTGCCGACTTCGCCCCACAGGCTGTGGTCGAGCATCAGGCGCCCCGTGATATTGCGGATGCCTTTGTCGCGCAACTGGCGTTGGACGGCAAGCAGGTTTTCCTGATTGAAAACGGGGTCGCCGCTGCCCGCCCAATACAGGTTTCCGTCAAGCGTGCCGTCGTTTACCGTACCGTTGCTTTTAAACTCGGTCGCCCAGCGGTAATTGCTGCCGAAGGTTTTGAAGGCGGCAAACGCGGTAACGAGCTTCATCGTGGACGCGGGATTGACGGGTATGCCGGCACGGTGGTCAATGATGACTTTTCCGCTGTCAAGCTCTTGGACATATACAGCGATTTCGTTTTGCGGAATGCGGCCGGTATCGAGCGCGTGTGCGGCGAGGGAGGCGAGAAGCAGCAGCAGGGAGGCCGCTGTTTTGGGGAAATTCATAGGTGAATCTGTTACATAAAAAAAGCAATTATAAGGCAAAGCCGGATAAGTGGGAACGCCGGGGCGGCGGACCGGCTTGTTTGCAGGGGAAATCGCATATATAATAATCGTTACCATTATGAAATGATTGAAACGCACAAACTTAATATTCAGGAGGAATGATTGTGGCTAAGAAAATCAGTATTTTGGTGGGCAGCCTGCGCCGTGCTTCGTTTGCGCGCAAAGTGGCATTGAATGCGGCGGAGATGTTCCCCGAAGGCTGGCAGGCGGAAATCGTCGAAATCGGACATCTGCCGCTTTACAATTTCGATTATGACGACCCTGCGGTGGAAGATGTGCCGCTGCCCGAAAGCTACACGGCTTTCCGCGAAACGATTAAGGCTTCGGACGGCATTTTGTTCGTTACGTCCGAAAACAACCGCACCATTCCCGCCTGTTTGAAAAATGCGGTGGACATCGGCTCGAAACCGAATGCCGACGTGGCTTGGAAAAACAAACCGGCCGGCATCATCAGCCATTCCGTCGGCAAGATGGGCGGTTACAGTTCCCAAAAAAACCTGCGCCTTGCCCTGTCGTATTTTGATATGCCCGTAACCGGACAGCCGGAAGTATTTTTGGGCAATTCGCCGACGCTGTTTGATGAAAACGGTAAGTTGATTGACTCGGCAAGGGATTTTGTTCAGTCATATATCAATCAGTTTGTCGGTTTGATTGAAAGAAATGCCAAATAAACAAACCAAATCGAAAAGCCGCAAAACCGGTTCGGGTTTTGCGGCTTTTTTATATGGGCCGGGCAGCGTCAAGGTTTGCCGTTTGCCGGATAATGCGCGGCAAATGATCGGCGTGTTTAAAATCCCGCGTGTACGGCACGCCCCGCGCTCCGCAATGATAGGGGGCAGTTTCGTTTGAATCGGTGCACCGGGACGGATGCCCCCGCGCCGTCCCGATACCGGTTTGCCGGTTGTTCCGTATCGGTTGCCGCCGTGAAATGACGGACGGATGCAGAAATGTCGGCAGGCGTGAATGGAATGCAAGGCTTATCCCGCATATGTTGGCGGTTTTGCCTGCACGGGCGGTTTTATGTTCAAAATAAAATAAAGGAGAAATAATGCTGACGTTTATCGGATTGCTGATTATCGGGGTCATCGTATGGCTGTTGCTGACGGAAAAAGTGTCGCCCATCATCGCATTAATCTTGGTGCCGCTGATTGGGGCGTTGCTGGCGGGGTTTGATGTATCCCAATTAAAAGAATTTTATTCGGGCGGCACGAAATCGGTGACGCAGATTGTGATTATGTTTATGTTTTCCATTTTGTTTTTTGGAATCATGAACGATGTGGGGCTGTTCCGTCCGATGATAGGCGGTTTGATTAAGCTGACTCGGGGTAATATCGTGGCAGTGAGTGTGGGGACGGTCTTGGTGTCGGTGGTGGCACAGTTGGACGGGGCGGGCGCGACGACGTTTTTATCGGTCGTCCCCGCCCTTTTGCCGCTTTACAAGCGTCTGCATATGAATCCTTACCTGCTGTTTTTGCTGCTGACTTCCAGCGCGGGGCTAATCAACCTTTTGCCGCGGGGCGGGCCGATCGGGCGGGTTGCAAGCGTGTTGGGCGCAGATGTGGGCGAATTGTATAAACCTTTGTTGACGGTGCAAATTATCGGTGTGGTGTTTATCCTTGTGCTGTCCCTGTTTTTGGGTGTGCGTGAAAAAAGGCGGATTGTCCGGGAGTTGGGCGCGTTGCCCGCCGTGGCGGATTTGATAAAGCCGGCGCCTTTGTCGGAAGAAGAACAAAAATTGGCGCGTCCGAAACTGTTTTGGTGGAATGTCCTGCTGTTTTTGGCGGCGATGAGCCTGCTTTTTTCGGGCATCTTCCCGCCGGGTTATGTATTTATGCTGGCTGCAACGGCGGCGTTGCTTTTGAATTACCGCAGCCCGCAGGAACAGATGGAGCGGATTTATGCCCACGCCGGCGGCGCGGTGATGATGGCGTCCATTATTTTGGCGGCAGGTACGTTTTTGGGGATTTTGAAGGGCGCGGGGATGTTGGACGCGATTTCCAAAGACCTTGTGCATATCCTGCCGGACGCGTTGCTGCCTTATCTGCATATTGCCATCGGTGTGTTGGGTATTCCGCTTGAGTTGGTTTTGAGTACGGACGCTTATTATTTCGGACTGTTTCCGATTGTGGAACAGATTACCTCGCAGGCGGGCGTTGCACCCGAAGCGGCAGGCTATGCGATGTTGATCGGCAGTATCGTCGGTACTTTTGTTACGCCGCTTTCGCCGGCTTTGTGGATGGGTTTGGGTTTGGCGAAATTGTCGATGGGCAAACACATCCGTTATTCGTTTTTCTGGGCGTGGGGTTTGTCGCTGGCGATATTGATCAGTTCGATAGCGGCAGGAATCGTGCCTCTGCCGTAAACGGCGAAGCCGCCTGAAGCCCGAATCGTTCCTGTGATCTGTCGCAGGCTTTGCCTGCCTGTCCGTGTGTTGCAATCGCGCCCCAATGCCGTCTGAAGGCAGTTGCCGGCGCGGTTTTTTTGTGCCGCAGTTTTGAAAAATACCGGCAAGCGTAAAGGCCGCTAAATTGCGGCTAATTTTGGGGTGTTAGAATAGGCACGGTTTATTTTGAAGGGAAAGTTGATGCGTGTTTTGCTGGTGGAAGACGATGCGATGATTGCGTAAGCGGTGTCGGCAAGTTTGAAAGACGGCGGCTATGCGGTGGATTGGGTCAAAAACGGCGCGCAGGTTGCGGCGGCTGCCGCTGCCCAGCCTTATGACTTGATGCTGCTGGATTTGGGTTTGCCTGGGCGGGACGGTTTGGATGTTTTGTCGGAAATACGCGCGGCAGGCTGTACCGTCCCCGTGCTGATCGTTACGGCGCGGGATGATTTGTACAGCCGGCTGAACGGTTTGGACGGCGGTGCGGATGACTATATCGTCAAGCCGTTCGATATGGCGGAGTTTAAGGCGCGGATGCGGGCGGTGTTGCGGCGCGGGAGCGGACAGGCGCAGGCGTGTCTGTCAAATGGTGCGTTATCCCTCAATCCTGCAACGTATCAGGTAGAAATTATTGCCGAGGGGAGGCAGGTGGCATTGAGCAACCAGGAGTTTTCGGTATTGCAGGCTTTGCTGGCGAGGCCGGGTGTGATTTTGTCGCGCTCGGATTCGGAGGACAAGGTTTACGGTTGGGGCGGGGAAGTCGAAAGCAATGCGGTGGATTTTCTGATTCACGGGCTGTGCAAGAAATTGGGTAAGGAAAGCATACAAAATGTGCGCGGTGTCGGCTGGCTGATGCCGCGTCAAGATGCCGTCTGAACAGGAACGAAGATGCCGGACCGTTTTTTTAAAATTTTAAAACATTCGCTTCAGGTCAGAATCAGCCTTGCCCTGATTTGGATGTTTGTTCCGCTGGCAATGCTTGCAGGTATGTTTTCCTACTACGAAACCTTCCACGAAACGGAAGCGTTGCAGGACGACCTGCTCCGTCAGGCGGCATTGTATGTCGGCCCTGATTCCAAATCCGAAACTTTGCCCGAAGGCGACGGCGATACGCGTATTTTGGTACAGATGCCGCAGCAGGAAGACCCTGTTGTCAGCCTGCCCGCGCATCTGGCGGACGGTCTGCACACGCTTCGGGCGGACGGGGACGACGATTATTACCGCGCCTATATCCGCACGACCGAACAGGGACGGATTGCCGTCATGCAGGAAAACGAATACCGTGAAGATTTGGCGGAGGATGCGGCACGGCAAAGCGTGTTGCCCCTGTTGGCGGCACTGCCGCTGATGATACTGCTGACTGTGTGGATTACGCACAAAGCCATGCGCCCCGTCCGCAAATTGTCGCAAAGTCTCGAACAACGCCGAATCAATGACCTGCCTGCTTTGAGTGTGTACAATATTCCCAGTGAAATCAGAGGGTTCGTAACCGCCATCAATCTGCTTTTGAAACGTGTTGATGAAGATATACGCCGCCGTCAGCGGTTTGTCGCCGACGCGGCACACGAATTGCGTACGCCGATGACTGCCCTTTCCCTTCAGGCGGAACGGCTCAACAATATGCCGCTCCCACCCGATGCGGGGCGGCAGTCCGCCGTTTTGCAGCAGAGCATCAGGCGCAACAAACACCTGCTCGAACAGCTTTTGGCACTGGCGCGTTCGCAGTCGGACGAAACCCCTTTGACGAAAACGACATTCGGTCTGCAAAGCCGTTTCCGCCAAGTGTTGCAGGAACTGATGCCGCTGGTTTTGGAAAAACGTCAGGACATCGGTGTGGCGGTCGGAGGCGATGTCGAAGTGTCTGCCGACGAAACGGAAATCTATACGCTGGTTAAAACCTTTGCCGACAACGCGGTACGTTACACGCCGAACGGGGGCAGGATAGATTTGGGTTTCACGGACGAAGGGAAATATCTCGCCGTGTGGGTGGAGGATAACGGGAACGGCATTCCCGAATCCGAATGCGCCCGCGTCCTCGATCCGTTTTACCGTATTTTGGGAACGGAGCAGCAGGGGGCGGGGCTGGGGCTGTCGATTGCCGACACGCTGGCCAAAAAATACGGCGGATATTTGGAACTGACCGACAGCCGACGTTTCGGACACGGGCTGTTGATACGCGCGCTGTTGGACAAGGAAACCCTGAAATAGACAGATGCCGTCTGAAGGCTTCAGACGGCATTTTTTACATCAGCACAATATCGTACTGTTCCTGCGTGTAAGCGGTTTCGACCGCCAAAGAAATCGGTTTGCCGATGAAATCTATCAGCATTGCCAAGGATTGCGATTCTTCGTCCAAAAACAAATCGATGACGTTGGGGGCGGCGAGGATGCGGAAACTTTCGGCATCGTAACGGCGCGCTTCGCGGACGATTTCGCGCTGGATTTCGTAGCACACGGTTTGCGGCGTTTTCAGGCGGCCCCTGCCTTGGCAGGAAGGGCAGGGTTCGCAGAGGACTTGGTTTAAGTTTTCGCGCGAGCGTTTGCGCGTCAGCTCGACCAGCCCCAGGCTGGTAAAACCGTGCAGGGTAACGCGGGTACGGTCGAAGGCGAGGGCTTTGGCAAGCTCCTGCAACACGGCTTCGCGGTGGCTTTCCTGCGCCATATCGATGAAGTCGATGATGATGATGCCGCCAAGGTTGCGCAGCCTTAATTCGCGGGCGATGGTGTGGCAGGCTTCGAGGTTGGTGCGGAAGATGGTTTCGTCGAAATTGCGCGCGCCGACGAAGCCGCCGGTGTTGACATCGATGGTGGTCATGGCTTCGGTGGACTCGATAATCAGGTAGCTGCCGAAGTTGAGGTTGACGCGCGGTTGCAGGGCGCGGCTGATTTCCTGTTCGATGTTGTGGGTTTCAAACAGCGGGCGTTCGCCTTTGAACAATTCTATCCTGCCCAATGCGCCGTGGACGTATTGTTCGGCAAAACGCGTCATGCGCCCGTGGTTTACGGTGGAATCGACGAGGATTTTCTGCGTGTCGCAGCCGACCATATCGCGCAACACGCGCAGGCTTAAAGGCAAATCCTGATAAAGCAGGGTTTCCGGCGGCCGGATTTTCGCCTGTTCTTGGATGTGTTCCCACACTTTGGTCAGGTAGTCGATGTCGGACTGGAGCTGTTCGTCGGTGGCGTTTTCGGCGTTGGTGCGGATGATGTAGCCCCGGCAGGCATTTTCCGGCAGGAGCTTGTCGAGGCGTTCGCGCAGGCTGCTGCGTTCGGCATCGTCTTCGATGCGTTGGGACACGCCGATGTGGTCTTCTTGCGGAAGGTGGACGAGGAAACGCCCCGCCAGCGAGATTTGGGTGGAAAGCCGCGCGCCTTTGGTGTTGATCGGGTCTTTGATGACCTGCACCAAAACCGACTGCCCTTCAAACAGCATATGTTCGATGCGCTGGGTTTCTTCGGGGTTGCGGCGTTGTTCGAGGACATCGACGATGTGTAAAAATGCCGCGCGTTCCAAGCCGATGTCGATAAACGCGCTCTGCATCCCGGGCAGCACGCGGCGCACCACGCCCAGATAGATATTGCCGACCAGGCTGTGCCCGCTGTTGCGCTCGATGTGCAGCTCGCAGATATTGTTTTCCTCCAACACCGCCACGCGCGTTTCCTGCGGCGTGATGTTGACCAATATCGTTTCGGGCGGGCGCGCGATGTCTTTGGGGATGGGGAGTCCTGACAACATGGTTTTTCCTGAAAATGTAATAAAAATATTTTTGCATCCGTTCCGCCCCGCCGCGCAGAGCGGGTGCAAAAATATTTTTCACAATGCCTATCATACTTTAAAAAAGAAACTTTGACACACTCCGCACATGCCGTCTGAACGTGCATCTGTTCCGCACTTGCCAAACGGAGCGATTGCCCCTATATTGATTATCATTGCAAAACTTTCGGAAAACCAATATGCAGACCGTTACCATGTACACAGGTCCGTTTTGCCCCTACTGCGCGATGGCGAAAAGGCTGCTGCACGCGGCAGGTGTCGGACATATCGACGAAATCCGTGTCGATGCAAGCCCCGAAGCCTTTGCCGAAATGCAGCGGCTTTCGGGACAGCGCAGCGTGCCGCAGATTTTCATCGGCGAAACGCACGTCGGCGGATTTACCGACCTCTACCGCCTTCAGCAGGAAGGCGGGCTGGACGGACTGCTGAACCCTTAACCCACAACTAGGAAAACAAAATGAGCGAAGAACTGCAACCCGTATTCAGCATCGAGCGACTGTATGTCAAAGACTTGTCTTTGGAAGTGCCGCACGCGCCGCAAATCTTTTTGGAACAGGGCGATCCCGAAGTGGATATGCGCGTTTCCACCGGCAGTCAAAAGCTGGAAGACGGCTACTACGACGTGGACGTTACCGTAACCGTTACCGCCAAATTGGATAACGAGCGCACGATGTTTTTGAACGAAGTAACCCAAAGCGGTATTTTCCGTCTGGAAAACATCCCCGAAGAAGATGTGCAGCTGCTGTTGGGCGTGGCGTGTCCGAACATCCTCTTCCCTTACGCGCGCGAAGCGGTTTCCGGTACGGTAACGCGCGCCGGCTTCCCGCCCGTCCTGCTTGCGCCGATTAATTTTGAAGCGATTTACCAACAACAGCAGGAAGCCGAAGCCGCCGGGGCTTGATTCCTGCCTGCCGATGCCGTCTGAATCCGTTTCAGACGGCATTTTTGTTTTCGGATAAAATAGGCGCGTCCAAAGGAATCTGCCTATGATGTCGCCCGAAACCCAGAAACAGCTCAAAATCACCGATGTTTCCGCCAAGAAGCTCGACAAACTCAACCTCCATACCGCGTGGGATTTGGTGTTGCACCTGCCGCTGCGTTACGAGGACGAGACGCACATTATGCCGATTAAGGACGCGCCGATTGGCGTGCCGTGTCAGGTCGAGGGCGAGGTTATCCATCAGGAAGTAACGTTCAAACCGCGCAAGCAGCTGATTGTTCAAATTGCCGACGGTTCCGGCAGCGTCCTTTTTCTGCGCTTCATCCACTTTTACGCCAGCCATCAGAAGCAGACGGCGGTCGGCAAACGCATCCGCGCCGTGGGCGAAATCAAACACGGATTTTACGGCGACGAGATGATTCATCCCAAAATCCGCGATGCCGAGGGCGGCGGTTTGGCGGAAAGCCTCACGCCGGTTTACCCGACCGTAAACGGTTTGAACCAGCCCACTTTGCGCCGCATCATTCAGACGGCGTTGGACGTTACGCCGCTGCACGACACCTTGCCTGATGCTTTATTAGGCCGTCTGAAGCTGCCGCGCCTTGCCGAAAGCCTGCGCCTTTTGCATTCGCCGCCGCCGAGTTTCACTATTCATCAACTTTCAGACGGCACGCTGCCGGCGTGGCAGCGGCTCAAATTCGACGAACTTTTGGCGCAACAGTTGTCCATGCGTTTGGCGCGGCAGAAGCGCGTCAGCGGCACGGCGGCGGCATTGCGCGGCGACGGCACATTGACCCAAGCCCTGCGCCAAGCCCTGCCGTTTGCCCTGACCGATGCACAAGAAAAAGTTGTTTCCGAAATCTGCCGCGATATGGCGCAAACCCACCCCATGCACCGCCTGCTGCAAGGCGATGTCGGCAGCGGCAAAACCATCGTCGCCGCCTTATCCGCGCTGACCGCCATCGAATCAGGCGCGCAAGTGGCTGTAATGGCGCCCACTGAAATCCTTGCCGAACAGCACTTCATCAAGTTCAAACAATGGCTCGAACCTTTGGGCATTGAAGTTGTCTGCCTTTTTGGCAGTTTGCGCAAAAAAGCCAAAGACGAAGCCAAAGCCAAACTCGCCGACGGCAGCGTCAAAATCGCCGTCGGCACGCACGCCCTGTTTTCAGACGGCGTGGCGTTTCACAATTTGGGCTTGAGCATTGTGGACGAACAGCACCGTTTCGGCGTTGCACAACGCCTCGCACTCAAAAACAAAGGGCGCGAAGTCCATCAGCTGATGATGTCCGCCACACCCATCCCGCGCACGCTCGCCATGAGTTTCTTCGCTGACTTGGACGTATCCGTCATCGACGAATTGCCGCCCGGGCGCACTCCGATTAAAACGCGCCTCGTCAACAACGTCCGCCGCGCCGAAGTCGAAGGCTTCGTCCTCGGCACTTGCCGAAAAGGGCGGCAGGCATATTGGGTCTGCCCATTGATTGAAGAAAGCGAAACCCTGCAACTGCAAACCGCCGCCGAAACCCTCGCCCGGCTTCAGACGGCATTGCCCGAACCCAATATCGGACTGGTACACGGGCGCATGAAGGCCGCCGAAAAAGCCGAAGTCATGGCGGAATTTGCCGCAGGCCGTCTGAACGTCTTGGTCGCCACCACCGTTATCGAAGTCGGCGTAGATGTGCCCAATGCCGCCCTGATGGTCATCGAACACGCCGAGCGCATGGGCTTGGCGCAGCTTCACCAATTACGCGGACGGGTAGGGCGCGGCGCGGGGGAAAGCGTGTGCGTCCTCCTGTTTGCCGAACCCTTGGGCGAACTCGCCAAAGCGCGGCTGAAAGTCATCTACGAACACACCGACGGCTTCGAAATCGCCCGCCAAGACCTCAATATCCGCGGCCCCGGCGAATTTCTCGGCGCGCGCCAAAGCGGCGTGCCTATGCTGCGCTTCGCCAAGCTCGAAGAAGACTTACACCTTTTGGAACAAGCGCGCGAAACCGCCCCGATGCTGATTGAACAAAACCCTGAAATCGTCGAAGCGCATTTGGCAAGGTGGCTTTCCGGCAGGGAAGGTTATTTGGGTGTGTGAGCAAAATGCCGTCTGAAATATTAGAAAAAAGTGTAACGGAAATCTGATTTTTTATAGTGGATTAACAAAAATCAGGACAAGGCGGCGAAGCCGCAGGCAGTACAAATAGTACGGAACCGATTCACTTGGTGCTTCTTCAGCACCTTAGAGAATCGTTCTCTTTGAGCTAAGGCGGGGCAACGCCGTACCGGTTTTTGTTAATCCACTATATTATTTTTGACACATTTTCATGATTTTAAATCCCGTTATTCCCAAGCGGGCGGAAATCCGGCAATGAGAAGCCGCAGAGGCTTATCGGGAAAACGGCAAGCCCCCCGCCGTCATTCCCGCGCAGGCGGGAATCTAGAAATGAAAAACCACGGTGTTATCGGAAACGATTGAAACCGGAGAAATGACGGAATCGAAGCGTGTGGAAATGTGTCGGAAATTGCTGAAGCCGGATAAACTAGATTCCCGCTTTTGCGGGAATGACGGTATTTTAGATTTTTGTTTTCGCGGAATGATGAGCCAAAAGCCGTCCGAACCCAAAAAAACCGCCGCTTTCAGACGGCATCAAAAAACCGCAGGCACGATGCCTGCGGTTTGCCGTTTGCGCTTCAGGGGAGCAGGGGGATGCCGCCCAAGCCGTGTGTTTCCTCCAGTCCGAACATAATGTTCATATTTTGCACCGCCTGTCCCGCCGCGCCTTTGACGAGGTTGTCGATGACGGAAAGGACGACCCACACATCGGATTGCGCCGCCTGACGGATGCTGATGCGGCAGAGGTTTGCGCCGCGCACGCTGCGGGTTTCGGGTGTCGAACCGGCGGGCAGGATGTCCATGAACGGGCTGTCGCGGTAGTAGTCGCGCAGGACGGTTTCGGGGTCGCTGCCGTCTGAAAGGTGGAGGTAAACGGTGGCGTGCATACCGCGTATCATCGGCGCGAGGTGCGGCGTGAACACGAATCCTTCGGCGATGCCGTCCTGAAGCCCGGCGATGGTCTGCCTGATTTCGGGCAGGTGGCGGTGTCCGGCTGTGCCGTAGGCTTTGAAGTTGTCGCCGGCTTCGCACAACAGCGAACCGACATTGCCTTTCCTGCCCGCGCCGGACACGCCGGATTTGCAGTCGGCAATCAGCGGCATACCGGGCTTCAGACGGCATTGCCGCAACAGCGGCACGAGCGGCAGGGATACGCAGGTCGGGTAGCAGCCGGGGTTGGCGACGAGGCGCGCCTGTGCGACGGCTTCGCGGTTGAGTTCGCTCAATCCGTACACGGCTTGGGAAACGAGGCCGGGGGCGGCGTGGGTCATGCCGTACCAGTGTTCCCAGGTCGGAATGTCCCGTATGCGGAAGTCGGCGGAAAGGTCGATGACGCGCACGCCCTGTTCAATCAGGCGCGGCGCGTCTTTCATGGCGATGCCGTTGGGCGTGGCGAAGAAGACGATGTCGCATTGTTCCAAACCTGCCTCGTCGGGCGTTTGGAAGGCGAGGCCGTACACGCCGCGCAGACTTGGAAAGTAATCGGCAACTGCGGTTCCCGCTTCGCTGCGGCTGGTTACGGCGGCGACTTCGACATCTGGATGGGCGGCAAGCAGGCGCAGCAGTTCCACGCCCGTGTAGCCCGTCGCGCCGACAATGCCGGCTTTGATTTTTTTGCTCATGGTTTTTCCTTTGTGTGGTTGGCGGGTATGCCGTCCGAACGCTGTCCGACGGGGTTTTGATTGGCGGCGGAAGGACGGTACACAAAGCGTTTGAAAAGCGTGTCTAATTTAATGGCATCCGCCTCTGTTTTTAAAACCCAGCCCTGCCGTCCGGAATAAACATAACCGTAGGCGCGAAAGCCGGCAGGATATTTCGGTGCCTGCCCCAAACCCAAACCGTATGCGGCGAAAGCGGTGGTCGTGGAGAAGGAAAGCAGGAGGAGGACGAGGGTTTTCATAAGGTTCCAAACCGAACGGGGCGATGAAGCCCGATTATAGCAAAAGGTCGTCTGAAAACCGTTTTCAGACGACCTTTTATTTAATTACAATAAAAATGTTAACAACAAAAAACAAACCGCTTTTTTCCGTTTGGAGACAAATTTTTAACTAAAATTGTCAACAATTCCTTGACGCACACACAAAAAACTCTAATATTTGTAACTATATGTAACATTATAAATTTTAAATATTTTCTGAGTAAGGGGAAGTAATGGGCATCCATCTCGACTTCGGCATTAGTCCTAAAACGTTCCGACAGACTTATCTGTATCAAAAGCCCAAGCTCTTTAAAGGAGCGGTTCGGAATCTCGAAGCCGCATCTTGTAAATATATCAACGAGATATACCAACGAGCAGACCCAACCGCACCGCTGTTTCATCTGCGTAAAAAAGGCGCAATCGTTCCTAAAGAAGAATACGTCGAAAGTTTCGACGATTTGGGCAAAACTCGCTACCGTTTTATTAAATCCGTTATCTACGAACATATGAAGAACGGTGCGTCGTTAGTCTATAACCATATTAACAACGAGCCGTTTTCAGACCATATCGCCCGTCAAGTCGCCCGCTTTGCCGGCGCACATACTATTGTTAGTGGATATCTTGCTTTTGGCAGCGACGAATCTTATAAAAACCATTGGGACGCCCGAGATATGTATGCCGTCCAGCTTTTCGGCAAGAAACGTTGGCAACTTACTGCCCCTGATTTCCCTATGCCATTGTATATGCAACAGACTAAAGATACTGATATTTCCATTCCTGAACATATCGATATGGATATTATCCTTGAAGCAGGTGATGTCCTCTACATCCCACGCGGTTGGTGGCACAGACCTATCCCGCTCGGCTGTGAAACCTTCCACTTCGCTGTCGGTACCTTCCCACCAAACGGCTATAATTACCTCGAGTGGCTAATGAAGAAATTTCCCACCATAGAAAGTCTGCGCCACAGTTTCTCAGACTGGGAGCAAGATAGGACGCGTATCAACGATACTGCCGCACAAATTGCCGCCATGATTGCCGACCCCGTCAATTACGAAGCTTTCAGTGAAGACTTTCTCGGCAAAGAACGTACCGATACCGCTTTTCATCTCGAACAGTTCGCGAATCCCAACGCTACTCCGCTTTCAGACGACGTCAGGTTGAGACTAAATGCCAATAATTTGGATACGTTGGAAAAGGGATATTTGATTGGGAATGGGATGAAGATAAGCGTAGATGAGTTGGGGAAAAAAGTGTTAGAACACATCGGTAAGAATGAACCGTTATTGTTGAAAAATCTACTGGTTAACTTCAATCAGGCAAAACATGAAGAAGTTAGGAAGTTGATCTATCAGTTGATAGAGTTAGATTTTCTGGAAATTTTGTGAGGGATTCTATGAAAAACTGGAAGCAGTTTATATTTTTCGTAATATTAGTAATAGCTTGTTATCAATTGCTATATTCTTTATCAGATATGTTTCTTCTCGACTACATTAACAAATATAGTTGGAATTTGAATTTTATTCAGGGTACTTTGAATTTTTTTTCAATATATCTGCCATATGTTTTTGTTAGCCGAATTTTTAGGAATACCAACCAAGAAAAGGAGTATAAAAATGATTGAACTTCAACTTCATGAATTGAAGCTGGTTTCAGGGGGAGGTCCTGTAACAGACAATATAGCTGGAAATGTAGCTAATGCTGCCACAACCAAAGGAGGTCCCACATGGGGGGATTTTGTTGCAATACCTGCTGCTGCAGCGACAGTTCATTTCCTGCCGAAAAATTTTTTCGGTGCTATATGGTGCAAATGGTGTATACAATCTGACTCGTGATTGGGTAAATGATGCCGTTAATGCACCTCCTTATAACGGAAGACCAATCTTTGAGATTGAACATGGATTAACTGCTCCCGCAACAAAAGCAGATAAATCAGGAAACGGCTACACTGACGGTACAGATTACTGCTGATATTTCTCATTGTTCAGACGATCTCTAGGGGTCGTCTGAAACTTTCTTAACTTCAATTTTATGAATAGACCCAAGCAACCTTTCTTCCGCCCTGAAGTCGCCATTGCCCGCCAAACCAGCCTGACGGGTAAAGTGATTCTGACACGCCCGTTGTCATTTTCCCTGTGGACGACATTTGCATCGATATCTGCGTTACTGATTATCCTGTTTTTGATATTCGGTAACTATACGCGAAAGACAACAATGGAGGGACAAATTCTACCTGCATCGGGCGTAATCAGGGTGTATGCACCGGATACGGGGACAATTACAGCGAAATTCGTGGAAGATGGAGAAAAGGTTAAGGCTGGCGACAAGCTATTTGCGCTTTCGACCTCACGTTTCGGCGCAGGAGGTAGCGTGCAGCAGCAGTTGAAAACGGAGGCAGTTTTGAAGAAAACGTTGGCAGAACAGGAACTGGGTCGTCTGAAGCTGATACACGAGAATGAAACGCGCAGCCTTAAAGCAACTGTCGAACGTTTGGAAAACCAGAAACTCCATATTTCGCAACAGATAGACGGCCAGAAAAGGCGCATTAGACTTGCGGAAGAAATGTTGCGGAAATATCGTTTCCTATCCGCCAATGATGCAGTGTCAAAACAAGAAATGATGAATGTCGAGGCAGAGCTTTTAGAGCAGAAAGCCAAACTTGATGCCTACCGCCGAGAAGAAGCCGGGCTGCTTCAGGAAATCCGCACGCAGAATCTGACATTGGCCAGCCTCCCCAAACGGCATGAGACAGAACAAAGCCAGCTTGAACGCACCATTGCCGATATTTCTCAAGAAGTTTTGGATTTTGAAATGCGCTCTGAACAAATCATCCGTGCAGGACGGTCGGGTTATATAGCAATACCGAACGTCGAAGTCGGACGGCAGGTTGATCCTTCCAAACTGTTCTTGAGCATTGTTCCCGAACGTACCGAGTTATATGCCCATCTATATATCCCCAGCAGTGCAGCAGGCTTTATCAAGCCGAAAGACAAGGTTGTCCTGCGTTATCAGGCATATCCCTATCAAAAATTCGGGCTTGCTTCCGGCAGTGTCGTATCAGTGGCAAAAACGGCACTGGGCAGACAGGAATTGTCGGGATTGGGCATGGTATCCTCCGATTTGGCGAAGAGCAACGAACCTGTTTATCTCGTGAAAATAAAACCCGACAAACCAACCATCACTGCATACGGTGAGGAAAAACCGCTGCAAATCGGCATGACGTTGGAAGCAGACATCCTACACGAGAAACGGCGGCTGTACGAATGGGTATTGGAGCCGATTTATAGTATGTCGGGCAGGTTGTAAGGCTTGAGGCTGAAAGATAAAAAGTCGTCTGAAACCTCTTCAGACGACCTGTAAAACACTACATAAAAACATAAACACTGAAGCTGCCTGAAACTACCCCCTTTTACAGATACTCTGTCTGTAAAAGCAAGGATAGGGTATCTGCCCTTTCAATTTTTCGATTGACGATAAAAGGAGTTTTGAAATGAGAGAATTGACCATGAACGAAATGACCGCTGTTTTCGGCGGCACAGAAGGTACTTGGGGGCACAGGGGCAGAGTCTCCGGAGAATGGGTCGGAAGCAAGGTCGGCGGCGCTTTAGGGTCGTTTGCCGGAGCATTCACGGGAATGGCGGGCGGCGCATTGGTAACAGGCGGAAATCCTGCCGGAATTGCCATCGGCGGCGCAGGCGGCGGATGGGTAGGAGCTGAGGGCGGTTCTGCATTGGGTGGGCATATCGGCGGTTTTGCCGGTGCAAAATGGGGAAAAATGATCGACAGGCGCAACTGCCAATGCGGAGAAGACTGCCATTGCGACCCGTGCAACTGTTGATCGGGAGTTTGAAATGGATTTCGCACATTACCTGAAACACTGGAAAGCCGCTGTGCTGATTTATCTTGCCATCAGCATCCTGACCGACATCCTTTGCTATTTTTTAAACTTCGACGGTGTGTTTTACAAAGGCAGGTTTTTTTCGGTTACCGTCGCAGGACCTGTAGGGGCTTTGTCTTTTCTTGCGTATCTGCTGTATTTGAAACGCGAGGAAAACCGGTCGCATTGAAAGCCAATATGGGGCTGCAGGCCGTCTGAAACCTTTTCAGACGGCCTTTCCAAACCATTCCCGTCTTCCCATCTCTCTTTTCTGATTCAACAAGGAAATCTATGGATTATCTGCAAAACCTGTCTTTGGGCTTGACAAAAAAGCTGCCCGTTATACTGCAAACAGAAGTAGCGGAGTGTGGCTTGGCATGTCTAGCGGCTGTGGCCGGATTTTATGGTTTCTATACGGATTTGCGCGCACTGCGTTCAAAATACTGTCTGTCACTTAAGGGTGAGAATTTGGCAGATATTGTTCGTTTTGCTGATGATATGGGGCTGACGGGACGGGCGTTGAGGCTGGATTTAGACGAATTGGGCAGTTTGCGCCTGCCCTGTATTCTACATTGGGATTTGAATCATTTTGTGGTGCTGGAATCGGTATCTTCGGACGGGGCTGCCGTCATGGATCCGGCTTCGGGACGACGCAAAGTCAAGACGGAGGAAATATCGCGCAAGTTTACGGGAATTGCTTTGGAACTGTGGCCAAACACGCGTTTCGAGGCAGGGGAAGAAAAGCAGGAAATCCGCATCCTACCCATGTTGCGCGGGATTTCTGGGCTGGGGCGGACATTGTTTCAGCTTTTGGCTTTGGCAGCAGCAATGGAAGTGTTTGCTTTGGTGTCGCCGTTTTTTATGCAATGGGTCATCGACCATGTAGTGGTAACGGGCGATCGTAATCTGTTAGCGACGCTGGCTTTGGGCTTTACCTTGCTACTTCTGGTACAAAATACGGTGTCTGTCATGCAGGGCTGGTTGGGCATGCATTTTTCAACGACGTTAAATGTTCAGTGGAAATCAAACGTTTTCAAACGTTTGATAGATTTGCCTACAGATTATTTCGCTAAACGGCATTTGGGCGATGTGGTATCGCGCTTCGGGGCGGTGGATAGTATTCAGGGAACTCTGACTTCAACGTTTTTCGTGTTGGTTTTAAACAGCCTAACGGCTGTTTTTACTTTTGTGCTAATGATAGTGTACAGCCCGATACTGACAGGTTTGGTTGCAGCGACACTGGTAGTTTATATCGCAGTCCGTTGGGTAGCCTATTATCCGTTGCGACGGGCGACGGAGGAAAACATCATCCATGCTGCCAAACAAAGCAGCTATTTTATGGAAACGGTACGCGGTATCCGAGCAGTCAAGCAATTTGGTAAAGGTCCTCAACGATATTCTGCATGGATGGGGCTGCTGGTGGATACGGTTAATACGGGACTGACGGTGCAGAAACTGGGAATTTGGTTCGGCTTGGCAAACAAGCTGCTTTTCGGTTTGGCAAATATTTTGATTATTTACTTAGCTGCGGGCATGGTATTGGACGGCGTCTTTACCGTCGGCGTACTAATGGCTTTTTTGGCTTATAAGGGACAGTTTGAAGGACGTATTGGCTCGCTGGTCGATCAGTTTGTGCAAATTAAAATGCTGTCACTTCATGCGGAACGGTTAGCTGACACCGTATTGAATGAAACAGAATCGGAAGCTACGCCGGATGTAGGCATTCCTGATATTTCAGACGACATCGAAATCTCAGTGGAAAATGTTTCTTTCCGTTATGCCGATAACGAGCCATATGTTTTACAAAACGTCAGCTTCAAGATCGGACGTGGTGAATCGCTTGCGTTAATCGGACGATCGGGCTGCGGTAAATCGACATTTTTGGATATTTTAAGCGGCAATCTACCTCCCGAATCAGGCAAAGTCATGATAAATGGGCACGACATTTACAGCTTACCGCCACGTTTTATCCGCAATTTGAGTGCGATGGTCAGGCAGGACGATGTTTTATAGTGGATTAAATTTAAACCGGTACGGCGTTGCCTCGCCTTGCCGTACTATCTGTACTGTCTGCGGCTTCGCCGCCTTGTCCTGATTTAAATTTAATCCACTATATTTGCATTTGCTGGCTCAATTGCAGAGAATATCTGTTTTTTCGATACAGAACCGGATAGAGGAAAAATCGAACATTGCGCCTGCCTTGCTATGATTCGCGAAGAAATCTCCGCCATGCCTATGGGCTATGAAACCTTGATCGGCGATATGGGCAGCGCACTGTCAGGCGGACAAAAACAACGTATCGTATTGGCGCGGGCCTTATATTGCGAACCGAAAATCCTATTTTTAGATGAAGCGGCCAGTCATTTGGATATTGCCAATGAAAAAGCAGTCAATGCAAACTTGAATGGCTTGTCTATTATAAAAATTATGGCGGCACACAGAAAGGAAACGGTGGAATCAGCAGATAGGAAAATGTCTTTAGGATAAAAATACAGTTTCAAAAATACTCAAGACTGCTGCTGTTTTTTCGCCTGAGCGTCAAACTCCGCCAGCGTCATGTTCAAAGTCTGCAAACACGGCGTCATTACCGCATCGACAGCTTGGTTCACATGATCCCTTTCCACAGGCAACGGGCGGTACACGAAGAGCTTGAAGAGTTCGCTCAACTCAATCGAATCCGCCCCCGTTTTCAAAACCCAGCCCTGTCTGCCGGAATAGATATAGCCGTACCGCGCCAGCTTTTCCAAAAGCTCGCCCAATTCATCGTAACCCATATTGATATGCCGTCTGAACTCCTGAACGGACAGGGTTCGGCCTTCTTTTTGCGCCGCATCCAGAAGCAGCAGGATTTTCAACACGTCGTCAAACCGTCCGCGCGAGTCGAATCCCCTGCGGAAGGCCTCGCCCTGCCAATAAGACAGCGACGAAGTCAGCACCGCCCCGCCCAAGACCAGCGTCCACAGCAGGTTTAACCACAGCAGGAAAAACGGCACGGCGGCAAACGCGCCGTAAATCGAGCGGTAGCCGTCGAAATTGCCCATATACCAGGTGAACAGGAAACGTGCCGTCTCCAGGCAGAATGCCGTAATCAAAGCTCCGACAAACGCCTGCCGGGCGGGCACGAAGCGGTTGGGCACGAAGCGGTACAGCCCCCACAGCAAAAGCGTCATGAAAGCCAGCCTTGCCGCCGTCTTCAACGCGTCCGCCCATTGTTGCGCTCCGGAGGAGAGTACGGAGTCTTGAACCGACCCGACCATAAAGGAAATGCCCACACCCAAAGACAAAGGCCCGAAAGTCAGCAACGCCCAATAAACGAGGAACTGCATCATCCAGGGGCGTTGCGTGTTAACCCGCCAGATGCGGTTGAACGCATTGTCTATCGTCCGAATCAGCATCAGCGAGGTTACGACCAGCATCACGCTGCCGATGGCGGTCAGCCGGTTTGCCTGATCGCGGAATGCGTCGATATAGTCGAACACCATATCCGCGCCCTGCGGCACAATGGTTTGGTTGACGAAGGAGACGAACGAATCCGACCAGCGGTCGAACACGGGGAAAATCGAAGCGACCGCGACCATTACGGTCAGTACGGGGACGAGTGCCAGCAGTGTCGTAAACGTCATGCTCGCCGCTGCCTGCGGTACGCGCTCTTCACTGAAACGGCGGATGACGAACCATGCAAATGCACAGATTTTATTGTCCGCCAAACCTTGCCAACGTTGTAAAAAGGTCATAATCTCTTACCAGGTTTCATGTTTTCAAACTGTTTCAGACGGCATCTGGACAACCGTTATGCCGTCTGAACACCAAACTATTTTAACGGAATCCGCCCATGAACCCAAATCCCCTCAAAATCCTCGTCCTCTACTATTCCCAAAACGGCAGCACCCGCAATCCCGCACGCCGAATCACTCGCGGCATCGACAGCGTTGAAGGTTGCGAAGCCGTATTGCGCACCGTCCCCAAAGTGTCCGCCGTCTGCGAAGCCGTCAAATAAAGATATTCCCGACAGCGGCTCCCGTCCTGACCGCCGAAGAAAACAATATCGCCTTCGCACAAGGCAGACGCTTGGCGGAACTCGCCGTCAAGTCGGCATACGCCGCGTGTTCAGACGGCATGGCGTTCAGATGCCGTCTGAACGCGTTTGCCTGTATAATCCGCGTCTTTACTGTCCAACTTCGCGGTTCGCAAACCTCCCGCGTTACCAAAACTAGGATTCGATATGTCAAACCAAAAAGCCTTGGTCATCTTCTCGGGCGGTCAGGATTCGACCACCTGCCTGATTCAGGCAATCCAAACCTACGGGCGCGAAAACGTCCAAGCCATCACCTTCCGATACGGGCAACGCCATGCCGTCGAGCTGGAACGCGCAGAGTGGATCGCGCAGGATTTGGGTGTCAGTCAAACCGTACTCGATTTGAGCCTGATGCGGCAGATTACGCACAACGCCCTGATGGACGAAACCGCCGCCATCGAAACCGCCGACAACGGCGTTCCCAATACCTTTGTGGACGGGCGTAACGCGCTTTTCCTGCTTTACGCCGCCATTTTTGCCAAAGGGCAGGGTATCCGGCACATCATCGCGGGCGTGTGCGAAACCGATTTTTCGGGCTATCCCGACTGCCGCGGCGTGTTCGTCAAATCCATGAACGTTACCCTTAATCTGGCGATGGACTACGATTTTCAAATCCACACGCCGCTGATGTACCTGACCAAGGCGCAAACGTGGGCGTTGGCGGACGAAATGGGCGTGTTGGACTACATCCGCGAACAGACCCACACCTGCTACAAGGGTATCGTCGGCGGCTGCCGCGAATGCCCGAGCTGTATCTTGCGCGAACGCGGGCTGGCGGAATGTCTGGAAAGTAAAAAGGCCGTCTGAACGCGCGCAAAGCACAAGGAATACGATATGCCCAAGCTCCATATGTTTTACCTCGGCGGCAATGCCGGCCGGTCGAATATCGAAGTGCACGACATCCAATTTGCCGTGTGCGACGACTACCGCGAAGCCGTCCCCGCACTCAAAGCCGCGTGGTTCGGCGATACGGACAAAATCCACATCGACGGTTGGCAGGTTGTCGAATGGGCGGACGGTTACGACATCGCCGTATCCGAAACGCCCAAAACGAAAATGCCGCCTGAAAACGCTCCGCGCCTGTATTTTGCCAATGTCGGCGGCTATCGCGCGGGGCAGCTTGCCGAGGCGCACGCGTTCGGGCTGTTCGCCGCCGCCACGCCTGCCGAAGCCAAACAAAAAGCCCTGCAAACCCTGTTGACCGACTATGTTCGGCAGCATAAAGACAACTTGAAAGACGTGGACAACCTGCTTGCGCTCGAGCACATCGGCAATTTCCATATCCGCCTGACCCCGAATCCGCACGGCAAACCCGCCGAAATCGGCTTTCAAGGCTATTTGCCCATTTGAAGCAGCGGATATGTTTCCACGCAAAACGACACATCAAGCCACCCTCCGGGCGGAAAGCGGCGGGGTTTTCACTGAAAAGGAATCGGGATGAAAATTACCAAGATATTTACCTTCGACTCCTCGCATATGCTCGACGGGCATGACGGCAAATGCCAAAACCTGCACGGACATACCTACAAACTCGAAATCACCGTTTCAGACGGCACCATCAAAGGCGGAGCGAAAGACGGTATGGTGATGGACTTTACCGACTTGAAAGCCATTGTGAAACAACACATTACCGACCATTTCGACCACGCCTTCATCTACCACGGCGGCAACGGGCGCGAATCTCAAATCGCCGCGCTTTTGGAAGGCTGGAACATGAAAACCCTGCGCCTGCCCTGCCGCACAACTGCCGAAAATATGGCGGTCGAAATGTACTGCCGTCTGAAAAACGCGGGGCTGAACGTGTGCCGCGTCAAATTGTGGGAAACGCCGACATCGTGCGCGGAGTATGAAGGGGGGGAGGGAATATTTTGAACGTATCGATATAGTCAATTCAAATAAGGTATGGTGGATTAACTTTAAACCGGTACGGCGTTGCCTCGCCTCAGATCAAAGAGAACGATTTTCTAAGGTGCTGAAGCACCAAGTGAATCGGTTCCGTACTATCTGTACTGTCTGCGGCTTCGCCGCCTTGTCCTGATTTTTGTTAATCCACTATACCGTAAAGCGGTTTTTTCTCCTGCACGGATTCCCCGTTTTTTCAGACGGCATTTCATACCGGCACACCCATTGAAAGGAACACCCATGAAACTCCTCTCCACCATTCTCGTCCTCCTCGTCGCCGCCGAACATTTCTACATCGCCTGGCTTGAAATGACGCAGATTCCCGGCGAAAAAGCGGCGGAAATGTTCAAGCTGCCTTACGAATTTATGGAACAGAAACGCGTGCAGACCCTGTTTGGCAACCAAGGGCTGTACAACGGCTTTCTTGCCGTCGGGCTTTTGTGGGCGCAGTTTGCCGCTCCGGATAATGCCGTGTACGGCGCGACGGTACTGTTTCTCGGCTTCGTCCTGATTGCCGCCGCGTGGGGTGCGTTTTCGTCCGGCAACAAAGGCATACTCGTCAAACAAGGTTTGCCCGCATTTTTGGCGGCGGCGGTGTTGGCGGTATGAAAAAAATCAGCGTCGCCCCCGAAAATCCCCAGTACCGCATCGTCGAAATCTTTGAAAGCCTGCAAGGTGAGGGCCGAAACACGGGTATGCCTGCCGTTTTCGTCCGCTTGGGCAAATGCAATCTGGCGTGCGGCTGGTGTGATACCGATTATTTGACATTTGGTATGATGAGCCTGTCCGACATCTTAGGCTGTCTGAAAACTTATGCCGCCCGCAACATCATCATCACCGGCGGAGAGCCGACCATCCAGCCGCATCTCGATATGCTGCTGGACACGCTCAAGGCGGAAGGCTATTTCCTCTGTCTCGAAACCAACGGGCTCAAGCCCGCGCCGCCGCAAATCGACTATGTCGCCACCAGCCCCAAAGCCTGCTACGCCGCCAAGTATGAAACCAACTGCATCGCCGAAGCCGACGAAGTACGGATTGTTGCCGACGGCGATGTCCTTGCGTTTTGCGAAAATATGGAACGCAAAATCCGTGCGCGCCATTACTATCTTTCTCCCTGCGAACAAAACGGCGTGATGAACATCTACGACACCATCCGCCAAATCGGTATTTTAAACAGCCGCCCCGACGCGCCCGTGCATTGGCAGTTGAGCGTGCAGACGCACAAATGGGCGGGGATAGAGTAGTTTAAGCAGTGTAACCCAAAGGGCAGGCGTACCTTCTTGCCGATGTTTGACATACGGGGAAAGTGTGCCGCTTCCGCGTGAAACTGCCGACATTTCCGCCGCCCAATCAGGACGAAGCCTTAATGAATAAGATGCCGCGGTTGGGTACACGCCAGGCTTCCTAGATTCCGATGGTCTTTTGAACCTTGCCGATACCCTTTGTCAGTGCGCGCAAATGGCAGGATTGGGGAAAACGAAATGCCGTCTGAAACAGCATTCTGTTTCAGACGGCATTTTTTTGTTGCCGCCAAAAGGGAAAAACCGCCCCGGCAATCGATGTCGAGGCGGTCTGAATATGGTCGGAATGAGAGGATTCGAACCTCCGACCCCTTCGTCCCGAACGAAGTGCGCTACCGGGCTGCGCTACATTCCGAATTAATTAAGGTGTGATTATAGCGCAAAAAATACGGCGTGCCTATACCGTTTTGCCTTTTTGCCGCGTGTCGGGCGGATTTAAAGGGCTGTGTTTGAATACAGTATTGATAAGCATCCTTATCTCAAAGTAATTCAATAAGATAACTTTCTATTTGACCGAAAAAATCATTGCCTTTCCCTGACGAACGGTTGAAATCGGCAGATTGTTGAAACGCAGCCGGTTTAAAAGGCCTCTCCGACTTTTACGCCGCCCGCCGTGTCCTGCGGCGAGGCAAGGCCGGCAACAAAGGCTTGCGCCGCTTGGAAACCCGTTGTCTGTATAACGGCTTGTACCGCCTCATGACCGAGCGAGTTTTCCATGTATTGCCAACGTCCGGCCAAATCCTGGTTGTCGGGAATGGTGAAACCGTCGCGCAATTCATCGACCAAGTCGGGACGGTTGCAGACCAATATGATGTCGCAACCTGCCTCAAAGGAAATGCGGGCGCGTTCTTTGATGCCGCCTGCCCCGCACGCGCCCTCCATGGTCAAATCGTCAGAAAAAATCACGCCTTTGAATCCGATATCGCGGCGCAAAATTTGTTTGAGCCAGATTTCGGAAAACCCTGCGGGCTTTGTGTCCACTTGCGGATAAACGACGTGGGCGGGCATGACCGCCGCCATACCTTCGCGGCTCATAATGCGGAAGGGGGCGAGGTCGGCGGCTTCGAGTTCGTCGAGGCTGCGCCCGTCTTCGGGTAGCACCAAATGGCTGTCCCCTTCGACAAATCCGTGTCCGGGGAAATGTTTGCCGCATGATTTCATACCGCCTTTTGCCAAACCTTTTTGAAGGGCGAGGGCGAGGCGGGCGACCGCTTCGGGATTGCGGTGGAAGCTGCGGTTGCCGATGACGGCGCAGTTTCCCCAGTCCAAATCTAAGACGGGCGTGAAGGACAAATCGATGCCGCAGGCGGAAAGCTCGGTTGCCAAAACCCATCCGATGTGTTCGGCATGAGTTTCGGCAGTTTCCGCGCCTTCACTGTCCCAAATCTCGCCGAGCGTACTCATTGCGGGCAGGCGGGTAAAACCTTCGATGAAACGTTGCACCCTGCCGCCTTCGTGATCGACGGCGATAATGAGTTCGGGTGTGCGCAGGGCTTTGATTTCGGCGGTGAGTGTTTTGAGTTGTTCGATGTTTTGGAAGTTGCGGCGGAAGAGGATGATGCCGCCGATTGCGGGATCGAGCAGGCGTTGTTTTTCTTCTTCGGTCAGGCGGAAGGCGGCAATGTCTGCCATGACGGGGCCGCGCGGAATATGGGGGACGGTCATTGCGGTTTGCTCCAAAAAACTTCAGACGGCATATGCCGTCTGAACAGGGGGGAAGGTCAAACGTCGGCGCGTTTTTTATCTTTCAACAGAAAAATCAGCACCGCCAATACAATACCGGTCGTGCTAAAGCCCAACAGCGCGGATTTTGTCAGACCCAATGCGAGGTAGCCCGATGCGGCGGCGGCGGCAACCGTCAGGGCATAAGGCAGTTGCGAGGTAACGTGGTCGATGTGGTTGCAGCGCGCGCCGGTGGACGACAGGATGGTCGTGTCGGAGATGGGCGAACAGTGGTCGCCGCATACCGCCCCCGCCATTACTGCGGACATACACGGGATAATCAGCGCGGGTTCGACTTTGACCGCCATGGCGGCGGCAATCGGCAGCATAATGCCGAACGTCCCCCAGCTTGTGCCTGTGGCAAACGCCATCACGCTGGCGAGCAGGAAGAGGATGACGGGCAGGAAGCCGGGATGGATGTTGCCCGCAACCAGCGTGGAGAGGTAGTCGCCCGTGTGCATTTCGCCGACAACCGTACTGATGAGCCAGGCAAGGATTAAAATGGCGATTGCGCCGAACATGGATTTCGCACCCTGCCACACGGCTTTGGGATAATCGGCGGTTTTAATCGTGCCGAACGTGCAGAGGACGACGGCAAGCACGCCGCAAGTGCCGCCGAATACCAGCGAAGTGTTTACGTCGGTATTTTCAAATGCCCCCAAAATGCTGAAGGTTTCGCTTGCCTGCGCGCCGGTGTAGATCATGGCGGAAACCGTTGAGGCGATTAAGGCCAAAACGGGAATAATCAATGCGTAAACACGACCTTTGGTAGCGTCTGAAGCGGCGGTTTCGTCGTGGGCTTCGTTCAACGCAGCCTGTTCGAAACGCGCCATCGAGCCGATGTCGAAGGAGAACCATGCGACGACGAATACCATAATCAGGGCAAACAGCGCGTAATAGTTCATCAGGCTCATGGCGACAAACGTCCCCATCGGCGTGTATTCGGTAATTTTGTAGGTAACGAGCAATCCGGCAAGCGTGGCGATAATCGACGCGCCCCAGCTTGAAACGGGCATCAGCACGCACATGGGCGAGGCAGTGGAGTCGAGGATGTAGGCGAGTTTGGCGCGGGAAACTTTAAACTTGTCGGTAACGGGGCGGGCAATCGCACCGACGGCGAGGCTGTGGAAATAGTCGTCGATAAAGGTTACGAACACGAGGCAGGCGGTCAGCATTTTCGCGCCGCGCCGGTTTTTAATGTGCCGTTTTGCCCAGTCGGCAAACGCCTGATTGCTGCCGGAGTAGGTCAGCAGTGAAGTGAAAATGCCCAAAAGTATCAGGAAAACCAAGATTTTTGGTTTGCCCAGCGACCAATCGCCGTCTGCCCAAGCCAAGCCGACGACCATGTCTTTCAGGTGTGTCAGACCGTCGACGGGGTTGCCGCCGACCAAAAAGGCAACGCCGACCAAAATACCGATGCCTAAAGACAGCAGTACGCGGCGGGTAATGACGGCAAGTGCCAGTGCCAAAAAGGGTGGCACAACCGAGAAAAATGAATGTGAATAGTCAATCAGCTGCATGGTTTATGGGGGGTGTTAAGCGTCCGGATGGGTGGGTATCTGTCCGCCTCCGGTTTGGGTTTTGTTGGCAAAATGGGTGGAAATATTTTTTGTCGTAAAAAATATTTGTTTAAAATCAACCAACTGATTTTTGTAAAATGCCCGTTAATCGGTATTGACGGGCATTTTATCATTTAAAAATATTTTGGTTAAATTATGTGCGTTATTGCAGGTTTAATGCGATGAACAGCGTGTTGCCACGGCGCATGACCAGCAGGGGGACGTTTTTGCCTGCCTTGTCCATAGCTTTGCGGAAACCGGCTTCGTCATTGACGGGGACTTGCCCGACGGCGAGGATTTCGTCGCCGCGCCTTAAGCCTGCGCGTTCTGCCGCGTCGGAAACCCGTACGACGACGAGGTGTTTGCCGCTGCTGTCGGTATGTGTCTGAAGGGTAATGCCTGCGGATTCGACCGAGAACGTACCGGATTGCTGTTCGGTGTAGGGGGCTTCATCTGTTTTGGATGATGCGCCGGTATGCTCGGCGGCGTTGCCCAGCTTGGCTTTGATTGTGATTTCTTCGCCTTTGCGCCATACGCCGAGGCTGACTTCTTTTCCCGGCGTAATGGCGCCGACCATGACGGGAAGGTCGCCGGAAGAACGTATTTCTCCGCCGTCGAGGCTGAGGACGATGTCGCCCGCCTGCAGGCCGGCACGTTCTGCGGGGCTGCCGGGAAGGATTTTGGCAATCAATGCGCCGCTGGCTTTATCCAGACCGAACGACTGTGCCAAACCGTAGGATACTTCCTGAATAATCACGCCCAGTTGTCCGCGTTGGACTTTGCCGGTGTTTTTCAGCTGTTCGGCGACATTCATGGCAACGTCAATCGGGATGGCAAAGGAGATGCCCATGAATCCGCCGCTGCGGCTGTATATTTGCGAATTGATGCCGACGACCTGTCCTTTTAAGTTGAACAGCGGGCCGCCGGAATTGCCCGGATTGATGGCAACGTCGGTTTGGATGAAGGGTGTGTAGCTTTCGTTGGGCAGGCTTCTGCCTTTGGCGGACACGATGCCGGCGGTCACGCTGTTGTCAAAGCCGAAGGGCGCGCCGATGGCAGCGACCCATTCGCCCGGTTTCAAATTTTTGGGATTGCCGATTTTGACGACGGGTAGCTCTTCCGTTGCGTCGATTTTCAGAAGGGCGACATCGGATTGGACATCCGAACCGATGAGTTTGGCGGTATATTCGCGCTTGTCGTTGAGCAGGACTTTGATACTGCCCATACCGGCAACGACGTGGGTATTGGTCAGGATGTAGCCGTTTTTGCTGATGATGAAGCCCGAACCGAAGTTCAATCCGCCGTCATCTGCTTCTTCTTGGGGGATTTCGGGCATGTTCGGGACGAGGCGTTTGAAAAATTCGTAGAACGGGTCGCTGTCGGCAAGCGGGTCGGAATCGGTTTCGGCATTGCCGCTGCCGTTTTGGGTGCGCGGGGCGGGGGCTGCCTGAATATTGACGACTGCCGGGCCTTCGCTTTGAACCAGTTGGGCAAAGTCGGGCAGCAGCATACTGACACTGCCGTCGTCTTTGGTGTGTTCGATGCGTTCTACGAAGGATGCTTCTTTTTTGTCCGCACCGAAAAAGCTGCCTGCCTTTTCGCAGCCTGCCAGCAAGGCGGCACACAGTGCCGCCAAAGCGAAGTATTGGTATTTTTTGAACACGTTTTGTCCTTTGTCGGATGCCGGTACCGGCTTTAATGCCGTCTGAAGCGCATTTTGTCGGCTTCAGACGGCATAGGTTGAAATTCTACAACGTCCGTCCGAATTTTCAAGCGTTTCATTTTGAAGGGCGGCGGCGGTCAGGATTTGGCGGGATATTCGCACAAATCGTTGATGATGCAGGTTTGGCATTGCGGCTTTAAAGCCTTGCAGGTGTAGCGTCCGTGCAAAATCAGCCAGTGGTGCGCGTCCATCAGAAATTCTTTAGGAATGAAGCGCATCAGTTTGTCTTCGACTTCGCGTACATCTTTTCCGGGTGCGATTTTGGTTCGGTTGGACACGCGGAAAATATGCGTATCGACCGCCATAACGGGGTGTCCGAACGCTGTGTTCAATACCACGTTTGCCGTTTTGCGCCCCACGCCAGGCAACGATTCCAAAGCCTCGCGGTCTTCCGGCACTTCGCCGTTGTATTTTTCCAGCAGGATGCGGCAGGTTTGCATAATGTGTTTGGACTTGGTTTTATACAGCCCGATGGTTTTCGTGTATTCCATCACGCCGTCCAAGCCCAAATCCAGCATCGCCTGCGGCGTATCGGCAACCGGAAACAGCTTCGCCGTCGCCTTGTTTACGCCGACATCGGTCGCCTGCGCCGAAAGCAGAACGGCGATTAAAAGCTCGAAAGGGGAGTTGAAATTCAGCTCGGTGGTCGGATGGGGGTTGGCGGCGCGGAAACGCTCGAAGATTTCTTGGCGGATTTGTCTGTTCATTTTTTTTGTGCAGTCGGTTTGTGCGGTCGGCATTATAACGCACGGTTCAGGCGGCGTAATATTGCATTCCCCACAGAATGAAGGCGTAACGCGCCGTTTTGCCGATAACCAGCATCAGCCCGCTTGTCCACGGATTCAACCGCAGCCAGCCGGCGGTAAGCGGCAGTGCGTCGCCGACGACGGGCAGCCACGCAAACGCAAGCAGCCAAATACCGAAACGCCGTATCAGATTCAGTGTTTTTTCAGACGGCATTTTTCGGGAAGGCAGCAAACGTCCCATCCAATAGGAAACCATACTGCCCAATCCGTTGGCAAGGCCGGCGCACAGCAACGCGCCGTATGCGTGTTTGGGGAAGTTGCGGACGAACAGGGCGAAGGCGGCTTCGGACGTGCCGGGCAGGAGGGTGGCGGAAGTGAAGGCGGAAAAAGCGAGGGCGGCGTAGGTGTAGGAGGGTATCATTGCAAACAGTCCCAAACAGGTAAAAATCGGCGACGGATTATACGGTATTTTCACGCCCCCGCCGAAGGGCGGAGGACGGTGCAAAAATACGGCACAGCCGTATGCCCCTTTATTTGTCGGGCATACGACATTCTTTCCGCTCCGGTTTTGATGCCCCGATGCGGCATTTCCGAATTTTCCGGATACGGCGGAGGATTTTCATTTTATTGGGAACGGTTTTTGCAAGCCCGCCGGAATTTTTTAAAATCTATTAAAACCTATGCAAACAACTGTAAAATATTAATTTCTGCTGCTTGAATTTCAGATCGGCGCATTGCCTGCATCCGATAAAGTTTGCAAAATGTTCAAATATCAGTATGATTTGCATTGCCGTTAAGAAATGTCAATTTCTATTTTCTTGAAACGGGCAATATTCCGACACCACGAAAGGCAAATCATGTCTGCGCAATCACAAAACAACCATACGTCCCCATTGGTCGTCTTGACCACGCTGTTCTTCATGATGGGTTTTATTACCTGCATGAACGACATCCTTATCCCTCATTTGAAAGAAATTTTCGACCTCTCTTACGTTCAGGCGATGCTGATTCAATTTTGCTTTTTTACCGCCTATGCGGTAATGTCCATCCCGATGGGGGCTTTTGTCGGCAAAGTCGGCTACAAAAACGGCGTTATCGGCGGCTTTCTGCTGACGGCGGTCGGGTGCCTGCTGTTTTATCCTGCTGCGGGCAGCCATTCTTACGCGGTATTTTTGGGCGCGTTGTTTATTTTGGCTTCCGGCGTAACGCTGCTTCAGGTCGCCGGTAATCCTTATGTTACCCTGCTGGCGAAACCCGGCAAGGAATCGGCAACGCTGACGCTGGTTCAGGCGTTTAACGCTTTGGGTACGACCATTGCGCCTCAAATCGGCGCGTTCCTGATTTTGGCGGACGCAACCCAAACCGTCAGCAAGGCGGAACAGATTTCTTCCGTACAGATTCCCTATTTGGGACTGGCGGGGCTGCTGATTATCCTTGCCGTTTTCGTGAAAATGATCCGGCTGCCCGACGCGCGCAAAATTGCCGCCGAGGAAAGCGCGCACAACCACGACGGCAAAACCGGCGTATGGCAATACAAACATCTCGTGTTCGGTACGGCAGGCATTTTCTGCTACGTCGGTGCGGAGGTGTCTATCGGTTCGCTGATGGTCAATGTGTTGGGTTATCTGAAAGGGCTGGATCATGCTTCTGCCGCGCATTACCTGTCGTTTTATTGGGGCGGAGCGATGGTCGGACGTTTCCTCGGTTCGGCGGTAATGGCGAAATTCGCGCCCAACCGTTATTTGGCGTTTAACGCATCGGCTGCGGTCGTGCTGCTTGCCGTCGCGATGGCGACGGGCAGCGGCAATGCGGATGTGGCGATGTGGTCGCTGCTTGCCATCGGTTTTTTCAACTCGATTATGTTCCCGACGATTTTCTCTTTGGCAACCAAAGGGTTGGGCAAATTCACCAACGCGGCTTCCGGTGTGCTGTGTACCGCGATTGTCGGCGGTGCGGTCGTGCCGGTAATTCAAGGTTGGGCGGCGGATACTTACACCCTGATGTCTTCGTTTGTCGTTTCCGTTATCTGTTACCTGTATATCGTGTTTTTTGCGGTGTACGGATATAGGGCGGACAAATAATCTTTTTCTTGAGAAATGCCGTCTGAACATCTTTCAGACGGCATTTTTGCGTACCGGTGTTTGCGGCGTGTGTGCCGAGGTTTTAATACTTCAATCCATAAAAGTCTTATATGCCAACAAATAAAAAAATAAAAATTATATTTCAAAAAAATTAATTTAAATCGAGAAAATTGCCGTTTTGTTTCTGCCCGGCTTTTGTAAAACGCTAAAATGCCGTCTGAAAACGTCGGGCAGATTCGGCATGGTGTGTTAGAATCCGTTAACTTTATATCAAATCGGGCAAAGAATCATGTTTGCTTTCAAATCCTTACTCGATATGCCGCGCGGTGAGGCACTTGCCGTCGTCGTCGCTCTGATTGCCGCAATGGGCTATACCATCATTTCATTGGAGTGGCTGCCGCATATGTCCATTATTGCCGCCATCGTCGTGCTGATTTTGTACGGCTTGGCGCGCGGTTTGAAATACAACGATATGCAGGCAGGGATGATAGGCGCGTTGAATCAGGGTATGGGCGCGGTTTACCTGTTTTTCTTCATCGGGCTGATGGTCAGCGCGCTGATGATGAGCGGCGCGATTCCGACGCTGATGTATTACGGTTTCGGGCTGATTTCCCCGACTTATTTTTATTTTTCCGCCTTCGCGCTGTGTTCCGTCATCGGCGTGTCCATCGGCAGCAGCCTGACCGCCTGCGCCACTGTCGGCGTTGCCTTTATGGGGATGGCGGCGGCGTTTCAGGCCGATATGGCGATGACGGCGGGCGCGATTGTTTCCGGTGTGTTTTTCGGCGATAAAATGTCCCCGCTTTCCGACACCACGGGCATTTCCGCGTCCATCGTCGGTATCGACCTGTTTGAACACATCAAAAACATGATGTACACCACCATCCCCGCGTGGCTTATCAGCGCGGCACTGATGCTTTGGCTTCTTCCCAGCGTCGCCGCGCAGGATTTGAACAGCGTCGAATCCTTCCGCAGCCAGCTTGAAGCCACGGGATTGGTGCACGGCTATTCGCTGATTCCGTTTGCACTGTTGGTCGTTTTGGCATTGATGCGCGTCAATGCCGTGGTCGCCATGCTCTTTACCGTCATTGCCGCCGTTGCCGTAACGTATCTGCACAGCACGCCCGATCTGCGTCAGCTCGGCGCGTGGTTTTATGGCGGCTACAAACTCGAAGGCGAAGCGTTTAAAGACATTGCCAAACTGATTTCGCGCGGCGGCTTGGAGAGTATGTTCTTTACGCAGACCATCGTTATCCTCGGTATGAGTTTGGGCGGGCTGCTGTTTGCGCTCGGTGTGATTCCTTCCCTGCTGGAGGCCGTCCGTACCTTCTTGACGAATGCCGGACGCGCGACGTTCAGCGTTGCCATGACTTCGGTCGGGGTCAATTTCCTGATTGGAGAGCAATATTTGAGCATCCTGCTTTCGGGAGAAACGTTCAAACCCGTTTACGACAAACTCGGCCTGCATTCGCGCAACCTGTCGCGGACTCTGGAAGATGCGGGGACGGTGATTAACCCGCTCGTGCCGTGGAGCGTGTGCGGCGTATTTATCAGCCACGCCCTTGGCGTACCCGTTTGGGAATATCTGCCTTATGCCTTTTTCTGCTATTTGAGTTTGGCTTTAACCCTGTTATTCGGCTGGACGGGGCTGACTTTGAGCAAAAAATAAGCGGATAAGCGAAATGCCGTCTGAACCTGTTTTCCGGTTTCAGACGGCATTTTTATGTTTGGCGGATGGGGCGGATTGAAACAGAAAACGACCGTACCGTCATCCTAAACTGTGCAGAAACGGCGGTGCGGGCAGGCTCTGCTTACTTCACGCGGGTCGCCATCAGCGTGTGCAGGCGGCGGTTGTCGGCGCGGGCGACGGTGAACTGCAAACCGCCGATAAGGACTTTTTCGCCGCGCACGGGCAGGTGTCCCAATTCCTGAATGACCAAGCCGCCGATGGTGTCGGCTTCTTCGCTGCCGTATTCCGTACCGAAAAAGGCGTTGATGTCTTCGATTTCGGTAGCCGCGTGGATGCGCCAGCGTTCGGCGGAAACGGAGTGGATGTTGTCGGCGCTTTCGTCTTCGTCAAACTCGTCTTCGATGTCACCGACGATTTGCTCGATGATGTCTTCAAAGGTGACCAAACCCGACGTGCCGCCGTATTCGTCGATGACGATTGCCATATGGTTGCGCTGTTCGCGGAACTCTTTTAAAAGGGCGGTCAAAGATTTGCCTTCGGGCACGAAAACGGCAGGGCGCAAGACGGATTTCAGGTGGAACTGCTCGGGGTTGAACATATATTTGAGCAGGTCTTTGGCGTGCAAAATGCCCAAAACTTCGTCTTTGTCTTCGCCGATGACGGGGAAGCGCGAATGGGCGGTATCGATGACGTAGGCGGTGATGCGTTCGATGCTGTCGTTTTCTTTCAATACGTTCATGCGGCTGCGCGTAATCATCGCATCGCGCACTTCCAGCTCGGCAAAGTCCAATACTTTTTCCAGCCGGGTCAGTGTGTCGGCATCAAAAACTTCCTGTTCGTGCGCCTGCCGAAGCAGGTTTAATACGTCTTCGGCGGAATCGGGTTCGCGGGCGAGTCGGGCAATCAGGCGTTCAAAAAAATTTGTTTTCGGTTGTGCGCCGTCCATTTTAATGTCCGTCCTCTCGGTAGGGGTTGGGATAGCCTGCCGCCCGCATCAGGCGGATTTCTTCGGCTTCCATTATTTCGGCCTCATCGTCTTTGATGTGGTCGTATCCCATCAGGTGCAGCGTGCCGTGTATGGTTAAGTGGGCAAAATGCCGTTCAGGCGTTTTGCCCTGTTCGGCGGCTTCTTTTAAAACGACTTGCGGGCAGATAATCAAATCGCCGTACAGTCTTTCCGAAACTTGGCAGGGCAGGATTTCGCCTTCGTTGAGCGCGAAACTCAATACATTGGTGGCATAATCTTTGCCCCGGTAGTCGCGGTTGTAGGCGCGGGCTTCTTCTTCGTCCAAAAGAATCAAACCGATGTCGGCGCGGAGGTATTCGTTTTTCAAGGCAGACCACGCCCAGCGGTAAAAATCGCGTTCGGCGGGGATGCCGGCGGCGGAAGAGACGTTTTCAAAGTTCAAATGGAAACGTTGCCGCTGCAACGTTAAGAAAGGGTATTTTTTGGCGCGTTTCATTGTGGCGGGTTTCGTGTTTTGTGGGCGTAAATATAACATAGACCTGACGGTGCCGTCTGAAGAAACGTTCAAAATATGATAGACTCCACGCCGTTTCCATTCTTTGAACGCATTGAACATGAACCCGAAAAAACTTGTTATCGCCAGCCGCGAAAGCCTGCTTGCCATGTGGCAGGCGAAACATATCCAAGGCCGTCTGAAGGCGCTGTATCCCGATTGCGAAGTCGAAATTTTGGGCATGACCACGCGCGGCGACCGGATTTTGGACAGAACTTTGTCAAAAGTCGGCGGGAAAGGCTTGTTTGTCAAAGAGTTGGAACAATCCCTTCAAGACGGGCGCGCCGATTTGGCGGTGCATTCGATTAAGGACGTGCCGATGGATTTGCCCGAAGGCTTCGCCCTTGCCGCCATCAGCGAACGCGCCAATCCGTTTGACGCGTTTGTGTCCAACCGATACGCGCGTTTGGAAGAAATGCCCGAAGGCGCGGTTGTCGGCACATCCAGCCTGCGCCGTGAAGCCCAGTTGCGCGCGCGCTATCCGCATTTGCTTATCAAACCTTTGCGCGGCAATGTGCAAACCCGTTTGTCCAAACTCGACAACGGCGAATACGATGCAATTATCTTGGCTGCCGCCGGGTTGCAGCGTCTGGAATTGGATGAACGCATCCGCATGATTTTGTCGGAATCCGACAGCCTGCCTGCCGCCGGACAAGGCGCATTGGGTATTGAAATTGCCACGCATCGCGAAGATTTGTACGAAGTCTTGAAGCCGTTAAACCACGATACCACACACGCCTGCGTTACCGCCGAACGCGCTTTGGCGCGCGCTTTGGGCGGAAGCTGCCAAGTGCCGCTGGCCGCGTATTGCACTGAAGAAAACGGGCTGCTGATCTTGCGCGGATTGGTCGGGCACCCCGACGGATCGATTGTGTTGCAGGCGGACGCGCAAGCCCCTGCCGGATATGCCGATGCGCTTGGACGTGCGGTTGCCAAAAAACTGGCGGACGACGGTGCGCAGGAATTGATTGGAGCAGTATTGAATACGGAAAATTGATTTTATCGAAAATTTAAAGAAAATAATATAAGTTATTGTTTTTAATTAATTTATTTAATAAGTTCTACTTACCTTATTTCGTCATTCCCGCGCAGGCGGGAATCCAGTTTGCTCGGTTTCAGTTGTTTTTAATCAATTCTTGCAGCATTGGGTTTCCAGATTCCCGCCTGCGCGGGAATGACGGCGGAAAGGTTTTTGTGGCTTCGGATAATACTGTGGCGTTCAAATTTTGAATTTGAGAATGATGATATTCGTATTTTTTATTGTGGCTGTACTAGATTATCCCTAAATTCCACACCGATCCCGCAGGATTTTTAGCTGCCGGGACGGTGTGCCGAAGTTAA

>19 |ref|NC_017511.1| Neisseria gonorrhoeae TCDC-NG08107 | Coordinates: 143100,153709 | Forward

ATTTTAATTGACTATATTTGTGTGAATGATGAAATAAAAATGCCGTCTGAACCCGATTGGATTCAGACGGCATTTTGTATGGCAGGATTTATTCGCTTTCCAACTGACCGACCCATTCTGACAAGGCAGTCAGGCGTTGTTCGGATTTCGCCACGAACGGGTTTTCGGTATCCCACGCGTAGCCTGCCAAAATCGAAGAAAGCATACGGCTGATTTCCGGGCTGCGGTCGGGCGCGTACACGACGTTTTGGAAGCGGAAATCATACCAGCCGTCCACATAGGTGCGGAACGCGTCTACGCCGATCATCAGGGGTTCGGCAAATTCGGTTTGCCAATCGGCGGCTTCGCCTTTGAGTTGTTTTGTCAGCAGATCGGCAGCAAGTTCGGCGGAGTGCAGCGCGATGGTTACGCCCGACGAGAACACGGGGTCGAGGAACTCGGCGGCATTGCCCAACAGCGCGAAATGCCTGCCGTGCAGTGATTTGACGTTGGCGGAATAGCCTTGGATGGAGCGGAACGGAAAATCGTTTTCCCAAACGGCTTTGTCCAAAATTTCGCTCAACATCGGGCATTCGTAAACAAATTTTTTCAACACCGTTTCCGATTCGCCGGCAAGTTTGTCGGGTGTGCCGACCACGCCGACGGAACAGCGGTTGTCGCCGAAGGGAATCAGCCAAATCCATACGTCGCGGTTGTTGCGGATGGGTGGTAATCAGGATTTTGTTGCGGTCGAATTTCGGGTGGGTAATGTTGTCGTCGATGTGCGTGAAATGCGTTTGGCGCGGCGGCAGGTGCGAGGGCGTTTCCAAGTTTAGCAGGCGCGGCAGCACGCGTCCGTAGCCGCTTGCGTCCAAGACGAATTTCGCGGTCAGTTCATAGCTCTCGCCGGTGTCGGTTTCGATGTTCAAGCGGGCAAAATCGCCGCTGTTGTCGAACGCGGTTACGCCGTGCCCGAAACGTACTTCAACGCCTTGTTTGGCGGCTTCTTCAATCAGGATTTTGTCGAACACGGCGCGGCGGACTTGGTAAACCGTGCCGGGGCCGTCTGAAAATTTATCGGTGAAATCAAACTCGGTATAGCGGCTGCCCCACGAAAACGCCGCACCGTTTTTCAACTGAAAGCCGGGCCCGGTGCGAACGGCATCGGCAAAACCGGCTTCTTCCAGCATTTCCATACAGTGCGGCAGCAGGCTTTCGCCGATGACGAAGCGCGGAAAGTGCTGTTTTCCAACACGCAGACTTGATAACCTTTTTTGCGGAGCAGGGCGGATGCGGCCGAACCTGCCGGACCTGCGCCGATTACGGCGACATCGAATTGGGTGGACATTGGGAAATCCTGATGGTTTGAGTGGAAAAGGAAATGATATTGTACAGGCAGGAAATGCCGTCTGAAACTGTTTGCGGAAGAAATTTATCCGGTGCGGGCGGTTGCTCCCAATGCGTCAGTCCGGTTTGAAAAATGCCGTCTGAAACGGGAAATGTTCAGACGGCATTTGATTTTCAGGGTTATTTTACGCCGTAACGCGGTTGGAGCAGGGCGCACGTCCGGTGTCCCCGGCGGCTTCGGCGGCTGCGCCCGGCAATTTTTCCGCAGGCCGCGCCCGCATCGTTTGCCGTGGTTTCCGGCCGGGTATTTTTTTCGCGCTGAGCCCTGGCGGATTCGTAGCGGTTGCGTTGTCTCGGTTCGCTTGTTTCGGTTTTTTCCGGTTTTGAGCCTTCCTGTTCCCACCATCGGGGCTCGAAGCCTTCGATGCGTTCGATGAGCAGCTTGTTGCCGGTCAGTTCTTTGATGGCCTCAAACATTTTCTGTTCGGATTCGTCCATCAGGGAAATCGCCACGCCGTCCGCACCGGCGCGCCCCGTGCGTCCGATGCGGTGGATGTAGTCTTCGGGCTGGGCGGGCATTTCGTAATTGATGACGAAGGGCAGTTCGGCGATGTCCAGCCCACGCGCGGCGATATCGGTGGCGACGAGGACGCGCAGGCTGCCGTCTTTGAAGGCGTTGAGTGTTTCGAGCCTGCTTTGTTGGGAACGGTCGCCGTGTATCGCCTGTGCGGACAGGTTGCGGCGCACCAGTTCGCGCGTTACGCGGTCGACGCTTTGTTTGGTTTTGCAAAAGACGATAACTTGGTTCATATGCAGATCGACAATCAGCCGTTCGAGCAGGTTGCGCTTTTGGAAGGTATCGACGGCGATGATGTGTTGTTCGACGTTGGCGTTGGTGGTGTTTTGGGCGGCAACCTCGACGGTTTCGGGCGCGTTCATGAAGTCTTGCGCCAGTTTGCGTATCGGGGCGGAGAAGGTGGCGGAAAAGAGCAGGGTTTGGCGTTGGCGGGGCAGCATCTGCATGATTTTGCGGATGTCGTCGATAAACCCCATATCCAACATGCGGTCTGCTTCGTCCAGAACGACGATTTCGGCTTTGTTTAAACTGATGTTTTTCTGTTTCACATGGTCGAGCAGCCGTCCGACGGTGGCGACGACTATTTCGCAGCCGGCACGCAGGTCGGCGGTTTGTTTGTCCATGTTGACACCGCCGAAGAGGACGGTATGCCGCAGCGGCAGGTTTTTAATGTAGCCCTGCACGTTTTGGTCGATTTGGTCGGCAAGTTCGCGCGTCGGGGTCAACACGAGCATACGCACGGGGTGCATTGCAGGCGAGGTGCTGGCGGTGGCGTAACGTTTGAGGCGTTCCAGACTGGGCAGCATAAAGGCAGCGGTTTTGCCTGTGCCGGTTTGCGCGGCGGCAAGCAGGTCGTGTCCGGCCAGTGCTTTGGGAATGGCCGCGGCTTGGATGGGCGTCGGGTTTTCGTAACCTTGCGCGGTCAGTGCGGAAACCAGTTCCGTACCCAAACCTAAAGAGGAAAATGGATTACTCATGATTGTAGTCTTTCTTTCAGACCTTATGCCGTCTGAAGCGGGAAACCGATAGGACGGGGAAATAAACTGCCCGCCTGTGAACGGCGGGGACGGGAATTTGTGTGCGGATTATGCCATAAAACGGTGCCCGCCCCAATCGCGGGCGCGTCCGCAGATTGGAAATCCTGCTTAAAAAATGTACAATGGCGCACTTTTTTGAAACGCGGGCCCATTATGCACATCGGCGGTTATTTTATCGACAACCCCATCGCACTTGCGCCGATGGCGGGCATTACCGACAAACCCTTCCGCCGCCTCTGTCGGGCGTTTGGCGCAGGTTGGGCGGTGTGCGAAATGCTGGCCAGCGATCCGACGCTCAGGAATACCGGAAAAACCCTGCACCGCAGTGATTTTGCCGATGAAGGCGGCATCGTTGCCGTGCAGATTGCCGGCAGCGACCCCGAACAGATGGCGGATGCGGCGCGTTACAACGTCGGACTCGGGGCGCAGGTCATCGACATCAATATGGGCTGCCCCGCCAAGAAAGTGTGCAACGTCCAAGCCGGTAGCGCGCTGATGCAGGACGAGCCGCTGGTTGCCGCCATTTTGGAGGCGGTGGTCAAGGCGGCGGGCGTACCCGTTACCCTCAAAACCCGTTTGGGTTGGCACGACGACCATCAAAACCTGCCCGCCATCGCCAAAATCGCCGAAGATTGCGGCATTGCCGCCCTTGCCGTCCACGGGCGCGCGCGCACGCAAATGTACAAAGGCGAGGCGCGTTACGAACTCATCGCCGAGACCAAAAGCCGTCTGAACATCCCGGTCTGGGTCAACGGCGACATCACTTCGCCGCAAAAAGCCGCCGCCGTCCTCAAACAAACCGCCGCCGACGGCATCATGATAGGGCGCGGCGCGCAAGGCAGGCCGTGGTTTTTCCGCGATTTGAAGCATTATGCCGAACACGGCGTTTTACCGCCTGCCTTGAGTTTGGCAGAATGCAGAGCCGCCATTTTGAACCACATCCGCGCCATGCACGCGTTTTATGGTGAGACCGTCGGTGTGCGCATCGCACGCAAACACATAGGCTGGTACATCGGCGAAATGCCCGACGGCGAACAGGCGCGGCGTGAAATCAACCGCTTGGACAATGCGGCGGCGCAATACGACACACTTGCCGGTTATCTTGAAAGGCTTGCCGGAAAAACCGACCGTTGGGCGTGCGGCTATCGGGAAGGGTAGGGCAGTATTGCCATGCCGCCGTTCGGGTTCGGACGGCATCTGTCTGCATGGTTCGGAGGTCGGGCGGAATCCAGGCCGGCGAATGTCGAAAACAATAAATGCCGTCTGAAAACGAGTGGAACGGGTTTCGCCAAAAGGCTTTCAGACGGCATTCCGTCTTAAAAACATTTCAATCAAAAGGATATGCGATGAACCCTGCGACTGCGGACATTGCGCAATGTATCGAGCAGAACTTGAACCAATATTTCAAAGACCTGAACGGCACCGAACCTTGCGGCGTGTACGATATGGTACTGCATCAGGTGGAAAAGCCGCTGCTGGTGTGCGTGATGGAGCAATGCGGCGGCAACCAGTCCAAAGCCTCCGTGATGCTGGGACTGAACCGCAATACCTTGCGTAAGAAACTGATTCAACACGGTTTGCTGTGAATATGTCGGCAACCGTCCGTATCTTGGGCATCGACCCGGGCAGCCGCGTAACGGGTTTCGGCATCATCGATGTCAGGGGGCGCGATCATTTTTACGTCGCCTCCGGCTGCATCAAAACGCCTGCCGATGAGCCTCTGGCAGACAGGATTGCCGTGATTGTGCGGCATATCGGCGAAGTCGTTGCCGTTTACAAGCCGCAACAGGCGGCGGTGGAACAGGTGTTCGTCAACGTCAATCCGGCATCGACGCTGATGCTCGGTCAGGCGCGCGGAGCGGCATTGGCGGCTTTGGTCAGCCATAAGCTGCCCGTTTCGGAATACACGGCCTTGCAGGTCAAACAGGCGGTGGTCGGCAAAGGCAAGGCGGCGAAAGAACAGGTGCAGCATATGGTGGTGCAAATGCTGGGACTTTCGGGAACGCCGCAGGCGGATGCGGCGGACGGTCTTGCCGTCGCGCTGACCCACGCCTTACGCAACCACGGGCTTGCCGCCAAACTCAATCCTTCGGGGATGCAGGTCAAGCGCGGAAGGTTTCAATAGTTTCAGACGGCATTTGTATTTTGCCGCCTGAAAAGAAAATGTGTACCGAGATGAAATTTATATTTTTTGTACTGTATGTTTTGCAGTTTCTGCCGTTTGCGCTGCTGCACAAGATTGCCGGCCTGATCGGTTCGCTTGCCTACCTTCTGGTCAAACCGCGCCGCCGTATCGGCGAAATCAATTTGGCAAAATGTTTTCCCGAATGGGACGAAGAAAAGCGTAAAACCGTGTTGAAACAGCATTTCAAACACATGGCAAAACTGATGCTCGAATACGGCTTATATTGGTACGCGCCTGCCAAATGCCTGAAATCGCTGGTGCGCTACCGCAATAAGCATTATTTGGACGACGCGCTGGCGGCGGGGGAAAAAGTCATCATCCTGTACCCGCACTTTACCGCGTTCGAGATGGCGGTGTACGCGCTTAATCAGGATGTCCCGCTGATCAGTATGTATTCCCACCAAAAAAACAAGATATTGGACGAACAGATTTTGAAAGGCCGCAACCGCTATCACAACGTCTTCCTTATCGGGCGCACCGAAGGGCTGCGCGCCCTCGTCAAACAGTTCCGCAAAAGCAGTGCGCCGTTCCTGTATCTGCCCGATCAGGATTTCGGACGCAACAATTCGGTTTTTGTGGATTTTTTCGGCATTCAGACGGCAACGATTACCGGCTTGAGCCGCATTGCCGCGCTTGCAAATGCAAAAGTGATACCCGCCATTCCCGTCCGCGAGGCGGACAATACGGTTACATTGCAATTCTATCCCGCTTGGAAATCCTTTCCGAGTGAAGACGCGCAAGCCGACGCGCAACGTATGAACCGCTTTATCGAAGAACGCGTGCGCGAACATCCGGAACAATATTTCTGGCTGCACAAGCGTTTCAAAACCCGTCCGGAAGGCAGCCCCGATTTTTACTGACTACATAAAATTACAAAACAAATCAGGCGTTTCAGATCAAAAACCCCGATTGTTTTTGGGAATTTGAAACCCGGGTTGTACAAACAGGATTTGCCGGACGGTTTTAACGGTTCAGTTGTTTGTAAAAACAATGCTTTTTTAAAATTGACAAAAAACGAAATCGGTTTTAAAGGCTTATTCCGAGAACAAAGGGGAGTGGATGCCGAAAACCCGGTTAATATATTATAGTGGATTAACAAAAACCAATACGGCGTTGCTTCGCCTTAGCTCAAAGAGAACGATTCCCTAAGGTGCTGAAGCACCAAGCGAATCGGTTCCGTACTATTTGTACTGTCTGCGGCTTCGCCGCCTTGTCCTGATTTTTGTTAATCCACTATAAAATTAAATTTGTTTAAAAACATAAAGTTGTAAACAAGTATCTCATATAAGCCTTTTTCATTAAACAGATAGTCAGATATTTTGTGCTAAAAATTTATATAATATTTAAATTAATATCAAGTTATAAAAAATATATGGAATTTTATTTTGTTTATTTATAATTTTAAGCAATAATCTAAATCAGGCATTTTTATTCCCCTGTTTTAAAAAATATTTGGCAAGGTGTGAAAAAAGGCGTACATTCCGCTACACAGAATTACAGATACAGCGGAGCAATGCCGTCTGAAAGGATTTTCCGGTCAGTCTTGCGGTTGGTCGGGGTTTCATCGGATACGGTGAAACGAAAGTTTGCCGGCGCAGGGTTGAGCTACGCGGGTAAAGCCGCAGGCGAAAGCCTGTATTGTTTGTGAAGCGTAAATCTCTGATTTGAGGTATTGGGGCAATCCTGTGGGGGTTGCCTCTTTTTTTATCCGCCTTTTAATGACACAATAGGCGCAAGTCTGTTTTTGAATGCAAGGTACTGCCATGAATACGATTTTGGCTTTCGATATTGAAACCGTACCCGATGTGCAGGGTATCCGTACGTTGTATGACTTGCCGTCTTCCCTGCCGGATGACGAAGTGGTGCTGTTCGCGCAGCAGAAACGCCGCGCACAGACCGGCGGCGATTTTATGCAGCACCACCTCCATCAGGTTGTGGCGGTTTCCTGCTGTATGCGCTGGGGGCAGGACAAGGTTCATGTCGGCACCATCGGCGAGATGGACGATGGCGAGGAAGTGGTTATCGCCAAATTTTTCGAGTTGGTGGAAAAACATACGCCGCAACTGGTCAGTTGGAACGGGGGCGGGTTCGATTTGCCCGTACTGCATTACCGCTCCCTGATATACGGCATCAACGCCGCGCGCTATTGGGATACGGGCGACGGCGATTTCGGCGACAGCCGCGATTTCAAGTGGAACAACTACATCAGCCGTTATCACCAACGCCACTGCGATTTGATGGATTTGCTCGCGCTTTACCAGCCGAGGGCGAATGTGCCGCTGGACGATATGGCGAAACTGTGCGGCTTTCCGGGCAAGCTGGGTATGGACGGCAGCAAGGTGTGGGAGGCGTTCCACGCGGGCAGGCTGAAGGAAATCCGCAATTATTGCGAAACCGATGCCGTGAATACGTATTTGATGTATCTGCGTTTCTGTCTGGTCAGCGGCAGATTCGACGCGGACGAATACGAAATGGAAATCAAGCGGATCAGAAACTATCTCTCCGCCCAAACAGAAGACAAACCGCATTGGGCAGAATTTGTCCAAGCGTGGAAATAGAAACCTGCTGTCCGAAGGCGGTACGCTTGTGCCGCCGTTTCGGTTTCAGACGGCGTATGGTGGATTAAATTCAAACCGGTACAGCCTTGCCTCTCCTTGCCTTACTATCTGTACTGTCTGCGGCTTCGTCGCCTTGTCCTGATTTTTGTTAATCCGCTTTAAAAGCTGTATCCGTGCCGTCTGAAGCCTTTTCCAGTCAAACCAAACAATATTGAAAGAAAAAAATGAGCAAAACCGTCCGTTATCTGAAAGATTACCAAACGCCTGCCTACCGCATTCTTGAAACCGAACTGCATTTCGACATTGCCGAACCGCAAACCGTCGTGAAGTCGCGTTTGACGGTCGAGCCGCAGAGGGCGGGCGAGCCGCTGGTGTTGGACGGTTCGGCAAAACTCTTGTCCGTCAAAATCAACGGCGCGGCGGCGGATTATGTGTTGGAAGGCGAGACGCTGACGATTGCAGACGTACCGTCCGAACGCTTCACCGTCGAAGTGGAAACCGAAATCCTGCCGGCGGAAAACAAATCGCTGATGGGGCTGTATGCTTCCGGCGGCAATCTGTTTACCCAGTGCGAGCCGGAGGGCTTCCGCAAAATCACGTTCTACATCGACCGTCCGGATGTGATGTCCAAGTTCACGACCACCATCGTCGCGGACAAAAAACGCTATCCCGTTTTGCTTTCCAACGGCAACAAAATCGACGGCGGCGAGTTTTCAGACGGCCGCCATTGGGTGAAATGGGAAGACCCGTTTGCCAAACCGAGTTATCTGTTTGCTTTGGTCGCGGGCGATTTGGCGGTAACGGAAGACCGTTTCACCACCATGAGCGGCAGAAACGTCAAAATCGAGTTTTACACCACCGAAGCGGACAAGCCCAAGGTCGGCTTTGCCGTGGAATCGTTGAAAAACGCGATGAAGTGGGACGAAACGCGCTTCGGGTTGGAATATGACTTGGATATTTTCATGGTCGTCGCCGTAGGCGATTTCAATATGGGCGCGATGGAAAACAAGGGTTTGAACATTTTTAACACCAAGTTCGTCCTCGCCGACAGCCGCACCGCCACCGATACCGATTTCGAAGGCATTGAATCCGTGGTCGGACACGAATATTTCCACAACTGGACGGGCAACCGCGTAACCTGCCGCGACTGGTTCCAGCTTTCGCTGAAGGAAGGGCTGACCGTGTTCCGCGACCAAGAGTTTTCCGGCGACCGCGCCGGCCGCGCCGTGCGCCGCATCGAGAACATCCGCCTGCTGCGCCAGAACCAGTTCCCCGAAGACGCAGGCCCGACCGCCCATCCGGTGCGCCCCGTCAGCTATGAGGAGATGAACAATTTCTACACCATGACCGTTTATGAAAAAGGCGCGGAAGTGGTGCGGATGTATCATACCCTGCTCGGCGAAGAGGGCTTCCAAAAAGGCATGAAGCTATATTTCCAACGCCACGACGGACAGGCAGTGACCTGCGACGATTTCCGCGCGGCGATGGCGGATGCGAACGGCATCAATCTCGACCAGTTCGCCTTGTGGTACAGCCAGGCGGGCACGCCCGTTTTGGAAGCCGAAGGCCGTCTGAAAAACAATGTTTTCGAGTTAACCATTAAACAAACCGTGCCGCCCACGCCCGATATGGCGGACAAACAGCCGATGATGATTCCCGTCAAAGTCGGGCTTCTGAACCGCAACGGCGAAGCGGCGGCATTCGATTATCAGGGCAAACGCGCAACCGAAGCCGTATTGCTGCTGACCGAAGCCGAACAGATCTTCCTGCTCGAAGGCGTAACCGAAGCCGTCGTTCCCTCGCTGCTGCGCGGGTTCAGCGCGCCGGTGCATCTGAACTATCCGTACAGCGACGACGACCTGCTGCTTCTGCTCGCCCATGACAGCGACGCCTTCACGCGCTGGGAAGCCGCCCAAACGCTCTACCGTCGCGCCGTCGCCGCCAACCTTGCCGCACTTTCAGACGGCATCGGGTTGCCGAAACACGAAAAACTGCTTGCCGCCGTCGAAAAAGTCATTTCAGACGACCTCTTGGACAACGCCTTCAAAGCCCTGCTTTTGGGCGTGCCGTCCGAAGCCGAACTGTGGGACGGCACGGAAAACATCGACCCGCTGCGCTACCATCAGGCGCGCGAAGCCTTGTTGGATACGCTTGCCGTCCGCTTCCTGCCGAAATGGCACGAATTGGACCGTCAGGCGGCGAAGCAGGAAAACCAAAGTTACGAATACAGCCCCGAAACCGCCGACTGGCGCACGCTGCGCAACGTCTGCCGCGCCTTCGTCCTGCGCGCCGACCCCGCGCACATCGAAACTGTTGCCGAAAAATACGGCGAAATGGCGCAAAACATGACCCACGAATGGGGCATCCTGTCCGCCGTCAACGGCAACGAAAGCGATACGCGCAACTGCCTGCTGGCGCAGTTTGCCGACAAGTTTTCAGACGACGCGCTGGTGATGGACAAATATTTCGCCCTTATCGGCTCAAGCCGCCGCAGCGACACCCTGCAACAGGTTCAAACCGCCTTGCAGCATCCGAAATTCAGTCTCGAAAACCCCAACAAAGCCCGTTCGCTCATCGGCAGCTTCAGCCGCAACGTCCCGCATTTTCACGCACAAGACGGCAGCGGCTACCGCTTCATCGCCGACAAAGTCATCGAAATCGACCGCTTCAACCCGCAGGTCGCCGCCCGCCTGGTGCAGGCGTTCAACCTCTGCAACAAGCTCGAGCCGCACCGCAAAAACTTGGTGAAACAAGAATTGCAGTGCATTCGGGCGCAGGAAGGATTGTCGAAAGACGTGGGCGAAATCGTCGGCAAGATTTTGGGTTGAGGCAGTCAAACAGAAAAAACAAGGGTCTGTACCAGATTAGCAGATATGTTGCCCTCGAAATATGAAGATAACACACTGCAAATTAAAGAAAGAAGTACA

>20 |ref|NC_017511.1| Neisseria gonorrhoeae TCDC-NG08107 | Coordinates: 153710,173870 | Forward

TTAACTTCGGCACACCGTCCCGGCAGCTAAAAATCCTGCGGGATCGGTGTGGAATTTAGGGATAATCTAGTACAGCCCCTAAGATAAAGAAACGGCAACACACGCCAACAGAAAAACATATTTGAACTTCATCATATTTTCCACATAAAAGGCAGCCTGAAAATCTTTCAGGCTGCCCTTGTCAAATTATTCCTAGCTTTCGGCTTTTTTGGCAAACCAAACAATCCGATTACCCGCATAATACTTTCCATTTATTGAAATCCGACAAGCCGCGCCCAAAAAATGCCATGCACTGCCGATTTCCGCAGCAATCTTTGTACCGTTTTCTTCAAATTCCAAATATTCACCCAATAATAAACTTGAAACAGAACACGCGGGCAGCAAGTGCCCACCCTACGCTTATTCAAATAATTTGATTAAATAAAGAATAAAGAAAAGGCATCAGGAACAAAATTATAATCTGCCACTTGACTCGCACCGCTTTTAAAGTAAGGGGCATCAAAACCAAAACCGCAAAAAAATAATTTTTGCATTGATTTTTAATAGATTTAAAATTCAAATATAGTGTTTCTCCAGGTTTAAAAAGTTTATCTTTATCTATATATTTGATGCTTTCCCTATCCAAAATAAATATTTCAAACATTAAAAAATCATTACATGACCAAGCCAAATCATAAATTTGATTCAAATGGGTATCATAAAGATAAAAATAATAAGGTTTGGGAACAGGTAAAATATTTAATGGAAAAGGAGGATTAATTTTCTTAATATCTGATGACTGATATCCATATCTTTCTATATTCTTAAAAATTTCATCTTTCTTAAGATAACAAGACATTTTGACAATCATAACCTGTTCATCAGATATATTATTTATTTGCATGGCGCGTATAACACGCCATGCCTGATTAAAATTAGTCTCCCTTACCTTAGAATTTATTATCTTTTCCAAAGATGAAAATGCCCCTTATCTTGAACTCTCCAGTTAGAACCTGGAAGTTTCGTACCTCTTAAATGTGTATTAATATTTCTTATAGTTTCATTAAAATGCCACGCGCTGCCGATTTCAACGGTAATTTTCGTACCGCTTTCTTCAAATTCCAGGTATTCCCCCATTAGCGAACGCAAAGAAGCAGACGCCATCCCGGCTTCGTTATGATAAACCCGCCTGCCGTTGATATAGACTTCCGCCCCTGTCCGTTTCAAATTCCAAAAATTCCGTACTGAAATTTCCATATCCCGATATTGTGCAGACCATGTTTTTTCGAAGGTTTTCATAAAATTTCCTATACCTGTCCAATCGGCACATATCAATTGCATTATTACATCTCAATACGATAAATATTTCTTAAGTCAAAATGCAAGCCTGACCGTACCTCAACTGTCAAAATTTTATTATTTTTTATTGATTTTAAGACAATTTCTGAAAAATTCTCTTCGCTTTCTCCCTTTTGTAGAAGCACATCAGAAAAAATAAAACTTTCCCGATTAAATTCATAAATATGTTTCAACCATTCACCTCCTCTTTCTGTAAGGCAAGATTCAGTTTCATTCTTCCTTATTGTATAAATATTTCCTTCACAAAATCTGAAATAAATCCATAAATCCATCTTATCCATAATTAAAGAAAAAGTTTCACCTCGAGATTTTGTCAACAATTCGCAAGGTTGCGATGTTGCAATCAAATAGCCGAAAGACATTTTTTACCTCATACATGGTCGAAATCAGTTTCTGTTAGTTCAGAATCCATTTTTTCGTCAACAACTGAATCCGCATTTTTGAATTAACGTTTTCATCAGCTGCCGTTTATCTAAACCGGCAGGTTCAGTTTCAGAATAAGCCTTATATGAAGACTGTAAGCATTTCAGAAAAAGATCATCAGAAGGCATATCTGCCGAATCAAATACAACTGTTTTGATTTTGGTACTTACCCAAAACCCTTTTTGCTCTTTTTCTACTATACGGAAATTCAGAATATTTCCAACCGAATCAAAAGCACGGTAAACATCATCCATCAAATCCTGCGGCTCTATTTTCTTTTCCAATTCCGACAATCCTTGAAATATATCCAAAGACACATCTTCAAATAGAAAAAAAGGAGGAGTTAGAAGCGGTTTTTCCATGATCTGTCCGTAGATTTTGATTCCCAAGGGCGATGACGACCAATTCCCTGTCCAGGCAAAGTCTTGCCCGTATTATCCGTAACTCGACGATGATAATGGGGAAATTTTCCAATAGGATGACCTGTTCTATTACCGAAAGGGGCTATCCGCATATTATTGCCGATTTTAATCTCACGTCCATATTTAGCAAAGGAAACAACCTTTCCTGCAGCACCTACATCACCAGGAATTGCGCCTAATCCGCCAGCAACATCTCTAACAGAAGCTGGTCTGCCTGTCGTTGCATAACTAAAACCATGCTGTGTCCACATACCAATGGCAGCACCACCCAAGATAGCCAATGGAAGAAAAGCCCCTTCAGTCTCCTTCATCTCCTTCTGAGAAAGCTCCGCCAACTGCATCGGCGCATCTGCCCGCGTGTGGAACACTTGGTCTTCAAATGCCTGATTGTCCAATCCGTTTGCCATTGCGGGGGCGATCATAGACAGCATCATTACGGCTGCGGTGATTTGTTTTTTCATAATAACTCCTTTGGATTACAAGGTTGGAAAATCAAAATCTGCTTAAAATGTATGCTGTACGCCCAATTTCAGTTCGGAACTGCTTTGCCCTGAAACGTTGAAACGTGCGGATGCGTTTAAAGCCGCGGTTTTGGTGAAACCGAAACCTGCGCCGAAATGGGCGTAGGTGGATGTGTTTCTTGCGGATTCTTTTTTGCCGTCTATGCGGTCGGGCTGTTTGCCCAGCCATTGGATGCCTCCGGTCAGGCTGATTCTGTCGTTGGCGGCAAATGAGATGTTGGGATTCAGCATCCAGTAATTGCCTGCTTTGTATTTGACGTCGTCTGAAAGGGTTTTGCTGCCGTTGATGCGGTAGGCGGCGGTGAGGGAAAGGACAATCGGATCTATGGCTTTGTAGGTGGTGGCGCCGATGAGCCACGATTTTCCCGACGAGGCTTTGTTGCGCGATTTTTCGTAAACCGTGCTTTCGAGGAAAGCGATGAGTGCGGGATTTTTGCCGTCTTTGAGGAAGGTGTGGCTGATGCCGGCGGATATGTCGGACATCCGTTTGTTGCGGGTTTTGCCGTTGCCGTCGAGTTTGCGTTCTTCGTGCCACAGATAGCTGCCGCTGCCGTAAATGTCGGTATTGCCGGTCAGTCCGTAGCGCAAACCGAGCGTGCCGGCGAGCATATCGGTATTGCTGCCGTTTTCTTGAATTTCGGTCGGGACGGGGATAAAGGAAGCGGAGCCGGTCTGAATGTAAACCGGTGCGGCAAGTTCGGAACGGCTGTTTTCGCTGTTCAGGTAGGTAAGGGAAGTTTCCAGTTTCCATTTTCCCTTGTCGGTCATTATGTCTTCGATATTCAGCGGCAGATCAGTGCGGACACATAATGGCACAAAGCTAACAAATACTAAGAGAATATTTTTCATGACATTGTTTTCTTGATTGAAATCAAAGTCTACTGATGTTAATGAAATTCCATGTAATCTGTCTAAATAATATTTTTAACGATATTTTCAATACTGATTGTTTTTTTCTTATCAATATTTGTTTACTGCTTTATTGCACAAGAGAAGGCCGTCTGAAAACCTCGTATTTAATTTTCAGACGGCCTTTTGCCTTTTCAAATTCAAACCAATCAAACGGTTTTATTGCTTCATCGCGTTGGTCAAGGCTTTGACGTTGTGGCGGTACATGCCGATGTAGGTGTCTGCGGGCGCGTTGCCGAGTGCGTCGGAATACAGTTTGCCGCTGACGTTGACGCCGGTTTCTTTGGCGATGCGGTCAACCATGCGGGTGTCTTTGATATTTTCGGTAAATACGGCTTTGATGCCTTCGCGTTTGATTTGCCGGATGATGGCGGCGACTTGTTTGGCGGACGGCTCGGCTTCGCTGCTCACGCCTTGCGGGGCGATGAAGCTGATGTTGTAGCGGTTGCCCATGTAGGAAAATGCGTCGTGCCCGGTCAGGACTTTGCGTTTGGCGGCAGGGACGGCATTAAATGCGGCTTGTGCGTCGCTGTGCAGTTTTTTAAGCTGCATTTGGTAGTTGCCCAAGCGTTGTTGATAATAAACTTTGCCTTCGGGATCGGCCTTTATCAGGGCTTCAGCGACGTTTTGGGCATAGTCGGACATAAGAACAGGGTCGTTCCAGACGTGGGGGTCATATTCGCCGTGGTCGTGGTGGTGTCCTTCGTGGTCATGATCGTGGTCGTGATGGTGTCCGCCTTCTTCTTCGGCTTTGAGGGGTTGGATGCCTTTGGTCGCTTCGGCATAGGATACTTTGCTCTGTTTGACGGCGCGTTGGATGTCGGCGGCTTCAAGTCCCAAGCCGTTGAGCAGGACGAGTTTTGCACTGCGGATTTTTTTAATGTCGCCACTGGTCATGTGATAGGCATGAGTATCTTGGTTGGCTCCGACGAGGCTTTGTACGGCTACGCGCTCACCGCCGATTTGTTTGGCTACGTCGCCTAAAATGCTGAAACTGGTTACAACCGGAAGGGGTGCGGCAGTTGCGGCGGTGGCCAGCAATGCGGCAATAAGGGTAAGTTTGAGGTGTTTCATAACTGTTTTCCTGTGATATAACATAACATTCATTATGGTAAAACAAGCCGCCTGTTTGTTCAAGCGGCTTGCGGGGTCAGGTGGTGTGGTGGCGGTGGTTTTTGAACCATTTGGGCAAGATGCCGCCTTCTTTGCCGAGTATGACGGAAAAGAGATAAAGGACGCTGCAACAGAGGATGATGGCGGGGCCGGAAGGGATTTCGATGTGGTAGGAAATGAGCAGCCCGATCAAACCGCAAAAAAGGGCGATGAGGACGGACAACAGAATGAGCGTCCCCATATTTCTTGCCCATAAGCGGGCGGTAATGGCGGGCAGCATCATAAGTCCGACCGACATCAGGATGCCGAGAGCTTGGAAGCCGGATACGAGGTTCATAACGACGAGGATGAGGAAAACGACGTGCCAAAGCCCGCCTTTGCCGTTGACGGACTTGAGGAACAGGGGGTCTATGCTTTCTAGCACCAGGGGGCGGTAGATGACGGCAAGGGTAATGAGCGTGAGGCCGGAGACGGCGGCGATGAGTTGCAGTGCGGGAATATCGACGGCAAGCACAGATCCGAAAAGGAGGTGGAGTAAATCGACGCTGCTGCCGTTTTTGCTGATGAGGATTACGCCGATGGCGAGGCTGCTCAGGTAAAAGGCGGCAAAGTTGGCATCTTCTTTCAGGGTGGTAAAGCGGCTGACGAGTCCGGCAAGCAGCGCCATCAGCATACCGGCGGCAAACCCGCCCACACCCATAGCGGGCAGGCTCAAGCCGGCAAACATGTAGCCGACGGCGGCACCGGGCAGGACGGCGTGGCTCAATGCGTCGCCTATCAGGCTCATACGGCGCATGACGAGGAATACGCCGACGGGTGCGGCGCTGAGGGACAGGCAGAAGACGGATGCGAGGGCGTAGTGCATGAAGTCGAATTCTGCAAAGGGGGCAAGGAGCAGGTCGTAGAGGTTCATGGTGTCTGGTGTGGGGCGTGGTTTCGGGCAGTATTTATGAGGCGCACCAGTCGGGGCTTTCCTGTTGCTGCATTTTGGCGCTGGCTTGGGCGAGGTAGGATTCTGTCAGAATGGTCTCGGTTGCGCCTGCCGCAATTTTTTCGCGGGCGAGCAGCAGGGTATTGGGAAAGTAGGCACGGACTTGTTCGTAATCGTGCAAAACGGCAATGATAGCGTGTCCGCCGCAATGGCATTTTTGCAACACGTCGAGAAGCTCGTAGGTTGTCCGTGCATCAACAGCATTGAAAGGTTCGTCCAAAAGCAGGAATTTTGCATTTTGAACCAGCATCCGGGCGAAAAGGACGCGTTGGAATTGTCCGTTTGAGAGGTGGGCGATTTGGCGGCGGGCAAACTGCCGCATACCGACACGCTCCAAGGCTTCGTGAACGCGTTGTTTTTGAGCGGTATTTATCCCTTTGAAAAAACCGATTTCATACCACAGCCCCATTGCCGCCAAGTCGAAAACGGTCATAGGCTGGGAACGGTCGATGTCGGATTGTTGGGGGAGATAGGCAATGTTTTGACTGGTCAATCCGTCCAGCCGGATGCTGCCCGTATCGATAGGCTGCAATCCCATTAAGGATTTGAGAAAGGTAGATTTTCCTGCGCCGTTGGGACCGAAAACCGCCCACATACTATGTTCTTCAAAAGTAATGTCGACGTGGTGTACGGCGGGGCGGCGGCGGTAGCTGACCGTCAGGTTTTCAACGATGATGCTCATACCGATACCGCCCAAAGGTAAACACCCCATAAAAGGAATACGGCAATCAGGGCGAGGAAGAGTCGGAAGGTCAATCCTGACAGTAAGAGGGAAGGTGTCATAATGATTTGCAGTTTTGAAAGGGAAGTTGGCAAAGCGGTTATCGTTATATGGCGGATATGATACTGTATAACGTTTAGTCTGTAAATTCTGCTTGAATAGGCGGGAGTGATTGTTAATTAAGGTGGATGAGGGTCAGGCATATCGTTGCCTTGCCGGCATCGCGGCAATAAAAAATGCCGTCTGAACGTTCAGACGGCATTGGGGGAAAACGGTTTGAATCAACCTTTGCGTGCAGGCAGTTTTTCTTTGATGCGTGCAGCTTTGCCGGTCAGACCGCGCAGGTAGTACAGTTTGGCACGGCGTACGTCGCCACGGCGTTTGACTTCGATTTTCTCAACAGTAGGGGAATACAGTTGGAAAGTACGTTCAACACCTTCGCCGCTGGAGATTTTGCGGACGATGAAGTTGCTGTTCAAACCACGGTTGCGACGGGCGATAACCACGCCTTCGTAGGCTTGCAGACGGCTGCGGGTACCTTCCACGACGCGTACGGATACGACTACGGTGTCGCCCGGTGCGAATTCGGGGATTTCTTTGTTCAGGCGGGCAATTTCTTCTTGCTCGAGCTGTTGAATCAGGTTCATTGTTTTTTCCTAAATTATGATTGGATTTCCCGTTGCTCTTGTAGGATTTTATTCAAGAGGCGGGATTCCTTTGGGATTAAAACGCGCTTTTCCAAAAGATCGGGTCTGCGCTCCAAGGTGCGGCGCAGCGATTGTTCCAACCGCCATTCCGCTATCAAGCCATGATTTCCGGAGCGCAATACTTCCGGAACAGCCATGCCTTGAAATTCTAAGGGTTTGGTGTAGTGGGGGCAGTCCAAAATACCGCTTGAGAACGAATCCTGTTCGGCAGACTGAATGTCGCCCAATATGCCGGGTACGAGCCTCAATACCGCATCCATCAGCATCATGGCGGGAAGCTCTCCGCCGGAAACGACGAAGTCTCCGATGCTGATTTCTTCATCGACGCTGCTTTGCAGCAGTCTTTCGTCTATTCCCTCATAGCGTCCGCACAGCAGAATCAGATGCGTAAGTTCTGCCAGTTCTGCCGCTTTTTGGTGTGTCAGCGGTTTTCCTTGGGGGCTGAGGTAGATGACTTTTGCGGTTTGGGAAGATTGTGCTTTGGCGTGTTCTATCGCCGCATGAAGCGGCGGAGCCATCATAATCATTCCCGGACCGCCGCCGAACGGGCGGTCGTCGATATAGCCCAATCTGTTGTCGGCAAACTTTCGGGGATTGACTGCTTCAAACTGCCAGATTCCCTGTCTGTTCGCGCGTCCCGTTACGCCGTAGCGGGTAATGCTGTCGAACATTTCGGGGAAAATGGTAACTGCCTGGATAAGCATCAGTAGTCCAAACCCCAGTCGGCAGTAATGGTCTTGCTGCCGGTATCGACGGTTTCGATATATTGGGAAACGAACGGAATCAGAATCTGCCCGTGTTCTCCGTCAATCATCAATACGTCGTTTGCGCCGGTTTCCATCAGGTTGCTTACCTTGCCTAAAACGGTATCGTCTTTGTTGACGACGGTCATGCCGACCAAGTCTGCCCAGTAGTATTCGTCTTCTTCTGTCGGGGCGAATGCTTCACGGGGTATTTCGATGGTGTAACCGCGCAATGAGAATGCTGAATCGCGGTCGTCTATGCCTTCGAATTTGACTTGGAGTTCGCCGTTGACGACTTTTCCGGCTTCAAGGGTAACGCTGACGGTTTTGCCGTCCTTGGCCAAATGCCACTCGGGGTAGTCCAAAAGGCTGTCGGAATATTCGGTGTTGGCGGCAATTTTCAGCCAGCCTTTTATGCCGAATACGCCTTTGATGTAGCCCATGGCTACCCGGTTTTGAGTGTCTGTCATGGCGGCAAACGCGGATTAGACGGCTTTTTGTTCTTTAATCAGTTTTGCAACGGAGTCGCTGACTTGCGCGCCTTGTGCAATCCAGTGGTTCAGGCGGTCTGCATTGAGGCGGACGCGCTCTTGTTTTTCGTTGGCTACGGGGTTGTAGAAGCCTACGCGTTCGATGAAGCGGCCGTCGCGGCGGCTGCGTGAGTCAGTAACGATGACGTTGTAGAAGGGGCGGTGTTTCGAGCCGCCGCGTGCCAAACGGATAACTACCATTTTGAGTCCTTTTGAGAAAATCGGATATATGGAAACTGCCGATTTTAGGTTATTTTGTGGCCGGTGCGCAAGTTTTTATTTGTTTTTCCTGTTGTTTTGTCTGCCGCAAGGTTCAGATATGCGCGGTACAGGTTTTTTTCGGTGTCCGATTCCTTGAGGGTAAATCCTGATTTTTCAGCAAGTTTGATCATGGGGGTATTGGTTTTGAGAATGTCGGCACTCATGCTCAGGTAGCCTTGCTGTGCGGCGGTTTGGATGATGAGTTCCATCATTTTCTGTGCCAGCCCGCTGCCGCGCATATGTTCCGCCAGTGTGATGCCAAATTCGCATTCGTTGCGGTTCAGGCGGCTGTGGCGGACGACGGCGACGATGTTGCTGTCGGCATCCCTTGCCGTCCATGCGGTTCACAGTGGTAATCGGGGTTGCACAGGCGTGCCAACGTGGCTGCGGGCAGTTCGTTGGTGTGGGTCATGAAGCGTGTGTACCGTGCTTCGGGGCCGAGGCTGCGGACGAACTGCTGTTTGGCTTCTGCGTCTTCGGGTAAAAGGGGGATAATGGTAACGGTCGTGTTGTTTTTTAGGGACAGTGTTTTGGGGTGTGCTGCGGGATAGGGGGCAAGTACGTTGGGTACGGCTGCTCCGGTTTCGGTTTTGCTGCCGAGCAGTTCTGCGGCGGCTTCGCTTGTGTGGCGGAGAAATTCGGCGGCTGTCGGGTTTTTGTGTTCCAGGTATGCGGCGGCACTCTGCATTTTTGCGGCGGCATGTTCGAGGGTTTGGGCGGCTTTGCCTGTGTTCTTGCGTTTGGGCGTGTCGTGTGTTCCGGGTGTCTTTAAGATGAAATCGCTGCTGTATTGTCCGCCGTTGAGGTTGAGGGTGATGCCGAAAATGTGTTGGCGGTATTCGGGGATGACGGTCAGTGTGTGCAGGAACTGATCGAGGGTTTGTGTGCCGTCGAGTTCGGCAAAGCGGGCAAGGTGGCGGCTGTCGAGCGTGGTAAACGGCGGGAGTACGGCAGTGGTTTGTCCGTTGCAGCGTGCGGTCAGGATGTCGCCATAGAGGGGGTGGCTGCCGAATTGGAATTGGACGGCGTTATGGGTGGTGTGCCGGTAGGGGGAGGTGCAGGGCTTCGGCGAGCAGGGCGGGATTTGCCGCTGCAAGGGCTTTTTTGATGTTTTGGGGTTGCGGTGTTTTCAGACGGCATGGCTGCGGCGGTGCAATGTCGAGCTGTGCCTGTTTCAGGGCGGCGGCGGTGTTACGGTAGGAAAGGGTGCGGATTGCCTGTGCGGGGGTGTCGAAATGTGTTATGCCGTCTGAAAAGGGGCTGTTGACGAGCAGGGGTTTGGCGGTCTGTTCGGACAGGCGGATAAGGGCGCGTGCTGTTTTTTTGTAATCCTCGTGTCCGGAGGGACCGAGGATGGTCAGGACGGCTTGGGTGTCGGGGCGGGCAAGCTGACGTGAGGCGATGTCGTGGCAGATTGAGGGTGTGGGTGTGCCGGTCAGGTGTCCGTTGCGGATGTGGTGGGGAAGGTTGGGAAAGTGGAGGGTGAGGTTTTTTGGTGCGTGCGCGTGCAGCCATTCGGCAGGCGTGTCGGACAGGATGTCGAGTCGGGACAGGGGTGGAAGGTCGGACAGTTGGGCGCTCAGTGCGGCTTCGAGGTCGTCGGCGTTGAAACTGATGAGGAAGTTGCAGTGTCGGGCGAGGCAGTGCAGTACGGCACGGTCGGTTTCTGTCGTGAGGCAGGTGATGTGGAGAATCAGCGGCGTATGGCGGGTAAATTGGCGGATTGCGCTGAACAGCCTGCGCTGATCCTCTTCAGGGTTGTGGTGTAGGACGGCGGTTTTGGTGTGCAGGCTGTGTCCGAAGCGGTTGAGCCAATCGGCGGATGTGATGGGGCTGATGCCGGGATGCAGGTTGATGTGGCGGGATGTGCCTTGACGGAGTTTGTTCAGGATGTTGTCGATTTGGCGGCTGACGGCGGCATTGCCGGTCAGTATGGCGGTATGGCCTGCGGCATATCCGTCTTGGGTACTGATGTTGAGTCCGAGTGAGGGCAGTTGGATGCCTGCGGTGGTGTAGGCGGTGATGTTGAGTCCGTTGCCGTGGTGTTTGCGGATGGCAGTTTCGGCGGTGTGCAGTTCTGCGGCAGACAGGCTGTCCCAGTCCTGTATGAGGATGATGTGTCGGAGCTGCTTTTTACGGCAGGTTTTGAGGAGGGTGTCGTAACTGTCGGGCGGGGTAACGGTAATAATCAGGTCTGCACTGCCGGGGATTTTGTTGAGGCTGGTGTAGGCGGGAAGCCCGGCTATGGTGTGGTGGCGCGGGTTTACGGGGGTGATTTTTCCTTGAAAGGGCGTACTCAGCAGGTTGCTGAGTACGCGTTCACCCAGGCTGTACGGTTGTTCGCTCGCGCCTATCAGGATAATGTGGTTGGGCATGAAGAAGTAGCCCGGATCGGTTTGTGCCGGCATGATATATTCCTTTGCGGACGGTATGTGCGTGATTTTTGGAGATGCACCCGCTGTGTGTTTGTTTTGGGGTAACTGTTTGTGCAATGCCGTCTGAAGCGGGTTCAGACGGCATTATGGTCAGTTCGCACTTTTTTCTGTTTTGAAACCGGTTTTTTTCTTGGGCAGGATAAAGCGCATCCGCAGACCGTTCGGTTTGATGTTTTCGGCGATGATTTTGCCGCAGTGCTGTTCAATAATATGTTGGGTCAATGCAAGCCCCAGTCCTGTTCCGGGTTTGTTGGCGCTGGAGTCTGCACGGTAGAAAGCGGTGAAGATGTGCGGGAGCTGCATTTCGTCCACGCCGGGGCCGTTGTCGGTAACGTCGATTATCCAGTGTTTGTGGTCTTGTCCGATGTTGATCAGGATGGTGCTGCCTTCGGGACTGTAGTTGACGGCGTTGCGGATGACGTTGTCGAAGGCGCGGTACAGGTAGCTTTCGTTGGCAAGGATGGTTGTGTTTTCGGGGATTTTTCCGTCGGCAGACAGGGCGACCGTCTGTCCGTTCTTTTGGGCGATACTTTGGTTGTCTTCTACCAGGTTGCCCAGGAAGGGCAGGAGTTTCAGGCTTTCTTTTTCCAAAGCCATATTGGAGGTTTCGAGGCGGGACAGGGTTAACAGTTCCCCGGCCAGCGTATCCATGCGGGTCAGTTCGCCTTCCAGCCGTTTGAGATATTGCTCCCGTTTTTGGGGCTGTGCCTGAATCAGTCCGACAATTGCCTGCATACGGGCAAGCGGGGAGCGCATTTCGTGGGAAACGTGGTGGAGTAGGTAACGTTCTTTGGCGACAAGTTTTTCGAGTTTTTCTGCCATTTTGTCGAATTGGATGGCAAGATGGGACAATTCGTCGTCGCGGTCGTCGACCTGTTGGGAGATGCGGGTTTCAAGTTCTCCGTTTGCCACCCTGTCCATGCCGTTGCCTAAGATTCTGATGGGTTTGGCAATGTTGCCGGCAAGGATATATGCCATCAGCAGTCCGACAATGATGATGAAGGAGAGGATGATGAATTCGTGCCAAATCGGGGCAAGCGGCAGGCCCGGGATAAACAGCGGGCTGGGCAGGCGTTGTGCCTGGTGGTTGTCCCAGCCTTTAATGAAGAACAGGTATTCTTCGCCGAAACGGTCGTATTCGATGCGGACAAGGTTGGAATGGGGGTTGTTGGCGGCAAACAACCGGGCGCGTTCTATGGTGTAATTGTCGATATAGCGGTTTAAGATGTCTTTTTTCTCGTCGCCCTGTATGACGTAAACGGCGGATGAGACGGGGCTGTTTTTCCATTCGGTCAGGATTTCGCGCGCGCCGTTGTCGCCCCGTGTCTTGAATGCGGAAATAATGCTGCCCATCAATGTGGTTTCGATGGTGCGGCGTTGGTTGAATTGGTTTTCGGCAAGGGTGTTCTGCACCAGCCAAAAAGAAAAACTCGCCACAAAGATTGCGCAGACGATAACCGCGCAAAATGTGGCGAAAATGCGTTGGAACAGTTTCATTTGCCCGCTGCTTCAGTTTTTGACAAACAGGTAGCCCAAGCCGCGTACGGTTTGAATCAGGGAGGCATCGCCCAGCTTGTGGCGGATGCTGGAGATGTGTACGTCGATACTGCGGTCGAATTTTGCCAGTTTGCGGTCGAGTGCTTCGACGGACAGGGTTTCTTTGCTGACTACCTGTCCGGCATGGCGCATCAGGACTTCGAGCAGGTTGAATTCGGTGCTGGTCAGTTCGAGCGGCATGTCTTTGACGGATGCCTGGCGTTTGGCGGGGTATAGGACGACATCGCTGACGGAGATGCTGTTGGGTGCGTTGTTCTGTTCGCCGCTGTGTTGTGCTCGGCGGAGGATGGCATTGATGCGTGCCAAGAGTTCGCGTGGTGTGCAGGGTTTGGGGACATAGTCGTCCGCACCCATTTCCAAGCCGATGATTCGGTCGATGTCGTCGCCTTTGGCGGTCAGCATGATGATGGGGACGGTGCTTCGGGCGCGTACGTTTTTCAAGACATCCAAGCCGTTCATTTTGGGCATCATGGAATCCAATACGACTACATCGTATTGCCCGCTCAGGATTTCCTGTACGCCTGCTTCCCCGTCGGGAACGCTGCGGACGTTCAGACCTTCGGCGCTCAGGTATTCGGTCAGCAGTTCGGTCAGCAGGGCATCGTCATCTACGAGTAATACGCGGCTCATGGTGTTTCCTTTTCGTAAGGGTATGCCCCGACCCTGTTTCGGGCGTGGCGTGAAAAGATTGTTTGACAGTTTATCTTAACACGGCTGCAATGTTTTTTGATAGCGTATTTCCCTACCGGTTTGCTGTTTTTTGCAATGTCTTGCATGGAGCTTTACATTTCGGGCGGTATCCGCATCCGCCGACGCGGGTCATTTGCAGGGTTTTGTTTCCGGATGGCCGGGAGCGGCGGCGAAGGCTTTGCAGTCTTTGAGCAGTTCGGGCAGCAGCGGTGCCCATACGGGCAGTTTGCGGATTTCGTCGGCGTATCGGGGCATCAGGTAGGGGTAATAGGACTGTGTTGCCCGCATCCATTGTTTTGCTTCTGCAACTTTGCCTTGCCGCATCAAGTAGAGGGCGATGCGGTAGGTGGCGGAGTAGGGGCGGTATTTTAGTGCTTTGAGGGTTGCTTCTTCCGCCCAAGTCTGGGTTTCGGGGTATTCCGGCAGGGCGAAGTTTACGAGGGAGAAGTCGGCATAAAAGGACAGCATCGGGCTGTTTGCGGAAATATAGCGCAGTTCGTTGATTTTCCGGTTGAGGGTTTTGGCACTGTCGTCAGCGGCGGGGGAAAAGGAGTTAACCAGCCGGGTGTATGTCCAGTCCAAGTGCAGCAATCCTGCGAATATGGCGGCGGAGGCGGTCAGTATGCCGAGATTGGCGGCTTTTTTGAAGGCGATGCCGTCTGAAGCCTCTGCGGGGGACAGAAAGAGCATCAGTCCGAAGGGGATGAGGAAATAGACATACCACAAAGGATATTCGAGCATACTGTGGCACATACTGACGGCAAGCGCGCACAGCAGGAAAAGTGATGCGGGGGTCAGGGAGCGTTTCAGCAGCCCGGCAATGCCCGTCAGCAGGGTTGCGGCAACCAGAAGCGTGCCGCTGATCCCCATTTCTGCAAGGAGTTGGAGGATGATGTTGTGGGAATGGGTGAACAAGGTGCTGAGGAAGTTGTCGTGTATGGTGTGCTGTTCGGCATTGATCAGGAAGGTTTGTTGGGCAAAACTGTTCCAGCCGTGCCCGAATATCGGGGCGGACTGGAAGGCGGCAAGGGCTTTATTCCATTCGCTTTGGCGCGGCAAGTCTGTGAAACCGCCGTTGGCGACGCGTTCGACGGCAGTTTCGTAGCGGATGCCTGTAAAGGTTTCCAGAATGGCGTTCATGGAAAATTGGAACAGCGCGGTAAGGAATACGGCTGCGGCTATGCCGAGCATCGTCCGTCTGTTGGATTTGTCCGAACGGAAATACCAGAAGGGAAGGATGAGGGCGATGGCGGCTATGTAGGTCAAGATGGTGCGCGAATTGACCAAACCTAAAACGGCGGTCTGCATAATCAGGCAGATTGCGCCGAGGGCTGCGGGGATTTTTCGTTGTCCGTTGAGGTAGGCGGAGGCGAGTATGCCCCACATGAGGTAGTGTCCGAGGTTGTTGCGCTGCCCGATGTGTCCGATTACGCCTTGCCCTCTGTGAACGATGATGTTTTGAAGCAGGGGGGTGTTTTTCCAGCCGGCAAACTGGATGACGACGATGCAGGATTGAAGCAGGGAGCCGATAAGCAGCGACCAGGCAAACAGGGTAACGATGCGTTCTTGTCCGTAGTGTGCGACCAAACTCTTGCAGGCCCACGCGCTGACGGCGAGCAAGATGAAAACCCAAGAGGCGATGTCGTTCATTCCGGGATAAATCAGGTTCATCAGGCGTGCCTGAAGCCACCAAAATGCCGCCATTGCAAACAGGAGGAAGCTGATGGCGGGGATTTTGACATCAAACAGCTTTTTTCCTGCCGTGAGGAACAACAGGACAATCAGGCCGGCCGCGGCGGCGGCATCGTGGTAAAAGTCGGGCGACGGTTTCAGCCTGAGTGCGAAGGTAAAGGGGATGATGCCTATCCAAAGGAAGCAGGGCAGGATGTAAATCGGCAGTTTGGCGGCGGGGCGCGCGCCGGATACGGTCGTTTCAGCGGACATTGTTTGTTTCCTTGTATTGTTTGACGAACGACAGGCAGGATATGAAGAAGATGATGCTGAATACTGCGGAAAGCGCGGCGCAAATTTTTTCTGCGGGATAGGCGTCGAACAGGCGCATGACGGCTTCGGCAAAGTAAATCAGAACCAGCATGGAGCTGTATTGGTAGGTATAGATTTTTTTCTTTAGAATACCTGAAAGCGGCAGGCAGAGGGGCAGGGCTTTGAGCGCGAGCCACGAACCGCCCGGGCGTAACGGTGCAATCCATAATTCCCAAGAAAGGGATAGGGCAATCAGTGCAATCAGGCTGAAGGAGGCAAGGAAGTAAGCGGTTTGTCTGTTCACGGCGGACTTTACGGTTTAAGGGCGGATAAGGGGGGGCGGTATCCCAAATCCTGCAACATGGAAACGGTTTCATAAACGGGCAGCCCCATAATGCCGCTGAAGCTGCCTTCGATAGATTGGATAAAGATACCGCCTATGCCTTGCACGGCGTAGGCACCGGCTTTCTCCATCGGTTCGCCGCTTTGCACATAGGCGGAAATTTCTTCCGAACTCAGGGGCTTGAAAACGACGCGGTTGGTTTGGACGCGGCTTGACGTTTTGCCGCGATAATGAATGCAGACAGCAGTCAGGACGGTATGTTGTTTGCCGGACAATCGGTTTAAAAATTCGATTGCTTCGGCTTGGGAGCGAGGTTTGCCCAATATGATGCCGTCTGAAAAGACGCAGGTGTCGGCGGTAATCAGGGGGAAATCGGGCATTGCGCCGTTGGTTTCGCAAAAGAGGGTCAAAGCCGCCTGATTTTTTTCTTCCGCCATCCTTTGGACGTAAGGGGCGGGCGTTTCGCCGGCTTTAACGGTTTCGTCGATGCCGGCGGGCAGCTTGACGACCCGATAGCCCAACTGTGTCAGGATTTCCATCCGGCGCGGGCTGCCCGAACCTAAGTAAAGGGTGTTCACGGTGTGCTCCTTAATGTGCGGCGGGGCTTCAGACGGCATAGTGTCAGGTTTTTGCAGGCGGCTGTATGTCGCCATCCTGTTCTGAACGTGGAGTGAAGAAGCGTCCGAACCAAATGCCCGCCTCGTATAAGAGGATCAGCGGAATGGCAAGTAGGGTCTGAGAAATAATGTCGGGCGGCGTGATGACTGCGGCAACGACAAACGCGCCGACAATGACATAAGGCCGGGCGTGTTTGAGCTGCTCGGTCGTGACTGCGCCGATTTTGGTTAACAGGATGACGACGATGGGCACTTCAAATGCCGTGCCGAACGCAACGAACATACCTAAGATAAAGGACAGGTATTTGTCTATGTCGGTCGCCATATTGACCCCGACGGGGGTTACGCCGGCAAGGAATTTGAAAATGACGGGGAAAACCAGGAAATAGGCAAATGCCATGCCGATGAAAAACAGGCTGACGCTGGAGAGGACGAGCGGCGTAATCAGGCGTTTTTCGTTTTGGTAGAGTGCGGGGGCGACGAATGCCCAGATTTGGTAGAGCGTATGCGGCAGTGAAACCAAAAACGCCGCCATCAGGGTAACTTTGACCGGAACGAAAAACGGTGCGATGACATCGGTCGCAATCATACTGGTGTCTTTGGGCAGGTTTGCCATCAGCGGGTCGGCGATAAAAGTATAGAGCTGCTGGGCAAACGGCATCATGCCGAAAAAGCAGACGAGAATGCCGACAACCATCCACATCAGGCGGCGGCGTAGCTCGATGAGGTGTTCGACAAGCGGTTGGACGGTTTGTTCGTTTTGTGTTTCGGACACCGGATGCCCCCTTTATGATTTACGGACGCGCAATTTCGGTTTGGCGCGGTGTTTCGGACGAAAATCGCGTTTGCGGTTTATTGCCTGTTTGCGCAGGGAAGTGGTGTGCGGAACAGGCGTTTCAACAGCAGTATCGATATAGCTGACTTCGACGGCCTGTACGACAGGTGCGGCGGCAGAAGCAGTCAGGTATTCCCGCCATGCGCGGTCTTTGTCGGTTTCCGCAGGTTCGGCTGTACTGCCGGTTTGCCTGTCGTCCCCAAGGGTTTCGGCGGAAGTATCGGAACGTTCAGACGGCATAACGTCGGAAATGCCGTCTGATACGGTGTTTGCCGTATCGGGAAGGGGATTGCCGTTTTCATCGACACCGAAATCGGCAGGCGTGCGCTGTTCGGGCAGTTTTTCCCAAGGCTTCAGACCGTCGGAAATGTCGTGCAGACTGTTCTGCATATCCGTATCGGTTTCTTTGAGGCTGTCTCGAACCTGAGCGGCGGCAGCTTCGAATGCCTGCTTGACCTTCCTCAGCTCTTCCAGTTCGATTTGAGTGTCAAGTTCTTGTTTGACGCTTCCTACAAAGCGTTGCAGCCTGCCGATAAGCCGTCCGGCAGTGCGGGCGGCTTCGGGCAGGCGTTCTGGACCAAGGACAATCAGGGCGATAATGCCGACAAAAATCAGCTCGCCCAAACCGAAATCAAACATAAGTTATGCTTTGTCTTCGTCTTTTTTGTGTTCGATTACATCGTCTTTTTGGGCTTCTTTGCCGTCTGTGCCTTCGTTCAGCCCCTGTTTGAAGTCATGAACCGCACCGCCGAGGTCTTTGCCGACGTTGCGCAATTTTTTGGTGCCGAATATCAAAACGACGATAATCAGTACGATAATCCAGTGCGTCAGAGAAAAACTGCCCATGATGTATCCTTAAGTAAGTATTAGGGGTTGATTGTGAAATAACGGTTTATACGGGTGTGCCCATGATGTGTATATGCAGGTGGAAGACCTCTTGTCCGCCGCCTTTGCCGGTATTGATAAGGGTTTTGAAACCGTCGGTCAGTCCCGAAGCTTTGGCGATTTCGGGAACTTTCAGCATCATTTTTCCCAAAAGGGGCTGATGTTCGGGCGCGGCGTGTGCCAACGAATCAAAATGGACTTTGGGAATCAGCAGCAGATGAAGCGGAGCGGCGGGGTTGATGTCTTTGAAACAAACCATTTCGCCGTCTTCATAGACGGTTTGCGCCGGAATCTCTTTGGCGGCGATTTTGCAGAAAATACAGTTGTCCATAACGGCTCCGTTGCCGTCTGAAAAAAGCACGCAGACGGATTAAACGTGGAAGGGATGAGATTGTAATATAAATTCAGGATTCTGTACGGGCGGCTTTTTCGGCCAGCCCCGACAGCCCCTGACGGCGGGAAAGTTCGTCCAATACGTCTTCCGCCTTCAGGTCGTGGTGTGTCAGAAGAATCATGGTGTGGAACCATAAGTCGGCAACTTCGTAAACCAGGTGGGACGGGTCTTTGTCTTTGGATGCCATCAACACTTCGCCCGCTTCTTCAATCACTTTTTTTAGGATTTTGTCCTCGCCCTTGTGCAGGAGCTGTGCGACGTAAGATTCGGACGGGTCGGCAGATTTGCGCTGTATAATGGTTTGTTGGATGGCGGATAGTACGGAATCTCCCATGATTTTCCTTCTGTTTGTTTCTGTTTGTTCGGAATGATAGGCTAAACGGCTGCTCTCGGGCAATACGCCTGTTGCGTTTCGTTGGAAAATGCCGTCTGAGCGTTTCAGACGGCATTTGTGCTGTTGCAAATGTAATTTGCTTACAGGTTTGGATTCACAATAATTTTAACGGCGGATTCGTTGTTGTGGATCAGACGCTCGAAGCCTTCGGAAACCAGTTTGTCCAGCTTGATGCGCTGGGTGATGAAGGGTTCGAGGTTGATTTTGCCTTCTTCGACCAGTTTGATGGTTTCCGCGTGGTCGTTGCAGTAGGCAATCGTGCCGCGCACATCCAGTTCTTTCATCACGACGCTGTGGACGTTGACGGTGGCGGGGTGGCTCCAGATGGATACGATAACCAAGTTGGCGCCGGGTTTGCAGGCTTCGACCAAAGTATCCAACACTTTGTTCACGCTGGTACATTCGAACGCCACGTCCACGCCTTCGCCGTTGGTCAGTTTTTTCACTTCTTCAACAACATCGACTTCGGACGGGTCGAGGATGTAGTCGGCAACGCCGGATTCGCGGGCTTTGTCTTTGCGTGCTTTACTCAACTCGGTAATGATGACTTTGATGCCTTTGGCTTTCAACACGGCGGCCAACAGCAAACCGATCGGGCCTGCGCCGCCGACCAATGCGACATCGCCTGCTTTCGCGCCGCTGCGTACATAGGCGTGGTGTCCGACAGACAGAGGTTCAATCAAAGCAGCTTGATCCAACGGGATTTTGTCGGAAATCGGATGCACCCAACGGCGTTTGACGGCGATTTTTTCGGACAGGCCGCCGCCGCAGCCGCCCAAGCCGATAAAGTTCATGTCTTTGGAGAGGTGGTAGTTGCTGCCTTCGCCGGTCGGTACGTCGTCGCGGATGATGTAGGGTTCGACCACGACGTGTTGGCCGACTTTAATGTCGTCCACGCCTTCGCCGACGGCATAAACCACGCCGGAGAACTCGTGTCCCATCGTTACGGGCGCGGACTCGCCGGAAATCGGATGCGGATGACCGCAGGGCGGGATGAAAATCGGGCCTTCCATAAATTCGTGCAGGTCGGTACCGCAGATGCCGCACCAGGCGACATTGATGCCGACGGTGCCGGGGGCGACGGTCGGTTCGGGGATGTCTTCGATGCGGATGTCGCCTTTGTTGTAAAAACGTGCTGCTTTCATTGTAACGCTCCTTGTTTTCAAGTAGGAATGCCGTCTGAATCTAGCAGGCGGCGGTTGAAATGGGAATGGCGTGAAGAAGCCTGACCGTTTCCGATTGAATCTGTTTAGATATTTTACTACAAACTGAGACCTTTGCAAAATTCCCTTCCCTCCCGACATCCGAAACCCAAACACAGGTTTTCGGCTGTTTTCGCCCCAAATACC

>21 |ref|NC_017511.1| Neisseria gonorrhoeae TCDC-NG08107 | Coordinates: 173871,174936 | Forward

GCTTTCAACAGGTTCAAACACATCGCCTTCAGGTGGCTTTGCGCACCCACTTTGAGCAGCCCGAAATAGGCTGCCCGCGCATAGCGGAATTTACGGTAGCGGCATAAGGTGCTGCAACCGGGGATGCCCGGTTCGTCAAAACGGCAAAACAGGTTGAAACCGATGCGGGTGATGAGGCTGTGTTCGAGTTCGGGATCGGAGAGGCTGTGCCATTGTCCGGGCAGGACGGCTTTGAACATGGACGACAGGGGATGGGCGGGACGGCCGCGGCGGTCTCGGAGGTAACGGGTTTTTTGACGGATCAGGTATTGTTCGATCGGCTGCCAATCAATCACCTGGTCCAACTCCGATAGCGGGAAGCGGCCGATGTGTTTGGCAGTCATGGCTTGGCGGTTTGCCGGAAGAAGGTGTTCATGGGAAATCCCCTAAATGCCTTGGTGGGAATTTAGGGGATTTTAGGGGAATTTTGCAAAGGTCTTCGGGTTTGTATTATAAGATTTGGGAAGGTGGTTGGAGAATGCCCGCGCTGCCGTTTTTCAGAACATCTATCCCTATGCTTGTCCAAAATCGTATGGAGTCGATTTTCAACCACAACAAAGATGCCATTTTCTTGGAAGGATGGAGCTTGGGTGATGCCGCCATGATTATGGAACTTTTGTGGCAAAACATAAGCACTTCACGAAGAGAACTTACCAAACTGTTTTTATATAAAAACTTTTGGGGTTGTACTAGATAACCAGACCAAATTCCCATTAACTAATTGTCTTAAAATCTAAATTTGAGATTCTATTTCAAATGCCATTGGCATTTCTTTAAATATAGCCCCAAATGCTCTTTGGGAATGCCGTTAAACTTACGTAAATGGCTAAATTCACTAACATCAAGCACATCATAACTACGAAAGGTATCCGTGTATACAATGCCATCAGGCTTAACTTTCTTTCGGATAATTGGCAACAATGTCGCTGATTGCGCATTAGGGACAACGACGGTATAAACCTTATATATTGCGTCCCTAAGAAAGGGCATTAATTTTTGTTAATCGCCCCTTCTTAGGGACGCAATA

>22 |ref|NC_017511.1| Neisseria gonorrhoeae TCDC-NG08107 | Coordinates: 174937,183130 | Forward

ATTCGGCAAAGTTTGACAACGATTCAAAAGGTTTAGATCAGTTTTCGGACCGGTTGAAAAGCTTGGGATGTCAGAATCTGCATATCTGCATGGAGGCAACGGGAAACTATTATGAAGAAGTTGCCGACTACTTCGCGCAGTATTACAGCGTTTACGTAGTGAACCCGCTGAAAATAAGCAAGTATGCAGAAAGCAGGTTCAAGCGAACCAAAACAGACAAACAGGATGCAAAACTGATAGCGCAGTATTGCCGGTCGGCGCAGGAAAGCGAGCTTGTAAAGAGGCAGAAGCCTACGGACGAGCAATACAGGCTTTTACGGATGACCGCAGCATACGCGCAAATCAAAAGCGAATGCGCGGCAATGAAAAACCGTCATCACGCGGCAAAAGATGAAGAAGCGGCCAAAGCATATGCGCAAATCATCAAAGCCATGAATGAACAGCTTGAAGTTTTAAAGGAGAAGATAAAAGAGCAGACGGAGAAGCCTAACTGCAAGGAAGGCGTGAAGCGTCTTGAAACCATACCGGCAATAGGCAGAATGACCGCAGCCGTATTGTTTCATCATCTAACATCTTCGAAATTTGAAACATCAAACAAATTTGCAGCGTTCGCAGGCTTAAGCCCGCAACAAAAAGAATCCGGGACAAGCGTAAGGGGAAAAGGCAAACTGACCAAGTTTGGCAACAGGAAATTACGCGCCGTCTTGTTTATGCCGGCCATGGTCGCATACCGGATAAGGGCATTTCCCGACTTCATCAAAAGGCTGGAAGAAAAGAAGAAGCCTAAAAAAGTCATCATCGCAGCATTGATGCGTAAACTCGCCGTTATTGCGTATCACGTACATAAGAAAGGCGGAGATTACGATCCATCGCGTTACAAATCGGCGTAAATCCCGAAAGGAAAAAAGGCATTTTTTAAATGCCTGCTTTGCCGCGTCTGAAATCCGGTGAATTTTCAAATATTGAAATTCAATGGGTTGAAAATGAATTGTAAAGATGCTGTTGTCAATTAAAGTAGTATCTTGTCATTCCCGCAAAAGCGGGAATCCAGATCATTGGGTAGCGGCAATCTTCAAAAGTCGTCTGAAAAATCAGAAGTTCTAGATTCCCGTTTTCACGGGAATGACGGAATTTCAGACGGCATCCTCCCGCCCCGTCATTCCCGCGCAGGCGGGAATCTAGTCCGTTCGGTTTCAGTCATTTACGATAAATTCCTGTTGCCTTTCATTTCCGGATTCCCACTTTCGCGGGAATGACGGTTTGGAAGTTGCCTGAAACTTGAAAACAACTAAAACTGAACGAATCGGATTCCCACTTCCGTGGGAATGACAAACTTTAAGGTGTGATGACTTATCCAAAACAGTCGAAACGCAAAAACCGGTTTCTCGTTTGCATAAGAACGGCAAATTTTTCGGTGTCTTGTTTTATGGGCGTTATCCCTTGTCCGCTCCGTAGGGCAGGTAAACGTCGAAGCGGGTGCTTTTGCCGGTGTATTGGTAGGCGGGGGCTTGTCCGGCAAGGTGTTCGCCGAGGCGCGGGCGTTTGACGACGACGCGTTTTTTTGCCGTTTGGCGTGCGGTATGAAGGAGGATGACCTCATCTTGCGCTTCGCCGACAAGCCGGTGGAAATAAGCCATTTCTTTTTTGACGGCGGCACTTTTGCGGCGTTCGGGATACATCGGGTCGAGATAAACGATGTCGGGTTTGCCTTGTGTTTTAACAAGGGCAGGCATTTGTTCGGCGGCATTGCCGAAATGGAGGTTGATGCGCGCGGCGGTGTCTTGCGTTTCGGGATTGAGGAGGGCGCGGCGGATGCCGTCTGAAAGCAGGCAGGCGACGGCGGGATGTTGCTCGAAGGCGGTAACGGTCAGTCCGAGCGAGGCGAGGACGAAGCTGTCGCGCCCCAATCCTGCGGTTGCATCCCATACGGTGGGGTGCGCGGTGTGGTTGACGGCTTTGGCGATGAGTTCTCCTCCGCCTTTTGTGCGCCGGTATTGTGCCGCGCCGGAGGTAAAATCGACGATGACGTTGCTTTTTTCCCCGACAAGCCTGAGGCTGACGGTATCGTGTTCAGCAAGGAGGTAACTGCCTTGTTCGGGCGGTTGGGAAACGGGCACGAGGGGGAATGCCCGTATCAGGGTGCGGACGGCTTCGGTGGCGGTATCGTCAATGAGGATGTCGGTCATGGTGTATTGTGTCCGTTCGGTCGTGGGGCGAACCCGCTTCCCCGTGCCGTTTCAGACGGCATTTTTTCGGGTGGGCGGAGATGGTGCGGATGCCGTTTGTCAGTCTATGTTGAGGCGTTCCATACGGTAGCGCATAGAGCGGAAGCTGATGCCCAAGCGTTTGGCGGCTTGCGTGCGGTTGCCTTCGGTTTGTTTGAGGACTTGTCCGATGATGTCGCGTTCGATTTTGTCGAGATAGTCCTGTATCTGCATGGTATCGGGGTCGAACGGGAGAAGGCGGGACGGTGCGGCGGCTGTTTCAGACGGCAAGGTATCGGCAACGGGGACGGCGGTTTCCGTCCGGACGGGTTTGTGGTGCACATCTTGGATTTGCAGGTCGTCGATTTGCACTGTATATCCGACGCACAGGGCGACGGCGCGTTCGAGGATGTTTTCGAGTTCGCGGAAATTGCCCGGATAACTGTAATTCAGGAGCATCTGTTGCGCGGCGGGGGAGAGTGTGTAGGGCCGGTTGTTGTGGCTGTGTTTGTACAGGAGGTAGGGGGTGGGCAGCTTCAAATTTTCGCGCATTTCACGCAGGGACGGCATATTGAGGCTGACGACATTGAGACGGTAATACAGGTCTTGGCGGAATGCGCCGCTTTCGACAAGGGCTTCGAGGTTTTTGTGGGTGGCGCAGACGATGCGGACATCGACGGGCTGCTCGGTCGCGTCGCCGATACGGCGCACGGCTTTTTCTTGAATCGCGCGCAAGAGTTTGACCTGCATGGAAAGGGGCAGGTCGGCCACTTCGTCCAAAAATAAAGTGCCGCCGTCGGCGTGGCGGATGGCGGACATCGGGTTGCGGATTTCGTGGGCAAGGTTGGCGGTCAGTTGTCCGAGCGCGGCAAGTTTGACGGACAGGGCTTCCGCCTGAATTTCGCTTTGCGGGCGGATATAGAGGATGAGCAGCTTGTTCTGCTTTTTGTTCATCGGCACGGCGCGGATGCGGGCGGTCAGTTCGGGCGTGTCGATATAGTGTTCGAAGGTGCGTGAGGATGTTTTATCCCATAAGATGGCGACAGGGTCGAACAGGGAGGTATGCTGTCCGATTTCAAGCATGGGGAGCAGATCTTTTGCCTTTTTATTGAACAGTATGGTCTGATGCTCGACGTTGATGACGACGACCGCTTCCTGAACGCGGTTGAGCACGATTTGGTTCAAGCCCCTGATACGGCGGTAGGCGACGTGGTTTTCATGGGCAAGTTTGCCGGCACGGTCGATGTATCTGACCGACAGCGAGGCAATCATAGCGACGAAATAGGAGCCGGCTACGACGACGAAGGTATTGGTGACGGTTTTTGCATCCAGAATCAGCGGATACATATTGATATTGCTGTCTGCAAGGGCGTTGAATATCAGCAGGATGGATGCATAGCTGGCATAGAGCAGGGGATAACGCCCGTAACTGAGTAGGCAGGAGCTGCCCACGAAGGGCAGGATCAGGATGCCGAACCCCGAATCGATGCCGCCGAACAGGTAGGTCAGCACGCCGATCATCGTGATGTCGGCCACGGCGCTGAAACTCGGTATTCTCAAAGCCTGCCATTGCCATTGCGGGTTGAGGGTGGAAAAGAAAATCATCCAGCAGGCAATGGCAAGCCACAAGTAAAACGCCCACGCCGTCCATGAGGCGTGCAGGTTCGCACGGCTGCCGGTTTCCAGCCCGAGGATGTGCATAATCATCAGCGGGAAAACGATGGCGACGCGGATGATGTTGATCAGATTGGGAATCCGGTCTTTGAGTTTTTCAAGTTCGCGGGGGTTGGAAATCACCATAATATGTGCGGAACCGGGTTTATTTGGGGGAAGAAAGCTGTTTCAGGCTGCCCGATATGGGCAACAGTCTGCCTTTTTTGCCGCGTTTTGCCTCAATCAGGGCGACGGGGAGGCGGTCTTTGTGCGCCGCGCCGCGCCTGCCTTCGCTTTCAATCAGGATTTCCGGCTCGGAAGAAACGGCGGTATGCGTCATCGATTCGCCGGCGTTTAATCCGATGATTTGCAGTCCTTTGCCTTTCGCCATAATTTTCAATTCGCCGATGGGGAAGGCGAGGGCGCGGTTTTGACTGGTGGCTGCAATGATTTTGCAGTCGGGGTTGATGAACGAGGAGGCATAGACGGCAACCGGCGGCAGGACGGTTTCGCCGCTGTCTGCGGTCATCACCACTTTGCCCGCTTTCACGCGTCCGACCATATCGCCCAGCTTGGCGATAAAGCCGTAGCCGCCGCTGCTTGATAATAAATAATGTTGTTCCGGCAATCCTGTCAACATCGCGACGGGTTTCGCGCCGTTTTGCAACTCGATTAAGGAGGAAATCGGTACGCCGTCGCCGCGTCCGCCGGGGATTTCGGCGGCATCGATCGAGTAGGTTCTGCCCGATGAATCGAGGATGACGACGGGTAAAACAGTGCGGCCTTCAAGGGTTTGTTTGAGGCGGTCGCCTTCTTTGAACGCGGTTTGGCTCAAATCGAGATTATGTCCGGCACGGCTGCGTATCCAGCCTTTTTCCGACAAAATCAGCGTGATGGGTTCGTCGGCGGCGGTTTGTGTCAGCACGGCGCGTCCGGCCTCTTCCACCAGCGTGCGGCGCGCGTCGCCGAACTGCTTCATGTCCGCCTGCATCTCTTTGATAATCAGCTTGCGTTTTTCGTTTTCGTCGCCCAAAAAGATATTCAGACGGCCTTGTTCTTCGCGCAATTCGTTCAATTCTTTTTCGAGTTTGAAACCTTCCAAACGCGCCAGCTGACGCAGGCGGATTTCCAAAATGTCTTCGGCTTGGATTTCGGTCAGCCCGAACACCGCCATCAAATCGGCTTTCGGGTCGTCCGATTCGCGGATGACTTTAATCACTTCGTCGATGTGCAGAAAGACTTTCAGACGGCCTTCGAGGATGTGCAGCCGTTTTTCCACTTGGTTTAAACGGAATTTCAGACGGCGTGTTACGGTAACGACGCGGAAATCCAGCCATTCCTGCAAAATCGTTTTCAGGTTTTTCTGCGCGGGGCGGTTGTCCAAACCCATCATCACCAAGTTCATGGACACATTGCCTTCCAGCGAAGTTTGCGCCATCAGCGTGTTGATGAAGGTATCGGTATCGATGCGGCTGGATTTCGGTTCAAATACAAGGCGCACGGGATGTTCGCCGTCGGACTCGTCGCGCACGCGGTCGATTAAATCCAGCATCAGCTTTTTGGTATTGAGCCGGTCTTGGTTGAGCTGCTTTTTACCCGCTTTCGGTTTCGGGTTGGTTTGCTCTTCGATTTCGGCAAGGATTTTGGCGGAGTTGGCGTTCGGCGGCAGTTCGGTTACGATGACGCGCCACTGTCCGCGCGCCAATTTCTCGATTTCATAACGCGCACGCACGCGCACGCTGCCCTTGCCGGTTTCGTAAATACGGCGCAATTCGTCCGCCGGCGTGATGATTTGACCGCCGCCGGCAAAATCGGGAGCAGGAATATATTGCATCAGGTCGGCGGTTTCCAGCGTCGGTTTCTTCAACAGTGCAATCGCCGCCTGCGTGACTTCGTTCAAATTGTGCGACGGAATCTCGGTCGCCATACCCACCGCGATGCCCGACGCGCCGTTGAGCAACACCATAGGCAAGCGGGCGGGAAGGTGCAGCGGCTCGTCAAACGCGCCGTCGTAGTTCGGCATAAAATCCACCGTCCCCTGATTGATTTCGGACAATAGCAATTCCGCAATCGGCGTCAGCCGCGCTTCGGTGTAACGCATCGCCGCCGCCCCGTCGCCGTCGCGCGAACCGAAGTTGCCGATGCCGTCGATTAAGGGATAGCGCAAGGTAAAATCCTGAGCCATGCGCACCATCGCCTCATAGGCGGGACGGTCGCCGTGCGGATGGTATTTACCCAAAATCTCGCCGACCACGCGCGCCGATTTCACCGGCTTCGCCCCCGCCGTCAAACCCATATCGCGCATGGCAAACAAAATGCGCCGCTGCACGGGCTTTTGGCCGTCTGAAACTTCAGGCAGCGCGCGGCCTTTGACCACGCTCATGGCGTATTCGAGATAGGCGCGTTCGGCGTATCGGCCGAGCATCAGCGTGTTGGAATCGGTATGGGAAGCGTGCGGTTGCGTATTCATTGTGTATGCGGAATGTAAAGCAAAGGACATTATTGTAAATCAAAATGGCAAACCATAGTGGGCGCCGTGCCTGAATCTGAATCAAATACGGGAAATGTGAAAATATGTTATAAATAAAGCTTTCCCACTTTCACACATTGGAGACGATATGGAATTGGTATTTATCCGCCACGGACAAAGCGAATGGAACGCGAAAAACCTGTTTACAGGCTGGCGCGACGTGAAGCTGAGCGAGCAGGGGCTTGCCGAGGCCGCCGCCGCCGGTAAAAAACTGAAAGAAAACGGCTATGAGTTCGACATCGCCTTCACATCCGTCCTGACCCGCGCGATTAAGACCTGCAACATCGTTTTGGAAGAATCCGACCAACTGTTCGTACCGCAAATCAAAACATGGAGGCTGAACGAACGCCACTACGGCCGACTGCAAGGTTTGGACAAAAAACAAACCGCCGAAAAATACGGCGACGAGCAAGTCCGCATCTGGCGGCGCAGCTACGACACCCTGCCGCCGCTTTTGGACAAAGACGACGCGTTTTCCGCACACAAAGACCGTCGCTATGCCCATCTGCCTGCCGATGTCGTACCCGACGGCGAAAACCTGAAAGTAACGCTGGAGCGCGTATTACCGTTTTGGGAAGACCAAATCGCCCCCGCGATTTTGAGCGGCAAACGCGTCTTGGTAGCGGCGCACGGCAACTCCCTGCGCGCGCTGGCAAAACACATCGAGGGCATTTCCGACGAAGACATCATGGGCTTGGAAATCCCGACCGGCCAGCCGCTGGTGTACAAATTAGACGACAACCTGAAAGTCATCGAGAAATTTTACCTGTAAGGGTGCGAAATAAAAAATGCCGTCTGAGGGCTTGAGCATTTCAGACGGCATTTTTGACGGTAAACGTAGGGAGGGCTTCAGCCTGCCAATTAATAATCGAAAAAGAAGAAACAGTAACCGCAGGGTGGGCTTCAGTCCACTAATTGACAAGCAAAAATGAAGAAACGGTGGGCTGAAGCCCACCCTACGCAATTACGCAACTGTTATCGCGGGAATGACGGGATTTTAGGTTTTTGTTTTTCTGTTTTCGTGTGAAGGATGGGTTTTAGGTTGCGGTCATTTATCGGAAAAGCAGAAACTGCTCCGCTGTCATTCCCGCGAAGGCGGGAATCTGGAACCTCTGAATTTTCAGACAACCTTTGAATATTGCCGCCGTCCCGCGTTCTGGATTCCCGCCTGCGCGGGAATGACGATTCATCAGTTTCCCGAAAAAACCGAAATCCGACAGGCAGGATTTTTGCTTTCGCGGGGATGACGGGATTTTAAGTTACGGTCATTTATTGGAAAAGCAGAAACTGCTCCGCCGTTATTCCCGCGAAGGCAGGAATCTGAACACGTCCGTAGGGAAACCTATATCCCGTCATTCCCACGAAAGTGGGAATCCAGGATGCAGGGGAAACCGTTTTATCCGATAAGTTTTCGCACCGAAAGGTCTGGATTCCCGCCTTATATGATGCGCTCTACGCGGGAATGACGGGATTTTAGTAATCGTAGGGTGGGCTTTAGCCCACCAATCAACAACCCAAAATGAAGAAAAAACGGTGGGCTGAAGCCCACCCTGCGCAACTAAAAATTACCAACTACGCTTTTTCATTTATTCCTCCCAAACATGACCATCTATGAGGCGAACCTTGTTGCAATAGATATTTGCTAGAGATTGAGCAGCGGATAGTACTTCTACCTCAAGGCAATTCCATTCATATCCTTTTTCTAATGGTAAAACTACTAAAATAGTAGGGGCTGTACTAGATTATCCCTAAATTCCACACCGATCCCGCAGGATTTTTAGCTGCCGGGACGGTGTGCCGAAGTTAA

>23 |ref|NC_017511.1| Neisseria gonorrhoeae TCDC-NG08107 | Coordinates: 183131,192312 | Forward

TGTACTTCTTTCTTTAATTTGCAGTGTGTTATCTTCATATTTCGAGGGTAACATATCTGCTAATCTGGTACAGACCCGAATAGTGTCATGTTCTTCACTAAGAGATAATTTATCTTCATTATATGGATTACCGAGTTTTAAAAAATTTGCTGCTTTTTCTATAGCAACTTGCCAAGTTTTATATTTATCGTAATTTTTATCAAAACGTACTCCCCCTTGTTTATTGGATAAAAAAGTAATTATCTGATTCATATTAAAAATATTTCCATTATGGAAAACTCGTTTTAAAGTTAAAAATTTTTCAGCAGGACATAAAATTATATTTGTTTCAGCATAGATTATTGGTTCACCAGAAAATTCTTGTGATGAATGATATATAGGGCTAATAATTTTTCCGCCTAAATAAACTCCACCAGCCATATAGAGTGGATTAAATTTAAACCGGTACGGCGTTGCCTCGCCTTGCCGTACTATTTGTACTGTCTGCGGCTCGCCGCCTTGTCCTGATTTTTGTTAATCTACTATAAAAATTAACTTTATTCTCTAAGGTTGACAATTTTTCAAATACCAAACTTGTATCAAGGATGGGCAATTCTATAGTTAAACCTATTTCTTTTGCGAGAATATTTAATTGTTTGTCAATTAACCATCGACGGACAATAGGAGTTGCAATGCTACGTACATATCTTGGAGGAAGAGCATCTACACGAATTAGTGTTTCTTTTAAAGTTGATATATCCTCTTCTAAAAGTTTTTGTAACTCTGTTTTAGTATTCATTCTAACAATCCAAAATATAAGCCATTTGAACAAGACTCAAATGGCTTATTAAGATTTAAATTTAACTTAATTTCTGACAAAGCCAACCTTCCGCGCTTGCCAGCATTTCGGGCAATTTGTCGGCATCCGTACCGCCGGCTTGCGCCAAGTCGGGACGGCCGCCGCCTTTGCCGCCGACTTGTTCGGCTGCAAATTTAACCAGATCTCCTGCTTTGACTTTTCCGGTCAGGGCTTTGGATACGCCGGCGCACAGGGAGACTTTACCCTCGTTTACTGCCGCTAAAAGAATCACGGCGTTGTCGGATTTTCCGGTCAGGTCGGTTACGGTTTCGCGCAGGGCGGCTGCGTCGGCTTCGATTTGTGCGGCAACGAGTTTGGCCGCGCCCAGGTCTTTTGCGTCGTCCAAGAGTTTGGCGCCTGCGTGGACGGCGAGTTCGGCTTTGGCGCGTGCCAATTCTTTTTCCAATGCTTTGGCGTGTGCCGCGCCTGCTTGGATTTTCGCCAGTACGTCTTTTTCGGTTTGGGCTTTGGTTTCGGCAATGATGTCTTTTACCAAACGCTCTTGCTCTTGCGCCCATTTGAGTGCGTTCAGGCCGGTGATGGCTTCGATACGGCGTACGCCTGCGGCAATACCGCCTTCGCTGATGATTTTGAAGAGGCCGATGTCGCCGGTGCGTGAAACGTGTGTGCCGCCGCACAATTCGGTAGAGAAACCGCCCATTTGCAGTACGCGTACTTCGTCGCCGTATTTTTCGCCGAAGAGCATCATGGCGTCGGTTTTTTGCGCGTCTTCCATGCTCATAATGGCTGCGTTGACGGCAACGTTGGCTAAAACGGCTTCGTTGACGCGGCGTTCGACTTCAGCGATTTCTTCGGCGGTTACCGCTTGGGGATGGGAAATGTCGAAACGGGTGGATTCGGCGGTAACCAAAGAGCCTTTTTGTTCGACGTGTCCGCCCAATACGTCGCGCAGGGCTTTGTGCATCAGGTGGGTCGCGCTGTGGTTGCGCATATTGGCATTGCGGATTTCGTCATCCACTTTGGCGGTAACGCTGTCGCCGACTTTCAGACGGCCTGAAGTTTGTACGCCGAATTGTCCGAATACGGCCGCTTTGATTTTTTGGGTATCGCGTACTTCAAAGCGGTTTTCGCCTGCGAAGATATAGCCGACGTCGCCGACTTGGCCGCCGGATTCTGCATAGAACGGGGTAAAGTCGATAACGACGGCACCGCTGTCGCCTTCGTTCAATTCGACGACTTGCCCGCCGTCTTTGTAGAGGGCGAGGACTTTGGATTCGGTTTGGCGTTCGCTATAACCTTTAAACTCGGTGTCTTGACCGTCGTAAGGCAGTTGGGCGTTGGCTTTGAAGCTTTGGGCGGCGCGTGCGCGTGCGCGTTGGGCTTCCATTTCGCGCTCGAAGCCTGCTTCGTCCGGTTCGATATTGCGTTCGCGGCAGATGTCGGCAGTCAGGTCGTATGGGAAGCCGTAGGTATCGTAGAGTTTGAAGATAATTTCGCCGCCGAGTGTTTTGCCGCCTTTGGCCAGCGCGTTTTCCAACAAAGCCATACCGGTTTCCAGAGTTTGGGCAAAACGGCTTTCTTCGTTTTTCAAAGCTTCTTCGATTTGCGCCTGTTTTTCTTTCAATTCAGGATACGCGCCGCCCATCTCTTGAACCAAATCGGCAACGAGTTTGTGGAAGAACGGTTTGCTTTGACCCAGTTTGTAACCGTGGCGCACGGCGCGGCGGATGATGCGGCGCAATACGTAGCCGCGTCCTTCGTTGGAAGGCAATACGCCGTCTGCAATCAGGAATGAGCAGGAGCGGATGTGGTCGGCGATAACTTTCAGGCTGGGTTCTTCCATACTGAACGGCGCGCCGGTTTCGCGGGCAACGGCTTTGAGCAGGTCTTGGAACAGGTCGATTTCGTAGTTGCTGTGGACGTGCTGCATCACGGCGGCCATACGTTCCAAGCCCATACCGGTATCGACGGACGGCTTGGGCAGTGGATTCATATTGCCTTGTTCGTCGCGGTTGAACTGCATGAATACGCAGTTCCAAATTTCAATCCAGCGGTCGCCGTCTTCTTCGGGGCTGCCGGGAATGCCGCCCCAGATTTCTTTGCCGTGGTCGTAGAAAATTTCGGAGCAGGGGCCGCAAGGGCCGGTGTCGCCCATTTGCCAGAAGTTGTCGGACGCGTATTTCGCGCCTTTGTTGTCGCCGATGCGGACGATGCGTTCAGACGGCATACCGATTTCGTTCAACCAGATGTTGTAGGCTTCGTCGTCTTCTGCGTAAACGGTCGCCAAGAGTTTGTCTTTGGGGATGTTGAGCCATTCGGGGGAAGTGAGAAACTCCCAAGCGAAGTGGATCGCGTCGCGTTTGAAGTAATCGCCGAAGGAGAAGTTGCCCATCATTTCAAAGAAGGTGTGGTGGCGGGCGGTGTAGCCGACGTTTTCCAAGTCGTTGTGCTTGCCGCCTGCGCGTACACATTTTTGCGCGGTGGTGGCGCGGCTGTACGGGCGTTTGTCGAAACCTAAAAATACGTCTTTAAACTGGTTCATGCCTGCGTTGGTAAACAGCAGGGTCGGGTCGTCGTGCGGCACGAGGCTGGAAGAGCGGACGACGGTGTGGCCTTTGGTTTCAAAAAATTTTAGGAATTTTTGGCGCAGTTCGGAGGTTTTCATAATTTTTTCAATGTCTCTCAAATGTCTTGTCATGGTAAAAGCAGGGAAAACGAACGGCGGTATATTACCGCAAATCCCTGTTTCTAGCTATGGAAGCGGCGGCTTTCAGACGGCATTGCGGGATTTTGAATGCCGTCTGAAGCCCTGTTACCAATATCGGCTATAATGGCCGCTTTCTCCAACCCGATATGCAAGGAATGATAATGGTCAAACATCTGCCACTCGCCGTCCTGACTGCTTTGCTGCTTGCAGCGTGCGGCGGTTCGGACAAACCGCCTGCCGAAAAACCGGCACCGGCGGAAAACCAAAACGTATTGAAAATTTATAACTGGTCGGAATACGTCGATCCGGAAACCGTTGCCGATTTTGAAAAGAAAAACGGCATCAAGGTTACTTATGATGTGTACGACAGTGATGAAACGCTGGAAAGCAAGGTGCTGACCGGAAAATCCGGTTACGACATTGTCGCGCCGTCCAATGCGTTTGTGGGCAGGCAGATTAAGGCAGGTGCGTATCAGAAAATCGATAAGTCGATGATTCCCAATTATAAACATCTCAACCCTGAAATGATGAGGCTGATGGACGGGGTCGATCCCGACCACGAATACGCCGTGCCGTTTTATTGGGGGACAAATACCTTCGCCATCAATACCGAACGCGTGAAAAAGGCTTTGGGTACGGACAAGCTGCCGGACAACCAGTGGGATTTGGTGTTCAACCCCGAATACACGTTCAAACTCAAACAATGCGGCATCAGCTATTTGGACAGCGCGGCGGAAATTTATCCCATGGTGTTGAACTATTTGGGCAAAAACCCGAACAGCAGCAATACGGAAGACATCAGGGAGGCAACCGCCCTGCTCAAGAAAAACCGCCCCAATATCAAACGCTTTACTTCGTCCGGCTTTATCGATGATTTGGCGCGCGGCGATACCTGCGTAACAATCGGTTTCGGCGGAGATTTGAACATCGCCAAACGCCGTGCCGAAGAAGCGGGCGGCAAGGAAAAAATCCGCGTGATGATGCCGAAAGAGGGCGTGGGGATTTGGGTGGATTCTTTCGTGATTCCGAAAGATGCGAAAAACGTCGCCAACGCGCACAAATACATCAACGACTTCCTCGATCCGGAAGTGTCGGCGAAAAACGGCAATTTCGTTACCTACGCGCCTTCGAGCAAGCCGGCGCGCGATTTGATGGAGGACGAATTTAAAAACGACAATACGATTTTCCCGAGCGGGGAAGATTTGAAAAACAGCTTTATCATGGTGCCTATCCGGCCGGCGGCATTGAAGTTTATGGTGCGCCAGTGGCAGGATGTGAAGGCGGGGAAATAAAGCCCGATATGCCGTCTGAAGGATGTTCGGACGGCATTTTTTATCTTTGGCGGAAGAGGGCTTGCAGCCGCTGTTTGAAGGCAATGGGGCGGATGCTGCTCAAAATGCCGCTGAGGATAATGATGCACATACCGAGTATTTCCTGCCAGAAAAGCTCTTCGCCCAGAAAAAATGCGGCAGACAGGGCGGAAAAGACGACGGTCATATAGGAAAGCGAGGCAACCGTGAATTTGTCGCCGACTTTGTAGGCGCGCGTCATCGACAGTTGGGCAATCAGCGCGGACACGCCGATGCCCGACAGATAAACCGCCGATGGAAAGGACAGGGTGTGCCAGCCGGTCAGCGTCGCCCAAACCGACGACATCGCCACGCCGGTTGCGGAAAGGTAAAAGACGACGCGCCAGCCGGGTTCGCCCGCCAAAGACAGTTCGCGCACTTTCAAATACGCCCAGCCGGACATCGCGCCGCCCGCCAGCCCGGCGAGTGCCGCCGGTTCCTGACCGCTGCGGAACGAGGGATTAAGCAGCAATACCACGCCGGCAAAACCAAGGAGCAGCACCGCCTGCGTGTAAACGGAAATCCGTTCTTTCAAAATCAGGAAGGAAAATACCGCCAAAAAAATCGACGAGGTGTAACTCAGGGTAACGCCGGTTGTCAAAGGCAGATGCGTTACCGCGTAAAACAGCAGCAGCATCGCCCCCGTCCCGACCATACTGCGGTTTAAGTGGTTTTTCCAATGGGGCGTGCGGAAGGTGTCGCGCCGCAATACGGCGGCAGCACCGAGCGTAACGGTTGAAAACAGCATGCGCCAAAAGACCAATTCGCCGCTGCCGAGGGCAAATTTTGCCGATGCCTCTTTAATCAATACGTTCATAACGGTGAAGCAGGCCGCCGCCACCAGCATCCAGCCCGATCCTAAAATGTCTTTTTTTGCGGTATCCATAAACGGTCGTTGCGATAAGGACGGTCGGATTGTAAACCTTGCGGCAAGGCTTGTGGAATGTGTTTTTGCCTGCTTCTGATGCCGAAATTTTATTTTTCTTGCCGAACAATTTGTTTTCTCAAGGCAAACTTGATTATAATGGCGGGTATGAAAAAATACCTTATCCCTCTTTCCATTGCGGCAGTCCTTTCCGGCTGCCAGTCTATTTATGTGCCCACATTGACGGAAATCCCCGTGAATCCTATCAATACCGTCAAAACGGAAGCACCTGCAAAAGGTTTTCGCCTCGCCCCTTCGCATTGGGCGGATGTTGCCAAAATCAGCGATGAAGCGACGCGCTTGGGCTATCAGGTGGGTATCGGTAAAATGACCAAGGTTCAGGCGGCGCAATATCTGAACAACTTCAGAAAACGCCTGGTCGGACGCAATGCCGTCGATGACAGTATGTATGAAATCTACCTGCGTTCGGCGGTAGACAGCCAGCGCGGCGAAATCAATACGGAACAGTCCAAGCTGTATATCGAGAATGCCTTGCGCGGCTGGCAGCAGCGTTGGAAAAATATGGATGCCAAACCCGATAATCCCGCATTTACCAACTTTTTGATGGAAGTGATGAAGATGCAGCCCTTGAAATGACGCGGTACGCAAATGCCGTCTGAAAGCTTTTTCAGACGGCATTTGCGTTTGAAGCCCCGATTTATTTTTGCCCGCCTTCTTTCCGGTATTGCCCCGGCGAAACGTGATATTGCCGTTTGAACGCCTTGCCGAAATGCGTTTCCGACTGAAAGCCCACCGACAGCGCGACCTCCAAAACCGAATCCGGGGTTTTCTTCAGCAGCAATGCGCCTTTTTGCAGGCGGATATGGTTCACAAAGGCGTGCGGGCTGAGTCCGACTTGGCTTTTGAAGCGGCGCATCAGTTGCGCGCGCGACATATTGGCGGCGGCAACCATTTTGTCAATATTCCATTCGTCTTCCGGTTTGTCTATCACCTTTTGGATCAAATGTCCCAAACGTTTGTCCTGCCAACCTTTCAATACGCCCGAGAGTTCGACATCCTTATCCTGTTCGAGATAGGCGCGCAGGATAAGCACCAGCAGGACGGACGGTAATGCGTTGACCACGGAAACCGTCCCCGTCAAAGGTTTTTCGCTTTCCAGTTGCAGCATTGAAACCACATACTGCAAACTTGGATGGGCAATGTTCAGAAAAACGGTTTCCGGCAGCCCGTTCATCAAATCGGCGTGGGTGTCGTAGCGGAAACGGGCGCAAAACAGGCTCATATCCAGCCCGTTGCCGCACTGTTTGACCATAAATGTGCCGTTTTGCCGTATGTCCGGTTGTAAACTTTCTCCGTATTTTCCGTCGTGGCTCAACACATGACCCAAGCCGCGCGGGAAAAATACAATATCGCCCGTGCCGACCGGACGCGGGGAAGTTTCGCCGTCGATGCAGAGATAACCGCTGCCCGCCGTAACAATGTGTACCAGCCCTTCGCATTGCAAGGTTTCATGCCGTACCGACCATTGTCCGCCCAAAAGGCACTGCACATCCGCACTGCCCGTCAATTGGGCGAGATCGACCAGTTTGTCCAGAATGTCCATAAATCTTTTTGAACCATAAAATGAGATGATTAAACGAGAAACACAACTGAATAATTGCAATAATACGCACATCAAAAACAGATACGCAAGCGCGTATCAGGGTTAAACGCAAGAGAAAGGAAAAGAAAATGTTTAAAGATTGGAAAGAACATACCGCATTGGTTAAAAAATCATTCGGCGAGCTGGGTAAGGCGCATCCTAAAATGCTGCAAGCCTACGGCGCATTGGAACAGGCCGCCGCCGCCGAAGCACTCGATGCCAAAACGCGCGAACTGATTGCCATCGCCGTTGCCATCACCACCCGTTGCGAGAGCTGCATCAGCGTTCACGCCGCCGCCGCCGCCAAAGCCGGTGCGACCGACAGCGAGATCGCAGGTGCATTGGCAACCGCCATTGCCCTGAATGCGGGTGCTGCTTACACTTACGCCCTGCGTGCATTGGAAGCGGTTGAAACGCAAAAATAATCCGTTTCGGATAAGAAATGCCGTCTGAAAATATTTCAGACGGCATTTTGCCTGTTATGCTTCCCGATATTTTGCAGACAACATATGCAGCCGTTTTTTTCAGACGGCATTTGAGAAACATGGAACAAGTATGAAATATCACCGCTTGGCATTGTTTGCCGCCATAAGCTGCCTGTTGTTGTCCGCCGTTTTTATTGCACCTTACCTGACGGCATTTCACGAACAAGAAAAAATATTTGAATATGCGGATTTGACCGTTACCGCCCCCAACCGCAGCGGACGGGCAATCAAATTGGAGGCGGACGGGCGGCAGTACCGGCTTTCCTGCTACGGGTTCGACAGTTTGTGCACAGGCGGCAATATCGGCAGAGCCATCAGGGCGCGGCAGGTTAAGATTGTTTTAAGCGAAACCGTCGGCAAAGGTTTTTTAAACGGCGTTCTGTTGGAATACCGCAACAGTGGCAGTGTCTATAGCAATAAAGATTTTTCCCGCACGGAAGACCGCCTTGTCGAAGTGTTGGCACAACCTGCCGTTTTCAGCCTGAAACCGGGTATTTTGCTTTTATTGCCCGCCATTTTCCTGCGTTTGAAAAAAATGTGAAAACAGATTGGCGGGGCGGGGGAATGACTATGCGGCAATTTTACGTTTTGGGTTATGAGGATAACCGTTATAATCATAATTTTACAGTCCGCACAGAGAAAACCGATGCCTTGGAACATCCCCATCTTCCTCACATGGTTGAGGGTCTTGCTCATTCCCGTACTGATTGTCCTTTTCTACCTGCCGTTCTCATGGTTTTCAGAAGAAGCAGTCAATGTTGCCGCCGCCGTCATTTTTGCCGTTGCCGCCTTGACCGACTGGTTTGACGGATTTTTGGCAAGGTTGTGGAAACAGACTTCCGATTTCGGCGCATTCCTCGACCCCGTCGCCGACAAGCTGATGGTTGCCGTATCGCTGCTGCTGCTGGTCAAACTCGACCGGACCTATGTTTTGTTCGCGATGATTATCATCGGCAGGGAAATTACCATTTCCGCATTGCGCGAATGGATGGCGCAAATGGGAAAAAGGAGCAGCGTTGCCGTCGCCGCCGTCGGCAAGTTCAAAACCGCCGCGCAAATGCTGGCGATTTTCTTTTTGCTGCTGAATTTTCCCGATTTTTACGGATTTAATCTTGTTGTTATCGGCAACATATTGATGTTTATCGCATCTTTGCTGACAGTCTGGTCGATGCTGTATTATCTGAAAATGGCGTGGAAAGAAATTGCCTGAAAAAAACATAAAAATAGCTTGACGGTAAAAACATAATCCATAATAATTGCGTCTTCTTCGATGTCGGAGAGTGAAATCGGGCGGGAATAGCTCAGTTGGTAGAG

>24 |ref|NC_017511.1| Neisseria gonorrhoeae TCDC-NG08107 | Coordinates: 192313,230206 | Forward

ACTCGTTTCCCGCTCCAAGTTTTGTAGACAAGCCAGTTTTAGGGCGAGATAGCAAAGTGGTTATGCAGCGGATTGCAAATCCGTCTACGCCGGTTCGATTCCGACTCTCGCCTCCATATATCTTACGGCGGGGTGGCAGAGTGTTTATGCTATGAGGGGTGCAACCTTCATATAGGCCGGTTAAAATCCGCGCCCCCGCCTCCACCTTTCACAAATGCCCGGGTGGTGAAATAGGTAGACACAACGGACTTAAAATCCGTCGGGACTAAACATCCCATGCCGGTTCGATTCCGGCTCCGGGCACCAAGCTGAAAATGACAATGCCGCTCCAAGCGGTTATTTTTTTATCTGTCGGATGGGGATGGACAATATTGATATGTTCATGCCTGAACAAGAGGAAATCCAATCAATGTGGAAAGAAATTTTACTGAATTACGGTATTTTCCTGCTCGAACTGCTTACCGTGTTCGGCGCAATTGCGCTGATTGTGTTGGCTATCGTACAGAGTAAGAAACAGTCGGAAAGCGGCAGTGTCGTACTGACAGATTTTTCGGAAAATTATAAAAAACAGCGGCAATCGTTTGAAACATTCTTTTTAAGCGAGGAAGAGACAAAACATCAGGAAAAAAAAGAAAAGAAAAAGGAAAAGGCGGAAGCCAAAGCAGAGAAAAAGCGTTTGAAGGAGGGCGGGGAGAAATCTGCCGAAACGCAAAAATCCCGCCTTTTTGTGTTGGATTTTGACGGCGATTTGTATGCACACGCCGTAGAATCCTTGCGTCATGAGATTACGGCGGTGCTTTTGATTGCCAAGCCTGAAGATGAGGTTCTGCTCAGATTGGAAAGTCCGGGCGGCGTGGTTCACGGTTACGGTTTGGCGGCTTCGCAGCTTAGGCGTTTGCGCGAACGCAATATTCCGCTGACCGTCGCCGTCGATAAGGTCGCGGCAAGCGGCGGCTATATGATGGCGTGTGTGGCGGATAAAATTGTTTCCGCTCCGTTTGCGATTATCGGTTCGGTGGGTGTGGTGGCGGAAGTGCCGAATATCCACCGCCTGTTGAAAAAACATGATATTGATGTGGATGTGATGACGGCGGGCGAATTTAAGCGCACGGTTACTTTTATGGGTGAAAATACGGAAAAGGGCAAACAGAAATTCCGGCAGGAACTGGAGGAAACGCATCAGTTGTTCAAGCAGTTTGTCAGTGAAAACCGCCCCGGGTTGGATATTGAAAAAATAGCGACGGGCGAGCATTGGTTCGGCCGGCAGGCGTTGGCGTTGAACTTGATTGACGAGATTTCGACCAGTGATGATTTGTTGTTGAAAGCGTTTGAAAACAAACAGGTTATCGAAGTGAAATATCAGGAGAAGCGAAGCCTGATCCAGCGCATTGGTTTGCAGGCGGAAGCTTCCGTTGAAAAGTTGTTTGCCAAACTTGTCAACCGGCGAGCGGATGTGATGTAGTTTGCCGAACGGATGGAAATGCCGTCTGAAGTGTGTTCAGACGGCATTTTTCAAGTTCCGGCTTTGATGTTCCGTTCCGACCACTCTATCCGTTGTCCGATGTCTTTCAAATTGGAATATTGTTCGACCAATCGCGGCACATCGTCCAAACTCATGGCAAACATCCACAGATAAGTGCCGACCGAATAAATATGCCCCGCGCTGCCGTAGCCTTTGAGCGTCATCATCACAAAAGCAAAGCCGAACAAAATACCCATCGCCGCGCCGACGCAGAGATAGCCGAAGGCTTCGCGGTTGGAAATCAGCACACGCAGGCGCGAAACCAGTCCGTAATGGCGGTACAGCTGCCGCTCGTCGAAGTGGTTGTCGCGTTCCAAGCTGTTGTTCAGGCGGAAATACAGGTTTTCGCTGATGGCGGCAAAACGTGGCAAAAGCCATAAAAACAACGCAAGTATGCCCACCGCCGACACGCCGACCCAAAATTCCAGCACCAGCAGCATGATGCACGCGCCGAATATGGATACGACGGATGTCGCGGCAATCGGCAGGTGTTCTTCAAAAAAGCTGACAAATTCACGCGACAGGGCAACCCGTGCAGTTACCGCTGAATGCGGGACTTGCCGCTGCCGTTGTTCCAACACAACCGGCACGGCGATTTCGGTATAAATCCGCGTAAACGTGCGCGTATCGGCAATCCGCCGTGCCGCACCGACCAGCCACATCAAAAATACAACCAAAGCGTACAGCAACGCCTGCCACACCCTCCCCGCAATCACGGCATTAATCGCCCAGCCGCCAAACACCGGATACCCCAGCATCAAAAGGTTTTCCAGTCCGACCGGGGAAAATGTGCCAATCAATCGCTTGCGGTGGGTTTTGGCTATGTGTTTCAACATTTTCCACATATTTGTCCCTCTATTTTTTATTTTCCGAGATTGTTTTTTCCATCACTTCCGCCAGTGCATCCAAATCGGCAAACAGCCGGGTTGTGCGCTTGTCGCCGAATGTGGAAAATACTTTGTCGCTGAATTCCTGCGCGTTTTCTGTTAAAGGTGCGGCATGGACTTTGCCTTTTTCGGTCAGCGACAGCAACCGTTCGCGCCTGTCCTGTTCGCCTTCCTGCCATTCAATCAACCCTTGTCCGGCAAGGGTTTTGCATACGCCTGAAACGGTCTGTTTGGGCAGACTCCACTCTTCTCCGATGTGCTTTTGCGTGCGGCTGCCTTCGGTTGCCAGGGTATAAAGTACGGTAAAGAGGTTGTAATTCAAATCCTGCTGCCCGATCCATTTGTCAAAGACATTGCAAATCAGGTTGATACGGATTCCAAGTTGGTCGAGTCGGTTCATAATTGGTCTTGATATTGACTAAATTTGTGCGGATTATAGTGGATTAACAAAAACCGGTACGGCGTTGCCTCGCCTTGTCGTACTATTTGTACTGTCTGCGGCTTCGTCGCCTTGTCCTGATTTTTGTTAATCCACTATATAGTCATAATCGGGACTGATTTCAAGCGTGGGGCGATAAAAATGCCGTCTGAAACGCTTTCTGGCTTCAGACGGCATATTGTTTGGAAAGGGAAGTCAGTGTTCTGCCAATTTCAGGTAAACGCCGGAAACGTCCTGTTCGCCGTAGCCGGCTTCAACTGCTTTGCGGTAGCTGGCAGCAACGGTTTCGACGGCGGGCAGGGTGTTGCCTGCCTGTTCAAGCTCTTTGACGGCGAGGTTAAGGTCTTTGGAAGCGTGTTTGAGTGCAAAGGCAGGGGGGAACTCACGGTTTGCCCATAGTGATTTTTTTGTTTGAAACATAGGCGAGTCCATTGCCGAGCCGCCGATGGCTTCGACGATGGTGTCGGTATCGATGCCGAACTGCCGCGCCATCAGCATCGCTTCGCTGTACGCTTCGCCGAAAATGCCTAAGAGCGAGTTCAAGACGAGTTTCGCGCCCGAGCCTTTGCCGACATCGCCGAAATGGAAGGTTTTTTTGCCGACAAGGGAAAATATTTTTTGCAGCGGGTTTAAAACGGCTTCGCTGCCGCCGAACAGAATCAGCAGTGTGCCGTTGGTGGCGGGTCCGACCGATCCGGAAACGGGTGCTTCGGCAAACTGTCCGCCTGCGGCTTCGACAAGTGCTTTGACGGCGAGGTTTTCGGTCGGGGAGATGGTGCTCATGTTGACGATGATTTTGCCGGCCAATCCGTCGCGGACTCCGTTCAGGATGTCGCACACGGCGGCATAGTCGGAAACCATCAGGAAAATGACGGGACAGGCGCGGACGAGTTCGGCGGTGCTGCCGTAAACTTTTGCTCCTTTGGCGGAGATGGGGGCAGTTTTGTCGGGCGAGCGGTTGTATACGCCGACTTCGATGCCGCCGTCCAAGAGCCGCGTTACCATAGGCAGACCCATTTGCCCTAAGCCTACCCAGCCGATTTGTGTGTATGTTTCTGCGGACATAGTGTTTCTCCTTTGTTGGGGCGCTGTAATGCCGTCTGAAGGCTTCCTGCGCTTCAGACGGTCTGTTTGGGGATGTTATGCGGTCAGCGTGCCTTTGGTCGAGGGGGTTTGCCCAGCCATTCTCGGGTCGTGTTCGACTGCCATACGCAGGGCGCGCCCGAAGGCTTTGAATACGGTTTCCGCCTGATGGTGGGCGTTTTTGCCGCTGAGGTTGTCGATGTGCAGGGTCATCATGCTGTGGTTGACGATGCCGTGGAAAAATTCTTCAAACAAATCGACATCGAAACGTCCGATTAGTGCGCGGGTAAATTCGATGTTGTACACGAGTCCGGGGCGGCCGGAAAGGTCGATGACGACGCGGCTGAGGGCTTCGTCGAGCGGGACGTAGGAATGTCCGTAACGGCGGATGCCTTTTTTGTCGCCGAGTGCCTGCCGGATTGCTTGTCCGAGTGTGATGCCGATGTCTTCGGCGGTGTGGTGGTCGTCGATGTGCAGGTCGCCTTTGCAGCTGATGTCGATGTCAATCATGCCGTGGCGGGCGATTTGGTCGATCATGTGTTCGAGGAAGGGAACGCCGGTATCCAGCCTGCTTTTGCCGCTGCCGTCGAGGTTGATGGAGACGGTGATTTGGGTTTCGCAGGTATTGCGGTTGACGGTAACGCTGCGGCAGCCGGCAGCAGATGCGGTTTCGGCAATTCGGGTTTCGGCAGTTCCGGTGCTTTCTGCGGCGGTTTCGGGGACAGTGCGTTCGCGGTGTTTTCTGTCGAGCCAGCCTTTGGGTTTGCCGGTGTGTTTTTCGAGTTTTGCCATCAGGCTGGGACGGATGCCGCGCGCATCGGGGTCTTCTGCCTGCTTTTCAAGGCGTTGTTTGAGTTTGTAAAGTGCGACGGGGGTACGGTAGCCGCAGAGTTTGGCAAGCTTGGGCAGCGAACCTGCTTCTTGGGCGAGAGTCAGGAAGTTGTTCAGGTGGAGTTGTGTCTTGGTCATAGGGATTCCGTTTGGTTTATCGGTAGAGCCGGCGGATAACGTCGAGGACGGCATCGTTTTGTGCGGAGCTGCCTATGGTAATGCGCAGGCAGTGTTCCAAAAGCGGATGTGCGCCGTGCAGTTTTTTGACGAGGATGCGGTTTTGTTTGAGTGTGTCAAACAACAAATCGGCATCGGGTACGCGTATGGTAATGAAGTTTGCCTGACTTGGAAAGGCGTTCAGACGGCATATTTTGCCCAATTCGGCGAACATCCGTTCGCGTTCGTTTTTCAGGCTGTCGATGTTGGCAGAAATGATGCCGTAGTGTTGCAGGGCGAGTTTGGCGGTGGTCAGGCTCAGTTGGTTCATATTGTAGGGCGGCAGGATTTTTTGCAGTTCGCCGATGACTTCGGGACAGCCTGTCGCATAACCGATACGCAGTCCGGCAAAACCGATTTTGCTGAGGGTGCGTAAGACTATCAGGTTGGGAATCCTGCCCGCCTGCGGCAGGAAGCTGTCGCCGTTGAATGCGCCGTAGGCTTCGTCGACGACGACGATGCCGTCTGAAGCTTCGATGGCGGCTTCGATTTCGGCACGCGTGAAACATACGCCGGTCGGGTTGTTCGGGTAGGCGATAAAGGTCAGGGCAGGGCGGTGCTTCCTGACGGCTTCGAGGACGGCGGGCAGGTTGAGGGTGAAATCTCCGTTCAGTGGAACGCCGACATAATCCATGCCGTACAGCGCGGCGTTGTGGCGGTACATGATGAAACCGGGTTCGGCTGCCAACATTGCCGCGCCGGGTTTGGCGGTCAGCATGGTGATGAACTGTATCAGTTCGTCCGAACCGTTGCCTAAGGCGACGGCGGCGCAGTCAGGGATGTCGAACGCCGAACGCAATGCTTCCTGCAAACCGCAGCCGGAGGGGTTGGGATAGAGGTGGATGGGGGCGGATGCCAGTTGCGCCCGCCATTCCCGCATCAGGGCTTCGTGCCCCTCAAAAGGATGGGCGGGGCTTTCCATCGCATCGAGTTTGGCAAAACCGGGCGGAACATCGGTGATTTTGTATGCGGACATGGCGAGGATGTCGTTGCGGATGAAGGAGCGGACGGATTTCATCGTGTTTCCTTAATGGTTGGAATATGCCTGTACGCCGTTTCGGCATTATTTCATACGGAACTCTGCCGCGCGTGCGTGGGCGGTCAGGCTTTCGCCGTGTGCCAGCACGCTGGCGGTTTCGCCTAATTTTTGCGCGCCCTGTTCCGAAACCTGAATCAGGCTGGAGCGTTTTTGGAAATCATATGTCCCCAAAGGCGAGGAAAAGCGGGCGGTTCGGCTGGTGGGCAACACATGGTTCGGGCCGGCGCAGTAGTCGCCGAGGCTTTCGCCGGTGTAGCGTCCCATGAAAATCGCGCCGGCATGGCGGATTTTTTTCGCCCATTCCTGCGGGTTTTCGACTGACAGTTCCAAGTGTTCGGGGGAAATGTAGTTGGAGATTTCGCAGGCTTCGTTCAAGTCTTTAACGAGTATCATCGCGCCCCTGTTGCCGAGCGAGGCCTCGATGATGTCGCGGCGCGGCATGGTTTCGATGAGCCTGTCCATGGCGGCTTCTACTTCGTCGAGATACGCTTGCGACGTGCCGATGAGGATGGCTTGGGCAATTTCGTCGTGTTCGGCCTGGCTGAACAAATCCATCGCCACCCAATCGGCGGGTGTCGTGCCGTCGGCGATGACCAGTATTTCAGACGGCCCCGCCACCATGTCGATGCCGACCACGCCGAACACGCGGCGTTTGGCGGCGGCGACGAAGGCGTTGCCCGGACCGGTGATTTTATCGACTTGGGGGATGGTTTCCGTGCCGTAGGCGAGGGCGGCGATGGCTTGCGCGCCGCCGACGGTGAAGACTTTGGTTACGCCGGCGACGTATGCGGCGGCAAGCACGATGTCGTTGCGTTCGCCTTTCGGTGTCGGTACGACCATAATGATTTCTTTCACGCCTGCGACGTGGGCGGGCATCGCGTTCATGATGACGGAACTCGGATATGCCGCCTTGCCGCCTGGGACGTAAATGCCGACGCGGTCAAGCGGGGTAATCTGTTGCCCCAGCAGCGTGCCGTCTTCATCGGTGTAGCTCCACGATTCCATTTTTTGGCGTTGGTGGTAGCTTTCGACACGGCGGGCGGCGGTCTGCAATGCTGTCTGAACGTCGTTCGGAATGCGCTCGAACGCCGCGTTCAAATCGGCTTGCGTGAGTATTAAATCATCGATGCTTTTAGCGTTTGTCTGATCGAATTTGTTGGTGTATTCAATCAAAGCCGCATCGCCGCGCTTTTGCACGTCGGCGCAAATGTCGGCGACGATGCGTTCGGTTTCGGGGTTTTGCGCGGTTTCAAAAGCCAGCAGGGCTTTGAGTCCGGCTTGGAAATCGGGCGATTGGGTGTTGAGTTTTTTCATGATTTGGGATTCTCCTTTGTTATTTTGGGACTTGGGATCGTCTGAAGCGTCTCTTGCTCGGTTTTGGGTCTGATATACGCTTTTCCTCCCCCGCTACCAAACTTCAATTCGGGCAGGCATTTTTGTTGCAGACCGAGTTTGGCTAATACCTCTTCGCTACGAGAGGTAGTACGGAAACGGTAACGGGTTTGTTTTCTGCCGTTTTTGGTTTCCTCAGTCATTGTTTCAAAACCGTCCACTTCGTACGCACTGACAACAGCGTTTTGCAGACCGGTGTGAACGCCGATAACGTATTTCACTTTTGAAGCAACATCTTTACCGATAACCCAGTTGCCCAACGTACGCGATTTGAGGTTGGCATCGTCTTGGTTGTCGAAAAGGTAGTCTAATTCTTCGTCAGTATCTAAATCGAAAGCATTGTGGATCTTGATGGCGAGAATCAGCCCGTCGGGGTTAATCTCGCTAAGTGGGAGAGAAGAGAATCCAAAGCGGCGATCTAGTTCTTCTACGCTAATACCACCCGGACCATGCCCGGCAATTTTATTTTTGAGTTTTTTACCCAAGACAGATTTAACAAAATGAATTAAGGCAGATTCGGCGGCGAGCGCTTCGACTTCAGTCAGATGATAGCTGATGATATAGCGACCGAGTTTCTTGCATTTGGAGATGGCTTTGAGTTTCCGATCGATAATCTCGCCGGAGACTGGGTCTTGTGAACGACTAGCAACCCATTCATGCTCGAAGATACGATTACCGCAACCTTTTCCGATGTAGAGGATTTTGTCTTTTTTCAGGTCGGTCAGACAATAGACATAAAACCGACGCTCGCCGTTATTCAAAACGGAAAGGGTCGAATCTGAGAATTTTTTTATTTTTGCAACCATTTTAACTTCTCCTATACAGTTTACTGCCGGTGATATTTTCAGACGGCCTGTCGGCGGGAACGGATAGGGTCGTCTGAAAACGCATCTTTATTCAAATGAATGCCCACTTCGCCTTCACTGCGCCGCCGAACGACTGAATAATCGGCTCCAGCAGCGCGTATTTCGTTTTCAAAGCAGCCTTGTTGACCACCAGGTAGCTGGAAATGTCGACGATGTGTTCGACTGCCTCCAAGCCGTTTGCCTTCAAGGTGTTTCCCGTCGAAACCAAGTCCACAATCGCATCGCTCAAGCCGACCAGCGGCGCAAGTTCCATCGAGCCGTACAGTTTGATAATGTCCACATGGACACCCTTGCCGGCAAAATGAGATGCCGCGATTTCAGGATACTTCGTGGCAATCTTCAGACGGCATCCGGGTTGCGAAGCGGCTTCGTAATCAAACCCTTTACGCACAGCAACCATCATGCGGCACTTGGCAATCTCCAAATCCAAAGGCCGGTAAAGCCCCGTGCCGCCGTGTTCGATCAGCACGTCTTTGCCCGCAATGCCGAAGTCCGCCGCGCCGTAGCGGACATAAGTCGGCACATCGGTTGCGCGGACAATGACAAGGCGGATGTTTTCATGGTTCGTCCCGATAATCAGCTTGCGCGATTTTTCGGGCTCTTCAGTCGGAGCAATGCCGGCAGCGGCAAGCAGCGGCAGCGTCTCCTCAAAAATGCGCCCTTTGGATAAGGCGATGGTCAAAGCATTATCCTGCATGGTGTGGATGTCCTTTGTCGGTTGAAACAATAATGCCGTCTGAACAGGCGCCGGCGGTGCAGACGGCATACGGTTTTGTCAGAGCAGGGTCCTTACATCGGCAGCAATCGTTTCCGGCTCGCTTCCGTAAGGCGAGAAAATGGCAACCTCACCGTTTTTGTCGATGAGATACGCACCGGAAGAGTGGTCGACCAAATAGTTTTCGCTGTCGTCTTTTTGATTGATTTTGGCAGAAACCACGCGGTATTGCTGCTTGATGACCGGCAGGTTTTGACCGCCCGTTGCCGTCAGACCGATAAAGTCCGGATTGAACTGTTTGGCATACTTGCCGATGATTTCAGGCGTGTCGCGTTCCGGATCGATGCTGACGAACACCACTTTCACGTCCTTAGCCTGCCCGCCCAACTGCTTCAAAGTGTCGCTGTACGTCAAAAGCTCTGTCGGGCAGACATCGGGACAGTGCGTAAAGCCGAAAGACAGAATCACGACCTTGCCTTTCAAATCGCTCAGGCTGAAAGGCTTGCCTTCGCCGTCGGTCAGCGTGAAATCGCCGCCGATGTCTTCCTTACGCATATCCGTACCGCGCGTTTGCGGCTTTGCCGCATTTTCCGCAGCCGGCGCGGATGCACTTGAAGAAGCGGCTTGCGCCGCACTGTTGTCTTGAGGTTTGCAGGCGGCAAGCGCGGCAAGTACGAAAACGCCCGGCAAAAAGGAACGCGGTACGGAAAACATGACAATCTCCCAAGAATAAATAGAAAAACCTGATTCGTACGGAATTTGAATATTTCCATCTTAACGCGTTTGGCAGATTTAATGTAGTATTTTTTTACGTTGTCCGTACGGCAGGAATTGCCGAATCATACAGAATCTTTGGAGCGGAAAGAACAGTAAGATTAAAATAATAAAAATTCTATTTAAAATCAAAAACAAAATAAAAACAGATAAAATAATTTCAAAAAAAGGTTTACAGAATCTCAAAATCCATTATAATGGCAATTTCTTCACCAGCCCAGGTGGCGAAATTGGTAGACGCAGGGGACTCAAAATCCCCCGCCGCAAGGTGTGTCGGTTCGAGTCCGACCCTGGGCACCACAACCGCTTTTGGAGCGGTTATTTTTTTGTCTATTGCTGTTTAATGAAATAGATAAGACTAAATATAGTGGATTAAATTTAAACCGGTACGGCGTTGCCTCGCCTTGCCGTACCATCTGTACTGTCTGCGGCTTCGTCGTTTTGTCCTGATTTAAATTTAATCCACTATACAATCAATATATTTAAGTCGTTGTTAGCCCTCTTGGTTCTGTCAATGCCGTCCGGTGCAATGAGCCATTTTCAGGAAGTATGGTTGGGAATATTTTTGATATATCAAAAATAAAATCAAGTTCAATCAATATCCCGAGTGAAACCCTGTAAATTTTAAAATTCTTGATACTTTCACATCTGCCCCCATGCAAACTGTCAACAATTTCCATCTTTCAAAAAAGGTACTCCTGATTTTATTCTTTTAAAATCAATCAATAATTTCTCCGCTTTCAAAAAAATTTCTCCGCTTTCAAAAAATATTAAAAGATGAAAGATTTTTCTTGATTGAACTGTCGGTTTGCGTTATCGTTTTTACTCTTTTTCACAAACTCTGTGTTCCTTTCCAATTGATTGGATAATCTGTCTGTTGCCGTGTTCCCCCAATGCGCGTACCCTAAATCGCTGCTTGGTGGAATTGCATTACAGGTGCTGTGGCAAAGCGGTTTGTCCCTATTGGTTTGAAACCGTATAAAAGAGGTCGTTATGCAGTTATCAGGCGCGCAAATCATAGTGCAGAGTCTCAAAGCCGAAGGCGTCGAGTACGTTTTCGGTTATCCCGGCGGTGCGGTTATCGAAATCTACGATGCCCTGTTCCAACTCAATAAATTCAAACACATTCTTACCCGCCATGAGCAGGCGGCAGTACACGCGGCAGATGCGTATGCGCGCGTCAGCGGTAAGGTGGGCGTGGCATTGGTTACATCCGGCCCGGGCGTTACCAATGCGCTGACCGGTATTGCTACTGCCTATACGGATTCGATTCCGATGGTGGTCATCAGCGGACAGGTAGGCAATTCCCTGATTGGTACGGATGCGTTCCAAGAAGTTGATACGGTGGGTATTACCCGTCCGTGCGTCAAACACAATTTCTTGGTTACGGACATCAATGAGTTGGTGGAAACCATTAAAAAGGCGTTCCAAATTGCCGCAAGCGGCCGACCGGGGCCCGTGGTGCTTGATGTCCCGAAAGATGTTACGCAGGCGATGGCGAAATTCAGCTATCCTCAGGAAGACATTTTTATCCGTTCGTATCAACCGGTTGTTCAAGGGCATATCGGTCAGATTAAAAAGGCCGTGCAGATGTTGGCATCTGCCAAACGCCCGGTCGTCTATTTTGGCGGCGGCGTGGTGTTGGGTAATGCTTCTGAAGAGCTGACCCGATTTGTCCGAATGACGGGTGCTCCGTGCACGGGTACGCTGATGGGCTTGGGCGCTTATCCTTCCGGCGACCGCCAATTCCTCGGTATGCTCGGTATGCACGGTACTTACGAGGCAAACCTTGCCATGCAGAATGCGGATGTCGTTCTTGCCGTAGGTGCGCGTTTTGACGACCGTGTGGTTTCCGTACCGTCCAAATTTTTCGAGAAGGCGAAGAAGGTTATCCATATCGATGTCGATCCTTCCAGCATCGCCAAACGCGTGAAGGCGGACATTCCGATTGTCGGCGACGTGAAAAACATTTTGTCCGAGATGGTTGCGCTGTGGCAAAAACAAGAGTCCGTGCCGTCTGAAGATGCTTTGGGCAAATGGTGGAAAACCATAGAGGAATGGCGTTCCCGCGATTGCTTGTGGTTTGACAACGGCAGCGAAATTATCAAGCCCCAATATGTGATTCAGAAGCTTGCCGAGATTACCGGCAATTCGGCAATCATCACATCGGATGTAGGGCAGCATCAAATGTTTGCGGCTCAATATTATCCTTTCGAACGTCCGCGCCAATGGCTCAACTCCGGCGGTTTGGGTACGATGGGCGTGGGTCTGCCTTATGCGATTGGTGCAAAACTTGCCGCCCCGGATCAAGACGTATTCTGTATTACCGGCGACGGCTCGATCCAGATGAACATCCAAGAGTTGTCCACCTGTTTCCAATATCGGATTCCGGTTAACGTCATTACGCTGAACAACGGTTATCTGGGTATGGTACGCCAGTGGCAGGAAATATATTACGGCGGTCGAGAGTCGGAAACCTATTTCGATTCTTTGCCCGATTTCGTCAAACTTGCCGAGGCATACGGCCATATCGGCATCCGCGTGGACAAGAAATCTGATGTGGAAGGTGCCTTGTTGGAAGCATTGAACCAAAAAGACAGGCTGGTGTTTATCGACTTCCTGACCGACCAGAAACAGAATGTGATGCCTATGGTCGGCAACGGCAAAGGTTTGGACGAAATGGTACTTCCGCCGCATATGCGTACGGACGGAAAGGCGTAAGGAGAGGCAAATGCGACATATCTTATCTGTTCTGATCGAAAACGAATCAGGTGCGATGAGCCGCGTGGTCGGTTTGTTCTCTGCACGCGATTACAATATCGATTCTTTGGCGGTTGCGCCGACCGAAGACAAAACCCTGTCACGGATGACCATCGTTACCCACGGCGACGAGCAGGTTATCGAACAAATCACCAAGCAACTCAATAAATTGATTGAAGTGATTAAAGTGGTCGATTTGAACGAAAGCCGTTTTGTCGAACGCGAACTGATGTTGGTAAAAGTCCGTGCCGCCGGCAAAGACCGCGACGAATTTTTACGCTTGACCGAAATCTACCGGGGCAGCATCATCGACGTAACCGACCGCAGCTATACGATTGAAATTACCGGCTCGACCGACAAGCTGGATTCCTTTTTGGAAACGGTCGGACGCGCCCAAATTTTGGAAACCGTACGTACAGGTGCGGCCGGCATCGGTCGCGGTGAGCGTATTTTGAAAATTTAACGCCGTAACCTTTCAGACGGCATGGTATTTGAATGCCGTCTGAAAAACGAACGGCAGGAGAGATTTATGTCGAACATTAAAATTGTCGCACTGGTTACCGTCAAACCGGAATACACGGAAACACTGGCAGCACAGTTTAAAGAACTGGTCAAAGCCAGCCGTGCGGAAGAGGGCAACATCAGCTACAATCTCCATCAGGAAATCGGCAAACCGAACCGTTTTGTTTTCGTGGAAAATTGGAAATCCCAAGCAGCTATTGACGAACATAATGCCAGCGCGCATTTCCAAGCCTTCGTCCAGTCCGTCGACGGCAAAACCGAAGCGTTGGAAATCGTATTGATGAATGAAGTTGCCGTTTAAGCGGCACACTCTGTTTAACCCGTCCGAAGCCGCTCAACATTTTAAGGCTTCGACAACCATTTACCTTAAAGGAAATCAAATGCAAGTCTATTACGATAAAGATGCCGATCTGTCCCTGATCAAAGGCAAAACCGTTGCCATCATCGGTTACGGTTCGCAAGGTCATGCCCATGCTGCCAACCTGAAAGATTCGGGTGTAAACGTGGTGATCGGTCTGCGCCACGGCTCTTCTTGGAAAAAAGCCGAAGCAGCCGGCCATGTTGTTAAAACCGTTGCTGAAGCGACCAAAGAAGCCGATGTCGTTATGCTGCTGCTGCCTGACGAAACCATGCCTGCCGTCTATCACGCCGAAGTTGCAGCCAATTTGAAAGAAGGCGCGACGCTGGCATTTGCACACGGCTTCAACGTGCACTACAACCAAATCGTTCCGCGTGCCGACTTGGACGTGATTATGGTTGCCCCCAAAGGTCCGGGTCATACCGTACGCAGTGAATACAAACGCGGCGGCGGCGTGCCTTCTCTGATTGCCGTTTACCAAGACAATTCCGGCAAGGCCAAAGACATCGCCCTGTCTTATGCGGCTGCCAACGGCGGCACCAAAGGCGGTGTGATTGAAACCACTTTCCGCGAAGAAACCGAAACCGATCTGTTCGGCGAACAAGCCGTATTGTGCGGTGGCGTGGCCGAGTTGATCAAAGCAGGTTTTGAAACCCTGACCGAAGCCGGTTACGCGCCTGAAATGGCTTACTTCGAATGCCTGCACGAAATGAAACTGATCGTTGACCTGATTTTCGAAGGCGGTATTGCCAATATGAACTACTCCATTTCCAACAATGCGGAGTACGGCGAATACGTTACCGGCCCTGAAGTGGTCAATGCTTCCAGCAAAGAAGCCATGCGCAATGCCCTGAAACGCATCCAAACCGGCGAATACGCAAAAATGTTTATCCAAGAGGGTAATGTCAACTACGCGTCTATGACTGCCCGCCGCCGTTTGAATGCCGACCACCAAGTTGAAAAAGTCGGCGCACGACTGCGTGCCATGATGCCTTGGATTACGGCCAACAAATTGGTTGACCAAGACAAAAACTGATTGTTTTCAAACGGGACTGCCTACACATCGTGTAGGCAGTTTGTTATATGGATACCGTCTGAACATCGTGTTCGGACGGTATCTGTGTTGCGGATGAATTTAAACAGGCACAGTTCTGTCAGTCGCCCAGAGCCGCGACCATTACCGCTTTAATCGTGTGCATACGGTTTTCCGCCTGATCGAACACGATGCCGGCCGGACTTTCGAATACTTCTTCTGTAACTTCCACACCGTTCAGCCCGAAGGTTTCGTAAATCCATTCGCCGACTTTGGTTTCGCGGTTGTGGAAGGCGGGCAGGCAGTGCATGAATTTGACTTGCGGATTGCCCGATGCCGCCATCAGTTCGGGCGTAACGCGGTAATCTTTCAGCAAATCGATGCGTTCCTGCCAGACTTCTTTCGGCTCGCCCATGCTGACCCATACGTCAGTATGAATGAAACCGACACCTTTGACGGCTTCATGCGCGTTTTCGGTCAGGGTAATTTTTGCACCGGTTTCTTTGGCGGCGGCGTGTGCGGCGGCGATAATGCCTTCAGACGGCCACAGGCTTTGAGGTGCGCCGATACGCACGTCCATCCCCAATTTTGCCCCTAAAATCAGCAGGGAATTGCCCATGTTGTAACGCGCGTCGCCGACGTAGGCAAACGCGGTTTGGTTCAAAGGTTTGCCGCTGTGTTCGCGCATAGTCAGTGCGTCGGCAAGCATTTGTGTGGGATGGAACTCGTTGGTCAGCCCGTTGAACACGGGTACGCCCGCATATTTTGCCAATTCTTCGACAGTTTCCTGAGCGAAGCCGCGATATTCGATGGCATCGTACATTCTGCCTAAGACGCGCGCCGTGTCTTTGATGCTTTCTTTGTGCCCGATTTGGCTGGCGGACGGTTCCAGATAGGTTGCATCCGCGCCTTGGTCGCGTGCGGCGACTTCAAACGCACAGCGTGTGCGCGTGGATGTTTTTTCAAAAATCAGGGCGATGTTTTTCCCTTTCATCCGCTGAATCTCGCGCCCTGCCTTTTTGGCGTCTTTCAACTCGGCGGCAAGGTCGAGGTAGGCGGTGATTTCTTCCGGCGTGAAGTCCAAAAGTTTCAGAAAATGGCGGTTTTTCAGGTTCACTGTCGTTTCCTTTATGTGGGGGCGTGAACGCCGTATTTGCAGCGTTTGCGGAAAGAAGGGTTAGCGGAATGTTGCAATGCCGTCTGAAGGTTGTTCAGACGGCATTGCGGTCAATCCCTTACTCCATACGGATGACGGGGATCAAACATTCACGGTCTCGGCTACTTCGTTGTAGCTGTCGATTTCGTTGAAGTTCATATAGCGGTAGATTTTATCGCCCTGTTCGTTGATGATGCCGATATTGGCTTGATATTCTTCAACGGTCGGGATTTTACCCAGTTTGGAGCAAATCGCCGCCAACTCTGCCGAGCCGAGGTAAACGAAGGTGTTTTTACCCAAGCGGTTCGGGAAGTTGCGGGTCGAAGTGGACATGACAGTTGCACCTTCGCGTACTTGGGCTTGGTTACCCATACACAACGAACAGCCCGGCATTTCCATACGCGCGCCGGCGCGGCCGAGTACGCCGTAGTGACCTTCGTCGGACAACTCTTTCGCGTCCATTTTGGTCGGCGGCGCTACCCACAGGCGGACGGGGATGTCGCTCTTGCCTTCCAAAAGTTTGGAGGCGGCGCGGAAGTGGCCGATGTTGGTCATACAAGAACCGATGAACACTTCGTCGATTTTGGTGCCGGAGCGTTCGGACATAAAGCATACGTCGTCCGGGTCGTTCGGGCAGGCGATAATCGGCTCTTTGATGTCGTCCATGTTGATTTCAATCACGGCGGCGTATTCGGCATCTTTATCCGCTTCGAGCAACTCGGGATTTGCCAGCCATTTTTCCATAGCTTTGATGCGGCGTTCCAAAGCGCGCGGATCTTGATAGCCGTTGGCAATCATATTTTTCATCAACACGACGTTGGATTTCATGTACTCGATAATCGGCTCTTTATTGAGCTTCACGGTACAGCCGGCGGCGGAGCGTTCGGCGGATGCGTCGGTCAATTCAAAGGCTTGTTCCACTTTCAAATCGGGCAGGCCTTCGATTTCGAGGATGCGGCCGGAGAAGATGTTTTTCTTACCGGCTTTGGCAACGGTCAGCAAACCTTGTTTAATCGCGTAAAGCGGGATGGCGTTCACCAAATCGCGCAGGGTTACGCCCGGTTGCAGCTTGCCGCTGAAACGTACCAATACGGACTCGGGCATATCGAGCGGTATTACGCCCGTTGCGGCGGCAAAAGCGACCAAGCCTGAACCTGCGGGGAAGGAAATACCGATGGGGAAACGGGTGTGGCTGTCGCCGCCTGTACCGACTGTATCAGGCAACAGCAGGCGGTTGAGCCACGAGTGGATGACGCCGTCGCCCGGACGCAGGGACACGCCGCCACGGGTAGAAATAAAGGCGGGCAGTTCTTTATGGGTTTTTACATCGACAGGTTTCGGATAGGCAGCGGTGTGGCAGAAAGACTGCATCACCATATCGGCGGAGAAGCCCAAACAAGCCAAGTCTTTCAACTCGTCGCGGGTCATCGGCCCGGTCGTATCTTGCGAGCCGACCGTCGTCATGCGCGGTTCGCAGTAAGTACCCGGACGCACGCCTTGTCCTTCGGGCAGACCGCAGGCGCGACCGACCATTTTTTGCGCCAAGGTGAAACCGACTTTGCTTTCGGCAGGCGCTTGCGGCAGGCGGAATGCAGTAGAGGCAGGCAGTTTCAGGGCTTCGCGCGCTTTGGCGGTCAGACCGCGACCGATAATCAGGTTGATACGGCCGCCGGCTTGCACTTCGTCCAGCAATACTTGTGATTTCAATTCAAACTCGGCAACAGTCTCGCCGTTTTTCACGATTTTACCTTCATAAGGAAGGATATTGACGACATCGCCCATTTTCAGCGCGGAAACATCGACTTCAATCGGCAGCGCGCCGGAGTCTTCTTGAGTGTTGAAGAAAATCGGCGCGATTTTACCGCCCAAGCACACGCCGCCGAAGCGTTTGTTCGGCACGAACGGGATGTCTTCGCCGGTATGCCAAATGACGGAGTTGGTCGCGGATTTGCGTGAAGAACCGGTACCGACCACGTCACCGACGTAGGCAACCGGATGGCCTTTGGCTTTGAGTTCTTCCAACAATTTAATCGGACCGACTTCGCCCGGTTTGTCGGGCGTGATGCCGTCGCGCGGGTTTTTCAGCATGGCCAGCGCGTGCAGCGGAATATCGGGACGACTCCACGCGTCGGGCGCGGGAGAGAGGTCGTCTGTATTGGTTTCGCCGTCAACTTTGAAGACGGTAACGGTGATTTTTTCGGGAACTTTGGCACGGGAGGCGAACCATTCGGCATCTGCCCAAGATTGCAAAACTTCCTGCGCGTATTTGTTGCCTTTTTCGGCTTTTTCCTGAACGTCGTGGAAGGAATCGAACATCAGAAGCGTATGTTTCAAGCCTTTGGCGGCAATGGGCGCGAGTTTGTCGTCGTCCAAGAGTTCGATTAAGGCGTGGATATTGTAACCGCCGAGCATCGTACCTAAGAGTTCGGTCGCATATTTGGGGGAAACCAGCGGGCTGGATGCGCTGCCTTCGGCAACGGCAGCCAGGAATGAGGCTTTGACTTTGGCGGCATCGTCCACACCGGGCGGAACACGGTGGGCAAGCAGCTCGACCAAGAACTCGCCTTCGCCTGCGGGCGGGTTTTTCAGCAGCTCAACCAAATCGGCGGTTTGCTGCGCATTCAAAGGGAGGGCGGGAATGCCGAGGGCGGCGCGCTCGGCGGCGGCTTTACGATAGGCTTCTAACATCTCTTTGTTCCTTTTTCCGTTTTTCTTTTGTCGGGTTGCAAATGATCTGCGTTAAATATTGTAAACAACATTTGTACCGCTATCATAGACTAGTTTTAACAAAAATGGAACTGTTATGACAGGTAATGTATAGTGGATTAACAAAAATCAGGACAAGGCGGCGAAGCCGCAGACAGTACAAATAGTACGGCAAGGCGAGGCAACGCCGTACCGGTTTAAATTTAATCCACTATAATAGGGAACGGCTATATGTCTGCGGGAATGTGTCAATGAAAACAAATGCCGTCTGAAGAGAGGTTCGGACGGCATTTGTTCAACGTTTTTCCTGTTTGCGTATCAGCCAAACAGCGAGCAAGGCGAAGGCTATGGTAGGCAGTGCGCCGGCGAGGAAGGGTGGGGTGCCGTAGAGTTGGCTGGTAAACCCGAAGAGCCTGCCGGCAAGGTGGAACAGCAATCCGAGACAGATGCCGCCGAAGAGTTTTAAGCCCATATTGCCGTGGCGCGTGGTTTGCGGCGTAAAGGCGAAGGCAACGAGCGCCATGACCCATGCGGCGACGGGGTAAACGAGTTTACGCCACCATGCGATGGCGTAGATTTGGGTGTTTTGGCTGTTGTTTTGGAGGTGGCGGATGTAGGTGGTCAGCTCGCCGACGGACATTTGGTCGGGCTTGACGAGCAATACGTCCATCAGGTTGCGTCTGACGGCAATCGGCCAAGTTTCTTCGGCGGCGGCGGATGTTTCGATTTTGTCTGTACCCATGATGCTGCGGCGGATGTTTTTCAACTGCCAGCTGCCGTCGCTGTTCAAAACGGCGGAATCGGCTTCCACTGCCTCTGCCAATTCGTTTTTATCGTTGCGCGCCCAAATTTTGATGCCCAAAAGCGTATGGTCGGGCAACATTCCGCGCACATTGATAATGCTGTTTTTTTCTTTCAGCCAAAGGCCGGTATGGCCGGTGCTGATTTTGCCGTTAATGGCGGCGGCTTTGATGTTTTCGGCTTTTTGGCTCAGCGTGGGCGCAACCCATTCGCCGAGCGCGACGGCGGCAATAGCAAAAATAAAACCGAACTGAGACAGAATCAACAGCAGCTTTTTGGTGCTCATGCCGCTGGCTTTGATGACGGCCAGTTCGCTGCCGGCGGCAAGCTGGCTGAGAGAGGCCAGTCCGCCGATGAGGACGGCGAGGGGCATGAGTTCGTAGGCGCGGGCGGGCATTTTGAGGGCGGTGTAGCCCAGCATTTCCCATATGCCGTAACTGCCTTTGCCGAGGTTGCCGGTTTCGTACAGGATTTCAAAAAAGCTGTACAAAGCGAGGAAGGCAAGGAGCGCGCAAACCGCCATAACCGCCATTTGGCGGATGATGTAACGTGAAATCAGGTTCATTTTCCGCCTTTCAATGTCAGACTTTTGCCAACCGCCTGCCAGAAGGGCTGGCTGGGCATACTGCGGACGCGCAGAAGTACGATTGCGATGACGAACATGATGATGTGCATAGGCAGCAGTCCGAGCCAAAAATGGATTTTGCCGTCTTCCACGGCTTCAAAAAGCAGGGTCAGCCCGTTTTGGTAAATTAAAAACAAACCGATGGCAATCAAGATATTGTAGGTATGTCCGCTGCGCGGGTTGAAATAGGAAAGCGGCACGGCGAGTAGGCAGAGCAGGAGGACGCTGACGGTCAGCGAGATGCGCCACATCAATTCTGCCTGATGTTGCGGATTGCTGCTGCCAATCAGTTGGGCGGTCGAAATGGTGCGGCGGTGGGAAACGGGGTCGATAAGTTTGGGCGTGGTGCTGATAATCAGGTTGAGTTTTTGGAAGGAAACCTGATTGTAGTCGGCGCGTCCGGGCGTGCCGCTGTAACGGTAGCCGTGGCGCAATTCGAGCGTGCGTTTGTTGTCGTTCAGCGAGAAGTTACCTTCTTTGGCGAAGATGATGTTGTCGCCGCCGTTTTTGTCCTGTTCGCGCAGGAACAGGTTTTTCATGATGCCGGATTCGGTGTCAAAGGTTTCGACGAAATAAACCCTGCCGTTGCGCTTGCCCAAGTTATTGAACTCGCCGGCTTCCACCAAAGACAATTCCTGCTTCTGCTTCAAAATTTCGGCATATTCGCGGCTGCGCAGTTCCGCCCACGGTATCACCCAAAGCTGCATGACGGCAATCAGGATGGCAAACGGCACGGCAAACTGCATGACGGGGCGTATCCACTGTTTCAACGCCAATCCGCAGGATAGCCAGACCGACATTTCGCTGTCGCGCCAGTAGCGGGTCAATACGGTCAGCGTGCTGATGAATGCGGTCAACACCAGCAAAAGCGGGGTCATACCGATGACCCAGAAGCCGACTAAGGCCAACACGGCATCGATGGCGACACGCCCGTCAGCTGCGCGGCCAAGCAGGTTGATCGCCTGCGTGGACACCAACACCGCCAAGAGGACGACGAAAATGCCGACGGCGGTAAAAGAGAGTTCTTTGATGAGGTTTCTTTGATAAATCATAAAATCGCGGTCAGGGGGGCGGTAAGCGGTTCGGCGCAGGCGGATTGTCCGCGCAAGGTTTCAGACGGTGCTTCAAAAAATGGGGTACAATCGTGCCAGCCGTCTTCAGACGGTCTTTTGAGCGAAAGGCCGTCTGAAAACTACCGACAACCGTCTCAATCAGGAGAATAAACGTGGAATTTAGCACAAAAACCGAAATCTTGCAGGAACAGCAGGCAGGCGCGCAGTTATTTGTCTGTGCCGACAAAGCACCCGAGCACAACACCGCCGCACATGCGCTCTTCTCTGCTTTGGAAGAGGGTCAGAATTTTTCCGACACCAAAATCCCGACGGACAACGGTTTGCAGGCAGTCGCCGTCGTCCGCCTCGAAAAAACCGACCGCGCCGCGCTGAACAAAGCCGCCGCCGAAGCCGCCAAATGGGCGCAAAATCAAGAAACGGTCAATGTGGACGTTCACGCCTTTGATGAGGCGCAAGCAGCAGCCGTTGCCGAAGCTTTTGCCATCGCGTTCGGCAACGCTGCATATCGTTTCGACCGCTACAAAAAAGAAGCCAAGCCCGCCAAATTTTCGCAAGCCGTGTTCCACAGCGCACACGAAGCCGCCGTCAAAGAAGCCCTGCGCGTTGCCGAAGCACAAGTTTACGGACAAAGCCTCTGCCGCGACTTAGGCAATGCCGCACCGAACGAATGCACGCCTGAGTTCCTCGCGCGTACCGCCAAAGCCGAAGCCGAGAAACTGGGCGCGCACGCCAAAATCATCGAAAAAGACTACATCAAAGAAAACATGGGTTCGTTTTGGTCGGTTGCCAAAGGCAGCGTCGAAGACCCATATTTGGTTGAACTGAGCTATTTCGGTGCGGCCGACAAAGAAGCCGCGCCTGTGGTATTGGTCGGCAAAGGCATTACCTTCGACACCGGCGGCATTTCCCTCAAACCCGGCCTGAACATGGACGAAATGAAGTTTGACATGTGCGGCGCGGCAACCGTCATCAGCACCTTCTGCGCCGCCGTCAAACTGCAACTGCCGATCAATCTGATTGCCATCGTCGCCACTTGTGAAAACATGCCTTCCGGTGCGGCAAACAAACCGGGCGATGTTGTGAAAAGTATGAAAGGGCTGACGATTGAAGTGTTGAACACCGATGCCGAAGGCCGTCTGATTTTGTGCGACGCGCTCACTTACGCCGAGCAGTTCAAACCCAAAGCCGTCATCGATGTCGCCACCCTGACCGGCGCGTGCATCGTCGCCTTGGGGCACGATGTCAGCGGCGTGATGGGCAATAATCAGGATTTGATCGACAGCCTGCTTGCCGCTTCCTACAACGTGGACGACAAAGCGTGGCAACTGCCGCTCTTTGAAACCTACAAAGACCAGCTCAAATCCAACTTTGCCGACATCCCCAATATCGGCACGCCCGGCGCGGGCACGATTACCGCCGCAACATTCCTGTCCTATTTCACCGAAGGCTACCCGTGGGCGCACCTTGACATCGCGGGTACGGCGTGGAAATCCGGAGCGGAAAAAGGTGCGACCGGCCGCCCCGTACCGCTGCTGATGAACTATCTGCGGAATCTTTAATTTTGCCACAATGCCGTCTGAAGCTTTTCAGACGGCATCATCCTGTCAAATGCTCAAACAATATGCCGAAAGTTACCTTTTATACCCATGTTGACCAAATCCCCCTTTTTACCTGTCGGTTGATTGCGCGCGCTATCCGAGACGGCGGCAGGATACTGGTGTGGTCCGACTCGTTCGGGCGGCTTCAGGAATTGGACAAAATGCTTTGGCAATACGAGGCCGAGAGTTTTATTCCGCACGAAATTTGGGAAACGGAAGAAGCCATGCCGTCTGACACATCCGTCCTGCTTGCCTGCGGCGGCAATCTGCCCCGAATTCCCGAAGGCATGGCCGTTTTGAACCTGTCCGACGGTTTTTGGAACACCGCTTCGGTCTTGCCCGCGCGCGTTTTGGAAATCGTCGGCAACAGCCTGGAAGATCTCGCCGACGCGCGCGAACGCTTTACCGCCTACCGCCGAAGCGGTTTTGCCATCGAACATCACGGCATGGAGGGCAAGGCATGAAATGCCGTCTGAAATGCCGCATTTACAAAATTTAAACAGGCTTGATTCTATTTGATTAAAATTTCCTTATCGGTTGAACCCCGCCACTTGGACATCTGTCCTTCGGGGCGGTAGAATCAAACCTTATTTTGGAAGGTTCAATCCCTTCCAAAACAGGGCAACACACAGATTGACGCTTTATGTGCCATCCTGTGTGTTGAAACATTCAAACTCGGCTATAATCCCGTTTTTCCGACTTTATCGACAGCGAAGATCCATCATGAACACCATTTTCAAAATCAGCGCACTGACCCTTTCCGCCGCTTTGGCGCTTTCCGCCTGCGGCAAAAAAGAAGCCGCCCCCGCATCTGCATCCGAACCTGCCGCCGCTTCTGCCGCGCAGGGCGACACCTCTTCAATCGGCGGCACGATGCAGCAGGCAAGCTATGCGATGGGCGTGGACATCGGACGCTCCCTGAAACAAATGAAGGAACAGGGCGCGGAAATCGATTTGAAAGTCTTTACCGATGCCATGCAGGCAGTGTATGACGGCAAAGAAATCAAAATGACCGAAGAGCAGGCCCAGGAAGTGATGATGAAATTCCTGCAGGAGCAGCAGGCTAAAGCCGTAGAAAAACACAAGGCGGATGCGAAGGCCAACAAAGAAAAAGGCGAAGCCTTCCTGAAGGAAAATGCCGCCAAAGACGGCGTGAAGACCACTGCTTCCGGTCTGCAGTACAAAATCACCAAACAGGGTAAAGGCAAACAGCCGACAAAAGACGACATCGTTACCGTGGAATACGAAGGCCGCCTGATTGACGGTACCGTATTCGACAGCAGCAAAGCCAACGGCGGCCCGGCCACCTTCCCTTTGAGCCAAGTGATTCCGGGTTGGACCGAAGGCGTACGGCTTCTGAAAGAAGGCGGCGAAGCCACGTTCTACATCCCGTCCAACCTTGCCTACCGCGAACAGGGTGCGGGCGAAAAAATCGGTCCGAACGCCACTTTGGTATTTGACGTGAAACTGGTCAAAATCGGCGCACCCGAAAACGCGCCCGCCAAGCAGCCGGATCAAGTCGACATCAAAAAAGTAAATTAAGTCCGAATCCATGCCCGAAACAGGTTTTCGGGCATTTTTACGGCAAATGCCGTCTGAAGCCACCAAACAGCGGTTCAGACGGCATCCCTTTCAAAAGCGCACCATTATGAAAAACATCGTCATCCTGATTTCCGGACGCGGCAGCAATATGCAGGCAATCGTCAATGCCGCCATTCCCAACGTCCGCATTGCCGCCGTGTTGAGCAACAGCGAAACGGCTGCCGGTTTGCAATGGGCGGCCGAACGCGGCATCCCGACCGACAGCCTGAATCATAAAAACTTTGAATCACGACTTGCCTTCGATACGGCCATGATGGAAAAAATCGACGCATATCAACCCGATTTGGTGGTTTTGGCAGGTTTTATGCGGATTCTGACCCCTGAGTTCTGCGCCCATTACGAAGGCAGGCTGATGAACATCCATCCGTCCATCCTGCCCTCGTTCACCGGGCTTCATACGCACGAACGCGCTTTGGAAGCAGGTTGCCGCGTTGCCGGCTGTACGATTCATTTCGTTACGGCCGAGCTGGATTGCGGCCAGATTGTATCGCAAGGGATTGTGCCGATACTTGACGGCGATACGGCAGACGATGTTGCCGCCCGGGTTTTGGCTGTCGAACACAAACTCTATCCGAAAGCCGTTGCCGATTTTGCCGCCGGCCGCCTGATTATCGAGGGAAACCGTGTCAGAAATTCGGAAAACGCCGATGCCGCCCGTTTCCTGACGGCGTAAACCGGGCGGGAGCAAATGATGAAGACTTTTAAAAATATATTTTCCGCCGCCATTTTGTCCGCCGCCCTGCCGTGCGCGTATGCGGCAAGGCTACCCCAATCCGCCGTGCTGCACTATTCCGGCAGCTACGGCATTCCCGCCACGATGACATTTGAACGCAGCGGCAATGCTTACAAAATCGTTTCGACGATTAAAGTGCCGCTATACAATATCCGTTTCGAGTCCGGCGGTACGGTTGTCGGCAATACCCTGCACCCTGCCTACTATAAAGACATACGCAGGGGCAAACTGTATGCGGAAGCCAAATTCGCCGACGGCAGCGTAACCTACGGCAAAGCGGGCGAGAGCAAAACCGAGCAAAGCCCCAAGGCTATGGATTTGTTCACGCTTGCCTGGCAGTTGGCGGCAAATGACGTGAAACTCCCCCCGGGTCTGAAAATCACCAACGGCAAAAAACTTTATTCCGTCGGCGGCCTGAATAAGGCGGGTACGGGAAAATACAGCATAGGCGGCGTGGAAACCGAAGTCGTCAAATATCGGGTGCGGCGCGGCGACGATACGGTAACGTATTTCTTCGCACCGTCCCTGAACAATATTCCGGCACAAATCGGCTATACCGACGACGGCAAAACCTATACGCTGAAGCTCAAATCGGTGCAGATCAACGGACAGGCCGCCAAACCGTAAATCGGTTTTCAGGCGGTTTTTTGCCGTTTTGTCCATATTTCTGTATAAGCCGCTTGAAATTGTTTACAATAACGCCATATGCAAAGCGGTTTTAACGCTATTTTCGGGAATGACACCATGCAGGTTACATCAAAATGGATAGACGGAATGTGTTTTGTCGGCACGGCGGAAGGCGGGCACAGCGTCGTTATGGAGGGATCGGCGGCAGAAGGCGCGGCCAAGCGCGGGCCCAGCCCTTTGGAAATGCTGCTGTTGGGGGTGGCGGGCTGTTCGAGCATCGATGTGGTGATGATTGCCGAAAAACAGCGTCAGAAAGTGACTGACTGCCGTGCGACGGTTACGGCGAAACGGGCGGACGATGCGCCGCGCGTGTTTACCGAAATCCACATCCATTTCAAGGTAATCGGGCATGATTTGAAAGAATCGGCCATTGAGCGCGCCGTTCAGATGTCTGCCGAAAAATACTGTTCGGCTTCGATTATGTTGGGCAAAGCGGCAAAGATTACCCACAGTTTTGAAATTGCCGGGGCGGAGAAATAGGCATCGGGCGGCATTATGGGGAAACGGCTGATGCCGTCTGAAGGGGAAAGGTGCGGCAGGCAATCCGGAGGCGCATAAAAAAGCAGATATATTCGGACTGCACCTCCCGAATATATCTGCCTGCTGTTTCCTCTTTATTCAGCCTTTATAATACTTGGACTTGTCGGGGTATTGTGCAGGCTTGATTCCGGATTGTCAACAATTTTCGGTCAAATTTTAAATGCCGCGTTTTAAAATGATGCCCGCCTGATTTTGCGGGCGGGCGGAAGGCGGGAATCCCGTTATTTTAAGCCATATATTAATTTATTGAATTAAAATAAATTTGTGCAATATAGTGGATTAACAAAAACCAGTACGGCATTACCTCGCCTTGCCGTACTATTTTTGTACTGTCTGCGGCTTCGTCGCCTTGTACTGATTTAAATTTAATCCACTATACTTTAATTTCTCCTACACGATTTGAAACACAGCCGTCTTAAAACATCTTAAAAAATTTGAAAGCGTAAAAATACGGAAAACATCATGAACTTTGAAAACGACGACATTATCCATGCGCCGACCACGTCTTCCCTGATTCTCGAAGAGCGGCACGATTCGGAGCTGTTCCGTGTTTACGCCCTGATTTTGGACGGCATTACCGATCAGGTGCTGCTGCCCGGCAAAAAGCTGACCGAATCCGAACTTTGCCGTCAGATGGTGTGTTCGCGCAACACCGTCCGCGGCGCGCTGTCGCTTTTGGCGCACGACAAGATTGTCGATTTGCAACCCAACAGGGGCGCGTTCGTCCACGTTCCCGATTTGAAAGAAATGCAGGATGTGTTCAATGCGCGCATCGAAATGGAGACGATGATTTTGAACATCCTCGCCGGCCTGCCGGATTTGGAAACGCGCCTCAAGCCGCTTTATGCGATGATAAGGTGCGAAGAAGAGGCCTCCGGCAGGGGCGACCGCGTCGGCTGGAACCGCCTGTCCAATGCCTTCCACGTCGAACTGGCGCGCCTGGTGGGCAACGATGTGCTGTTCGACATTATGAACACGCTGTGCGCGCGGTCTTCCCTGATTGTCGCCGTGGCGGGCGTGCATCGCGAGAAAAAACACGCCATCAATACGCATACGCATTCCGAACACCGCGAAATCCTCGACCTGCTGCTGGCGGGCAGGCGCAACCGCGTGGTCAAAATCCTGCGCCGCCATTTGGGCAACTGTATGGAGCGTTTGGAAAAGACTTTGGAAGATTGAATGCCGGAGCGGATAAGCCGCCTGAACCTTCAGGCGGTTTTTTAATGGCGAACCTGATGCCGTCTGAAATATGGATGCGGGTATCTGCAATTTTCAGACGGCAATTTTTAAGCCGCACATATCATGCGGCAATAAAGGAGGGTAGGGGATGAGCAGCCTGATGACGTTGTTTTCGGTATTGGTACCGATGTTTGCCGGATTTTTTATCCGTGTTCCCAAGCCTTACCTGCCCGCTTCGGACAAGGTGCTGTCGGTTTTGGTGTATGCCGTGCTGCTGCTGATCGGCGTATCGTTGTCGCGCGTGGAGGATTTGGGTTCGCGGTTGGGCGATATGGCGTTGACGGTTCTGTGGCTGTTTGTTTGTACGGTAGGGGCGAACCTGCTTGCCTTGGCAGTGTTGGGAAAGTTGTCCCCGTGGCGGATAGGGGGAAAAGGGAAGGGCGTTTCGGTCGGCGTGTCGGGCAGTGTGAGGCAGCTCGGATGCGTACTGATCGGTTTTGTGTCCGGCAAATTGATGTGCGATATTTGGATGCCGTCTGAAAACGCGGGTATGTACTGCCTGATGCTGCTGGTGTTCCTCATCGGCGTACAGCTCAAAAGTAGCGGCGTATCGTTGCGGCAGGTTTTGCTTAACCGGCGGGGCATCCGGCTGTCGGTTTGGTTTATATTGTCATCTCTTTCAGGCGGGCTGCTGTTTGCCGCATCGGCAGATGGTGTGTCGTGGACGAAAGGTTTGGCGATGGCTTCCGGCTTCGGTTGGTATTCCCTCTCGGGTTTGGTAATGACCGAGGCTTACGGGGCGGTATGGGGCAGCATCATGCTGCTGAACGATTTGGCACGAGAGCTGTTTGCACTGGCATTTATTCCGCTGCTGATGAAGCGTTTTCCGGATGCGGCGGTGGGGGTCGGCGGCGCGACCAGTATGGATTTCACATTGCCCGTAATTCAGGGTGCGGGCGGTTTGGAAGTCGTGCCGGTAGCGGTCAGCTTCGGCGTGGTGGTCAATATCGCCGCCCCGTTTCTGATGGTGGTGTTTTCCACGCTGGGCTGAACGCGGTAAAATCGGCATCCCGATGCAAGGAAGCAGAAAACGATGAAACCGAAAATCCAAAGGCATGGAGAGATTTTAAGCCTTGTCCGCCGGCATCAGTTTATGTCGGTGGACGAGCTTGCCGCCGCATTGGACGTTACCCCGCAGACGATACGTTGCGACATCCGCGAGTTGGAGGAGGGCGGCAGCCTGAAACGCCATCACGGCGGCGCATCTTCGGGCGGAAACTTGCCGGAGGGTCTGCCCGCCGACCGCCAAACCCGGTGTCAAAACGAAAAAAACGCCATTGCCCGGCTGATTGCGGAACACATCCCCGACGGTTCGTCGCTGTTCGTCAGTATCGGTACGACCATGGAAGCCGCGGCATCAGAGCTGGTGAAGCGGCGCAGCAGCCTGCGGATTATTACCAACAATATCCACGTCGCCTCCGTCGTTTCGGCACGTACGGATTACACGGTCATCATCACATCCGGCGTCGTCCGCCCTTTGGACGGCGGTATTACCGGCGTGGCGACCGTCGACTTTATCAACCAGTTCAAAGTCGATTATGCCGTGATGAGCACGCACGGCGTGGAAAGCGACGGTTCGCTTTTGGATTACGATTACAAGGAAGTCAGCGTCATGCAGGCGATGATTGCCAACGCGCGCGTCCGTTTTCTCGGCGTGGATCACAGCAAATTCCGCAGCAACGCGCTGGTCAGGCTCGGCGGCATTACCGGCGTTTGACAAAGTATTTACCGACAGGTTGCCTGATACCGCGATGCAGAAGATGCTGAAAGAGGCGGGGGTGGAATGCCTGATTGCCGATGCCGTCTGAACGCTATGTCAAAGCGCGCAAGTCGGGTACAATAAACACATCATCAAACCGCTTCAGACGGCATACGGAACCTCCCCAATGCCGTCTGAAGCCATCTGTTTAAAGAAAACCATGCTCAATAAAGACCAATTCGCGGACAACCATTTCATCCGCACCATCATCGAAGACGACCTCAAAAGCGGCAAACACGAAGCCGTCCAAACCCGTTTTCCGCCCGAACCCAACGGCTATTTACATATCGGACACGCCAAATCCATCTGCCTGAACTTCGGTTTGGCGTATATTTACGACGGTTTGTGCAACCTGCGTTTCGACGACACCAACCCGGAAAAAGAAAACGACGAATACGTCAACGCCATCAAAGAAGACGTCGAGTGGCTCGGTTTCCATTGGGCGGGCGAACCGCGTTTCGCTTCCGACTATTTCGACCGGCTTTATGACTACGCCGTCGGTTTAATCAAAGACGGCAAAGCGTATGTCGATGATTTGACGCCCGAAGAAATGCGAGAATACCGCGGTACGCTGACCGAAGCGGGTAAAAACAGCCCTTACCGCGACCGCAGTATCGAAGAAAACCTCGACCTGTTCACGCGTATGAAAAACGGCGAGTTCCCCGACGGCAGCAAAACCCTGCGCCTGAAAATCGACATGGCGGCAGGCAACATCAATATGCGCGATCCCGTCATCTACCGCATCCGCCGCGCCCATCACCACAACACCGGCGACAAATGGTGCATCTACCCGATGTACGATTACACGCATTGCATTTCCGATGCCATCGAAGGCATCACGCATTCCTTGTGCACGCTCGAATTTGAAGCGCACCGTCCGCTTTACGACTGGGTGTTGGACAACATCCCCGCGCCGCACGCCACCCGTCCGCGCCAATACGAGTTTTCCCGTTTGGAGCTTTTGTACACCATTACCTCCAAACGGAAATTGAATCAGTTGGTTGTGGAAAAACACGTTTCCGGCTGGGATGATCCGCGTATGCCGACCATTTCCGGTATGCGCCGCCGCGGCTACACGCCCGAAGGGGTGCGCCTGTTTGCCAAACGCGCCGGTATTTCCAAATCTGAAAACATCGTCGACATGAGCGTGTTGGAGGGTGCGATTCGCGAAGAGCTGGAAAACTCCGCCCCGCGCCTGATGGCGGTTTTGAACCCGCTCAAAGTGACCCTGACCAACTTTCAAGCCGGCAAAACCCAAAGCCGCCGTGCCGCGTTCCATCCGAACCACGAAGAAATGGGCGATCGCGAAGTACCTGTTTCACAAACCATCTACATCGAAGCCGACGACTTTGCCGAAAATCCGCCCAAAGGCTTCAAGCGTCTGATTCCCGGCGGCGAAGTACGCTTGCGCCACGGCTATGTCATCAAGTGCGGTGAAGTCGTCAAAGACGAAGCAGGCAATGTGGTTGAACTCAAATGCAGCATCGACCACGACACCTTGGGCAAAAATCCAGAAGGCCGCAAAGTTAAAGGCGTGATTCACTGGGTTTCCGCCGAACACGCCGCCGAAATCAAAGTCCGTCTGTACGACCGCCTCTTTACCGTCGAGCGTCCCGGTGCCGTGCGCGGCGAAGACGGCGAATACCTGCCGTTTACCGATTTCCTCAATCCGGAATCCGTTAAGGAAATCACTGCTTACGCCGAACCTGCCGCGAAAGATTTGCCGGCGGAAAGCCGTTGGCAGTTCGAGCGCATCGGCTATTTTGTGACCGACCGCCAAGACCACGGCAAAGACACGCCGGTGTTTAACCGCACGGTGACGTTGAAAGATTCTTGGCAGCCTAAGTAAACCCCATCCTTGCCGTCTGAATATTGTTCGGGCGGCATTTCTCCTTTACCCGCGAAATGCGGCACATTCGGCACACCGGAAAGGAAATATGATGAAAGTCCTCTTTATCGCCGACCCGATGGCAAGTTTCAAAACCTACAAAGACACCACCTACGCGATGATGCGGGAAATGGCAAAACGCGGCTGGCGGCTGTTTCATACCTTGAGCGGGGAATTGTCTGTAAACGGCGGTTTGGTAACGGCACAGGCATCGGCATTTGAATTTTCGGGTGCAAAAAACGATGATGACCATGAATGGTTTAAAGCGGCGGACAAAGTTCAGACGGCATTAAAAGAATTTGATGCCGTGATTATGCGTACCGATCCGCCGTTCGATATGCAATACCTTTACTCCACCCAATTACTGACGCTGGCGGAACAGCAGGGCGCGAAAGTGTTCAACAGCGGACGGGCGATGCGCGACTTTAACGAAAAACTGGCGATTTTGAATTTCAGCCGCTTTACCGCGCCCACGCTGGTAACGACCCGTTCCGCCGATGTCCGCGCATTTTTGAAAGAACACGGCGACATCATCGTCAAACCGCTCGACGGCATGGGCGGCATGGGCATCTTCCGCCTGACCGAAAAAGACCCCAACATCGGCAGCATCCTCGAAACCCTGATGCGGTTTGATTCCCGCACCATTATGGCGCAACGCTACATTCCCGAAATCGTACACGGTGACAAACGCATCTTGATTATCGGCGGCAAAGTCGTCCCCTATGCTTTGGCGCGTATCCCGCAAAACGGCGAAACACGCGGCAATCTGGCGGCAGGCGGGCGCGGTGTGGCGCAGGAATTGGACGGACGCGACCGGGAAATTGCAGAGACTCTGGCTCCCGAGCTTAAACGGAGCGGCATCCTGCTGGCCGGTTTGGACGTTATCGGCAGCAACCTGACCGAAGTCAACGTAACCAGCCCGACCGGATTCCAAGAAATTATGAAACAAAAAAGTTTCGACGTGGCGGCAATGTTTGCCGATGCCGTTGCCGCGTGGTCGGTACGTTAAACCGATGCCGTCTGAAAGGCTTTTGCTTCGTAACCGCTTGGTTTGTTCGGGCAGGCAGGGTTTTCCCTCGGCCGGCAGGGATGCGCTTAATGCCGTACGGACACCCCTCCGCCCCGTTTTCAGACGGCATATATTGAGGACATTTTGAAAGGATACCGATGGAACCTTCCTCCTACGCGGCAGAAAAAAAAGGCAAAAGCGGCATCAGGCGCGTCATCAACGCATTCGGCTATTCGATAGACGGCATCGCCGCCGCCTACCGTTACGAAGCGGCATTCCGTCAGGTTTTGTGGCTGAACGCGCTGCTGGTGTGCGCGGCATTTTTTTGGGTTTCCGAAACCGCCGTCCGCCTGCCGTTGATTATCGCGTCTTTTGTGTCGGTCATTGTCGAACTGTTCAACACCGCCGTCGAAGCCGCCGTCGATCATACTTCGACCGAAAAACACGAGCTGGCCAAACGCGCCAAAGATGCAGGTTCTGCCGCACAACTGGTTGCCATGCTGATGTTGGCGGCGGTTTGGCTGTCCGCCCTGTTCGGGTAAAACGCTTGCAGCAGGATTTGTAATCCTTTAGGATTAGTATTTATTATTCACTTAATCTACATCAAATTTCCGAGCAGTATTTGTATGTAAGATTAAGCACATTCCCCGTCTGATATTAAAGGAGCAGGAAGATGAAAAAATTATTGGCAGCCGTGATGATGGCAGGTTTGGCAGGCGCGGTTTCCGCCGCCGGAGTCCATGTCGAGGACGGCTGGGCGCGCACCACTGTCGAAGGTATGAAAATGGGCGGCGCGTTCATGAAAATCCACAACGACGAAGCCAAACAAGACTTTTTGCTCGGCGGAAGCAGCCCCGTTGCCGACCGCGTCGAAGTGCATACCCACATCAACGACAACGGCGTGATGCGTATGCGCGAAGTCAAAGGCGGCGTGCCTTTGGAGGCGAAATCCGTTACCGAACTCAAACCCGGCAGCTATCACGTGATGTTTATGGGTTTGAAAAAACAACTGAAAGAGGGCGACAAGATTCCCGTTACCCTGAAATTTAAAAACGCCAAAGCGCAAACCGTCCAACTGGAAGTCAAAACCGCGCCGATGCCGGCAATGAACCACGGTCATCACCACGGCGAAGCGCATCAGCACTAATCTGCTGGAAATATTTGAAATGCCGTCTGAAAAAGCCCGGGCTTTCAGACGGCATTTTTATGCCCGCCTTTAAAATGTGTTAAAATCCGCCTTTAAAAACCGCCGTTTCCAAGCCATCCTGCGTATGAATACGACATCAAACACTTCCAATATCATCGTCGGGCTTTCCGGCGGTGTCGATTCTTCCGTAACCGCCGCCCTGCTCAAGCAGCAGGGTTATCAAGTGCGCGGTGTGTTCATGCAGAACTGGGAAAACGACGACAACGACGAATATTGCAGCATCAAACAGGATTCGTTCGATGCCATCGCCGTTGCCGATATTGTCGGCATCGACATCGACATCGTTAATTTCGCCGCGCAATATAAAGACAAAGTTTTTGCTTATTTTCTTCAGGAATACAGTGCGGGGCGCACGCCGAATCCGGATGTGTTGTGCAATGCCGAAATCAAATTCAAATGCTTTTTGGACTACGCCGTAGGGCAGGGCGCGGATACCATTGCCACCGGACACTATGCGCGCAAAGAAGTCCGCAACGGCGTGCATTACCTGCTCAAAGGTTTGGATCGAAACAAAGACCAAAGCTATTTTCTCTACCGCCTCAAGCCTTTCCAACTCGAACGCGCGATTTTTCCGTTGGGCGGTTTGGAAAAACCCGAAGTGCGCCGCCTTGCCGCCGAATTTAATTTGCCGACTGCCGCTAAAAAAGACAGTACCGGCATCTGTTTCATCGGCGAGCGTCCGTTCCGCGAGTTTCTGCAGAAATACTTGCCGACCGACAACGGCAAAATGGTGACGCCCGAAGGGAAAACCATCGGCGAACACGTCGGGCTGATGTTTTACACATTGGGTCAGCGCAAAGGATTGGGCATCGGCGGCGCGGGCGAACCGTGGTTTGTTGCGGCTAAAGATTTGACGAAAAACGAACTCATCGTCGTACAAGGACACGACCATCCGCTGCTCTATACCCGCAGCCTTGTGATGAACGATTTGAGTTTCACGCTGCCCGAACGTCCGAAGGCAGGACGCTATACCTGCAAAACGCGTTACCGTATGGCGGACGCGCCTTGCGAATTGTGCTATTTGGATGATGAAACCGCCGAGCTGGTGTTTGACGAACCGCAATGGGCGGTTACGCCGGGTCAGTCCGCCGTGCTGTACGACGTCGACATCTGTTTGGGCGGCGGCATCATCCAAACGACCGACAAACCCGTCATCATCACGCGATAAAGGTAATGCCGTCTGAAACGGTTTTCAGACGGCATTGTTCCGCTCAATTCCACTTTAAAGACCGATACCTATGGAAAAAATCTGGTTAGAAAGCTACGAGAAGGGCGTCAGTGCCGAAATCGACATCACGCAATACAATTCCGTCAGCGACGTATTCCGCCAAAGCGTGGAAAAATTTGCCCGTCTGCCCGCTTTTCAAAATATGGGCAAAACGCTCACTTATGCCGAAACCGGCAAACTGGCGACCGATTTCGCCTCTTATCTGCAAAACGTCCTCAAGCTGCCGCGCGGCGAACGTGTTGCCATTATGATGCCGAACGTATTGCAATATCCGATTGCCCTTTTCGGTATTTTGCAGGCAGGTTTGGTGGCGGTGAACACCAATCCGCTCTATACGCCGCGCGAGTTGGAGCATCAGCTGAAAGACAGCGGTGCGACCGCCATCATCGTTTTGGAAAATTTCGCCAACACGCTGGAGCTGGTGCTGCCGCGCACGCAGATCAAACACGTCATCGTCGCCTCCGTCGGCGAAATGTTCGGGCTGCTTAAAGGTTCGCTGATCAATTTCATCATCCGAAAAATCAAGAAAATGGTTCCCGAATACCGTATTCGGGAAACCGTTTCCTTTCAGACGGCATTGAAAGAGGGGGCGAAGCACGTTTTCCAACCTGTCGCATTAAACCGCGAAGATACCGCATTGTTGCAATACACGGGCGGCACGACAGGCGTTGCCAGAGGCGCGGTGCTGAGCCACGGCAACATCTGCGCCAATATGCTTCAGGCAAAAGAATGGATTAAAAACCAATTGCGCGAGGGAAAAGAAACCGTTATCGCCGCCCTGCCGCTGTACCACATCTTCGCCCTGACAGTGAATCTGATGATTTTTGCCAATGCCGGCTCGAAAATTGTCCTGATTGCCAACCCGCGCGATATGAAAGGCTTTATCGGCGAACTGAAAAAGCAGCGGGTTAACGTATTTATCGGCGTGAACACGCTGTTTAACGCGATGGTCAACCGGCCCGATTTCGCCGAAGTCGATTTTTCAGGATTGCGGCTGACTTTGGGCGGCGGTATGGCGACCCAAAAAGCCGTTGCCGAAAAATGGAAAAAAATCACCGGCACGCCCATCGTCGAAGCCTACGGTTTGACCGAAGCCAGCCCCGGCGTGTGCTGCAACCCCTTAAACATCGAATCATACAGCGGCAGCATCGGTTTGCCCGTCCCGTCCACCGAAGTCGAACTGCGCGACGCAAACGGCAAAGAAGTCCCCGTTGGGCAGCCGGGCGAATTGTGGGTAAAAGGCCCGCAAGTGATGCAAGGCTACTGGAACCGCCCCGAAGAAACCGCCAAAGCCATAGACGCGTGCGGCTTTTTGGAAACCGGCGATATTGCCGTGATGGACGAAAAAGGCCGTCTGAAGCTGGTCGATCGCAAAAAAGACCTCGTCGTCGTTTCCGGATTCAATGTTTATCCGAACGAAATCGAGGAATTCATCGCGCACCACGAAAAAGTTATGGAAGTTGCGTGTATCGGCGTACCCGACGAAAAAACCGGCGAGGCACTCAAAGTGTTCGTCGTCAAAAAAGACCCGTCTTTGACCAAAGAAGAACTCACCGCTTTCTGCCGTACCGGTCTGACCGCATACAAAGTGCCGAAAGACATCGAATTCCGCGACGAGTTGCCCAAGTCCAATGTCGGCAAAATCCTGCGCCGCGAGTTGCGCCAAAGTGCCGGGAAATAAAGAAAAGATACCGTCTGAAAACAGCCGTCCACCGTTCAGACGGCATCCGTCCGTTTGCAAGAACCGCGCGCTTCACGTTAAAATCACGCATTCCAATACGGGTATTCCATCATGACCAAATTCATTTTCGTAACCGGCGGCGTTGTCTCCTCACTGGGTAAAGGTATCGCCGCCGCTTCTATTGCCGCCATCCTCGAATCGCGCGGCTTGAACGTTACCATGCTCAAGCTTGATCCTTATATCAACGTCGATCCCGGCACGATGAGCCCGTTCCAACACGGCGAAGTGTTCGTAACCGACGACGGCGCGGAAACCGACCTCGACTTGGGACACTACGAACGTTTCATCGATTCCACGATGACCCGCCGCAACAGCTTCAGCACGGGTCAGGTGTACGAAAACGTCATCGCCAAAGAACGACGGGGCGACTATTTGGGCGGCACGGTTCAAGTCATCCCGCACATTACCGACGAAATCAAACGCCGCATCCACGAAGGCGCGGCGGGTTACGATGTGGCGATTGTCGAAATCGGCGGTACGGTCGGCGACATCGAATCGCTGCCGTTTTTGGAAGCCATCCGCCAGATGCGAAGCCAGTTGGGACGCAACAACACCCTGTTCGCCCACTTGAGCTACGTTCCCTACATCGCCGCCGCAGGCGAAATCAAAACCAAGCCGACCCAGCACACCGTGAAAGAAATGTTGAGCATCGGCTTGCAACCCGACATCCTGATTTGCCGTATGGACAGGAAAATGCCGGCAGACGAACGCCGCAAAATCGCCTTGTTCTGCAACGTGGAAGAGCGCGCGATTGTCGGCAGCTACGATGTGGACAGCATCTACGAATGCCCCGAAATGCTGCACGACCAAGGCATCGACAACATCATTACCGAGCAGTTGCAGCTTAACGTGCAGCAGGCGGATTTGACCGCGTGGAAAAAAATCGTCCACGCCGTCAAAAACCCGAAACACACCGTCAAAATCGCGATGGTCGGCAAATACGTTGATTTGACCGAATCCTACAAATCATTGATTGAAGCCTTGAAACACGCGGGCATCCATACCGAAACCGATGTGCAGATTACCTTCGTTGACAGCGAAAGCATCGAGAAAAACAAGGGCGACGTTTCCGTACTCAAAGATATGGATGCCATCCTCGTTCCCGGCGGCTTCGGTTCGCGCGGCGTGGAAGGCAAAATCGCCGCCGTGCGCTACGCCCGTGAAAACAACGTGCCATACTTGGGCATCTGCCTCGGTATGCAGATTGCGCTGATTGAATACGCCCGCGACGTGGCAGGTTTGAAAGGTGCGAATTCCACTGAGTTTGACTTGAAATGCGCTGCCCCCGTCGTCGCCCTGATTGACGAATGGCAAACCGCCGACGGCAGCGTCGAAACCCGTGACGAATCCGCCGATTTGGGCGGCACGATGCGTTTGGGCGCGCAAGAAGTCGAATTGAAAGCAGGCAGCCTCGCCGTCAAAATCTACGGCAGCGGACACATTCGCGAACGCCACCGCCACCGCTACGAAGTCAACAACAACTACGTTTCCGCGCTGGAACAGGCAGGTTTGGTCATCGGCGGCGTATCCGCCGGACGCGAACGCTTGGTCGAAACCATCGAACTGCCGAACCATCCTTGGTTCTTCGCCTGCCAGTTCCATCCCGAGTTCACGTCCAACCCGCGCAAAGGGCATCCTTTGTTCACCGCGTTTGTCAAAGCCGCGTTGAACAATAAAAAAGCCTGATAAAGCGTTACTTGATGATCAAAATGCCGTCTGAAAGCCTGACAAAGACTTTCAGACGGCATTTTCGCAAATCGGGGATCATAAAAGCCATCAATTTAATTTGGGTCTGTACTAGATTAGCAGATATGTTACCCTCGAAATATGAAGATAACACACTGCAAATTAAAGAAAGAAGTACA

>25 |ref|NC_017511.1| Neisseria gonorrhoeae TCDC-NG08107 | Coordinates: 230207,241363 | Forward

CGATTTAACTTCGGCACACCGCCCCGGCAGCTAAAAATCCTGCGGGATCGGTGTGGAATTTAGGGATAATCTAGTACAGCCCCTAAGTTTTTAGTATGAGGGGAATATGCGGAGAATTTGGTTTTAATCAGCCGCCGGAGTAGGGAATAATCCGCATCGTCGGGCGGCAGCGTCCAAATGGCGTGAATATGGTTCGGCAGCACGCATACGGCGACGGTTTCAAAGGGATATTGTTTTTGCACATCCATATAAGCCGCACGCGAAGCCGATATGTCCGACAAGCAGACGCGATTTCGGGGCGGCGGGTTTGACGGTGAAAAAGAATGTGCCGCCGGCAATGAAGTTTCTGCGATAACGCGCCATAGGATTTTTGTTTGTGGTTTGGGTGTTGGAGAAGGAATGGTTTGTTGTTTAAGGTTTAACTTTGTTGGACCTGATGTTTCCAAACGACATTGGAGGCCGTCTGAAAGCACTAGCTTCAACGAAGTTAAAAGTTGAAACGGCAAACCGTCAATCAAGCAACCGCGTGCGTACCGCACACACGCAGTGGGCGGGTTAGTTGTGCAGGCTGCTTTTCTCCGACAACACCCCCATCAGATGTTTGCCTAAAATTTCCGACAATTTGACAGGAACCGCATTGCCAATTTGTCGCATTGCTTCTCCCCAAGCTCCAGAAATGACAAATTCTTCAGGGAATGTTTGAAGAAGTTTTGCTTCATAGCTGGTAAAGTATCTGACTGTTCCATCATCATAACGAATCATATTTTCTCCGCCGGGAACGCCATGCCCACCTGCTTTAATGGTTTTGGACGGTTCGTCTATCCCGCTTCCTGTGTGTCCGGGATAAATTCTTGCGCCATCCCTATATTCATGTCCTGTAATTTTATGATTCCCCAAAGGATGCGGGATGTCGGACAAGGTATCCCTTACCGTTTGCCAAGGTTTTTTTTCTGGTTCGAATATACCGTATTTTTTTTGTAACTTTTCAGCAATATCTTTATTGAATCTTTTGGGTTCGTTGTGTTTTTCCCAGTATTCCCCCGTTACATACTTTTCCCAATTCAACCTGTCTTCGGAATGTGTTCTTTTTGGGAATTTCCAATCAATATCCAAATCGGCCCGGATACCTACGATAACCACGCGTTCGCGTTTTTGCGGCACTCCGTAATCAGCTGCATTCAACAATTGATATGAAACCTTATATTTGATTCCTTTATATAAATTGAATTCAATTTCTTTCAGCCGTGTCAAATGGCCTTTCCAATCTTCATTCTGCAAAATACCAAGATTCGGATAAGTAAGCCGAAGTAGGATATATTCAAAATAATCAGCGAATGATTTTCTTAAAAGTCCTTTTACATTCTCAAAGATAAAAGCTTTGGGGCGGTAATACTCCACATATCTGACCGCATGAGGAAACATATCCCTTCTGTCTTCATGGGCAAGGTGTTTCCCGCCCAATGAAAAGGGCTGGCAAGGAGGACCGCCTGCAATGACTTCTATGCCTTCTTGACTGGAAAGATCAAAATCTGCCACATCTCCCTGATAAACAAGCTTGGGGTTAAAGTTGCTTCTTAAAGAATTGCATGCATCTTTATTGAGTTCAATAAAAGATGCATGTTGGAATCCTGCAAGTTCAAGGCCTTTTGCCAACCCGCCCGCGCCGGAAAAAATTTCCAAAGACTTCATAGGTTATTTTCTCTTTATTATCTCTAAATGTGTGTTGATATTTTCAAGTATGGACTCATAGCTATTCGGTATGCCGTTTAGCCTATCTGCCCAAACCCGCCCGCTATGAAGGACATCCCAATCCGATTTGGCTTGTTCGTAACGGCCTTTTCCGGGGTCGTGGTTTCCGAAACCATCGACACAAGAGTTCCATAGAGGTTTGTGTAATTTAATCAGGGCAGCTTCGATTGTGCCTATCATGTCAGAACCGGCACCTTCAAAGATAACGAATCTGCACATGAAATCGGAAGGATCTAAATCAGAAACAGCGGCAATACTCCTGCTGTGTTCTTTCAACCGGTGGAACAGTTCGGTTGATTGGTTTAGCGCATTATCAGAATTTCTTGCTTGCCGCCAGCCTTTGGGGACGGCCTTGCCAACATAAATTGGCGCATTGTAGGATAATCTGTTCCAGTCCGCATATTGTTTGTACAAAGGATTGTTACCGATATAGTAAATCGCATAAACCCCTGCACCTTTGAACTGTTTGGGTGGAGGAAGTGTATGAACAGGCGTACCATGAAAAAAGCGTATGGCATCTTTAACCAGTTCTACAAAAGCTTCGTTATGATAGATATGCTTGCTGCGGTCAAAATTATTGTTCATTTTCTATATCCGTTCGTTTACTTCAAAACCACTTTCAAATACTTCCCGGTATAACTCCCCTTAACCTTCGCCACTTCCTCAGGGCTACCTTTCGCGATAACCTTACCCCCACCATCGCCGCCTTCCGGCCCTAAGTCCACAATCCAATCCGCGGTTTTAATTACATCCAGATTATGCTCGATAATCACAATCGAGTTGCCTTTGCCTTTCAGACGGCCTATGACTTCCAGCAGCAGGGCGATGTCGGCGAAGTGCAGGCCGGTGGTGGGTTCGTCGAGGATGTAGAGCGTTCTGCCGGTGTCGCGTTTGGAGAGTTCCAAGGCGAGTTTGACGCGCTGGGCTTCGCCGCCGGAGAGGGTGGTGGCGGATTGGCCGAGGCGGATGTAGCCGAGACCTACGTCCATCAGGGTTTGCAGTTTGCGCGATACGGTGGGGACGGCGTCGAAAAATTCGCGGGCTTCTTCGACCGTCATGTCGAGGACTTGGCTGATGTTTTTGCCTTTGTATTGGATTTCGAGGGTTTCGCGGTTGTAGCGTTTGCCGTGGCAGACTTCGCAGGGGACGTACACGTCGGGCAGGAAGTGCATTTCGACTTTGATTACGCCGTCGCCTTGGCAGGCTTCGCAGCGGCCGCCTTTGACGTTGAAGGAGAATCTGCCGACGTTGTAGCCGCGTTCGCGCGAGAGGGGTACGCCGGCGAAGAGTTCGCGGATGGGGGTAAACAGGCCGGTGTAGGTGGCGGGGTTGGAGCGCGGGGTGCGGCCGATGGGGGATTGGTCGACGTTGATGACTTTGTCGAGGTGTTCGAGGCCGCGGATGTCGTCGTATGGGGCGGGTTCTTCTTGGGCGCGGTTGAGTTCGCGGGCGGTGATTTTGGCGAGGGTGTCGTTAATCAGGGTGGATTTGCCGCTGCCGGATACGCCGGTGATGCAGGTAATCAAACCGAGCGGCAACTCGAGGGTGACGTTTTTGAGGTTGTTGCCGCGCGCACCTTTGAGGACGAGCATTCGGTCGGGATTGACGGGCGTGCGTTCAGACGGCACGGCAATGGATTTTTTGCCGCCGAGGTATTGTCCGGTAACGGATTTTTCGCATTTGGCGACATTTTCGGGTGTGTCGGCAATCAGTACGTTGCCGCCGTGTTCGCCCGCGCCGGGGCCCATATCGACGACGAAATCGGCTTCGCGGATGGCGTCTTCGTCGTGTTCGACCACAATTACGCTGTTGCCCAAATCGCGCAGGCGTTTGAGGGTGGCGAGCAGGCGGTCGTTGTCGCGCTGGTGCAGGCCGATGGAGGGTTCGTCCAAAACGTACATCACGCCGGTCAGGCCGCTGCCGATTTGGCTGGCGAGGCGGATGCGCTGGGCTTCGCCGCCGGAAAGGGTTTCGGCGGAACGGCTTAAATTCAGGTAATCCAGCCCGACGTTAATCAAGAAGCCGAGCCGCTCGGTGATTTCTTTGAGGATTTTTTCGGCGATCTGTTTTTTGTTGCCGTCCAAATCCAGCGTTTCGAAGAATCGGTGGGTTTTGGTCAGCGGCCAGGCGGAGACTTCGTGCAACGGTTCGCCGCCGACGTAAACGTAGCGTGCTTCTTTGCGCAAACGTGCGCCGCCGCAGCTCGGGCAGGCACGGTGATTTTGGTACTCGCGCAGTTTTTCGCGCACGGTTTCGCTGTCGGTTTCGCGGTAGCGGCGTTCGAGATTGGGGATGATGCCCTCGAAGGCGTGGCTGCGGTTGAAGGTGGTGCCGCGTTCGGACAGGTAGGTGAAATCAATGACTTCTTTGCCCGAGCCGTGCAACACGACTTTTTTGACTTTTTCAGGCAAAGTTTCCCAAGCGACGTTGACATCAAATTTATAATGATGCGCCAGCGATTGAATCATTTGGAAATAGAATTGGTTGCGTTTGTCCCAACCGTCAATCGCGCCGGTTGCCAGCGACAATTCGGGATGGGCGACTACTTTTTCGGGGTCGAAGAAATTGGTGTTGCCCAAGCCGTCGCAAGTCGGGCAGGAACCCATCGGGTTGTTGAACGAAAAGAGGCGCGGTTCCAATTCGGGCAGGCTGTATGAGCATACGGGGCAGGCAAAACGCGCGGAAAACCAATGTTCTTCGCCGCTGTCCATTTCCATCGCCAGCGCGCGCTCGTTGCCGTGGCGCAGCGCGGTTTCAAAACTTTCCGCCAGTCGTTGTTTGATGTCGGCTTTCACTTTCACGCGGTCGATGACGACGTCGATATTGTGTTTGATGTTTTTTTCCAGCTTCGGTACTTCGTCAAGCTGATAGACTTCGCCGTCCACGCGCACACGGGCAAAGCCTTGCGCCTGCAAGTCGGCAAAGAAATCGACAAACTCGCCCTTACGCTCGCGCACCGCCGGCCCCAGAATCATCACGCGCGTATCTTCCGGCAGTTTTAATACGGCATCGACCATTTGAGATACGGTTTGGCTGGATAGCGGCAGCTTGTGTTCGGGGCAATACGGCGTGCCGACACGGGCGTATAAAAGGCGCAGGTAGTCGTGGATTTCCGTTACCGTGCCGACGGTGGAACGCGGATTGTGGCTGGTGGATTTCTGCTCGATGGAAATCGCAGGCGACAGGCCTTCAATCAAATCGACATCGGGTTTGTCCATCATCTGCAAAAACTGCCGCGCATAGGCAGACAGGCTCTCGACATAACGCCGCTGCCCTTCGGCATATAAAGTATCAAACGCCAGCGATGATTTGCCGCTGCCTGACAATCCTGTTACCACCACGAGCTTGTGGCGGGGAATGTCCAAATCGATGTTTTTCAAATTATGCGTGCGCGCGCCGCGAATGCGGATGGTGTCGTTGTCGTGCGAATGTCGGGGATGATGGTTGCACATAATGGATGCCGCCTGAAAAAATAAAGGAAAACCGATATTGTAGCACTTTCTCGGATGCCGTCTGAAGCCGCGTTCAGACGGCATTTGTCGGCGGAACGCGGCGGATTCCGTTATAATGTCGCTATTTAATATATTTGAATAAAAGGATGACAAATGAACCGTCTTTATCCCCACCCGATTATCGCCCGTGAGGGCTGGCCGATTATCGGCGGCGGTTTGGCTTTGAGCCTGCTGGTGTCGATGTGCTGCGGCTGGTGGTCTTTGCCGTTTTGGGTGTTTACCGTATTTGCATTGCAGTTTTTCCGCGACCCTGCGCGTGAAATTCCGCAAAATCCTGAAGCGGTGTTGAGTCCGGTTGACGGCCGTATCGTGGTGGTCGAGCGCGCACGCGATCCGTATCGTGATGTCGATGCTTTGAAAATCAGTATTTTTATGAACGTGTTCAACGTGCATTCGCAAAAATCGCCTGCCGATTGTACGGTAACGAAAGTGGTCTATAACAAAGGCAAATTCGTGAATGCGGATTTGGACAAAGCCAGCACGGAAAACGAACGCAACGCGGTTTTGGCGACTACGGCTTCCGGTCGTGAAATTACTTTTGTTCAAGTGGCCGGTCTGGTGGCACGCCGTATTTTGTGTTACACCCAAGCAGGTGCGAAACTGTCCCGCGGCGAACGCTATGGCTTTATCCGCTTCGGTTCGCGCGTGGATGTGTATCTGCCTGTCGATGCGCAGGCGCAAGTGGCGATTGGTGATAAAGTAACCGGCGTAAAAACCGTATTGGCGCGTTTGCCGCTGACTGATTCTCAAGCCGATCCTGTTTCACAAGCTGCTTCGGTTGAAACAGCGGCAAACCCATCTGCCGAACAGCAGCAAATCGAAGCGGCGGCGGCTAAGATTCAGGCGGCTGTGCAAGATGTGTTGAAAGATTAATTTTGCGAACTGAAATAGAAAATATCAGTACCATCATTCACACGAATGAGGAAGTTTGGTTTTTTGAATTTTTGCTAATGTTCACACCGTTATGTTCACGAAAATGGGAATCCGGGAACTTAACGTTACGGTGATTTATCAGAAATAACAGAAACCGAACGAATTGGATTCCCGCCTGCGCGGGAATGACGACTCATTAGTTACCTAAAACTTAAAAAACAGAAACCTTTACGCCGTCATTCCCACGAAAGTGGGAATCCGGGAACTTAACGTTACGGTGATTTATCGGAAACGGCTGAAACCGAATGAATTGGATTCCCGCCTGCGCGGGAATGATGGGATCTTGGGTTTTTGCTTTTGATTTTTCTGCTTTTGCGAGAATGACGGCGTGAAAGCAAGAATGATGAAACAAAGAAAATGGGAATGATGGCACAGTGGTTTGTTCCTTGTCTTTGCCATATTTCCTAACAAGTTGATTAAAAAGAAAAAAAGGTTTTCAGAATGCCGTCTGAAAACCTTTTTTGTTTGCCTGTCCGATTTTAAAACTTCACGTTTACGCCGCCGGTAAAGCTGCGGCCCATTTGCGGCGTATCAGAGAGGAAGCTGCTGTGGGCGTAAACGGATTGGTTGAGCAGGTTGTCGGCTTTGACGTACCAATTCCACTCGCCATAGCGCGTATTGCGGCGGTAGTTTGCACCGAGGTTGAGCATATGGTGTCCGGGCGTACGCGTTTCGTAGCGGGCGAGTTTGTTTTGGGCGAACACGCGGTAGTAGTCCAAATTGGCATCGATACGGTCGGTTAGCGAGGTTTTCAGGTGGAAGCCGAGGCGCGCAGCCGGAATGCGGGGGGCGTTTTGGTCGTCCTGCGCGATGAAGGGACGTTTGCCGTAGGGATCTTCCCTGCCGGGTAGGGACGGCAGGTTTTTCAGACGGCCTCGTACATAGTCGCCGGAAACGCCGATGCGGTAGCGCGGTGTCGGTTTGAAGTAGATTTCGCCTTCCGCGCCGTAGAAGTCGGCACCGGATTGGTTGTAGCGCACGAGCTTCATTTCGCTGTCGTCTTCGATGGATTTGGGGCCGCGTCCGTCGTTTAAGGTTTGGGCGTAAATGTAGTTGCCGAATCGGTTGCGGTAGGCTGCCAGATTGTATTGCCAGCGGTCGCCTTCGTAGCCCAGCGCGAGTTCGATATTGTTGGAACGCTCTTTGTTGAGGTGTTTGTTGCCGACTTCAAAGGTGTTGGTGGCGACGTGCTTGCCGTGTGCGTACAGTTCTTGCGTTGACGGCAGGCGTTCCTGATGGGAGGCGGTCAGGCTGAGTTTGTGGTGTGGCGTGAAATACCAGTTGCCCGAAAGTGCGAACGAGCGGGCGGTTTGGCGGTGCGCGCCGAGGTCGGGCAGGGGCTGGTTGTAGTAGTTTTCTCGATCAATCAATGCTTTGTCGTACCGGATGGAGGCTTTTTGTTTTTCCACGCGTACGCCGCCTTCAAGCGTGAAGTTGTCCCAATTTGCCTGTTCTACACCGAAAAAGCTGTAATGGCGGACATTGTTGTCAATCAACATCGGTTGTTGGACGGTTTCGGGAATGGCGGAAAGCGCGCTGGATTTTTGTCCCAAATATTGCACGCCCCAGCTGCCTTTCAGACGGCCTATGGGTTGGTGGCGCAACTCGATACGGGCGTTGTGTGTTTTGTTGTTGAAGAAGTTTTCTACTGCATCGCCTGCTTTTTCGTCGTGGTGGTAGTCATTGCGGTTCAGATGTACGCGCAGGGCTTCAAAACCGGGGAATGGCTGCTTCCATTCGGCGCGGAGTTCGTAGCGTTTGTTGCGCAGGTCTATCCACGGTTTGCCGTTGTGGGTGTGTGCGTGTGCACCGTCGCCGTCGTGGAAGCCGCAGCTCAAGCCCGGATTGTCGTAATCGATGTCTTCTTCGGTCAACAAGTGCGGATAAAGCTGCAAATAGCGTTTGTTGATCAAACTCTTTTGCCAGATGATGTCGGCGTGGCAATCATCGTATTCGTGGCTGTGGGCAGGCAGGCCGTAGCGGTCGCGACGGTCGCTGTATGCTGCGCCGATAAAGCCTTTTTCGCCTACCCAAGACAGCCCGATGCTGCCCGTTTGCGAATCGGCATGGCTGTCGGGCAGGCGTTTCAGATTGCGGTAACGCGGTACGGCGTAATCGCCCGATTTGCGGTACAAGCCTTCGGTATGCAGCACGAAGTTTTTGCCCAGTCCGATATTGATGCCTGCGGATGTCAGTTTTTCTAAATTGCCGCTGCTCAAACGCAATCCGGCTTCGCCCGATACGCCGTTTTCAGGCATTTTTTCGGGGATTTTTCCATCGGCAACATCGACCAGCCCCGCCACATTGCCCGAGCTGTACAAGAGCGTAACCGGCCCGCGCAGGATTTCAACCTGTTGCGACAAGGCGGTATCTACCATAATGGCGTGATCGGGAGAAAAGTCCGCCATATCGCCCGTTTCGCCGTGATGGTTCAATACTTTAATCCGTCTGCCCGTTTGACCGCGAATAACGGGAGCGGATGCGCCGCCGCCGTATTGCGAGGCATGAATGCCCGGTACGCCGTCCAAAGCGTCGCCCAAGTTGACGGCTTTTTGGCGCAAAGTATCGCCGGAGATGATTTTGTCGGAGGCGGTCGAAGTGTGCAGCAGCCCCGAAGTCGCGCGCGGACGGCTTTTGCCGACGACGCTGACCGTTTCCAAGCCCACCGATTGCTCAGTTTCATGCGCTTGGGCGAGGAGGGGTGTGTTGATTAAAAGAATTGATAAAACAATGGGTTTGAGTGTGATTTGTGCCATTTTGGCTTCTCGTCGCATTTCAAAAGTTTGTTATTATATAACATTGCATTTTTTATATCATAAGATTTTGAGAATACTCAGAGGGCATAGGCAAAAGTTTTTCAAATGACACGGTTGTGATATTTAATGGCGTTAATTTGTATTAACCCTAATTTTAGGGAAGTTAATGATGTGAACACGAACGCCGCTATATGTTAGCTATCGGATAACCAAAATTTATGAGGGCATAAAAACGGTACGTTCCCGAACGGTCGAAAAGGTGGCAAAATGGCGTACTTGTCAGAACGCGAGGCTCTGCGCCAGATTCACGAGGGCGCATCGGGCACGCTAAGATATAAGGGGGTATGGATGGGGGTATCGGAAATGCAGTTAATAATAAACAAATTATAAATCAATAGGTTAATCACAAAATGCTTTTGTTTATCGACAATTACGACAGTTTTACTTACAACATCGTCCAGTATTTCGCAGAATTGGGGCAGGAAGTCGCCGTGCGCCGCAACGATGATATTACGTTGGAGGAAATCGAGGCATTGAATCCGCAATATCTCGTTATCGGTCCCGGACCGTGTTCCCCTAAGGAGGCGGGTATTTCAGTAGAAGCCATGCGCCATTTTGCCGGCCGGCTGCCGATTATGGGCGTGTGCCTCGGGCATCAGACGATAGGCGAAGCGTTCGGTGGAGATGTGGTACGGGCAAAAACCTTGATGCACGGTAAGGTGTCGCCCGTGTCCCATTCGGGCAAGGGTATGTTTAAGGGTTTGCCCAATCCGGTTACCTGTACGCGTTATCACAGCCTCGTTATCGAACGCGGCACGCTGCCGGATTGCTTGGAAATCACGGCGTGGACGGAAGACGGCGAAATTATGGGCGTGCGCCATAAGGAATATGCCGTCGAGGGCGTGCAGTTCCACCCCGAAGCCCTCTTGACCGAACGCGGACATGATATGTTGAACAATTTTTTAGTTGAATTTCAAAACTTCAAACCGCAAAAAATCTGACGTGATGCCGTCTGAAGCCCTTCGGGCGGCATTTTCGTCCGAATATTGAACGGAGGACAAAAAATGATTACACCGCAACAGGCTATCGAACGATTAATCAGCAATAACGAGTTGTTTTACGATGAAATGACCGACTTGATGCGCCAAATGATGAGCGGAAAAGTGCCGCCCGAACAAATTGCGGCGATTTTGACCGGCTTGCGTATCAAGGTTGAAACCGTTTCCGAAATCACCGCCGCCGCCGCCGTGATGTGCGAGTTTGCGTCAAAAGTGCCGCTGGAGGATGCGGACGGGCTGGTCGATATCGTCGGTACGGGCGGGGATGGCGCGAAAACCTTCAATATTTCGACGACTTCGATGTTTGTTGCTGCAGCGGCAGGCGCAAAGGTTGCCAAACACGGAGGCCGGTCGGTCTCTTCCTCCAGCGGTGCGGCTGACGTGATGGAGCAAATGGGCGCAAACCTCAACCTGACTCCCGAACAGATTGCCCAAAGTATCAGGCAGACCGGTATAGGGTTTATGTTCGCACCCAATCACCACAGTGCCATGCGCCATGTCGCCCCGGTGCGCCGTTCGCTCGGTTTCCGAAGCATTTTCAACATATTGGGTCCGTTAACCAATCCTGCGGGCGCGCCGAACCAGCTTTTGGGCGTGTTCCACACCGATTTGTGCGGCATTTTGTCGCGGGTCTTGCAACAACTCGGCTCAAAACACGTTTTGGTCGTTTGCGGGGAGGGCGGTTTGGATGAAATTACACTGACGGGCAAAACGCGCGTTGCCGAACTCAAAGACGGAAAAATCAGCGAATACGACATCCGCCCAGAAGATTTCGGTATCGAAACCCGCCGCAATTTGGATGAAATCAAAGTTGCCAATACTCAGGAATCTTTGTTGAAAATGAACGAGGTACTGGACGGGAAAGAAGGGGCGGCGCGCGATATCGTATTGCTTAATACCGCCGCCGCCTTATATGCCGGAAATATCGCTGCTTCGCTTTCAGACGGCATATCTGCCGCACGGGAAGGCATCGATTCAGGTAGGGCGAAGGCGAAAAAAGAGGAGTTTGTCGGTTTTACACGGCAATTCGCCTAAGCCGGCAAACTTGATATAAAGCAACAAATGCCGTCTGAACGGCGGAATTGGCGTTTCAGACGGCATGAGGCCTTTGCAAAAAAGCCCTTCCTTCGACATCCGAAACCCAAACAC

>26 |ref|NC_017511.1| Neisseria gonorrhoeae TCDC-NG08107 | Coordinates: 241364,248268 | Forward

GCTTTCAACAGGTTCAAACACATCGCCTTCAGGTGGCTTTGCGCACCCACTTTGAGCAGCCCGAAATAGGCTGCCCGCGCATAGCGGAATTTACGGTAGCGGCATAAGGTGCTGCAACCGGGGATGCCCGGTTCGTCAAAACGGCAAAACAGGTTGAAACCGATGCGGGTGATGAGGCTGTGTTCGAGTTCGGGATCGGAGAGGCTGTGCCATTGTCCGGGCAGGACGGCTTTGAACATGGACGACAGGGGATGGGCGGGACGGCCGCGGCGGTCTCGGAGGTAACGGGTTTTTTGACGGATCAGGTATTGTTCGATCGGCTGCCAATCAATCACCTGGTCCAACTCCGATAGCGGGAAGCGGCCGATGTGTTTGGCAGTCATGGCTTGGGCGGTTTGCCGGAAGAAGGTGTTCATGGGAAATCCCCTAAATGCCTTGGTGGGAATTTAGGGGATTTTGGGGATTTTTGCAAAGGTCTCAAGAGATGTGTTTAAGCACGCGGAAGGCTTTCTGTTTGCGTCAGGTCAAATAATGATGCCGTCTGAAAACCGAATCGGCTTCAGACGGCATTTATATCGTAACGGTCGGATTGGGTAGGTTGGCGCACCTGTCCGGTTTTCGGTTTGGCAAACCGTTTTTTTGTTGGGTTCAGTGTTTTCTGATAGGCGGTTGCGGCATCGGATTTGCCCAGCCCCGCCAGTACGCGGATATGCTCGGCAGCAGATTGTGCCAGAGGTTCAAGGGTGTAGCCGCCTTCGAGTACGGATACGATTTTGCCGGGGCAGCTGGATGCCGTCTGAATGATTTTGTGTGTCAGCCAGGCAAAATCCGCCTCGTGCAGGTTGAGCCTGCCCGATTCGTCCAGACGGTGTGCGTCGAATCCTGCCGACAGCAGCACCAGTTCGGGTTTGAATGCGGCAAGCCGGGGCAGCCACTGTCTGCGGACGGCTTCGCGGAATGTGCGGTTGCCCGTTCCCGACGGCAAGGGCAGGTGCACCATATTGCCGCCGTCGGGCATACCGTTGTTTTCGGGGAAGGGGAAAAGGTCGGTTTCAAACAGGTTGAAAAACAGGATGCGCGGATCGTCTTTGAATATTTCTGCCGTACCGTCGCCGTAGTGGACATCGAAATCGATGACGGCAATGCGTTTCAGGCGGTATTCGGCAATGGCATGCATGACGCCGGCGGCAACGTTGTTCAGCAGGCAGAATCCGCCGGCTTTGCCGCTTTTCGCGTGATGCTCCGGCGGGCGGGCGGCGCAAAAGGCGTGCCACGCTTTGCGTTTCATGACCATATCGACTGCCTGAACTGCCGAACCGGCGGAAAAACGGGCGGCGGACAGGGAGTTTTTGCTGATAACGGTGTCGTCGTCCAAACGGCAAATCTTGCCGTCTTCGGGTAGGCAAGATTCCAAACGGTTCAGATATTTGCTCGAGCGGACAAGTGCAAGGCGCGTATCGCTGATTTCTTCCGCCTCTACGGTTTGGAGGTGCTGCCAAATACCGGCGCGGCGCAATGCCTGCTCGATGCAGAGGATGCGGTCGGGCGAATCGGGATGGTTTGCCCCGGGGTCGTGCCCGGTGCAGGCGGGGTGGAAAATCCAGGCGGTCTGGGCGTTTTTGCCCAAAAAAAGGCGCAACAGGGCGTAGAGTTTCAGGAACAGGCGGGTCAGCGGCATGGGTTCGGGAAAGGGGGTGCAGACGGCATACGGTTGGGTATGGGCGGCAAACCCGATATATTGTTTACGGTCTTATGCTATTATATACCCCGCTCGATTTTCAATCATATTTAGAAAGAACGGATAAATTATGAATCAAGCTGTTGCACAATTTGCTCCTTTGGTGTTGATTATGGTGGTGTTCTACTTCCTGATCATGCGCCCCCAACAAAAGAAATTCAAAGCGCATCAGGCAATGCTTGCCGCCTTGAAAGCCGGCGACAAAGTGGTCTTGGCGGCAGGTTTCAAGGGTAAGGTAACCAGAGTCGGCGAACAGTTTTTTACCGTGGATATCGGACAGGGTACAAAAATCGAGGTCGAAGTGGAACGCAATGCGATTGCCGCAAAAGTCGATTGATTTGTGCCGACAAGCCGCATCGGGAAAGCCCGAATGCGGCACTTTGTTTTGAATTCCAACCGAAGGCTTGACCATGTTCCGACACGCAGGGCGGCATATTCAAGATGCTGCCTTTCGGTCTTGCCTGGCAGGGAGGGGTTTTGCCTCTTCTGAAATAGCCCGATTCCGACACCACCGAAAGGGTGGGGTTCCAACCATTAAGGAACAATGATGAACCGTTATCCTTTATGGAAATATCTGCTGATTGTGTTCACGATTGCGGTTGCCGCAGTGTATTCGCTGCCCAACCTATTCGGCGAAACGCCCGCCGTGCAGGTATCGACCAACCGACAAGCCATCATCATCAACGAACAGACTCAATCCAAAGTGGATGCTGCGCTGAAAAACGCGGGCATTCAGACCGACGGGATGTTTGTTGTGGATAATTCACTGAAAGTGCGTTTCAAAGACACAGAAACGCAGCTTAAAGCGCGCGACGTTATCGAAAACACTTTGGGCGAAGGGTATATTACCGCGCTCAACCTGTTGGCGGACAGCCCCGAATGGATGGCGAAAATCAAAGCCAATCCGATGTTTTTGGGTTTGGACCTGCGCGGCGGCGTGCATTTCACCATGCAGGTCGATATGAAAGCCGCGATGCAGAAAACGTTTGAGCGTTATTCGGGCGACATCCGCCGCGAACTGCGCCGTGAAAAAATCCGCAGCGGCACGGTGCGTCAGGCTGAAAACAGCCTGACCGTCCCTTTGCAGGATGCCGGCGATGTGCAAAAGGCACTGCCTCAGTTACTCAAGCTGTTTCCTGAGGCAACGTTAAATTCAGAAGGCAGCAATATTGTATTGGCGCTTTCGGAAGAGGCGGTGAATAAAGTACGATCAGATGCGGTGAAGCAAAACATCACTACCCTGCATAACCGTGTAAACGAATTGGGTGTGGCAGAGCCGATTATCCAACAGTCAGGTTTGGACCGCATTGTCGTACAGCTTCCCGGCGTGCAAGATACTGCCAAGGCAAAAGACATCATCGGCCGTACCGCGACTTTGGAAGTCCGCATGGTGGAGGACGATCCTGCCAAGTTGCGCGAGGCATTGGAAGGCAACGTGCCGAGCGGTTATGAGCTGCTTTCAAGCGGCGGGGAGCATCCCGAAACTCTGCTGATCAGCAAACAGGTCGAGCTGACCGGCGACAACATCAACGATGCGCAACCGAGTTTCGACCAAATGGGCGCACCTGCCGTCAGTCTGAGCTTGGACAGCGCGGGCGGCAGCATTTTCGGCGAACTGACTGCCGCAAATGTCGGCAAACGCATGGCGATGGTTTTGATCGACCAAGGAAAATCCGAGGTTGTAACCGCACCGGTTATCCGTACTGCCATTACCGGCGGACGCGTGGAAATTTCCGGAAGCATGACGACAGCCGAAGCCAACGATACGTCTTTGCTGTTGCGTGCCGGTTCTCTTGCCGCACCGATGCAGATTGTCGAAGAACGTACCATCGGTCCGTCTTTGGGTAAGGAGAACATCGAAAAAGGCTTCCATTCGACTTTATGGGGTTTTGCCATCGTTGCTGCATTCATGGTGGTTTATTACCGCCTGATGGGTTTCTTTTCTACTATTGCATTGAGTGCCAACATACTGTTCCTAATCGGTATTTTGTCTGCCATGCAGGCAACGTTGACGTTACCGGGTATGGCCGCGCTGGCGTTGACTTTGGGTATGGCAATCGACTCCAACGTCTTGATTAACGAACGTATCCGAGAGGAATTGCGTGCCGGCGTGCCGCCGCAGCAGGCAATCAATCTCGGTTTCCAACACGCATGGGCGACCATTGTTGATTCGAACCTGACTTCGCTGATTGCCGGTATCGCGCTTTTGGTATTCGGTTCCGGACCGGTAAGAGGTTTTGCCGTCGTACACTGCTTGGGTATTCTGACTTCGATGTATTCGTCCGTCGTCGTATTCCGTGCGTTGGTCAATCTGTGGTACGGCCGCCGCCGCAAATTGCAGAATATTTCCATCGGTGCGGTATGGAAACCTGAAGCCGAAACTGCGGCAGGTAAGGAGTAAGCTATGGAACTCTTTAAAATCAAACGCGATATTCCGTTTATGAGCTACGGCAAACTGACAACCTTCATTTCGTTGGTTACCTTTATTGCCGCCGTATTCTTTTTGGTTGCCAGAGGCCTGAATTTCTCTGTCGAATTTACCGGCGGTACGGTAATGGAAGTCCAATATCAGCAGGGTGCGGATGTCAATAAGATGCGCGAACGCCTCGATACGCTGAAAATGGGTGATGTACAGGTTCAGGCATTGGGTACGAACAAACACATCATGATCCGCCTGCCGAACAAAGAAGGTGTTACTTCCGCACAGTTGTCCAATCAGGTTATGGATTTGCTGAAAAAAGACAGTCCCGACGTTACTTTGCGCCAAGTCGAATTTATCGGCCCGCAAGTCGGTGAGGAATTGGTAAATAATGGATTGATGGCTTTAGGTTTTGTCGTTATCGGCATCATTATTTACCTGTCGATGCGTTTCGAATGGCGTTTTGCCGTATCTGCCATTATCGCCAATATGCACGACATCGTGATTATTCTCGGCTGCTTTGCCTTCTTCCAATGGGAATTTTCGCTGACCGTCTTGGCAGGTATCCTTGCCGTATTGGGCTATTCTGTGAACGAATCCGTCGTCGTCTTCGACCGTATCCGTGAAAATTTCCGCAAGCCGGCGATGCGCGGACATACCGTGCCGGAAGTCATTGACAACGCGATTACCGCAACGATGAGCCGCACCATCATTACCCACGGTTCGACCGAGGCGATGGTTGTTTCTATGCTGGTGTTCGGCGGTGCAGCCTTGCACGGCTTTTCTATGGCACTGACCATCGGTATCGTGTTCGGCATCTACTCTTCCGTATTGGTTGCCAGCCCGCTCTTGCTGATGTTCGGTTTGAGCCGCGACAATATCGCCAAGGAAGCGAAACAGAAGGAAGAAATTGTGGTCTGATGTCAAATGCCGTCTGAAACCGGAAGATGTCTCCGTTTTCAGACGGCATTTCGCCGACATCGCAAAATATCGTGCAGAACAGCAAAAATTGTGTGATAATGCGCTGTTCCTGTTTCAGGAATAGGGAGTTTGCCATTGTCGAGGCTTGGCAAACTTGTCCGAATCCCATTTGGGGTTCTTTTTATTTTTCGGAGTTTTTCCATTATGGCACTGACCGTAGAACAAAAAGCACAAATCGTTAAAGATTTCCAACGTAAAGAAGGCGACACCGGCTCTTCTGAAGTACAAGTTGCCCTGTTGACTTTCCGCATCAACGATCTGACCCCCCACTTCAAAGCCAACCCCAAAGACCACCACAGCCGTCGCGGCCTGTTGAAAATGGTCAGCCAACGCCGCCGCCTGTTGGCCTATTTGCGCCGTACCCAGCCCGATACGTATCGCGCGTTGATTACCCGCTTGGGTCTGCGTAAATAATTGCGCTTTCCGACACCGCCCAGAAAAATGGGCGGTGTTTTCTTTTCTGTTGCTTTCCGACAAGCTCAAACCCATATTTATTACCCTAAAAACCTTATAAACTAATATAATGCGGGGTTCTTTAGAACCCCTTTTTATTTCATGCTGCCCGTGCGCTTCACAAGAGTTTCAGACGGCATCAGACGTTGCGACTCCCGCCAGCAATCAAACAGCTTTTTATCACCCATTCGAAAATCCGTTTTGCCGGTACTCGTCTTTTTATTGGAGTATTGCCATTATGACCGCAACCACAGCGTCTTCAGCCAAACCTTATCTGCAAATCCAAGGTTTGGTGAAAAAGTTTGGTGACAATTACGCTGTCGATAACATCGACTTGGACATTTATCAAAACGAAATCTTCGCCCTTTTGGGCAGTTCCGGCAGCGGAAAATCTACGCTGCTGCGTATGCTGGCGGGTATGGAAAGTCCCAATCAGGGAAAAATCATCCTTGATGGTCAAGATATTACCAAACTTGCACCCTATGACCGCCCCATCAATATGATGTTCCAAAGTTACGCGCTTTTTCCGCATATGACGGTGGAGCAAAACATTGCCTTCGGTCTGAAACAGGACAAAATGCCTAAAGGCGAAATCGATGCGCGCGTCGAAGAAATGCTGCGTCTGGTTCAGATGACCAAATTTGCCAAACGCAAACCGCACCAATTGTCCGGTGGTCAGCAGCAACGTATCGCCTTGGCGCGCAGTTTGGCAAAACGTCCGAAAATCCTGTTGCTTGACGAACCTTTGGGGGCGTTGGATAAGAAATTGCGCCAGCAAACCCAGCTCGAGTTGGTCAATACGCTGGAACAAGTCGGCGTAACCTGCATTATGGTTACGCACGACCAAGAAGAGGCGATGACGATGGCGACCCGCATCGCCATTATGTCCGACGGTCAGTTGCAGCAGGTCGGCACGCCCAGCGACGTGTACGACTATCCCAACAGCCGCTTCACTGCCGAGTTTATCGGCGAAACCAACATCTTTGACGGTGTGGTGATTGAAAATCATGCCGACTATGCCGTTATCGAATGCGAAGGTTTGGAAAACCACGTCCGTATCGACCATGGTTTGGGTGGCCCGAGCGAGCAGGATCTTTGGGTTAGTATTCGACCAGAGGATATTGATTTATATAAAGAAAAACCCGAATATTTGGGCGACTACAACTGGGCGAAAGGCACGGTAAAAGAAATTGCCTATTTGGGCAGCTTCGCCATATACCATATCAAACTCGCCAACGGGCGCGTCGTCAAAAGCCAAGTTCCTGCACCTTACTGGTATGTGCGTAACATCACGCCGCCGACTTGGGATGAAAC

>27 |ref|NC_017511.1| Neisseria gonorrhoeae TCDC-NG08107 | Coordinates: 248269,258662 | Forward

GCGGCGTTTTCTGTGCGTTTTAGGGCTTCGGGTAGGCTAGCCCCCAATACTTTGGCGATATTGCTCGGATAGGGCTTTCTCGCGCCCGCAATGCGGGTTTCTGCTTCCGCTACGGCTTCTGCCCCGTAGGTCTCTATCAGCCATGCGGCTGTTCGCCTGTCGCGTTCGTTCTCGGTAATCATCGGCTCATTCCCCATCCCCTGCTTTGGGTTCGTTTGTGTCGTTGGCTTATCGTTTGGCTAGTTGATTCAAGATTTCGCTCTGCCGTTGCTGTATTTCGCTCTGCCGCTCTAACTCGGCTGCCAAGCTCGCTAGCTGCTGCGCTAAACTCGTGTTTTCCTGCTCTAGCTCTGCCAACCTTTCGCCCAAGTGCGTTAAGGCTTTCATCATTCGCTTCTCGGTCGCTACGCATACCCGCGTTGCTTTGCTGTTCTCGACTGGGCAATTTTCCAGTGTCAAACCTTTGGTCTTGGTTTCCAACAGGTCTAGGGTGCGCTCTGCTTCGGCTCTCTGCTGTTTCAAGTCGTCCAGCTCGTTCTTGACGCTCCATATCGCTATGAACAGCCCTGCTATGACTATCAACCCTGCCGCCGATATACCTAGCAAGCTCCACAGATAGGGCTTGAATACTGCCTTGCTCATGCGTAACTGCCGGGCGTTTATATCGGCGGTTATTTTCTGCTCGCTTTGCTTCAATGCCTCGTTGATATTTTTCCGTAACGTCTCTAAGTCTGCTTTCGTTTGTTGCTCTATGCTGGCGGCTTCGCCGCCGACTTGGGATGAAACCGTCTATATCAGCTGGCCGGAAAACCAACCGACTCCGTTGTTCCGTTGATTTAAGGGGAGTGAGATGAACCTTAATAAACTGAAAAACAAACTGTTCCGCCGTCCGGGGCAGCGTGCGGTGATTGCCGTACCGTATATTTGGCTTTTGGTGCTGTTTCTGATTCCGTTCGCCATCGTGCTGAAAATCAGCTTTGCCGAACAAGAAATCGCCATTCCACCGTTTACTCCTTTAACGACGATAGATGAGGACTTGGGTCGCCTGAACATCGCCGTCAGCTATCAAAACTATGCCGATATTTTCCAAAATTTTTGGCATACCCTTAACCCCTTCGGCGACAGTGAAAACAGTAATATCTATCTGATGACTTATTGGTCTTCAATTAAGACTGCGCTGACGACGACGGTGATTTGCCTGTTGGTCGGTTACCCGACCGCCTATGCGATTTCCCGTGCCAATCCTTCCGTCCGCAATGGTTTGCTGCTTGCCATCATGTTACCTTTTTGGACATCGTTCCTGCTGCGTGTCTATGCGTGGATGGGTTTGCTCGGGCATAACGGCATCATAAACAACTTATTGATTAAGATGGGTATTATCAGAGAACCATTAGATTTGTTCTATAATGCGTTTTCGTTAAATTTGGTAATGGTTTATGCCTATCTGCCGTTTATGATTCTGCCGCTTTACACGCAGCTGGTGAAACTGGACAGCCGCCTGCTCGAAGCGGCTTCCGACTTGGGCGCGGGACCGGTCAAATCGTTCTTGACGATTACCCTGCCTTTGTCGAAAACCGGCATTATTGCAGGTTCGATGCTGGTTTTCGTCCCCGCTGTCGGTGAGTTCGTCATTCCCGAGCTGGTCGGCGGTTCGGAAAACCTGATGATCGGTAAAGTATTGTGGCAGGCATTCTTTGATCAAAACAACTGGCCGTTGGCTTCTGCCGTCGCCGTCGTGATGGTCGCGCTACTGGTTGTACCGATTGCCCTGTTCCAGCACTATGAAAACCGCGAATTGGAAGAAGGAGCCAAATAATGCAGAAATCCAAATTATCTTGGTTCTTGAAACTGATGTTGGCACTATCGCTGGCGTTTCTGTATATCCCGCTGGTTGTTTTGGTCATCTATTCGTTCAACGAATCCAAACTTGTTACCGTTTGGGGCGGCTTTTCGACCAAGTGGTACGGCGCATTGCTGGAAAACGACACCATTTTGGAAGCCGCTTGGCTGTCGCTGCGGATTGCCGTTGTGTCTTCGCTTGCCGCCGTCGTTTTGGGCACGCTGGCGGGCTATGCGATGGCGCGTATCAAACGCTTTCGCGGCAGCACGCTGTTTGCCGGTATGATTTCCGCGCCCATGGTCATGCCCGACGTGATTACCGGTCTGTCTATGCTGCTGCTGATTATTCAGGTGCAGATATTTTTGCAGGGCAGCGAATGGTTGCAACATCTCTACTTCGATCGCGGCTTTTTCACCATCTTCCTCGGACATACGACGCTGTGCATGGCATACATTACCGTTGTCATCCGTTCGCGGCTGGTCGAGCTTGACCAGTCGCTCGAAGAAGCCGCAATGGACTTGGGCGCGCGTCCGCTGAAAATCTTTTTTGTCATCACTTTGCCTTTGATTGCCCCTGCCATTGCTTCAGGCTTCCTACTCGGCATTACCTTGTCTTTGGATGATTTGGTGATTACCTCATTCCTCTCCGGCCCCGGTTCATCCACATTGCCGCAGGTTATTTTCTCCAAGATCAAGTTGGGTCTCGATCCTCAGATGAATGTCTTGGCAACCATCCTGATCGGCATCATCGGAACATTGGTCATCATCGTCAATTATTGGATGATGAGGCAGGCAACCAAGCGCAACCGAGAAGCGGCAGAAGCCTACCGCCAAGAAAAATTGGCTGCCGAGAAAGCAAATTAATTAATAAGGCAGGCTGACCGCATGACTGGGTCAGCCTGTTTTTTTTCAACCGATTTTCTGTTTGGACGATATGGCCCGACAGCCTGTATCATTCCGTCCGAAAATATACCCGATAAAGCAAACACAATGATTCGCCCTGATTTTCAAGAATATCTGCCTTCTTATTATTTTAGTTCGGTTAATCCGCATACTGTTTATCCGAAACTCCAATGCCGTCTGAAAGCCGAAACCTGCATTATCGGCGGCGGTTTGAGCGGTTTGTGTACCGCATTGCCGCTGGCGGAACACGGACATGAAGCCGTCGTTTTGGAAGCCGCACGTATCGGCTTCGGCGCGTCCGGCAGGAGTGGCGGGCAGGTTATCAGCGACTACGCCTGCGGTATGGGGGAAATTGAAAAACAGGTCGGCTTGGAGCAGGCGCAATGGTTTTGGCAACAGTCTTTGCAGGCGGTCGAACTGGTGGACGAACGCGTCCGCAAACATGCCATCGATTGCGATTGGCAGCGCGGTTATGCCACGGTTGCCGTCCGTCCGCAGCATTGGGAAGAGTTGCAGCAGTGGCATGAACACGCCCAACGGCATTACGGTGCGAGTCATTATCAACTTTGGGATAAAGCCGAGTTGAAACAGCAGCTTGACAGCGATATGTACCAAGGCGCACAGTTCGATCCCTTGTCCGGGCATCTTCATCCGCTCAATTACACGTTGGGTGTTGCCAGCGCCGCTGCCGAAGCCGGCGCGCAGATTTTCGAGCAATCCCCGATGACGCGCATCGAACCGTATCAAAACGGTTGGCTGGTTTACACGCCCGAAGGCAGCGTCGAGTGTAAAAATGTGGTCTATGCCGTCAATACTTATGTCGGTTTGAACCCGATATTCCGGCCTTTGGAACGCAAGGCGATTGCCGTCAGCACCTTTATTATTGCCACCGAACCCTTGGGCGCGCGCACAAAAGGGCTTATCCGCAACAATATGGCAGTATGCGACAACCGCCATATTTTGGATTATTACCGCCTCAGCGCGGACGGCAGGCTGCTTTTCGGCGGTAAGGATAACGAATTTATCGACAATCCTGCGCGTATGACCGAGCTTGTCCGCCAAGATATGCTTAAAGTTTTTCCGCAGCTTGCCGATGTTAGAATCGAATATTCGTGGGGCGGGGAGTGCGATATTACCGCCAACCTTGTCCCGCATTTCGGACGTTTGACCTCGAATGTTTTTTATACGCAAGGTTATTCCGGGCACGGGATGGCGATAACGGGCATTGCAGGCCTGGCGGTTGCCGAAGCAATTTTAGGGGACGAATGCCGTCTGAAACCGTTTGAACAGTTGCGCCAGCCGAATATTATCCTGCAACCGTTTTTGCGCAAACTCGGTTCTTTCCTCGGCTCGAAATATTATCAGTGGAAAGACAGCCGTTAAGCGTCGCAGGCAGTATGTTTATCCCCATCGGCGGCAAACGTGAAAAATGCCGTCTGAAACCCGATTTTCAGGCTTCAGACGGCATAGCCGTCCTTATTCCACGTGTTCGCCGTGGATATTCAGATCCAAACCTTCGCGTTCGACATCCTTGCCGACGCGCAGACCGCCGCAGATTTTCCCCACAGCCTTCAAAATCGCCCAACTCATCAGCCCGCTGTATGCCGCCATAATGAACCCGTCTTTTACCTGTATCCACAACTGCTGCCAAACTGCCGCATCCCCACCGAAAATGCGGTTGTCGAAAAAGATGCCGGTCAATATTCCGCCTACCAGCCCGCCGAATCCGTGTATGCCGAAAGCGTCCAAAGAATCATCGTAACGCAATTTGTGTTTGACGACGGTGACGGACACAAAGCACGCGGCGGCAGTCAATATACCGATGGCGGCCGCGCCCGACGGACCGGTAAAGCCGGCGGCAGGGGTGATGCCGACCAGCCCGGAAACTGCGCCGGAAGCCAGCCCCAAAGCAGAAGGTTTGTGTCCCGCTATTTTCTCGCAGGCAAGCCAGCCTGCCGCGCCGAATACGGCCGACACCTGCGTTACCGCCATCGCCATCCCCGCCGCCGCGTCTGCCGCAAGCGCCGAACCAGCGTTAAAGCCGAACCAGCCGAACCACAACATTGCCGCGCCAATCAGCGTCATCGCCATATTGTGCGGAGGCATCGCCTCGCGCCCGTAGCCTATGCGCCTGCCCAAAACCAAGGCGGCGACGAGTCCCGCGATACCGGCATTGATGTGCACCACCGTACCGCCGGCATAATCCAATACGCCGCCCTTGCTCATAAAGCCGCCGCCCCACACCCAATGCGCGCCCGGCACATAAACCAATAAAAACCATATGCCCGAAAACAGCATCATTGCCGAATATTTCATTCGTTCGGCAAACGCGCCGGTAATAATGGCAGTCGAAATAATGGCAAACGTCATCTGAAAAAACATAAATACCGGTTCGGGAACAGTCGGCGCATTGGGCGACACGGTCAGCATCCGTGCGGCAGCGTCTATCTGCATCCCGCTTAAAAATACGCGCCCCAAACCGCCGATAAAGGCATTTCCCGGCGTGAACGCCAAAGAATAGCCGACGGCGACCCAAAGGATGCCCACCAATGTCGCGATGGAAAAGCTGTGCATCATCGTCGAGAGCAGGTTTTTTTTCCGCACCATACCGCCGTAGAATAAAGCCAGCCCGGGAAGCGTCATCAACAGTACCAAGGCAGCCGCAGTCATCACCCAGGCGGTATCGCCCGAATTGACGGCGGAATAAGGTTTCCACCAGTTTAAAGGTTCTGCCGATAGGGATGCCGGCAGCAAAGATGCCGCCCATATGTGTTTTTTCATTTTGATTAAAGTTTCCTTAATGGTTGGGCCCGTCTTTCGGAAAGGCGGGGTCGGGGCTTGTCCGGGATGGGCGCAAGCCCTGCCGGACCGGCGCGGGGATTTTGCCGATGTGCCGCCCTGTGCGTGAAACAAATCCCTTGTTTGAATATGGAAATATCGCATCCTATCCCTTGCCCCCGTTGTCCGGCGGGAGGATTTATCCTTAGGCGGCGCATATGCGGGCGTATGGATTGTCAACAATTTACTGTAGGAAAATATACAGAGGTTTGGGCGATAAGTCAAAAGATTGTTGACAATATTTTTATTTTATAAAATTAATTTATTGATTAATATATTAAAAATTTCTAATTGGAAATAAAAAATAAAATTTATACAAAAATGGGCGCGGTTCGGCGCAACCTTGAATCAAGTTCCCGCATCGGTTTTCATTGCCGGTAGGGATGCGTTCGAGCCGGTTTTGCAAAGGCCGCGCCTTCGGCAGGCGGACACGGACACTGCCGGCGGTTGCGCCGTTAGCGGGGGGGGGAGCTGCGCCGGCCGTGCGAATGAAAATGTCGTCTGAAACCCGATTTTCAGGCTTCAGACGGCATTTCGCATTAATGCGGGCGGCGCGTTTATTTTCCGCGCATCAGTTCAAAGAAATCATCATTATTTTTAGATGCTTTGATTTTTCCGTTTAAGAACTCGGTAGCTTCGATTTCGTCCATCGGGTGCAGGAACTTGCGCAGTAACCACATACGTTGCAACTGGTCGTTCGGCACAAGCAGCTCTTCGCGGCGCGTGCCGGATTTGTTGATGTTGATGGCGGGGAAGAGGCGTTTTTCCGCCATACGGCGGTCGAGGTGCAATTCCATATTGCCGGTACCTTTGAATTCTTCGTAAATCACATCGTCCATACGGCTGCCGGTTTCAACCAATGCGGTGGCGATAATGGTCAGCGAACCGCCTTCTTCCACGTTGCGCGCCGCACCGAAGAAACGTTTGGGGCGGTGCAGCGCGTTGGCATCGACACCGCCGGTCAGGATTTTGCCCGAGGCGGGGACGACGGTATTGTAGGCGCGGGCAAGGCGGGTAATCGAATCCAGCAGGATGACCACGTCTTTTTTGTGTTCCACCATACGCTTGGCTTTTTCAAGCACCATTTCGGCAACTTGGACGTGGCGTTGCGCCGGTTCGTCAAAGGTGGAGGAGACTACTTCACCTCGGACGGAACGGCTCATTTCGGTTACTTCTTCGGGGCGTTCGTCAATCAAGAGGACGATGAGTTCGACTTCAGGATAGTTTGCGGTAACGGCGTGGGCAATGTTTTGCAGCATCACGGTTTTACCGATTTTGGGCGGGGCAACCAAGAGGGCGCGCTGACCTTTGCCGATGGGCGAAATCAGGTCGATGGCGCGTCCGGTCAGGTTTTCTTCGGACTTTAAGTCGCGTTCCAGCTTCAACTGTTCGGTCGGAAACAGCGGGGTCAGGTTTTCAAACAGGATTTTATGGCGGCATACTTCCGGGTGGTCGCCATTGATGGAATCCAGTCTGACCAAGGCAAAATAGCGTTCGTTGTCTTTGGGGACGCGCACGCTGCCTTCGATGGTGTCGCCCGTATGCAGGTTGAAGCGGCGGATTTGAGTGGGCGAGACATAGATGTCGTCGGGGCCGGCAAGATAGGACGTGTCCGCGCTGCGGAGGAAGCCGAAGCCGTCGGGCAGGATTTCGAGCGTGCCGGAGCAGGTAAAACCCTCGCCTTTTTTCATCATCTGGCGGACGATGGCAAATACGAGGTCTTGTTTGCGGAATCGGTTGGCGTTCTCGATGCCGTGTTCTTCCGCCAATTCTAAGAGTTTGGAAATGTGCAGGGTTTGTAATTCGGAGACGTGCATAATCAATGATATATAGAATAGAAAGGAAAAGACGGGTGGATGCCGTCTGAAAGAAGAAGCTGACTGTTGCCGGTTGCTCGGAGAGGGGGGAATTGTAGGCAGTCGGCGCGTGGGTGTCAAATATTATCGCGGACGGGGCATCGACAGGAAATGCCGTCTGAACGGAGCTGCTTGGAAAAAATACCCCCGCGCTTTTCAGGCTTGGGGGTATGGGAATTGATTATTTGTTCAATTCATTCGCCAAATACAGCCAAGTTTCGATGACGGTATCCGGGTTCAGAGACACGCTTTCGATGCCTTCTCCAACCAGCCATTTGGCAAAGTCCGGATGGTCGGACGGACCTTGACCGCAGATACCGACATATTTGTTCTGCTTGCGGCAGGCGGAGATGGCAAGATGCAGCATCACTTTGACGGCAGGGTTGCGTTCGTCAAACGATTCGGACACCAAGCCGCTGTCTCGGTCGAGGCCGAGGGTCAGCTGGGTCATGTCGTTCGAGCCGATGGAGAAGCCGTCGAAGTATTGCAGGAATTGTTCCGCCAATACCGCGTTGCTCGGCAGCTCGCACATCATAATCAGGCGCAGGCCGTTTTTGCCGCGTTCCAAGCCGTTTTCTTTCAATGCCTTAACCACTGCTTCGGCTTCGCCCAAAGTGCGGACGAACGGAATCATGATTTCGACGTTGGTCAGACCCATTTCGTCACGAACGCGTTTCAAGGCTTTGCATTCCAAGGCGAAACAGTCTTTGAAGCTCTCGGCAACATAACGCGCCGCACCACGGAAGCCCAACATCGGGTTTTCTTCATGCGGTTCGTATACGCTGCCGCCGACCAGGTTGGCGTATTCGTTGGATTTGAAGTCGGACATACGGACGATGGTTTTACGCGGATAAACCGATGCGGCAAGCGTTGCCACGCCTTCGGCGATTTTATCGACGTAGAAGTCGACAGGGGATGCGTAACCGGCGATGCGGCGGATAATTTCCGCTTTCAGTTCGTCGTCTTGTTTGTCAAATTCCAACAAGGCTTTCGGGTGGATGCCGATTTGGCGGTTGATGATAAATTCCATACGCGCCAAGCCGATGCCTTCGCTGGGCAGATTGGCGAAGCTGAATGCGAGTTCGGGATTGCCGACGTTCATCATGACTTTGACGGGTGCTTTTGGCATATTGTCCAAGGCGACATCGGTAATTTGTACGTCCAGCAGGCCGGCATAGATAAAGCCGGTATCGCCTTCGGCACAGGATACGGTAACTTCCTGACCGTTTTCCAAGAGTTCGGTCGCATTGCCGCAGCCGACGACGGCAGGAATACCCAGTTCGCGCGCGATGATGGCGGCGTGGCAGGTGCGTCCGCCGCGGTTGGTCACGATGGCGGAAGCACGTTTCATCACGGGTTCCCAATCCGGATCGGTCATGTCGGTAACCAGTACGTCGCCGGCTTCGACGGAATCCATCTCGGAAGCATCTTTAATCAGGCGCACCTTGCCCTGACCGACTTTTTGACCGATGGCACGACCTTCGCACAAGACGGTTTTTTCGCCGTTGATGGCGTAGCGACGCAGGTTGCGGCTGCCTTCTTCTTGGGATTTGACGGTTTCGGGGCGGGCTTGCAGGATGTAGAGTTTGCCGTCCAGGCCGTCGCGTCCCCATTCGATATCCATCGGGCGGCCGTAGTGTTTTTCGATGGTCAGCGCGTAGTGTGCCAACTCGGTGATTTCTTCGTCGGTAATGGAGAAGCGGTTGCGGTCTTCTTCGGGGACTTCGACGTTGGTTACCGATTTGCCGGCTTCGGCTTTGTCGGTGAAAATCATTTTGATGTGTTTCGAACCCATGGTCTTGCGCAGGATGGCGGGTTTGCCTGCTTTGAGCGTGGGTTTGAACACATAAAATTCGTCCGGGTTGACCGCGCCTTGTACGACGTTTTCGCCCAGACCGTAAGAGGAGGTAACAAAGACGACTTGGTTGTAGCCGGATTCGGTGTCGAGGGTGAACATCACACCTGATGCGCCGCTGTCGGAACGCACCATGCGTTGAACGCCGGCGGAAAGGGCGACGATGTCGTGTTCGAAGCCTTTGTGGACGCGGTAGGAAATGGCGCGGTCGTTGTACAGGGAGGCGAAAACGTGGCGCATCGCTTTTTTAACGTTATCCAAGCCATTGATATTCAGGAAGGTTTCCTGTTGGCCGGCAAATGATGCGTCGGGCAGGTCTTCGGCGGTTGCGGAAGAGCGTACGGCAACGGAAATGTCCGCGCCGTCGGCATCGGTAACCATTTTGTTCCATGCCGCTTCGATTTCGGCATCGAGCTGTTCGGGGAAGGGCGTGTCCAAAATCCATTGGCGGATTTCTTTGCCGACGCGTGCCAGTTCGGAAACGTCTTCGACATCCAATTTTGCCAGAGCGGCGGAAATGCGTTCGTTCAGACCGTTGTGTGCGAGAAATGCGCGGTAGGCATCGGCTGTGGTGGCAAAGCCACCGGGGACGCGAACGCCTTTTTCGGTCAGTTGACTGATCATTTCGCCCAGCGAGGCGTTTTTACCGCCCACGCGTTCAACATCTGTCATACGCAGGTTTTCAAACCAGATTACGTAGTTGTCGGCCATTTGTGTGTCCAATCCAAAATATGTTAAAAAAGAAACAAATCCGCTTGCTTATTTTAAGCGATTCGTTCCGCCGCTGTCACGTTTTTATCTGTCCGGACAACCGCTGCGCCGTCTGAAAAATAGCGGGTTCCGGCTGTGTAGCGGTTTGAAACCGATGGCAGGCATATTGTTTTTTTCGGGTATTTCCTTTGTAAAACAGATGGTTTTGAATAGGTTAATGTTTTATGCCGTATTTTTCCTGTTTCTTTTTTTTGGAATTTTATTTTAAAGAAATCTGATTTTGAGATAATTTGTTTTAAAATATGATTTGTTTTTGGGTCTGTACCAGATTAGCAGATATGTTGCCCTCGAAATATGAAGATAACACACTGCAAATTAAAGAAAGAAGTACA

>28 |ref|NC_017511.1| Neisseria gonorrhoeae TCDC-NG08107 | Coordinates: 258663,283433 | Forward

CGGCACACCGTCCCGGCAGCTAAAAATCCTGCGGGATCGGTGTGGAATTTAGGGATAATCTAGTACAGCCCCTTGTTTTTTGGGGTGTAATCCGAGGTAGGGGCGGCTGGGGTGCTTCTCCCTTGTCTGCCGCTGCTGTTATGATGATATTTTTATTCTGTATTTAAGGAGGGGGTGATGAGCAGTCCGCGCCAAGTGTTTTATATTTCCGACCGCACCGGTCTGACTGCCGAGAATATCGGCGAGGCTTTGCTGAACCAGTTTGGCAATCTGTCGTTCAAACGCCATACGCATCCGTTTGTCGATACGCCGGAAAAGGCGCGCGCGGTGGTGGAGAAGGTCAATCGGAGCCGGCAGGAAAACGGTCAGCGTCCGATTGCGTTTGTCAGCGTGGTTGATGACGAAATCCGCCGGATTATTAAAGGGGCGGATGCTTTTCAGATTAATTTCTTTGAGACTTTTTTGGGACTGTTGGAGAAGGAACTCAATACCGAAGCAACGGTATCCGGGCAGGGGCATCACAGTATCGGCAATACGAAGCGTTACGATGCGCGTATGGAGGCGGTCAATTTTTCTTTGAACCACGATGACGGGGTCAGCGATAAGAACCTTCAGGAGGCGGATGTGATTCTGATGGGCGTGTCCCGTTCGGGCAAAACGCCGACCTGCCTGTATCTGGCGTTGCAATACGGTATCCGTGCGGCAAACTATCCGCTGATTCCCGACGATTTGGAATCGGCCGATCTGCCGCGTATGGTCAAGCCGTATAAAGACAAACTGTTCGGGTTGACTATCCAGCCGGAACGTTTGCAGGCCATCCGTCAGGAACGCCGCCCGAATTCGGCTTATGCGCGCATCGACACCTGCCGCAGCGAAGTGGCGGACGCGCAGAGTATGTTCAGACGGCATGGCATTCCGTTTGCGAATACGACGGACAAGTCGGTTGAGGAACTGGCGGTACACATCCTTCAGGCGTGCAAGCTCAAACGCAGGTTTTGACGGGCTTTGATTCGGTTTGAAGGTGGAAATGCCGTCTGAAATCAGGTTTCAGACGGCAGTTTTATGTTTGTGGGGCGGATATTTTTCAGGGCTGTATTTTGTCCAGACATTCGAGCAGATCGAGCGGCGTGCGGATGTGGAAATCCGCCTGCCATGAGCCGGTATCGTCTTCATCGGAAATATAGCCCCATTCGGCGAGGACGGTTTTCATACCGGCGTTGCGTCCGGCTTGGATGTCGCGTTCCGCGTCGCCGACGTAGAGGGTGTGTTGCGGGTCGGCGTGGATTTTTCCGCAGGCGTGCAGCATGGGTTTGATGCTGGGTTTGGGTTCGCCGCAGGTGTCGCCGCTGACGACGGTGGCGGGCGGAACGGCGAATCCGAGTTTGGGAACGAGTTTGTCGGTGAAGCGCATGGGTTTGTTGGTGATGATGCCCCATTTGATGCCGCGTCTGCCGAGTTCGGCGATGAGTTCGTTCACGCCGTCGAAGAGAGTGGTGTCTTGGGCGTAGCGGCTGTCGTATTCCTCCAGATATTCGGTGCGCCATGCGGTATAGTCGGGGTGTTCGGGCGTGATGTTCGCGCCGAGTTTGAGCAATCCTGCCGCGCCGTGGCTGGCTTGGGTGCGGATTTCGTCCATGCTTTTTTCGGGCAGTCCGTGGCGGGCGAGTTGGGTGTTGAGTGCGCCGCCGAGGTCTAGGGCGGTGTCGGCGAGCGTGCCGTCGAGGTCGAACAATACGGCTTGTATCATGTGTTTTCCTTTTTTATAAAGTGTGGGACGAAAGGTTTCAGACGGCATGTTTATTTTGTTTCAAACCCTGCTCGAAATCTTCCAACATATCCAATTCGAAGCGGCTGAAGCCCGCTTTTTCCCGCGCTTCGATGTTCACATAGCCGCGGAAGATAAACATATCGTAACGGGCAATCAGACTGCGGAACAGGGCGACAGGTTCCAAACCGCGTTCGCGGCAGAGGTGTTGATACCACCGGTTGCCGATGGCGACGTGTCCCACTTCGTCGCGGTAAATGATGTCCAACACGCCGCAGGTTGCCGAATCGCCGCGCTGCTCCACCTTCGCGCGTATCCCCGGCGTAACGTCCAGTCCGCGCGCCTCCAACACGCGCGGTACCAACGCCATACGCAACAGCGGGTCGTAGGCGGTTTTGTACGCCATATCCCACAAATGGTTGTGCGCCTCGAAACTGCCGTAATCGAAGCCGAAAGCGCGCAATCTGTCGCGCACCAGGCGGAAGTGGTACACCTCCTCCTTCGCCACTTTCACCCAGTCGCGGACAAACTGAAACGGCAGCGTGCGGAAACGGTATGCCGCGTCCAAAGCCAGATTGACGGCGTTGAACTCGATATGCGTAATCGCGTGCAGCATCGCCGCATAGCCTTCTGCCGTGTTCATTTTGCGCGGGGTCAGTTGCGAGGGCGCGACCAAAACAGGCGTGTCCGGCCGTCCCGCGCGGGGGAAGTCCGCCGGCGGCGCGTTTGCCTCCGCCCCGTCCGCATTTTGAACGGCGGCAAACGCCTCATCCGTCAGCCGTCCTTTTTCATCGGGGTCGCCCGAAAGCAGGGCGCGTTCCGGCAAAGCATAAATATCGGGTTTCATCTCAAGTCCGCCGTGTTCGGAAAACGAATATTATAGCGTTTAAAAAAAACAAGATGAGGCATATAATCTCCGCGATTCGGCATTCCGCGCCCAAACCGTCAAATATAGTGGATTAACAAAAACCAGTACGGCGTTGCCCCGCCTTGCCGTACTATTTGTACTGTCTGCGGCTTCGTCGCCTTGTCCTGATTTTTGTTAATCCACTATAACGCGGCATACAATTGAAAGGGCAGCGTGGCGCGCCTGCTTTTTCCGAGCGGTCAAAAAAATCAGCCCTCGGAAAACGCGGTTTGCAAAATGCAAACCGCCCGTAACGCCGCCCGTATGATTGTTTTGCCGCGCCGATACTTTGCGCCACACCCATCCCGACAAGGAAAAATAATGATGAAACCGCACAACCTGTTCCAATTCCTCGCCGTTTGCTCCCTGACCGTCGCCGTCGCTTCCGCACAGGCGGGCGCGGTGGACGCGCTCAAGCAATTCAACAACGATGCCGACGGTATCAGCGGCAGCTTCACCCAAACCGTCCAAAGCAAAAAGAAAACCCAAACCGCGCACGGCACGTTCAAAATCCTGCGCCCGGGCCTCTTCAAATGGGAATACACTTTGCCCTACAAACAGACTATTGTCGGCGACGGTCAAACCGTTTGGCTCTACGATGTTGATTTGGCACAAGTGACCAAGTCGTCCCAAGACCAGGCCATCGGCGGCAGCCCCGCCGCCATCCTGTCGAACAAAACCGCCCTCGAAAGCAGTTACACGCTGAAAGAGGACGGTTCGTCCAACGGCATCGATTATGTGCGGGCAACGCCCAAACGCAACAACGCCGGCTACCAATACATCCGCATCGGCTTCAAAGGCGGCAACCTCGCCGCCATGCAGCTTAAAGACAGCTTCGGCAACCAAACCTCCATCAGTTTCGGCGGTTTGAATACCAATCCCCAACTCTCGCGCGGCGCGTTCAAGTTTACCCCGCCCAAAGGCGTGGACGTGTTGAGCAACTGATGCCGGCCGCCCCGATGCCGTCTGAACGCCGCCGAGGCTTCAGACGGCATTTTCACGCAGGCGGAACAATGTCCCGCATTATCGGCCGATCGGGCAACGGAACGGCAAATCCGTGAAAATTAACGGTTGCGCCCCGGCTGTTTTTGCCGTTTAATGCAAACCTTGCTGCACCAAGGGCCAAGAAAGCCGACCGGCCGCCCCCACAGCTTCCGATGCAGGCGGCCCGTCCGTCCCTGCAATGTTTTTTATTTTTGAACGAAAGGTCGAAAACCATGAAAAAAACACTGGTGGCGGCAATCCTGAGCCTTGCCTTGACTGCGTGCGGCGGCGGAAGCGATACCGCCGCCCAAACCCCCTCCGCCAAGCCCGAAGCCGAACAATCGGGCAAACTCAACATCTACAACTGGTCGGATTATGTCGATCCCGAAACCGTCGCCGCCTTTGAAAAAGAAACCGGCATTAAGATGCGTTCCGACTATTACGACAGCAACGAAACACTGGAGGCAAAAGTCCTGACCGGCAAATCCGGCTACGACCTGACCGCGCCGTCCATCGCCAACGTCGGCCGGCAAATCAAAGCGGGCGCGTATCAGAAAATCGACAAGGCGCAAATCCCCCATTACGGCAACATCGATAAAGATTTGCTGAAAATGATGGAAGCCGTCGATCCGGGCAACGAATACGCCGTCCCCTATTTCTGGGGCATCAACACCTTGGCAATCAACACGCGGCAGGTGCAAAAGGCATTGGGTACGGACAAGCTGCCCGAAAACGAGTGGGATTTGGTGTTCAAACCCGAATACACCGCCAAACTCAAATCCTGCGGCATCAGCTATTTCGACAGCGCAATCGAACAGATTCCCTTGGCGTTGCACTATTTGGGCAAAGACCCCAACAGTGAAAATCCCGAAGACATCAAAGCCGCCGTCGATATGATGAAAGCCGTCCGAGGCGACGTGAAACGCTTCAGCTCTTCCGGCTATATCGACGATATGGCGGCGGGCAACCTGTGTGCCGCCATCGGTTACGGCGGCGATTTGAACATTGCCAAAACCCGTGCCGAAGAAGCCGCAAACGGCGTGGAAATCAAAGTATTGACCCCGAAAACCGGCGTAGGCGTGTGGGTGGATTCCTTTATGATTCCGCGCGACGCGCAAAACGTTGCCAACGCCCACCGCTATATCGACTACACGCTCCGGCCCGAGGTGGCTGCGAAAAACGGCAGCTTCGTTACCTACGCGCCCGCCAGCCGTCCCGCGCGCGAGCTGATGGATGAAAAATACACCTCCGACGCATCGATTTTCCCGACCAAAGAACTGATGGAAAAAAGTTTCATCGTATCACCCAAATCCGCAGAATCCGTCAAACTGGGCGTGAAGCTGTGGCAAGGGCTCAAAGCGGGCAAATAACCGGAATCCCTGCCGTCTGAAACCTTTCGGGCGGCAGGAAACGGCGTGTCCGCATTAAGTCAGGATTAGGATTATTTAGAAAGATGAGATGGATATGAATTTAAGTATTGTCGTTCCTATTTATAATGTCGAAAGTTATTTGGAAGCGTGTTTAAATTCTATAGAACCTATATTAAGTAATGAAAATGTCGAACTTATTCTTGTGAATGACGGGTCAAAAGACGGAAGTGAAGATATATGTTATAAATATATAGATAAAATATCAAACACCAAACACCAAACACCAAACACCAAACACCAAACACCAAACACCAAACACCAAACACCAAACACCAAATACCAAATATATATATCAGGATAACCAAGGATTGTCGGAGGCGAGAAATACCGGAATAAAAAATTCAAATGGAAAATATATAGCATTTATTGATTCGGATGATTTTATTAATTGTCAGGTTTTGCTGGATTTTCTTGGTAAAGATGATAGTGATATGCCGGATGTGGTTTTTTTAAATGCAGTTAAATATGATAAGGGAAGAGTTTCATATTTTGGCGAAGATTATCAGCCTGAAAAAATATTAAATCAATCCAAAGTCGAAGTTTTGAAAGGATTGTGCCGATTTAGAAAATTTCCGGGTTCGGCGTGTAATAAGATTATAAAAAGAGAATTGATTATTAAAGAAAAACTATTTTTTGAAAAAGGAATTTATGCTGAAGATATCGAATGGTCAATGAGGTTGTTTAATGCGGCAACAACTTTTTCTTATTTGGACGGTTGTTATTACTATTATCGGCAGGGAAGAAAAGATTCTATTACGGGAACTGTTTCGGAAAAAGGTATAAAGTCATTATTATATATTTTGGAGAAAAATGCGGAAATGGAATTTAATAGGGATATATCGAGTTATCTTTATTCTTTTCTTTCCTACGAATATCTCGTTTTGCTTTTTATAATGACAAGTAAAAATATAGCGTGTGATTCTGATATAAAAAGAAGGGCATATCATTTAAGGTTTATGCTGTTAAAGTCCAATAAGCTGATATATAAGCTGATATTCCCGATAATCACATTATTCGGGGTCGATATTACAGGCAGGATTTTAAAAGCAATCAGGGGGAATATTTAATAAATCCTTTAACAATATATACCTTACCGAAGGAGGAAAAATGAACGCAATCAGAACTTTCCAAAACCGCACGCCCGAAATCCACGAAACCTGCATGATAGACGAGGCGTGCGTCGTCATCGGCGAAGTCTCGCTTGCCGAAGATGTTTCCGTGTGGCCGTGCGCCGTGTTGCGCGGCGATGTGAACAGCATCACCGTCGGCGCGCGCAGCAATATACAGGACGGCAGCGTCCTGCACGTTTCCCACAAAACCGCCGCCAAACCCGAAGGGTCGCCGCTGGTTATCGGCGAAGACGTTACCGTGGGGCACAAAGTGATGCTGCACGGCTGCCGAATCGGTAACCGCGTCTTGGTCGGTATGGGCAGCACCGTTTTGGACGATGCCGTTATTGAAGATGACGTGATGATCGGCGCGGGCAGCCTCGTTCCGCCGCGCAAACGTTTGGAGGGCGGCTATCTTTATATCGGTTCGCCTGTGAGGCAGGTTCGTCTGCTGACCGATGAGGAAAAAGCCTTTTTGAAATATTCCGCCGCACATTATGTGAAGCTGTCGAAACAGTACGGGATGTGAAATCACATCGGCGTTCTTGCGTCAGTCCCAAATTCACGCGGTTGGGATGCCCGATAACGGTATCCGATGCGCCTTGATTTTGACCGGCTGCGTTTGAGTTGCAGGCAAAAATGCCGTCTGAAAGCCTTTTTTTCGGCTTCGGACGGCATTTTATTGCCGATTTCTTTTTAGAGTTTGACCGAATGTTCGCGCGTTTCGTGGAACACGATGTCCGGCCAACGTTCTTGCGTCAACCCCAAATTCACGCGGTTGGGGGCGAGGTAGGCGAGGTTGCCGCCTGCGTCGATGGCGAGGTTGCCTGCGTTGGCTTTTTCAAATTCCGCCAGTTTTTTCTTGTCGTCGCACGATACCCAGCGCGCCGACCAGATGGATGCGCTGTCGAACACGGCTTCCACGCCGTATTCGTTGGCGAGGCGTGAGGTTACGACTTCAAACTGCAACACGCCGACCGCACCCAAAATCAAATCCGCGCCGCTCATCGGTTTGAATACTTGAACCGCACCTTCTTCGCCGAGTTGTTGCAAACCTTTTTGCAGTTGTTTGATTTTCAGCGGGTTTTTGATGCGGACGCTGCGGAACAGTTCGGGTGCGAAGAATGGGATGCCGGTAAACGCCAGTTGTTCGCCTTCGGAGAAGCTGTCGCCGATTTGGATGTTGCCGTGGTTCGGGATGCCGATGATGTCGCCGGCGTAGGCTTCTTCCGCCAGTTCGCGGTTGTGCGACATGAAGGTTACTACGCTGGAGGCGGCGATTTCGCGGTTGATACGCAGGTGTTTCATCTTCATGCCGCGCTCGAATTTACCGGAGCAGACGCGCAAGAAGGCGATACGGTCGCGGTGTTTCGGGTCCATATTGGCTTGGATTTTAAAGATAAATCCGGAAAATTTCGGCTCGTCCGGCCCGACCATGCGCATGGTCGCGTCGCGCGGTTTCGGTGCGGGTGCCCAGTCAATCAATGAATTGAGGATTTCCTGAATGCCGAAGTTGTTAATCGCAGAGCCGAAGAACACTGGCGTGAGTTCGCCGGCGAGAAATTCGTCGAGATTAAATTCGTTGGAAGCCGCCTGCACCAATTCGATTTCGTCGCGCAACTGCTGGATTTCCAACGGAAAGCGTTGTTCCAATTCGGGATTGTTTATGCCTTTGATGATGTCGAACTCGTGCGGCAGGCGTTCGCCGCCCGCTTCAAAGAGATAGATTTCGTCGTTCAGGATGTGGTACACGCCCTTGAAGTTTTTGCCCATACCGATCGGCCAGGTAACGGGCGCGCAGCGGATTTGCAGGATGTCTTCCACTTCGTCCAAGAGTTCCAAAGAATCGCGCACTTCGCGGTCGTATTTGTTCATGAAGGTAACAATCGGCGTATCGCGCAGGCGGCAGACGTTCAAGAGTTTGATGGTTTGCGCTTCCACGCCTTTTGCCGCGTCGATGACCATCAAGGCGCTGTCCACTGCGGTTAAAACGCGGTAGGTGTCTTCGGAGAAGTCTTGGTGTCCCGGCGTGTCCAAGAGGTTGACGGTGTGGTCTTTGTAGTCGAACTGCATCACGCTTGATGCCACGGAAATGCCGCGCTGCTTCTCGATGTCCATCCAGTCGGAGGTGGCGAATTTGCCGGTTTTCTTACCTTTCACCGTGCCTGCGCTTTGAATCGCGCCCGAAAACAGCAGCAGTTTTTCGGTCAGCGTGGTTTTACCCGCATCGGGGTGGGAGATGATGGCAAACGTGCGGCGGCGGCGCACTTGGTCGAGGATTTCTTGGGACATGGTTTTCTTTGCAAAAAGGTTCAGGCCGCTTTTCAGACGGCCCGGACAGTGTTTGAGACGGCGAAATTGTACAAAAAAATGCCTGATAATTCAATGTTGGAGGCGGTCAGTGCGTGCTGCCGTAAATCTCTTTTTCGTCTTTCAGGACGGCATCGGCGGTTTCCCACGCACCGCCGTTCCATTTTTTGTAAAAGCAGCTTTCGCGCCCGGTGTGGCAGGCGATGCCGCCGTTTTGGGCGATGAGCATCACAATGGTATCGCCGTCGCAGTCGAGGCGCAGTTCGCGGACTTTTTGCGTGTGTCCCGACTCTTCGCCCTTCATCCATTGTTTTTGGCGCGAACGGCTGTAATAGTGGGCAAAGCCGGTTTCGACGGTTTTTTGCAGGGCTTCGGCGTTCATCCACGCCACCATTAAAACACGTTTGGTTTCGGCATCTTGGGCGATGGCGCAAACCAAACCTTTTTCGTCAAATTTGACGGCTTCAAGCAGGTTTTTATCCATATTTCCTTTCAGACGGCATAGTCGGGGCGGTCAGAGGCGCACTTCGATGCCGGCTTCGCGCATAGTGCGTTTGGCTTCGCGGATGGCGATTTCCCCGAAATGGAAAATGCCGGCGGCAAGTACGGCATCGGCTTTGCCTTCGGTTATGCCTTCAATCAGGTGCCGGACATTGCCGACCCCGCCGGAGGCGATGACGGGGATGTCGACGGCTTCGGCAACGGCGCGGGTCAGCGGCAGGTTGAAACCCTGTTTCGTCCCGTCCCTGTCCATACCGGTGAGCAGGATTTCGCCCGCGCCGCGTTTTTGCATTTCGACCGCCCATTCCACCGCATCCAAACCGGTCGGATTGCGCCCGCCGTGGGTAAAGATTTCCCAGCGTGTGTTTTCGGGGTTGACGGCTTTGGCATCGACGGCGGCGACGATGGCTTGCGAACCGAAAAATCCGGCGGCTTCGTTAATTAAATCGGGACGGGTAACGGCGGCGGTGTTGATGCTGACTTTGTCCGCGCCTGCATTGAGCAGGCGGCGGATGTCGGCAACGGTGCGTACGCCGCCGCCGACGGTCAGCGGGATGAAGACTTGTCCGGCAACCTCTTCGATGATGTGCAGGATGGTGTCGCGGTTGTCGGATGAGGCGGTGATGTCGAGGAAGGTCAATTCGTCCGCGCCTTCGCCGTTGTAGCGTTTGGCGGCTTCGACGGGGTCGCCCGCGTCGCGCAAACCGATGAAGTTCACGCCTTTGACGACGCGCCCGTCTTTTACGTCGAGACAGGGGATGATGCGTTTTGCCAGTGCCATAATCGGATGCCTTTAGTCGAGGGAATCTGCCAGTTGCTGCGCTTGGGCAAAATCGATGCTGCCCTCGTAAATCGCGCGGCCGGTAATCGCGCCTGCTACGCCATGTTTTTCGGCGGCACACAGGGCGCGGATGTCGTCCAAGCCGGTCAGTCCGCCGGAGGCGATGACGGGAATGCGGACGGATTGGGCGAGTTTGACCGTCGCGTCGATGTTCACGCCGCTCATCATACCGTCGCGCCCGATGTCGGTGTAGATGATGCTGTTGACACCGTCGTCTTCAAAGCGTTTTGCCAAATCAATTACATGATGCCCGGTTACGGTTGCCCAGCCGTCGATGGCGGCCATACCGTCTTTGGCATCCAGTCCGACGATAATCCTGCCGGGGAAGGCTTTGCACGCTTCGCGCACCAAGTCGGGGTTTTTGACTGCCGCCGTGCCGATAATCACGTCGTTCAGTCCCAAATCCAAATATTGTCCGATGGTTTTCAAATCGCGCATGCCGCCGCCGAGCTGCACGGGGATGTCTTTGGCGACGGCGGCGAGGATGTCTTTGATGGCGGGCAGGTTTTGCGGAACGCCGGCAAATGCGCCGTTCAAATCTACCAGGTGCAGGCGGCGCGCGCCTTGTTCGAACCAGTGCAGCGCGGTGTCGGCGGGCGAATCGGAAAAGACGGTCGCCTCTTCCATCAGCCCTTGTTTCAAGCGGACGCAACGTCCTTCTTTCAAATCGATGGCGGGTATCAGCAGCATAGTTTTTCTCCTTGTGCGGGGCCGTGTCGGGCTTACCAGTTTAAAAAGTTTTTCAACATCGTCAGCCCGGCATCGTGGCTTTTTTCGGTGTGAAATTGCGTGGCGAATACGTTGTCTTTGCCGACGATGCAGGCAAACGGGGACGGGTAGTCGCTTTCGCCCAATATGGTTTCGGGATTTTCGGGGGCGAAATAGTAGCTGTGGACGAAATAAAAACGCGTGTTTTGGGGGATGCCTTGAAAAAGCGGGTGGTTTTGGGTTTGGCGCACGGTGTTCCAGCCCATATGCGGGACTTTCAGACGGCATCCCTGCGGGTCGCGGAGGTCGCGGGCAAAGCGTCTGACTTTGCCGCCGAACCAGCCCAAGCCGTCGGTGTTTCCTTCTTCGCTGTGGTCGAATAAAAGTTGCGCACCGACGCAGATTCCGAAAAACGGTTTGTTTTTTAAGGCATCTTTGACTGCCTCGTCCAAGCCGCCGCGCGTCAGTGCCGCCATACAGTCGGGCATCGCGCCCTGCCCGGGAAAAATGACTTTGTCGGCGCGGGACACGCGGTCGGGGTCGCCGCTTAAAAAGATTTTGGTATTTTTTCCGGCAAGCTGCCCCGCCGTCCGGACGGATTTCAATACGGAATGCAGGTTGCCCATACCGTAATCGATAATGGCGGTTTGCATGGCTTCCTCCTCTTTTTTGCAATATGGCTGCGATTTTAACAAACAAATGTGCCGGGCTGATAAAAATGCCGTCTGAAAACGGGAATCTGTCTTCAGACGGCATAGGGTTCAAACCCGGAAAGCCGTTTGTCAGCCTTCCATTTGTTTTGCCTGAACGGCAGTCAGGGCGATGGTGAACACGATGTCTTCCACCAGCGCGCCGCGAGACAGGTCGTTGACCGGTTTGCGCAGGCCTTGCAGCAGCGGGCCGACGCTCAAGACGTTGGCACTGCGTTGGACGGCTTTATAGGTGCAGTTGCCGGTGTTCAGGTCGGGGAAGACCAAAACGGTTGCCTGTCCTGCCACCGGGCTGCCCGGGGCTTTGGATTTGCCTACGCCCGGCACGGTTGCCGCGTCATATTGCAGCGGGCCGTCGATGGCGAGGTCGGGGCGTTTTTCCCGGGCAAGTTTGGTGGCTTCGATGACGGTATCGACATCGGGGCCGCTGCCGGAGTTGACGGTGGAGTAGGAAATCATCGCCACTTTCGGGTCGATGCCGAAGGCTTTTGCGGAATCGGCAGACTGGATGGCGATGTCGGCAAGCTGTTGCGCGGTCGGGTTCGGATTAACCGCGCAGTCGCCGAACACCAGAACCTGATTTGGTAGCAGCATAAAGAATACGCTGGATACGAGGCTTGCGCCCGGTGCGGTTTTAATCAGTTGCAAAGCGGGGCGGATGGTGTTGGCGGTGGTGTGAACCGCACCGGATACCAAACCGTCCACATCATTTTGCGCCATCATCATCGTACCGAGTACCACGGTGTCTTGCAGTTGCTTGCGCGCGTCTTCGGGTGTCAGGCCTTTGGATTTGCGCAGTTCGCACATCGGCTCGACGTATTGTTCGACCAATGAGGCGGGATCGATGATTTCCAAAGAGTCGGGCAGGCTGATGCCGCGTTCTTTGGCAACGGCTTCGACTTCTTCGCGTTTGGCAAGCAGGACGCAGCGGGCAATGCCTTTTTCGTGGCAGATGGCGGCGGCTTGGACGGTGCGGGGTTCTGCGCCTTCAGGCAGGACGATGCGTTTGCCGGCTTGGCGGGCGAAGTCGATCAGGTTGTAGCGGAATTGCGCCGGCGACAGGCGTTTTGCTTCGCGGCCTGCCAATACGGATACGTCTTTCAGCGCGTCGCTCGAACCGAAGAAGGTCAGGCCGGTTTTCTCGGCTGCCGCTTCGGCAACGGAGGCTGCCGCACCGTCCACGACAAAACCTTCCAATACGCCCGGCGCGGCGGCGAAGAACTGTTTGGCAAGGTTCAATTGGTGTGCCAGCGCGTCGGCATCGGTATTGTCGGAACGGACGGCGAAGACGGCTGCCGCGTCAAGGGACAATGCCAGTTCGACGTTTTTGCCTGCGAGATAGATTTTGTCGGCATCGGGCGCGATGCCTTCGATGACGAGGTTGGCGGCATCGAGTGCGGCAACTTTGCCGACCAGTGCGTCGAACCAGTCGTCGCTTTTGCCTTGCGCGAGCAGGGTTTCGGCGGTTGCGTCAACGGCTTGGAAAACTTGTGCGTCCAGTGCTTTTGCAAAGGCTTGTGCGGCGGCGGAGGTGTTCAGTCCGGCAGATACGGGTACGACGAGTACTTTTGCCATAATATATCCTTTCGTATGCTGCGGTGTGCGGCATATGTGGTTGGAAGGGGCGGCATATGGGCAGAAACGGCTGCCTGCGTGCCGTGCGTGCCGTGTTTGGCTTGGGGCGCGCAGGTTGAATATAGCAAACAAATTCTGTTTCCAACAAGATAAATATCCGCAGGCTTGTGGATGCTGCCGCCTTTCAGAGGGTATTTCCGGGGAAGAACAGGGCGGGACCGTCCAAATGGAGGACGGCGGAAATGCCGTCTGACAGGGTGGGGGCGGAAGGGAGGTTGAGCGTGAGGACGGTTTGTCCGGCCCTGAGGCTGATTTCGGTATGCCGCGCTTTGGGCGTGGTTTTGAGAACCACGGCGTGAATGGAGGCGGCGGGTGCGGAATGGGGGTGAAGGCTGAACTGTTCCGGACGGATGAGCAGTGTACCGCGCGTGCCTGCGGGTGCGCCGCTTTGGACGGGCAGGCGGCCCAATCTGCAATCGGCGGTGCCGTCGGCGTTGAGCGCGGCGGGGAACACGATGCCTTCGCCGATAAACAGGGCGGCATCAAGGTCGGCAGGTTGTCGGTACAATTCGTGAGGGCTTGCGGTTTGGAGGATGCGCCCCTGTTTCATCACGGCAATCCGGTCGGCGTATTGCAGGGCTTCTTCGCGGTCGTGGCTGACAAAAACGGCGGATTTTCCGTTGGCGCGCAGGGCGGCAATCATGTCTTCGCGAATCTGGCGGCGCAACTGTTCGTCCAGCGCGCTGAAGGGTTCGTCCAACAAAATCAGTTCGGGGTCGGGGGCGAGGGCGCGGGCGAGGGCGACGCGCTGTTGTTGTCCGCCCGAAAGTTCGTGCGGATAGCGTCCGGCAAGTTCGGAAATGCCGGTCAATTCCAACATGGCTTCGATGCGCTGTCGCTCTTGCGCCGTCCTGCCTTTGCCGTTGCCGAGACCGTAGGCGATATTGCGGTAAACGGTCAGGTGGGGGAACAGCACGCCTTCCTGTACGAGGTAACCCAAACGGCGTTCGCGGACGGGAAGGTTGGTATTTTTCGAGAAGATGGTTTTGCCGGAAAGCGAAATTTCGCCGGAATCGGGTTGTTCGAAACCGGCAAGGCAGCGTAAAAGGGTGGTTTTGCCGCAGCCGGACGCGCCGATGATAAAGAGAATTTCGCCCGGGTCGAGGCTGAGCGAAATGTCGTTTAAAACCGGGGTGTTTTGAAAACTTTTGGACAGGTGTCCGATGTGCAGGGCGGCGGTCATAGCGGTGCTTCCTCAAGCTGTTATTTGAAGGCGTATTTCTTCAGCAGGAATACGGGTATGCCGGAAAATAATACCAGCATCAGCGCGTAAGGGGCGGCGGCGGCGTATTGTGCGTCCGATGTGTATTCCCAAACGGCGGTGGAGAGCGTGTGGACATCGTCGGCGGTCAGCAGCAGGGTGGCGGTCAACTCTTTCATCAGCTTGAGGAAGACGAGTGCGAATGCGGCGGTAATGCCGGGCAGGATGGACGGCAGTACCAACGTCCTGAAAATAAAGAAGTGTCCGCGCCCCAATGTTGCGCCGACCTGTTCCATCCCTTTGGGGAGTTGTTCCAAGGAAGTCCTCAGGGTGGTTTGCGCCATCGGCAGGTAAAGCATGAAATAGGCAAGGATGACGACGATAAAGGTTTGGTAAACGGCAGGGGTGTAGTTGATGCTGAAATAAACCAAGGATAGGGCGATAACCAAACCGGGGACGGCGTGCAGTAAAAACGGCAGCCTGTCTATCCAAACGGTTAAAAAATTGCGATAGCGAACCGATGCCCAAACAAGGGGCAAGGCACATAATATAGTCAAAATCGCACCTAAAGCCGATACGCTTAAGGAACGGATAAAGGCATCAAATACGGATACGAGCGCGAATGTGCCGGAAGTGCCGACCATCATCCAATGTATCAATACGCCAAAGGGGATAATAATGCCCAAAATCAACAAGCTGCTTAAAAAAACAATCGCGCCGATCTGACCGGGCAGTTTGAGGGTTTTGACGGGATAAGGACGGGCAACGCCTTTGCCGCTGTGGTAAATCTTGGCTTTGCCGCGAAATATGCTTTCTCCAAATACGACGATGCCGCACACCGCCGTCAAAACAGCGGAAAGCAGGGCGGCGGTATTGTTGTTGTAGGACATTTCGTATTCTTGGAAAATGGCAGTGGTAAAAGTGGGGTAGTTCAAAATGGATACCGCGCCAAATTCGACCAGCATATGCAGGGCGATCAGTAACACGCTGCTGCCGATGGCGGGTTTGAGCTGTGGGAGGATGGCGGAAAAAAAGGTTTGCAGGCGGCTTTTGCCCAAGGACAGGCTGACTTCTTCGTAAGACAGGCTGATGCGTTTGAGTGCCGCCTCGACGGGCAGGTAGGCGAGCGGAAACGAGGACAGGCTCATAATCATCACTGTCCCCCAAAAGCCTTCGACGCGGAAGGTCAGGCTGATCCAGGTGAAACAGCTGACAAATGCGGGGATGCACAAAGGCAGGGTGATTGCCGTCTGAAAAAAGGTTTTGCCGAAGAAGCGGTAACGTTGGAACAAAAGGGCGCAGGCAATGCCCAAAACAATGGAAATCAGGGTAACGCCCGCCATCATCGTCAAGGTGTTGGAGAGCAAATCCCACATACGCGGGCGGAACAACAGTTCGACGGCGCGGTTGATGCCGACCTGCCACGAACGCATAGCGACATATAAAAAAGGCAGGGTAAGCGGCAGGGCGATCAGTAGGATGAGGCCGGTAAGCCAAATGGGTATTTTTTTAGGAGACATAGTGTTTTTCATCGGCAAAACGGGCGGACAGTATAAATGTCCGCCCGTTTGACAATCCGAAAACGGCTTATTTCATACCGGCTTGCTCAAGCAGCCGGGTGGCGTGTTCTTTTTCGGAAACAGTGGTGGCGGACACTTGGGGTGCTTCCAACTTGGCGATGGGTTCCAAATTGAAGGTGGATACCACGTGCGGATTCAAAGGATATTCGGCACGGACGGCGGTCAGGGCGCGCTGTCCTTCCTTGCCGGCGAGGAAGGCGACGAATTTTTTCGCCTCATCCTTGTTTTGGGAGGATTTTAACACGGCTGCGCCGGAATAGGTAACGAGTGCGCCGGGATCTCTGTGGCGGACGAAATTCAGGCGGGTGTGGACATTTTGTACGCCTTTTTCACGCGCGAAAGCGTGCCAGTAGTAGTTGTTGATGAGGGCGGCATCGATTTCGCCGTTTTCAACCGCTTGAAGGGCGACGGAGTTTTTAGCGTAAGGCTTGCCGTATTCTTTCAGGCCTTTGAGCCATTTCAATGCGGCCGCTTCGCCTTTCAGTTTGACGATGGCGACAATCTGTTCCAAGAACGCGCCGGAAGTGGGGACGTAACCGATGCGGTTTTTCCATTTCGGCGTGGCGTAATTCAGGACGGATTTTTCCAAATCTTTTTCAGACAGTTTGCGGGTGTCGTAAACGACGACGCGCGAACGTCCGCTCAGTGCCACCCAGTCTTTTTTGGCGGCAACCGGCACGCCTTTGCCGCGTGTTTCGTTGATGGTGGAGGCGGGCAGGGGCTCTAGGAGGTTGGCTGCGGAAAGGGTGGCGAGTGCCGGGATTTGTTCGGAATAGAATACGTCGGCGGGGCTTCGGCTGCCTTCTTCTTTGATTTGGCCGGCAAGCTGGTCGCCTTTGGCACTGTTGAGTTTGACTTTGATGCCGGTAGCCCGGGTAAAGGCATCTGCAACGGCTTGTGCCGCTTCTTTGTGTTGGCCGTTGTACACGGTAATGTCTGCCAGCGCGGGGGTGGCGGCGGTCAGGGCTGCGGCAAGCAGTGCGTATCGGATAGATGTTTTCATATCGATTTTCTCCTAAATGAATGAGGGTGTATACCTTGTTAAGACATAACGGGGTGTAGTGTATTCCTTCTTTTTTATAAATGCAAATAATTATTTTTTAAATTTGTTATTATCCGATCCGGTTATTGTTTGTTCTGACTTGTATTTTTTCCGTGCATCGCGCCCGTAAGGCGGAAGCGGCGGGCAATGCGTGGCGGAATGTGGGTAAAGGCGGCATTTTGATTTGTCGGAATGCTGGAGAACCTCTCCCTTTAAAACGCCGTCTGAACAAGGTTGCCGGAATAGTATTGCCATCCCGGCAGATACAGTTTGTCGGGATCTTGCCAATATTGTTCATCCAGACTGTTCGGCAGCGAGGCGGTTTTGTTGTCGAGATGTTTTGTTAATCCACTATGATATCCCACATTTCTTTTAGGTTTTTACCTTCCGATTGGAGGTGGCGGATTTCTTTTCCCAATGCGTCTGCCGCCCGATGGATCAGCATTGCGCTGTGTACCGCGCCGATTTTTTTCAGAACGGAACAAGTCTTTTCGGCGGCGGGAAAACCCCAGTTGCAGACAAATTGCAGTATGCCGCCGTTGCCGATGTCGGCTTCCGCCCGCCAAATCAAAACCAGCTCCTGTTCCCGTCCGTCCATGCTTTCGAGTTTGCCGTCATGCTGTTCAAAAAGTTTGTCCACCGCCTGCCGCATCATCTGCTCGAAGCGGTCGGCTTGGGTATCCGTATCGGTCATAATATTCTTGCCTTTTAAAAATGCCGTCTGAACATTTCTTCAGACGGCATTTGGGGGTTAAGCTGACATTTCCCGCCAGCGTTTGACTTGGAGGCGTACCTGTTCGGGCGCGGTGCCGCCCAAGTGGTTGCGCGCGTTGAGGCTGCCTTCGGGTGTCAGAACGCCGTACACGTCGTCTGAAATCAGCTTGCTGAAGCCTTGCAGGGCTTCGAGCGGCAATTCGCTCAAATCAACGCCGGCTTCATCGGCGTGGCGCACGGCTTGGGCAACGACTTCGTGGGCATCGCGGAAGGGCATACCTTTTTTGACCAGATAATCCGCCAAGTCGGTGGCGGTGGCAAAACCCTGCATCACGGCGGCGCGCATATTGCCGGGTTTGACGGTTACGCCGCGCATCATATCGGCGTAAATCCGCAGCGTGTCGATGAGCGTGTCGGCGGTGTCAAACAGCGGTTCTTTGTCTTCCTGATTGTCTTTGTTGTATGCCAAGGGCTGCGATTTCATCAGGGTAATCAGGCCGATAAGGTGTCCGATGACTCGGCCGGATTTACCGCGCACGAGTTCGGGTACGTCGGGGTTTTTCTTTTGCGGCATGATGGACGAACCCGTACAGAAGCGGTCGGCGATGTCGATAAAGCCGAAACGCGGACTCATCCACAAAATCAATTCTTCAGACAGGCGGCTCAAGTGAACCATAACCAGCGAGGCGGCGGCGGTGAACTCAATGGCAAAATCGCGGTCGGATACGGCATCGAGCGAGTTCTGGCAGATTTGTTCGAAACCCAACAATTCGGCGGTGATTTCGCGCTGAATCGGATAGGTCGTACCGGCAAGGGCGGCAGCGCCGAGCGGCATACGGTTGACGCGTTTGCGACAGTCGGCCATGCGTTCGAAATCGCGTCCGAGCATTTCGACGTAGGCGAGCATGTGGTGTCCGAAGCTGACCGGCTGGGCGACTTGCAGATGGGTAAAACCGGGCATGACGGTTTCGGCGTTTTGTTCCGCCAAATCCAATAATGCCGTCTGAAGGTTTTGAATCAGGTTTTGGATAACGGTAATCTGGTCGCGCAGCCAAAGGCGGATGTCGGTGGCGACTTGGTCGTTGCGGCTGCGGCCGGTGTGCAGGCGTTTGCCCGCGTCGCCGATTTTGTCGGTCAGGCGGCGTTCGATGTTCATGTGGACATCTTCCAAATCGAGCGGCCATTCGATTTTGCCGCTGCGGATTTCTTCGAGGATTTCCGACATGCCCCGACGGATGTCGGTCAGATCGTTTTCACTCAGCACACCCGACCGGGTCAGCATCTGCGCGTGGGCGAGCGAGCCTTGGATGTCCCATTCGGCAAGGCGTTGGTCGAAACCGATGGAGGCGGTGTATTGTTTGACGAGTTCGGAAACGGGTTCGTTGAAACGTCCGGACCAGGTTTTGTCGTGCATAAGGATTCCTTGATGGGGTTATTCGGTGCGGTATTTTTCCAAAAGGCGGCGGAAGGGTTCGCCGGTTTCGGGGTGTTTTAAACCGTAGGCGACGGTGGCTTCGAGATAGCCGAGTTTGCTGCCGCAGTCGTAGCGCGTGCCTTCAAAGGGGTGCGCCAAGACAAATTCGTGATCGAGCAGCTTGGCGATGCCGTCTGTAAGCTGGATTTCGTTGCCCGCGCCGCGCGGCAGGCCGGTTAAAAGGTCGAAAATGCGCGGGGTGAGGATGTAGCGTCCGACAACGGCAAGGTTGGAGGGCGCGTCTTCGGGCTTGGGTTTTTCGACAATGCCGGTAATGCGTTGGAACTGTTTGAGCTGTTCGGTTTCGACGATGCCGTATGAGCCGGTTTGCGACGGTTCGACGGTTTCTACGCCCAAAATGCTGTTACCGCTGCGCCCGTACACTTCGACCATTTGTTTGAGCGCGCCTTTGGGCGCATCGATTAAGTCGTCGGCAAGGATGACGGCGAAGGGTTCGTCGCCGATGGCGGCGCGGGCGCACAAGACGGCGTGTCCCAATCCCAGTGCTTCCGCCTGACGGATGTAGAGGCAGGTAATGTTCGGCGGCAGGATGTTGCGGACGTGTTCCAACAATTTGTCTTTATGGCGCATTTCCAACTTAGTTTCGAGTTCGTATGCCTTGTCGAAATGGTCTTCGATGCTGCGCTTGTTGCGCCCGGTAACAAACACCATTTCCGTGCAGCCGGCTTCCACGGCTTCTTCTACGGCGTATTGGATCAGCGGCTTGTCGACGATGGGCAGCATTTCTTTCGGGTTGGCCTTGGTGGCGGGCAGGAAGCGGGTTCCCATCCCTGCAACGGGGAAGACGGCTTTTTTTATCGGTTTCATTCTTTTTCCTTTGTATTGTTTTGATGTTTAAAGGGCGAGTTTGCGGATTAAATCGGCGAGTGCCTGCGCGCGGTGGCTTTCGCGGTTTTTGACCTCCGAATCCAATTCGGCGGCGGTTTTGCCGTGTTCGGGCAGATAAAAATACGGGTCGTAACCGAAACCGTTTTGCCCGAGCGGCGTGTCGTGCCATTGTCCGTGCCATATGCCCTCGGCGATAATCGGGCGCGGGTCGTCTTTATGACGGACAAAAACCAATACGCAGACATAAGAACAACTTTTGTCGGCCTTGCCGGCAAGTTCGGCGGCAAGTTTCAGGTTGTTGGCGGTATCGGATTTGGGATTGCTGCCGGCGTAACGTGCGGAATGGATGCCCGGCGCGCCGTTTAGGGCGGCGGCACAGATGCCGCTGTCGTCGGCGAGTGCGGGCAGCCCGCTGTGTCCGGCGGCGTGCCGGGCTTTTGCCAGCGCGTTTTCGACAAAGGTGGGATAGGGTTCGGGGCATTCGGGTATGCCGAATGGGGATTGCGGCAATACGGTGATGCCGTAAGGTTTGAATAAGTTGCCGAATTCTTTGAGCTTGCCGGCATTGCCGCTTGCCAAAACGATTTTTTCCGGTTTTTCAGACATAGCGGTTTCCCTGTGATGAAGATGGGGCGGCGCGTAGGGATTTGTGCCGCAGGTAGAGGGCGAGTGCGCCGATTTGCCCGAACAGCGCGCCGAATGCGAAAAGGAAGGACGCGACGGCGAAGGCTTTGCTGCCGGCGGTCAGCAGATAAGCACCCAAGAGGACGGGCAGCATAAATGCCAGTGTGAACAAGGCAACAGACAACAGATAGATTTTTCGGCGGTTCATGGCGTTCGGTCGGAAACGGGATGTTCGGATTATAGCCGATTAGGACGGCATTCCCTAGAGGCTGGAAAAATGCCGTCTGAAGCGGCGTTCAGACGGCATGGCGGGCATTATGCCTGTTTGTTCCAACGTTCGATGGATTCTTTGATGACTTTTTTCGCTTCTTCCGCACCGCCCCAGGATTCGACTTTGGTCGTACCCGCTTTTTTCAGGTCTTTGTAGTGGTTGAAGTGGAACTCGATTTGTTTGATGAGCTGTTGCGGCAAATCGGACAAAGTTTTGTAGGCGTTGCCGTTATTGCGGTCGTCGGCAGGTACGCAGACGATTTTGTCGTCCACTTCGCCGTCGTCGACGAATTTCATCACGCCGATAACGCGCGCTTCCAAGAATACGCCGGTTGCCAGAGGTTGTTCGGTAACGAGCAACACGTCCAATTCATCGCCGTCTTCGTCCAAAGTTTGGGGGATGAAGCCGTAGTTGGTCGGTTTGGCGAAGATGGCGGGTTCGATGCGGTCGAGTTGGAATGCGGCGAGTTTGCGGTTCCACTCGATTTTGTGGTTGCTGCCGGCGGGGATTTCGTTGACGACGTTGATGATGCCGCCGTCCACGTCGCCCGGGGTCAGGATTTGGTTGAAGTCTGCCATTTGGTTTCCTTTATTTGGGAAGGTTTGAAGTTTGAAAGTATAGCACAAACGTCCGGTTGAAAATGCGCCCGATGCCGTCTGAAAGGGTGTACGGGCGCGTGTTACCGTTTGCCCAAAAACCTGCCCAGTTCCAAAATCGCGCGCCTGTTGGACGGGGAAAACACTTTTTCCGCCGCTTCTTCCAAGCCGAACCAGCCGTAGGAGACGTGTTCTTCAGGCTGCAGGACGACGGGCGTATCGCGCGGGATTTCGGCCCAGAAGACGTGTTCGCGGTTTTCAAACACGCCTTTGGGATAGCGATGCCGCCAGTGGTGGTAGATTTCGTAAACCGTGCTGTCGTGCCGGTCTTGAAGCTGCCCGTCCTCCAGCAGGATGCCGGTTTCTTCCCATACTTCGCGCTTTGCCGTTTGGGCGACGGTTTCGCCCGGTTCGAGGCTGCCGGTTACCGACTGCCAAAATCCTTTCGGATGCGTGCGTTCGATGAGCAGGATGCTGCCGTCCCCGCTATAAAGGACGACCAGTGCGGAGACGGGGTATTTGAGCGGTTTTGCCATCGGCATCTTTCGGCGGGCTGCGGTAATGAAGGGGCTGATTATAGCAAACGCCGCACGTTATGGCGTTTGTCCTTTTCCGCATCCTTTCCCGTCCGGGCGGCGCGCGCCGGCGTTTGCCAGTAAATTTTCCGTTGTGTCAAAAAGATAAGGGCGGTTGTGATTTTAATGCTTGCCAAAGCGTCGGGCGGAAACTATAATCCGCGACTTACCGAGTCGGAGTGTGGCGCAGTCTGGTAGCGCACTTGCATGGGGTGCAAGGGGTCGAAGGTTCGAATCCTTTCACTCCGACCAAAAATTCCGAAAGCCGCTTTCAAAAGCGGCTTTTTTGCCGTCCGTATGATTATGATGTAGAGTACGCGGCGACAGACATTCAAATGCCGTCCGAAAACCGTTCAGACGGCATCTCTTTATCTTAATAGTGGATTAACAAAAATCAGGACAAGGCGACGAAGCCGCAGACAGTACAAATAGTACGGCAAGGCGAGACAAAAATCAGGACAAGGCGGAGAGCCGCAGACAGTACAAATAGTACGGCAAGGCGAGGCAACGCCGTACTGGTTTAAATTTAATCCACTATAGTTTCATTCCGTACCATCTTAAGGAACATCAAATTGGGCATTTCCCGCAAAATATCCCTTATTCTGTCCATACTGGCAGTGTGCCTGCCGATGCATGCACACGCCTCAGATTTGGCAAACGATCCCTTTATCCGGCAGGTTCTCGACCGTCAGCATTTCGAACCCGACGGGAAATACCACCTATTCGGCAGCAGGGGGGAGCTTGCCGAGCGCAGCGGCCATATCGGATTGGGAAACATACAAAGCCATCAGTTGGGCAACCTGATGATTCAACAGGCGGCCATTAAAGGAAATATCGGCTACATTGTCCGCTTTTCCGATCACGGGCACGAAGTCCATTCCCCCTTCGACAACCATGCCTCACATTCCGATTCTGACGAAGCCGGTAGTCCCGTTGACGGATTCAGCCTTTACCGCATCCATTGGGACGGATACGAACACCATCCCGCCGACGGCTATGACGGGCCACAGGGCGGCGGCTATCCCGCTCCCAAAGGCGCGAGGGATATATACAGCTACGACATAAAAGGCGTTGCCCAAAATATCCGCCTCAACCTGACCGACAACCGCAGCACCGGACAACGGCTTGCCGACCGTTTCCACAATGCCGGCGCTATGCTGACGCAAGGAGTAGGCGACGGATTCAAACGCGCCACCCGATACAGCCCCGAGCTGGACAGATCGGGCAATGCCGCCGAAGCTTTCAACGGCACTGCAGATATCGTCAAAAACATCATCGGCGCGGCAGGAGAAATTGTCGGCGCAGGCGATGCCGTGCAGGGTATAAGCGAAGGCTCAAACATTGCTGTCATGCACGGCTTGGGTCTGCTTTCCACCGAAAACAAGATGGCGCGCATCAACGATTTGGCAGATATGGCGCAACTCAAAGACTATGCCGCAGCAGCCATCCGCGATTGGGCAGTCCAAAATCCCAATGCCGCACAAGGCATAGAAGCCGTCAGCAATATCTTTACGGCAGTCATCCCCGTCAAAGGGATTGGAGCTGTTCGGGGAAAATACGGCTTGGGCGGCATCACGGCACATCCTGTCAAGCGGTCGCAGATGGGCGCGATCGCATTGCCGAAAGGGAAATCCGCCGTCAGCGACAATTTTGCCGATGCGGCATACGCCAAATACCCGTCCCCTTACCATTCCCGAAATATCCGTTCAAACTTGGAGCAGCGTTACGGCAAAGAAAACATCACCTCCTCAACCGTGCCGCCGTCAAACGGAAAGAATGTCAAACTGGCAGACCAACGCCACCCGAAGACAGGCGTACCGTTTGACGGTAAAGGGTTTCCGAATTTTGAGAAGCACGTGAAATATGATACGAAGCTCGATATTCAAGAATTATCGGGGGCGGTATACCTAAGGCTAAGCCTGTGTTTGATGCGAAACCGAGATGGGAGGTTGATAGGAAGCTTAATAAATTGACAACTCGTGAGCAGGTGGAGAAAAATGTTCAGGAAACGAGAAGAAGGAGTCAGAGTAGTCAGTTTAAAGCCCATGCGCAACGAGAATGGGAAAATAAAACAGGGTTAGATTTTAATCATTTTATAGGTGGTGATATCAATAAGAAAGGCACAGTAACAGGAGGGCATAGTCTAACCCGTGGTGATGTACGGGTGATACAACAAACCTCGGCACCTGATAAACATGGGGTTTATCAAGCGACAGTGGAAATTAAAAAGCCTGATGGAAGTTGGGAGGTGAAAACGAAAAAAGGTGGGAAAGTGATGACCAAGCACACCATGTTCCCAAAAGATTGGGATGAGGCTAGAATTAGGGCTGAAGTTACTTCGGCTTGGGAAAGTAGAATAATGCTTAAGGATAATAAATGGCAGGGTACAAGTAAATCGGGTATTAAAATAGAAGGATTTACCGAACCTAATAGAACAGCATATCCCATTTATGAATAGTAATATTTATGAAAAATTAGGAGATTAATGATGAAAAGAATTAAGTGCTTTTGTGATAAATTTCCATCAGGAGATACATTTAGAATGTGTATCATTCTGGATGACTATGATAATAGGGTTGATTATTATGTAGGAATATATGATTACATTACGTCTACCTTAATGAGCGATATTTACTATCGATCCACGATTGATGAGCATTTCAAGATTATAGAATTAATAGAAAATAATCCAAATGAAATTTATGATGATGGCGGTGGTCAACAATTTTGCCTAGAATTTCATCATGATAAGGTCATTTTTTATCACAATGAATTTGATGAAGAAGATGGTTATCCAGTATTAAGCTGTTCGCTGCATACTTTTAAAACTGCTTTAATTGCTTGGAATGCTTTTTTGCAATTGCCTAAAAGTATTCATTCGGTGGTGGAGACTGTGATTGAGGAATAAGCATAATTAGCTTAATGAATAGAATCAGCGATATAGA

>29 |ref|NC_017511.1| Neisseria gonorrhoeae TCDC-NG08107 | Coordinates: 283434,284049 | Forward

TTGGACTGCAAATCCACGCTTATACGCTGTGCCATGATTAACAGGAAGAGAGCAACTTGGATGGGCTAGTAGGGATGGTAAGCATTTAAATATATCAATTGATGGAAAGATTACACACAAATGACTAATTTAAAATTAGATTTCTACTCTGAAGTTATTATAAAAGATTCTTGCCCTAATGATTTGTTAGAAAATGGTGAGACTATTAAAGGAAAAAAAGGAGTGGTATTAGGTATAAGCGAAGAAGATGGTATAATCTATGGCTATACTATTTTACTTTTTGATATAAAATATTGTATATATATAGATAAGAAATATATTATACCGACTGGAAAAAAATTCTCTCGCGATGATTTTTATTAGCTAATTTAAGTAATTTAAGCAAGTTTTTTAGATACTGAAACTGGTAATTATGTTTATGTAAGCATAAGGAGTTCAGATTGAAGGATTCTACAGGGCAAATGAGGCTGGCAACCAAGGATTTGGCGGAAGCCATTAAACGAGGAGAAGTACGTAGTTCTGCTTTTACAACAAAGCAACTAAAGGCAATCGAAAAAGGCAAAGACAAAATCCCTAGCTACACTTGGCATCATCATCAAGATACAGGGAGAATGCA

>30 |ref|NC_017511.1| Neisseria gonorrhoeae TCDC-NG08107 | Coordinates: 284050,305715 | Forward

GCTTGTGCCTGAATGGGAACATTCTAAAACCGGTCACATAGGAGGGACGGCAATGGGGAAGGGTAAATAATTATGTGGAAAATTTATAAAGAAAATAGCACCGACTTAAATTTTGCCATAGGCAGTATATATTGCCAAGCAATCAATCTTACCGAATTTAAAATGTGGGTCGAAAAAATCATAAGGGAAGTGGATTTAGATGAAATTCCAAATTATTTTTTTGATTTGATAGATTTTCAATCACTATACGATCTGATTGATATTATAGGATTTGTTCCCGAAAATAACTTATCAAAAAATCAGGATAATGCATTAACCGGTATTGCTTTTTTAAGGGGGATAGATGTCTACGATCCTCCCATTTCAAAAGAAAAAGCATTAAAAGCCTTAGAAAAACATCCTGAAATTTATCAGAAGTTTCAGCATTTCTTTCCGTTTGTAGAGCTGCCTCCGCTTTAAACAGTCAAAATGCCGTCTGAAACGATATTCGGCTTTCAGACGGCATTTTTGATATAAAGCGGATAACCAAAGAAATGTTTGACGGCAAAGGAACATCTGAAATACCAAATTACACTTGGCATCATCATCAAGATACAGGAAGGATGCAATTGATTCGTGAAGACTCGCATCATGATACCGACCATATCGGTTGGAGAGCGATGAGTAAAGGAAAGTAACTATGTGGAAAATCATAAAAGAGGATAGTGATGATTTAGGATTTGCAATTAAATGCTTATTCTCTCAATCTATTGATTTAAATGAATTCAAGTTATGGATTGAACAAGTAATACGCGATATGCCCATCGAGGACATCCCTTTTTATATTTTTGACTTGGCGGATTTCAATGGGGGAATTGGAGACATTGACAATATTGTAGGTTTTGTTTCGAGTTACAGCCTATCAAAATCGAAAAAAAATGCCTTGACAGGCATTGCCTTCTTAAGGGGGATAGATGTCTATAGACCCTCCCGTTTCAAAAGAAAAAGCATTAAAAGCCTTAGAGAAATATCCTGAAATTTATCATAAATTTAAACGGTTCTTTCCTTTTGTAGAGCTTCCGCTTCTTTAAAGGACAATATGCCGTCTGAAAAGTTTTCAGACGGCATTTTTATTTCTTCCAGTAGGCGGGGGTGAAGAGGATGAAGACGGTGAAGATTTCCAGCCTGCCCAAGAGCATTGCGGTAACGCAGATCCATTTCTGCATCACGTCCAAACCGGCGTAATTGCCGGCGGGCCCGACTTCGCCCAGTCCGGGGCCGGCGTTGGTAATGCAGGCGATGACGGCGGTAAAGGCGGTGGTAAATTCCATACCGCTCGCCATCAGCAGGAAGCTGAAGAGGACGACGGTCATAAAGTAGATGAAGATGAAGGACATAACGGTCAGCGCGAGGCGGTCGGGTATGGCTTTGCCGCTGATTTTGACGGTGCGGACGGCTTTGGGGTGCAGCAGCACCATCATTTCGCGCAGGCTGAATTTGAACAGGACGAGGGCGCGTATGGTTTTGATGCCGCCGCCGGTCGAGCCGGAGCTGGCGAGGATGTTGGCGAGGAAAAACATCCACAGGGAAATCAGGAGCGGCCATTGTGCGAAGTCGGTGTTGGACAGCCCGTTTGCCAATCCGATGGAGACGAAGTTGAAGGCGGTGTAGCGCAGGGATTCGCCGAAGCCGGCGTAATGGCCGGTGTGCCACAGGTACAGGGCGGCGGCAAGGATGCTGCCGGAGAGCAGCAGCAGCATCGTCCGGCATTCTTCGTCTTTCCAATAGGTTTTGAGGCTGCGGCTGTTGAGGGCGGCGAAATGGCTGGCAAAATTGATGCCGCCGAAAATAGTGAAAACGATGATGACTGCTTCGATGAGGGGGGAGTTGTAATAACCGATGCTGGCATCGTGGGTGGAAAACCCGCCCAGCGAGAGGGTTGCCAGCGCGTGACAGACGGCATCGAACCAACCCATCCCGGCAAAATGCAGGCAGGCTGCCGCGAGGATGGTGATCAGGGTGTAGCCGAACCAGAGTTTTTTCGCCACTTGGGAAATGCGCGGCGACATTTTGCTTTCTTTGTCAATGCCGGGGATTTCGGCTTTGAATAACTGCGTGCCGCCTACGCCGAGCATAGGCAGGATGGCGACGGCAAGGACGATGATGCCCATCCCGCCCAGCCAGTTGAGCATATGCCGCCAAAAGTTGACGGAGGGGGCGAGCCCGTCGACGTGGGGGATGACGGTCGCGCCGGTGGTGGTCAGTCCCGACATCGATTCAAAAAATGCGTCGGTAAAGCCCATATTCGGGAAATACAGGTACATCGGCATCGCAGCCATAGCGGCAAACGCCAGCCACAACATCAGGACGAGGGTGAAGCCGTCGCGCGGGCGCAGTTCGCGCCTGAACCGGAGGGTGGCGAGCCGGACGATGCACGAGCCGGAAAGGGTAACGGTCGCGGTGGTGGCGAATGCGGTGTACGCGCCGTCCGAAAAGGCGTAGGAAAGGGCGGCGGGTATCAGCAGGATAAAGGAAAACAGCATACCCAGTCGGGAGAGGACGTGGGCGATGGGCAGGATTTTGTGCATAGTGGGGCGGTCCGTTATTTTGCGAAGCTTTTCCAGTCTATGCCGCCGGCGGCTTGGACTTGGGTAATTTCTTCCGTTTCGAGGTTGACGGCGGTCAGCTGCCCGCCCCACAGCGCGCCGGTGTCCAGCGAGATGACGTTGTCGGCATTCGTGTAGCCCAGCGAGGACCAGTGTCCGAAGATGATGGTGTGGTCGAGGTTTTGCCGATCGGGGGCTTTGAACCACGGGCGCAGGTAAGGCGGCATTTTTTTCACTGTGGATTTGTAGTCGAAATCCAGTTCGTTTTTAAAGGTCAGGGCGCGCATCCGCGTGAAGGCGTTGACGATGAAGCGCAGGCGGGCATAGCCTTCCAAACCTTCGTCCCATGCGGCCGGTTTGTTGCCGTACATTTTGGAGAAGAATTTGACGTATTTTTTGCCGCGCAGTTCGGCTTCGGCTTCTCCGGCGAGCGATTCGGCTTTGGCTATGCGCCATTGCGGCAGGATGCCGGCGTGTATCATCACGCGGCCGCCCTCGCGTATCAAAAGCGGTTGCGCGCGCAGCCAGTCGAGCATTTTTCCGCCGTCGGGGTGTTTGAGTATGGGTTCGATTGTGTCGCTGCGTTTGAGCGCGCCTTCGCCGCAGCCGACGGCGAGCAGGTACAGGTCGTGGTTGCCGAGGACGATTTGCACGCTGTTTTCGTGCCGGATGCAGAATTGCAGCGTTTCGAGGGATTTCGGGCCGCGGTTGACGATGTCGCCCGTCAGCCAGAGGGTGTCCGTGCCGTGGTTGAAGCCGATTTTGCCGAGCAGCGCGGTCAGTTCGTCGAAACAGCCTTGGATGTCGCCGATTGCGTAATGTGCCATTGCGGGTGTTGTGAAGTGGGAAAGTGTTGCGGTTCGGACGGCACGGTTGGAAATCTTATGCCGTCCGAACGTGGAATTATGCGTTCAAAACGAGGACGGCTTCCGCTTCGACCTGCACGCCTTTGGGCAGCGAGGCAACGCCGACGGCGGCGCGGGCGGGGAACGGCTCGGTGATAAATTCCGCCATCACTTCGTTGAAGACGGCAAAATTGCCCAAGTCGGTCAGGTAGGCGTTGAGTTTGACGATGTCGTCCAGCGAACCGCCTGCCGCTTCGGCGACGGCCTGCAGGTTTTGGAACACTTGGCGCGCTTCGGTGTGGAAATCGCCGTTGCCGACCACAGTCATCGTGGCGGGATCGAGCGGAATCTGACCGCTCATGTAAACGGTGCCGCCGGCGCGCACGGCCTGGCTGTATGCGCCGATGGCGGCGGGGGCTTTGTCGGTATGGATGACGGTTTTGGACATTTCGGATTCCTCAAAAAATAGGGCGGCAGAAGCCGCAGCGTTCGGGATTATCGTACAAAACCGCCGGCTTGTGTAGTTTTAGGCGGTATATGTGCCGGAAACAAAAAACCGCCGAAGGCTCGGCGGTTTGCGGAATAACGCGTATATCAGAATTTGACGCGCACGCCGGCGGACAGTTCGCCGGAACGGACGTTTTTGACATTGTTGACTTTGCCGATGTAGTTGTAGCGGTAGCCGGCATCCAGATCGACATTGGGTGTAACGGCATAGCTTACGCCCGCCAATACGCCGAGGCCGGCGGAGGTTTTGCTGAAGCTGTCGCTGCCGCCCAAGTGGGCGGAAGCGCGGTTGAGGCTCAAGCGCGCGCCGAAATACGGTTTGACGGGCGATTGGGTGTCGAAGTCGTAAATGACGGACGCGCCGATGCTGTAAAGTTTGAAATCGGTGGATGGGGCTTGTTTATAGTTTTTGTAGCGCGTGTAATCGACGGCGAAGCGGAGGTCGTTGATGCGGTAGCCTGCGGAGATGCGCGGGCTGAAGCCTTTGGCAGAACCTAAAGAGCTTGAGGCTTTGGCGTGTGCGGCATCGGCTTGGACGTAAAAGCCGGATGCGCCTTCCGCCAGTGCGGCGGCCGGGAGTGCGAGGGCAATCAGTGCGGCAAGTGCTTTTTTCATATTTTGGTTCCTTTATGGTCAGGGAGAAAAATTGTTAATAATCCGTTAAAGAATCCTGCCGTATTATACTGAAATTTTCTTTTTGCATCGTAATATTTTCAATGCCTCAAGATACGCGGCGGCATCCGGCTGCTTTGCCGACGGCAAAGCCGTTAACCCGCGCGTTGCCCTTAAACGGCGGCATCACGCGGCGGATGGGTGAAACTTGCAAACGGTTTGGAAAAAACCGCAAAGGCGGGGCGGCGGCATTTGTCAGATTGTTGCAGGCGCAGGCGTACGGTTTTTTGTGTGCGGCGTTACCTTAGGCGTCGGACATTTCCGGCGGCGGCTGTGCCGTCTGAAACGCCCGGCGGGGGATGCGGCTGCGTTTCCCATCGATAGGCATATTTGCCGGCCGCGTTCGGGGCGGGTTTTACCCGGGCGGCCGCCGATTTGTTTGCGCTTATAAAAAACACTGCAACAAATCGTTTAAAAACAAGCGTCCTTTTTCGGTCGGGCGGAATACGGTCGGGTCGGTTTCCAGCAGGCCTTTTTGCCTTGCCGTTTCGATTTGCACCATGATTTTGGCGGCGGGTACGCCCGTGCGCTCCTGCAACATCGCGGCGGGTACGCCGTCGGTCAGGCGCAGGGCGTTCATCATGAATTCGAACGGCAAATCTTCGGCGGCGACGGTTTTGCGTTCGACGGCTTCGCCCGGCTGACTTTGCATTAAGGCGAGGTAGTCGTTGGGGTGGCGGCGGCGGACGGTGCGCTCGATGCGGTCGGGATAGGAGATTTTGCCGTGCGCGCCCGCGCCTATGCCCAAATAATCGCCGAACTGCCAGTAGTTCAAATTGTGGCGGCACTGCATGGTGGGTTTCGCAAAAGCCGATGTTTCGTAGTGGATAAAACCCGCGCCTTCCAGTGTGCCGTGTACCGCGTCTTCGATGTCGAGCGCCGCTTCGTCTTGCGGCAAACCTTTCGGCGGCGTATGACCGAACGGCGTGTTCGGCTCCATCGTCAGATGATATGCGCTGATGTGGGTTGCGTCTGTGGCGATGGCGGTTTGCACGTCGTCCAATGCCGTCTGAACGGTTTGGTTCGGCAGGGAGTACATCAAGTCGATATTGACTTTATCAAATAATTTTAAGGCGGTATCGATAGCGGTTAAGGCTTCTTTGCCATTGTGGACGCGCCCCAGCCTTGAGAGCATATCGTCGTTGAAACTCTGTACGCCGATAGAAAGCCGCGTGATGCCTGCGTCTTTAAATCCTTGAAACTTCTCGATTTCAAATGTACCCGGATTGGCTTCCAACGTAATTTCCGCTTCGGGCTGCAAGCGCAACAGCGAACGCACGCCGCTTAACAAACGGTCAATCGATTCCGCCTGAAACAGGCTGGGCGTACCGCCGCCGAAAAATATCGTTTCCACCGGCCTGCCCCAAATATTGGGCAATTCAAGCTGCAAGTCGGTCAGCAGCGCGTCGATATAGGCGGCTTCGGGCAATCCGTTTTTCAGGCTGTGGGAATTGAAGTCGCAATACGGGCATTTTTTGATGCACCACGGGATGTGGATATAGAGCGACAGGGGCGGCAGGGCGGTGAGTCGGGTGCGGTTTGGAAAGGAAATGGTGTGCATGGTGCGGTTCGGAAAAGTGGACGATGCCGTCTGAAGGCGGTTCGGACGGCATGGGTTCAGCAGAGCAGGGTAAGCAGTTCGGCTTCGCTGAGGACGGAAACGCCCAAGGCATTGGCTTTTTCCAGCTTGCTGCCCGCGGCTTCTCCGGCGACGACGTAGTCGGTTTTTTTGGACACGCTGCCGGACACTTTGCCGCCTGCGGCTTCGATTAGGGCTTGGGCTTGGTCGCGTTTGAAGGTGGGCAGGGTGCCGGTTAACACAAAGGTTTTGCCTGCCACGGCTTTATTGATGCCGTCTGAACCTTGCGCCGCCTCGTCTTCAGACGGCATTTGCGCGAAGAAGGTTTTTAGGTTTTCGAGCAGGGCGGTGTTTTGCGCTTTGCTGCGCCACGCCTGCCAGTCGGCAGGGAGGGCTTTGTCGTTTTGCAGTCCTTCTATACTTTGTCCGGCGAGTTCCCATAAGGCTTGGGCTTTGTTTTCGCTGATTTTGAAACCGGGCAGGCGGGTAATCCAGCGTTGCGGCCCGGCATATTGTGCGGCAGGCAGGCTGACGGCTTGGGCTTGCGGGGCAACGCCTGCGGCGAGCAGCTCGTCTATCATCGCCTGCTGTTCGGCTTGGGCGAAGAAGTGGGCAATGGAACGCGCCACTACCGTGCCGATGTCGGGCAGGCAGGCGAGGACGGGTTCGGGGGCGCGGCGGACGCGTTCCAATGTGCCGAATGCCTGCGCCAGCGTTTTGGCGGTGCGTTCGCCGACGTGGCGGATGCCGAGCGCGAACAGGAAGCGGGCAAGTTCGGGCGTTTTGCCGGATTCTATGCCTGCGAGGATGTTTTGCGCCCACTTGGTCGGTTGTTTTTTGCCGTTTTGGGTATTGTATTTAGACAGGTCGCCTGAAACCGTTTCTGCGTCGCCGTTTTCGTTTTCAGATGACCCTTTATCCGCCGTTTCCTTCATTTTTTGCAGGGTCGGAATATCGATGCGGTAGAGGTCGGCGAAATGGCGGACGAGGTCTTGGGCGACCAGCTGTTCGATTTGTTTTTCACCCAAGCCGTCGATGTCCATCGCTTTGCGCGAGGCGAAGTGGATTAAGCCTTGCGCGCGTTGTGCCTGACAAAGCATACCGCCGCTGCATCGGGCGACGGCTTCGCCTTCTTCGCGTTCGATTTCGCTGCGGCAGATGGGGCAGCGGGCCGGCAGGCGGTAGGGCTTATGGAGCGGAACGGATTCGGTTTGCTTGGCGGACGGTGTTTCGGCAAACAGGTCGTCCTGCTGATGCCCGATGCCGTCTGAAACGGCAACGGCGGTTTCCTGCATCGGGCGGCGTTCGAAAATCACGCGCACGACTTCGGGAATCACGTCTCCGGCGCGGCGCACGACGACGGTATCGCCGACGCGCACGTCTTTGCGCGATACTTCGTCCTGATTGTGCAGGGTGGCGTTGGTAACGGTTACGCCGCCGACGAATACGGGTTGCAGGCGGGCAACCGGCGTTACCGCGCCCGTCCTGCCGATTTGCACGTCAATCGCTTCGACAATGGTCAGGGCTTCTTCGGCAGGGAATTTGTGGGCAACCGCCCAACGCGGCGCGCGGGAGATGAAGCCGAGTTCGCGCTGTTGCGCCAAGCTGTTGACTTTGACCACCATGCCGTCGATTTCGTAGGGCAGTTCGGGGCGTTTTTGCTGCATGTGTTCGTAAAACGCCAATACTTCGCCGATATTTTTGAAACAGCCGAAATTGCCATTGGGCAGACTGAAGCCGAGTTCTTGGAAATAGGCGAGTTCTTGGATGTGTTCTTCAGCTTCGAAACCGCCTTGCTGACGGGCAATCGAATAGGGGAAAAAGTGCAGTTTGCGTTGCGCGGTGATGCGCGAATCGAGTTGGCGCAGGCTGCCGGCGGCGGCGTTGCGCGGATTGGCAAAGGGTTTTTGCCCGTTTTCGGCTTGGCGTTTATTGAGCCCGGCAAAGTCGGCTTTGAGCATCAGCACTTCGCCGCGTACCTCGATGAGTTCGGGCACGTTTTCGCCGTGCAGCCGCAGGGGGATGTTGGATACGGTTTTGACGTTTCGGGTAACGTCTTCGCCCGTCGTGCCGTCGCCGCGCGTTGCCGCCTGCACCAATACGCCGTCGCGGTAGAGCAGGCTGATGGCGAGGCCGTCGAATTTGGGTTCGATAACGTATTCGGGATTGCCGCCGTCCAAGCCGTCGCGCACGCGTTGGTCGAAGGCGTACATTTCGGCATGGTCGAACACGCCGTTTTCATCTTGCGGGGAAAAGGCGTTGGTCAGCGACAGCATCGGCACTTCGTGGCGCACTTCGGCAAAGCCTGCCAAAGGCTCGCCGCCGACGCGCTGGGTCGGGCTGTCGGGCAGCTTGAGCTCGGGATGGTTTCGTTCCAACGCTTCGAGTTCGCGGAACAGGCGGTCGTATTCGGCATCGGGTATGCTGGGCGCGTCGAGGGTGTAGTATTCGTAGGCGTAGCGGTTGAGGAGGTCGGTGAGTTCGTGTATGCGTTGTGCGGTCGGGTTCATGGATTCGTACAGGTTCAGACGGCATGGACAAATGCCGTCTGAACGGTAAAACGTGTTGGAAATGGTCGGATTTTACCTGAAAACAAAAGGCGGATGCACCGTTGCCGATGTATCCGCCTTTGTGTTTGCGGAGTTATGAAAACAGGCGCAGGGCGGTTTTGCCGCCCGGTTCGATACCGACTTTGAGCATCTCGGACTGACGCGCCAATACATAAGTGCGTACGTCTTTGAGCCATTGGGTCGAAACTTCTTCCATTTTGTCGTTGACCAGATTCAGGTTCAACTGACCGGACAGGCGTACCGCCAAATCCATAAACAAATCGTCGAAGGTTTTTTCGCCTGCCGGAGAGTGCGGGATGTCGAGCAGCATACTGAAGCCTTTGTAGGACTGGTTGTCCAAAAGGGCATTGGTAAACGGCTCGTTGTTGAGCGAGCAGATGGAGAACATGGTCGAGCCCGACGTGTCGGTATAGTGGAACGCGCCGTCGTCTTCCAAAACGAAACCCACGCCCGTTACGGCGGAACGCAGTTCTACGCCGCTGATGCTGGTCGGCGAAACCAAATGGATGGCGATGGTCTGGTCGACGCGCGCGCAGAATGCGTCCAGTGCGGAAGCCACTTCGATAAAGGCGGCAAGGTCGGTGTGCAGCGTCTGACCGCCCATGCTTTGTGCGAATGCGTCCGCCTGGCGGTTGAATGCGGAGAGTTCTTCCTGCGAGGCAAGTCCGTTGCGGCTGACTGCCTGGATACCCACGATAAATGCCTGATAGCGGATGCCCGGGATGGGTTCGGCAATCTGGAAATGGTCGTCCATGGTGCAGCCGACAATCTGGTAGCGGCAGCGGTTGGAAAGGCGCGGCAGTGCGTGCAGTTCTTTGGCTTCGGTCAGCGCGATATAGGAGATGAAGTCGAAGCGCACGTCAAACCAGGGCAGCTCGACCTTCGACAGCTCTTTGAGCGTAATCAGCGGTTTTGCCGGCGTTTGCGGAACGGGAACGGGTTTTGCCGGCGCGTCGGCAGGTTTCGGTGCGGAATGTCCGGTTTGGGGTTCGGAAACGGTGTGGGCGGAGTTGCCGATAATGCCGATTTCTTCCAAGGCGGTTTCGATTTCGGTTTTGAACGGGGAGGCTTTTGCCTGTTTCTGCTTGGCGATGTAGACGGCATCCTGTTCTTGCAGGTTGCGCATGGCGGAGTCTTGGGGTTTGGCCGGTTTTTTGACCGCCGGTTGGGGTTTCGGCATCATGACTGGCCCGCCGGACGGTTTGCCGTCGCGGACATGGCTGGTTTTGCTGTTGAGCAGGGCATCTTTGTCGGAGTGTCCGAACTGGTCGCGCACTTTTTTGCGGTATTGGTTTTCCTGATACATATTGTAGGCGACAACGGCGAGGACGGCGGCGAGGAACAGTACGATGTAAATCATGGCAATCACTTTAAATTTCGGGATGCAGGATGCGCAAAGTGCGGGTACTGCGGTTAAATCGGGCTTGCGTTTCCGGCAGTCTGACGGAACGGCCGGTTATAACGTTTGAATTATAACGAAAATTGCAGGGTCTGACAGCAGTGTGTCGAAATAAGCGGAAATTTTCCGAAATGCCGTCTGAAATCTGTGGTTTTCAGACGGCATTTCTGTCCACGGGAAACCCTTTTTCCCGTATCCGCCGCCAGTCGAAAAAATGGCCGGGGTCGGTTTTGCGGCCGGGTGCGATGTCTTGGTGTCCCGTTACCGCCGTGATGGGGTAGCGGCGGCAGAGGGCTGCCAACAGTGTTTCGAGCGAACGGTATTGCGCTTCGGCAAACGGTTCGAAATCGCAGCCTTCCAGTTCGATGCCGATGGAAAACGCGTTGCACTTCTCCCGCCCCCGAAACGAGGATGCGCCGGCGTGGTACGCCATGTCGCCGCATGATACGAACTGCACCGTTTCGCCGTCGCGTGCAATCAGGAAATGGCTGGACACGTGCAAAGTGTGTATCAGGCTGAAGAACGGATGTCCGTCGGGGTCGAGCCGGTTGGCAAACAGCTTTTCCACCGCATCCGTGCCGTATTCGAACGGCGGCAGCGAAATGTTGTGCAGCACGATCAGGGAAACCGTTTCTCCCGTTTCCCTCGGGCTGAAATTGGGCGACGGGGTATGGCGTATGCTTTGAAGCCAGCCGTTTTGCCAGTGTGCTCCGGCGTGATTGTCCATGATGTTCTTCCTGTCCGGCGGGCAATTTGGGTTATACTGTCGCCCGAATTTTAAGACGTATTCCGAATGCTGGGAATCCTACCATGTTGAAAAAAATGTTGAAATGGACTGCCGTTTTTTTGACCGTATCGGCAGCCGTTTTCGCCGCGCTGCTTTTTGTCCCTAAAGACAACGGCAGGGCATACCGAATCAAGATTGCCAAAAATCAGGGTATTTCGTCGGTCGGCAGGAAACTTGCCGAAGACCGCATCGTGTTCAGCAGGCATGTTTTGACGGCGGCGGCCTACGTTTTGGGTGTGCACAACAGGCTGCATACGGGGACGTACAGATTGCCTTCGGAAGTGTCTGCTTGGGATATCTTGCAGAAAATGCGCGGCGGCAGGCCGGATTCCGTTACCGTGCAGATTATCGAAGGTTCGCGTTTTTCGCATATGAGGAAAGTCATCGACGCAACGCCCGACATCGGACACGACACCAAAGGCTGGAGCAATGAAAAACTGATGGCGGAAGTTGCGCCCGATGCCTTCAGCGGCAATCCTGAAGGGCAGTTTTTTCCCGACAGCTACGAAATCGATGCGGGCGGCAGCGATTTGCAGATTTACCAAACCGCCTACAAGGCGATGCAACGCCGCCTGAACGAGGCATGGGCAGGCAGGCAGGACGGGCTGCCTTATAAAAACCCTTATGAAATGCTGATTATGGCGAGCCTGATCGAAAAGGAAACGGGGCATGAGGCCGACCGCGACCATGTCGCTTCCGTCTTCGTCAACCGCCTGAAAATCGGTATGCGCCTGCAAACCGACCCGTCCGTGATTTACGGCATGGGTGCGGCATACAAGGGCAAAATCCGTAAAGCCGACCTGCGCCGCGACACGCCGTACAACACCTATACGCGCGGCGGCCTGCCGCCAACCCCGATTGCGCTGCCCGGCAAGGCGGCACTCGATGCCGCCGCCCACCCGTCCGGCGAAAAATACCTGTATTTCGTGTCCAAAATGGACGGCACGGGCTTGAGCCAGTTCAGCCATGATTTGACCGAACACAACGCCGCCGTCCGCAAATATATTTTGAAAAAATAAACCATGCCGTCTGAAAAGTTTGTGTTTTCAGACGGCATACCCTTACCGGAACTGCAAGCATGAAACCGCAATTCATCACTTTGGACGGCATAGACGGTGTCGGAAAATCCACCAACCTTGCCGTCATCAAGGCATGGTTTGAACGGAGGGGGCTGCCCGTGCTGTTCACGCGCGAGCCGGGCGGAACGCCGGTCGGTGAGGCCTTGCGCGAAATCCTGCTCAACCCTGAAACCAAAGCCGGTTTGCGTGCGGAAACACTGATGATGTTCGCCGCGCGTATGCAGCACATCGAGGAAGTCATCCTGCCCGCGCTTTCAGACGGCATCCATGTCGTGTCCGACCGTTTTACCGATGCGACCTTCGCCTATCAGGGCGGCGGGCGGGGGATGCCGTCTGAAGACATTGAAATTTTGGAACATTGGGTGCAGGGCGGTTTGCGCCCCGATTTGACCCTGCTGTTGGATGTGCCGCTGGAAGTATCGATGGCGCGTATCGGACAGGCGCGCGAGAAAGACCGGTTCGAGCAGGAGCAGGCGGATTTCTTTATGCGTGTGCGCGGCGTTTATCTCGACCGAGCCGCCGCCTGTCCCGAACGGTACGCCGTTATCGACAGTAACCGCAGCTTGGATGAAGTCAGAAACAGCATAGAAAAAGTGTTGGACGGACATTTCGGCTGTTGATGCGGCAAATATTGAAACAAGCGCATCCGCCCGCGCCGAAAACCAAACGGCAGTGCCGCAGGTGAAAATGGCGGTATGCGCCAAACTTTCGGCATGATAGAATTACGCTCGGTTACAAGGCAGGATGCGTCGGCAATATTAACGAACCGCCCGTAACATGATGACCCGAAAGCGTTTCGGACAGTTCGATTCAAATCTTTTTCTCGCAACAGGATTGACACATGGAAAACTCATTGAAAGAAGCCGCCCTCAAGTTCCACGAATTACCCGTTCCGGGCAAAATTTCCGTTACCCCGACCAAATCTCTGGCGACCGACAAAGATTTGGCGTTGGCGTACTCTCCGGGCGTAGCCGCCCCCTGTATGGAAATCCATGCCGATCCGCAAAATGCCTACAAATACACCGCCAAAGGCAACTTGGTCGCCGTCATTTCCAACGGTACGGCCGTTTTGGGCTTGGGCGACATCGGCGCGCTGGCGGGCAAACCCGTGATGGAAGGCAAAGGCGTATTGTTCAAAAAATTCGCCGGTGTGGACGTGTTCGACATCGAAATCGACGAAAAAGACCCGCAAAAACTGGTGGACATCATCGCCGCTTTGGAGCCGACCTTCGGCGGCATCAACCTCGAAGACATCAAAGCACCCGAGTGTTTCTACATCGAACGCGAATTACGCAAACGCTGCAAAATCCCCGTATTTCATGATGACCAGCACGGCACGGCCATCATTACCGCCGCCGCCGTATTGAACGCCCTGCGTTATACCGGCCGTAAAATCGAAGAAGCGACTTTGGTGTGCTCCGGCGCAGGTGCCGCCGCGATTGCCTGCCTGAACCAACTGCTGGATTTGGGCTTGAAACGTGAAAACGTAACCGTTTGCGACTCCAAAGGCGTGATTTACCAAACCCGCGAAGACAAAGACCGCATGGACGAATCCAAAAAATTCTACGCCGTTGAAGACAACGGCCGGCGCGTACTTGCCGATGCGGTTAAAGGCAAAGACATCTTCTTGGGCCTCTCCGGCGCAAACCTGCTGACGCCTGAAATGTTGAACACGATGAACGAAAAACCCATCGTGTTCGCCATGGCCAACCCGAATCCGGAAATCCTGCCGCCGCTGGCGAAAGAAACCCGTCCGGACGTGGTTATCGGTACCGGCCGCTCCGACTTCCCGAACCAAGTGAACAACGTATTGTGCTTCCCGTTCATCTTCCGGGGCGCGTTGGATGTCGGCGCAACCACCATCAACGAAGAAATGAAACGCGCCTGCGTGTATGCTTTGGCAGATTTGGCGATGGAAGAAGTAACCGAAGAAGTGGTTGCCGCTTACGGTAAGAAATTCGAATTCGGCGCGGAATACCTGATTCCTACTCCGTTCGATTCCCGCCTGCTGCCTCGCGTCGCTACGGCTGCCGCCAAAGCAGCGATGGAAAGCGGCGTGGCAACCCGTCCGATTGCAGATTTGGAAGCTTACGCTGCCAAGTTGGGCGAATGGAAGCTGTAAGCCGTTTGTGGTTAAAATGCCGTCTGAACTGTTTTCAGGCGGCATTTTGTTGTCAGATTGATAAATGAAAGATACTGGAAAATGAAAGAGATGAAACCTGTCCGTTATCATATTGGCGATATACCCGAAACTTCAAAACAAACCGCCCCCGGTCATGACGACAGGGCAGTGGGTGTTGACGATGATTGTTTTCATGATTCCTTTGGTCAATATCGTTATGTTTTTTGTTTGGGCGTTCGGCAGAGGCAACCCCAACCGTGCCAATTTCTGTAAAGCACTGTTCTTATTTACCTTGTTGGTTCGCTTATCGGTTTGATTTTCATATTGCTTATAGGTGGGTCTGCATCGGGTACGCATTATTAATGTGCCGGCTGATTCTGCTTCGAAGATTTGTATCGAATATGCCAAATTTTTTTAAATTTCATACCGTTATCGAACGGCATTGGCAAAAACCTTATCCGGTTTTGTCTTTTCTGCTCAAGCCGCTTTCCGGGCTGTTTGCCAAAATTGCGGCAAAATGGCGGGCGGATTTTTTATCGGGAAAACGGCAAAGCGAAAAGCTGTCCGTGCCTGTGGTCGTGGTCGGCAACATCCACGCGGGCGGGACGGGCAAAACGCCGATTGCCGCCGCGCTGGTGTCGGGCTTGCAGGAAAAAGGTGTCAAGGTCGGCATCATCAGCCGAGGCTACGGGCGCAAGAGCAAGGCGGTTTATGTATTGAATGCCGCGAGCCGTGCGGAAGATGCGGGCGACGAGCCTTTGCTGCTGTTCCGTAAAACAGGTGCGCCGACGGCAGTGGGCAGCAGCCGTGTGGAGGCAGGCAGGGCGTTGCTGGCAGCGCATCCGGAGCTTGAATTGATTGTGGCGGACGACGGTTTGCAGCATTACGCCCTGCAACGCGATGTGGAAATCGCGGTGTTTCCGGCGGCGGATACGGGGCGTACGGATTTGGACTTGCTGCCCAACGGCAATTTGCGCGAACCTTTGTCGAGATTGGAAAGCGTGGATGCGGTGGTGGTCGGCGGCAGGGCGGCGGATGGTTTTATGCCGTCTGAACATTTGTTCGGCAGCCGTATTGAGGCGGGTGCGGTTTACCGTTTGAACCGTCCGTCTGAAAAACTGGATATTTCGACATTGTCAGGGAAGCGCGTCGCAGCGGTTGCCGGTATCGCCAGGCCGCAGCGTTTCTTCGATACTCTGACACACATGGGCATCCGCCTTGACCAAACGGTTGCTCTGCCCGACCATGCCGATATTTTCAATCGGGATTTGCCGCCTGCCGATGTGGTGCTGGTAACTGAAAAAGATGCGGTCAAATTTTCAGACGGCATTTGCACCGATAATGTTTGGGTGCTGCCGGTTTGTGCGATAATCGAACCTGATTTGGCGGAGTTTGTGCTGGAGCGGTTGGAGGGTGTACCGAAGGCCGTCTGAAAGCGCGGTTTGGACGAAGTGATAACGGACTGGAATAAGAACGCCCTACGCCGTCATTCCCGCGCAGGCGGGAATCTAAGTCTCGAATTTTCAGGAATGCCTAGGAGACTTCAGAAATCCCAAATCTCCGGATTTCCACTTGGGCAGGAATGAGAAAGCCGGTTGTATTTTTTATCTGCATTAATCATTCGTTAAAGGAGTGGATATGAAGCTGAAAACCTTGTTATTGCCCTTCGCCGCACTGGCATTGTGTGCCAACGCATTTGCCGCCCCGCCCGGCGACGCGTCGTTGGCACGTTGGCTGGATACGCAGAATTTCGACCGGGATATAGAAAAAAATATGATTGAAGGCTTTAATGCCGGATTTAAACCGTATGCGGACAAAGCCCTTGCCGAAATGCCGGAAGCGAAAAAAGATCAGGCGGCAGAAGCCTTTAATCGTTATCGTGAGAATGTTTTGAAAGATTTGATTACGCCCGAAGTGAAACAGGCTGTCCGCAATACCTTATTGAAGAATGCCCGTGAAATATACACGCAAGAAGAAATTGACGGCATGATTGCCTTTTACGGTTCGCCTGTCGGTCAGTCCGTGGTTGCCAAAAATCCGCGCTTAATCAAGAAATCGATGAGTGAAATAGCGGTATCTTGGACTGCATTGTCAGGGAAAATCGCGCGACATCATCTGCCCGAGTTTACGGAAGAGTTACGGCGCATCATCTGCGGCGGTATAGTGGATTAAATTTAAATCAGGACAAGGCGGCGAAGCCGCAGACCGTACAAATAGTACGGCAAGGCGAGGCAACGCCGTACCGGTTTAAATTTAATCCACTATAAAAATCCCGATGCGGGCTGCAAGCAGGCCGGACAGCTTGGGAAAAGGTATCAGAAGTAAATAATAGCCGCCTGAAATATTGCGGAGGGCATCCGATTGATTAAACCATCAAACCCGAAAGCAACCTTATGGAAAAAAAATTCTTAGACATCCTCGTCTGCCCCGTTACCAAAGGCAGGCTGGAATATCATCAGGACAAACAGGAATTGTGGAGCCGTCAGGCGAAGCTTGCCTATCCGATTAAAGACGGCATTCCCTATATGCTGGAAAACGAAGCACGAGCGTTGAGCGAAGAGGAACTCAAAGCATGACCGAATTCGTCGTATTGATTCCGGCGCGGCTGGATTCGTCGCGCCTGCCCGGAAAAGCCTTGGCGGACATTCACGGCAAACCGATGGTCGTGCGCGTTGCCGAACAGGCGGCAAAAAGCAAAGCCGCGCGCGTCGTCGTCGCCACCGATCATCCCGACATTCAGACGGCCTGTCAGGCGCACGGTATCGAAGTCGTCATGACTTCAAACCGGCACGAAAGCGGCACGACGCGCCTTGCCGAAGCCGCCGCCGCGCTGAAGCTGCCGCCGCATTTGATTGTTGTGAACGTACAGGGCGACGAGCCGCTGATTGCCCCCGAACTCATCGACCGCACCGCCGAAGTACTCGTCGAAAACAACGTCCAAATGGCGACCGCCGGCCACGAATTGCACGATTTCGACGAATTGATGAATCCCAATGCCGTCAAAGTCGTCCTCGACAAAAACGGCAACGCCATCTACTTCAGCCGTGCCCCGATTCCCTATCCGCGCGATGCGATGCGTGCCGGAAAACGCGAAATGCCGTCTGAAACCGCCGTCCTACGCCATATCGGCATTTACGCCTACCGCGTCGGCTTCCTGCAACGCTATGCCGAAATGAGCGTCTCGCCGCTGGAAACCATCGAATCGCTGGAACAACTGCGCGTCCTGTGGCACGGCTACCCCATCGCCGTCGAAACCGCCAAAGAAGCCCCCGCCGCCGGTGTGGATACGCAGGAAGATTTGGACAGGGTTCGCGCTGTATTTCAGACCGTATAAAACAGGTTCAAAGGGAAAAGATATGCAGCAACATATTGAAAAGTGGCAACACTTGAGCCGTGAAGAACAGAAAATCCTTGCCGAAGTATGGGGTCTCGTGCAAAACGATGATCAGGAGGTTCACTATGAAATGCTCAAATTGAACGCACCTGATGAAGTCAGCGGTGAATTTTGGTTCAGAATGGCAGAAACACTCAGCACCCTGCCGCCCAATCGTTCCCTCGACCTTAGAATGAACGGCGGCAGGCTGTCGACCGCCGTATCCATCCTTTCCGTCATGATTGAAGACAATCCCGACATACCGCAGCTTTGGGCGTAAAAAATTACCGCCCTCAATTATTTGGTACACGGACACAAAGCCCGTGCCGACGGTTTGGTACAACAGCCCGACAAAGCGGCAGAAGCCAACGAGGAGGAATACCTGACCAAAGCCCTGTCGCAAAACCTGCTGTCAACATTGGATGCCGCACTTGCACGTTTTCCTGAAGACGCGTGGTTTCAGGAAATCAAACAGGATGCACAAAAGCATTTTGCTTGAGGACGTGGCGGTCAGGAATATTTCCATTCAGGAAGAAAAGAAGTGCCTAATTGGGTATAATCAGGGTAAATCTTATTTTATTTCAGAAGATTAATATTTGTTTTCCGTTTTTCCTTGACGGTATCGGAAAAGTTGATTATAGTTACAGCTTCCTTAGGAGTAATGGCTGAGAGGCTGAAGGCACTTCCCTGCTAAGGAAGCATGTGGGATCAACCTGCATCGAGGGTTCGAATCCCTCTTACTCCGCCAGATAAAAAAATAGACGCTGTGTTTTACAGCGTCTATTTTTTTATGCAATTTTATAGCGGGTTGGCGCAAACCCGGTATGGTATTGCCCTGTCTTGATTCTGAATTTTGTTAATCCGAGATGTTTGCCGTTTATTTTTGCCTCGTTCAAACGGCGGCTCTGATCTGCGCGGTTTCTGTTTGCCGTATTCGCCTACCCGTACCGCAAATGTTATACTGGGAAAAATTCACCGATTGTATTTTACGGCGTATTTGCCGATAGGATGGAAGAGACAAATGAGCAGAATCCGGCAGGCTTTTGCCGCTTTGGATGGCGGAAAGGCATTGATTCCCTATATTGCGGTGGGCGATCCCGATATTCGGACAACTTTGGCATTGATGCACGGCATGGTTGCAAGCGGTGCGGATATTTTGGAGTTGGGCGTGCCGTTTTCCGATCCGATGGCGGACGGGCCGGTTATTCAGCGTGCGGCGGAGCGGGCGTTGGCAAACGGGATTTCGCTGCGCGATGTCTTGGATGTCGTCAGAAAATTCCGTGAAACCGACACGCAAACGCCGGTTGTTTTGATGGGATATTTGAATCCCATACATAAGATGGGTTATCGGGAGTTTGCTCAGGAAGCCGCAAAGGCGGGGGTGGACGGCGTGTTGACGGTGGATTCCCCCATCGAAACCATCGATTCTTTATATCGCGAGCTGAAGGATAACGAGGTCGACTGTATTTTCCTGATTGCGCCGACGACGACGGAAGACCGTATTAAAACCATTGCCGAGCTGGCAGGCGGATTTGTCTATTATGTTTCGCTCAAGGGCGTAACGGGCGCGGCAAGTTTGGATACGGATGAGGTTTCGCGTAAAATAGAGTATTTGCGTCAGTATATCGATATTCCCATCGGTGTCGGTTTCGGCATCAGCAATGCGGAAAGCGCGCGCAAAATCGGCCGGGTTGCCGCCGCGATCATTGTCGGCAGCCGGATTGTGAAAGAGATTGAAAACAATGCGGGCAACGAGGCTGCCGCCGTCGGTGCTTTGGTCAAAGAGTTAAAGGATGCCGTGCGCTGATTCTGTCGCGCATTCTAAATGTTTTAGGAGTTGTCCATGAGCTGGTTAGATAAAATCCTGCCACCCAAAATCAAGAATCGTGGGAAAGACGGTTCTTCCAATGTTCCCGAGGGTTTGTGGCGCAAATGCCCGTCTTGTTCGGCAACCGTTTATTCGACCGAGTTGCAGCAGAACAATCAGGTCTGCCCGAAATGCAACCACCACAATCCGTTATCGGCGCGCCAGCGTTTGAACCTGCTTTTGGATGAGGAAGGCAGGGAGGAAGTTGCGGGTAATGTCAAACCGACCGATCCTTTGAAGTTTAAAGACAGCAAAAAATATCCGGACCGTTTGAGTGCGGCACGCAAGCTGACCGGGGAAGATGACGCGCTGGTGGTGATGAAGGGGATGATGAACGGTCTGCCCGTCGTCGTTGCCGCGTTTGAATTCCGCTTTATCGGCGGTTCGATGGGTTCGGTTGTGGGCGAGCGTTTCGTACAGGGTGTCCGCCGTGCGGTTGCCGATAATTGTCCGTTTGTCTGTGTGGCGGCTTCCGGCGGCGCGCGTATGCAGGAGGGTGTAAACTCGCTGATGCAGATGACGAAAACCAGTGCCGCGCTGCATTTGCTGACGGAAAAACGCCTGCCGTTTATATCGGTGTTGACCGATCCGACTATGGGCGGCGTATCTGCCAGCTTCGCATTTTTAGGCGATGTCGTGCTTGCCGAACCGAACGCGCTGATCGGTTTTGCCGGTCCGCGCGTGATTGAGCAGACGGTGCGCGAAACGCTGCCGGAAGGCTTCCAACGCGCCGAGTTTCTGCTGGAAAAAGGCGCGATCGACCAGATTGTCGACCGCCGCGATATGAAGCGGCGCATCAGTGATTTGATTACGCTGTTGTGCCGTCAGGACAAAGTTTCCGCCACCTGATGGCTGATGAATCGAATGCCGTCTGAAACCGATGTTTCAGACGGCATTTTTGTGTCTGGTTATTTGTCGTGCGGCTTCATCGACGGGGCATAGCGTCCGGCACGTTCTTTCAGGCGTTGTACCAAACCTTGCGTGTCGGCGGGTACGCCGCCCTCGCAGAATGCCTGATACAGGACGGCGCGCAATGCGTCGTTGCGCCCAAGTGTGCCGCCTATCGGTTTCCATTCGGCGTTTCGGGGCTGTATCCAGCGGCTGTTGACCGTATCGCCGTATCCGAATACTTTGTAGGAGGAAAGTTTGCCGTTGCCGAACCCGATGGAGGAGAGGGCGCAGAATATGCCTTCGGCAGTCAGGTTGTCGTAGCCTTTGTCGGAGCGGATATTGAGAATGTAGCGGATGCTGCCGTCGGGCGCGGGCATAATTTGCAGGCTGTCGAGCAGGATTTTCGGCTGTTTGCCGTAATTTTCATCCACATAAATGTCAAACCAGCCGTCCGAGTGCGCATCGGGCAGAGGCGGCAGTTCGGCGGTATGTTCTTTAAATTCGCGGGCGGCGGCTTCTTCGGGCGTTTCGCGGTAGCGGGTGTTGATCGGCGTGTCTTTTTGGCTGAAGCCGGCGGCAAGGGACGTGCCGACAGTCAGGGTCAGAATCAGAAGGATGGCGCGGCGCATAAGTTTCTCCAAATTGAAAACGGCTTTATTTTATGGTTTGGCGGGAAGGGCTGCAAGCAATCGGGGTATAATCTGACCTGATTTTCATTTTAAAGCGGTGTCGAACCATGAACAGCGAAACTTTAGACGTAACCGGATTGAAATGTCCCCTGCCGATTTTGCGGGCAAAAAAGGCTTTGGCGCAAATGCGGCAGGGCGAGGTATTGACCGTTCTGGCGACCGACGGCGGCGCGCCGGGGGACTTTGAGGCTTTTTGCCGCCAAACCGGTCATGTGCTGTTGGATTCTTCCGAACAGGACGGCGTGTTCACACTGGTCGTCAAGCACAAATAAATGCCGTCTGAAATGCGGATGTCCTGATTGATGTTATTGCTGTCGGCATTTGTGCTTGCTGCGTGTAATGCCCAAAACCGGCAATCCGGTGTACGGCATCAGGATTTTGCACGCGTAGCCGTCGATTTGTTTCGGAGTGCCTGCGTCCTGACTCAAGGTAGTTTCGAACCGGTTTCCAAGTTTGCCGCTGTCGGACATTTTGTCCCTGTCGGAAAGGAAGACCTGTCCCGTCTGCCGCCCGCTGTTGCAGAACCCGATGTACAGGCGTTGTGGACGCTTGAACGCGATGGCGGCATTTATTATTTGAGCCTGACGCGCGACAGTTGCAGTGTGAAAGCCGAGCGGGCAGACAGTGCCGCGCTTTTGGAACATTTCTCAGGGCTTGTCCGACAGCCGCCGAAAAATGCCAATATATAGTCAATTAAAATC

>31 |ref|NC_017511.1| Neisseria gonorrhoeae TCDC-NG08107 | Coordinates: 305716,310482 | Forward

GCTTTGTTTCTTAAGTCCGCAGAGTATGCCATGGTTAAACCTTCAACGTCGAGTGTTGTACTATTTTGTTTTTAATTGAATATAGAATTGAGGGCGGAACAGTCGGTGCAGGCACCGTTTGAAACCAGGCAAATCAGCTATGCCTGGCGGGAATCGGGCAGCCCTGAAGAAACCGTATTGACCGCACAAACCGCCGATTCCCCGGATTTACCCGTACAGGCAGTATTGAACCTAACGCACCGTTCCCATAACGGGAAACCCCTAATTCTCCCCTGACTTCAGACGGCATAAAGTCGGCATGCCGTCTGGAAACCAAAAATCTAAAAAGGAACAACCATGCAAACCCTGACCATCATCCGACCCGACGATATGCACCTGCACCTGCGCGACGGCGACGCGCTCAAAGCCGTTGCCCCTTATACCGCCCGCCAGATGGGGCGCGCCGTCATCATGCCCAACCTCAAACCGCCAGTCGTCAGCGTAGCCGACGCGCTTGCCTACAAAGCGCGCATTATGGCGGCGTTGCCCGAAGGCAGCGCGTTTGAGCCGCTGATGACGCTTTACCTGACCGACCAAGCCACGCCCGAACTCGTGCGCGAAGCCAAAGCCGCCGGCATCGTCGCCTTCAAACTCTACCCCGCAGGCGCGACCACCAATTCCGATTCCGGCGTAACCGACCTGTTCAAGCTCATCCCCGTGTTGGAAGAAATGGCAAAACAGGGCATCCTGTTCCTCGTTCACGGCGAAGTAACCGACCCCGAAATCGACATTTTCGACCGCGAAGCCGCCTTTATCGGGCGCGTGATGAAACCCGTTTTGGCGCAAGTGCCGAATCTCAAAGTCGTGTTCGAACACATCACCACCGCCGAAGCCGCCCGCCTCGTTTTGGAAGCGGGCGACAACGTTGCCGCCTCCGTGACCCCGCAACACCTCCTGCTCAACCGCAACGACCTCTTGGTCGGCGGTGTGCGCCCCCATCATTTCTGCCTGCCCGTCCTCAAGCGCGAAACCCACCGTCAGGCATTGGTCGCCGCCGTTACCGGCGAGAAGGCGCACAAATTCTTCCTCGGCACCGACTCCGCGCCGCACGCCAAATCCGCCAAAGAAAACGCCTGCGGCTGCGCCGGTATGTTCAGCGCGATGACCGCTATCGAGCTTTACGCCGAAGTGTTTGAAAAAGCAGGCGCGTTGGACAAACTCGAAGCCTTCGCCTCGGAAAACGGCGCAAGGTTCTACGGCATTCCGGAAAACGCCGACACGATTACCCTTGTCAAACAAAGCCAAACCGTTCCCGCAAGCGTCCCTTACGGCGACGGCGAACTTGTCCCGATGCGCGCGGGCGGCGAAATCGGCTGGACGGTGCAGTATTGATTGAACGGTCAAGCAAAATGCCGTCTGAAAGGTTTTCAGACGGCATTTGTGTATTTTCCGATTCGGATTTTCCGAGTATCAACGGCGTTTGGGTTCGTCTGGGCGGATTTGGGCGGCGAGTTTGTCGAGGATGCCGTTGACGAATTTGTGCCCGTCCGTGCCGCCGAAGGTTTTGGTAACTTCGATGGCTTCGTTGATAATGACGGGGTAGGGCGTTTCGGGCATAGCGGAAAGCTCGTGGCAGGCGGTCAGCAAAACGGCGCGTTCGATGGGGTTGAGGTCTTTTTCGTCCCTGTCGAGCAGCGGGCGGATTTTTTGGATGTAGTCCGCTGCGTTGGTTTGTGTGCCGAAGAAGAGTTTGTTGAACAATTCTTCGTCCGCTTTGGCAAAGTCGGACATTTCGCGGATGTTTTTAGCAATTTCGGGCGCGGCGGTGCGGTTGATAAGGGATTGGTAAACGGCTTGTACGGCAAGCTCGCGGGAACGGCGGCGGGCTGTTTTCATGATTTTTCCTTGAAACGGTTGGGCGGCACGGTATGCCGTCTGAAACGGAAAGGGCGCATCGGTGTACGCCCTGTTTGTTATTCTTCGTCTTCAAACTGTTCTTCGAGCAGAAGGTTGACGAGGTTGGCGCATTCTACGGCGACTTTGGCGGCATCCGAGGCTTTTTCTCCAATCCGTTCAATTGCCTGCGCGTCGTTTTCGGTGGTCAGGACGGCGTTGGCAATCGGGATGTTGTAGTCGAGTGCGACGCGGCCGATCCCTGCGCCGGATTCGTTGGCAACCAGCTCGAAATGGTAGGTTTCGCCACGGATGACGACGCCGATGGCAATCAGTGCGTCAAATTTTTCGGAAGAGGCAAAGTTCATCAGCGCGATGGGGATTTCAAGCGCGCCGGGTACGGTGGCGACGGTGATGTTTTCGTCTGCTACGCCCAATTCTTGGAGGGTGCGGCAGCAGACTTTGAGCATTTGGCTGCCGATTTCGTTGGTGAAGCGTGCCTGTACGATGCCGATGCGGAGGTGTTTGCCGTCGAGGTTGGGGGCGATGGTGTTCATTGGGTGTCCTTTGGTATTCGGGGTTTCGGAATGCCGTCTGAAGGTTTCAGTCTTGCGGCTGCCAGTCGGCAACGGTTTGGAATGTGCCGTCTTCGGCAAGCTCCCACGCGCTGCCTTCGGGTTGGGAAAGCAGTGCGGCGGTTTCAGGGTTGGTTTTGGTGATGTCGGCGAGGCTGACGATGCTGAAGTTGTCGGGGTCGTCGGTGTATTCGTCGGTTTCGTCGCCGCTGAAGAAACGCCAGCCGCTGTCGTTTTCAAAAACGGGGGCTTCGCGGTAAAGGAAGCCGACGGGCCGGTTTTGTTTGGCGACGGTGTTGGTGGCGATGCAGCGGTCGAGTGCCGAGGAAAGTGCTTGTGCAAATGCGTTCATTGCGGGAATACGTTGGGGGGGAAACTTGCGGATTTTACCACGATTCCCGCGTTGTCGGCAGACGGCGGCGGTTTGGTGGTACAATGTGCGCCGTTTGCAGCCTTAAGGTGTTTCTGTATTTTTGGAGTATGGAAACGCATTCGGGCTGTTTTTTGCGGAAGACGGTAATGAAAGACGATGTTTTGAAACGGCAGGCACATACTGCGATACAGAAAAAGTTAGGCTACGCGTTCCGCGATATGTCGCTTTTGCGGCGGGCTTTGACCCACAGGAGCCATCATGCGAAGCACAACGAACGGTTCGAGTTTGTCGGCGATTCGATTTTGAATTATACGGTGGCGCGGATGCTGTTTGACGCGTTTCCGAAGTTGACCGAGGGCGAGTTGTCGCGGTTGAGGGCGAGTCTGGTCAATGAGGGCGTGCTGGCGGAAATGGCGGCGGAAATGAATGTCGGCGACGGTCTGTATTTGGGGGCGGGCGAGTTGAAGAGCGGCGGCTTCAGACGGCCTTCGATACTGGCGGACGCGATGGAGGCGATGTTTGCCGCCGTCAGCTTCGATGCCGATTTCAACACGGCGGAAAAGGTGGTGCGCCATTTGTTTGCAGAACGCGTCCGGCGCGCCGATTTCCAAAATCAGGCAAAAGACGGCAAAACTGCTTTGCAGGAGGCGTTGCAGGCGCGCCGTTTCGCCTTGCCGAAATACCGCATCGAAGAGCAAATCGGCCATGCCAACGACAGTATGTTTGTCATTTCCTGCGATTTGGGCGAACTGGGTTTCGTGTGCCGTGCCAAAGGGACGAGCCGCAAGGCGGCGGAACAGGAGGCGGCGAAAGAGGCTTTGAAATGGCTGGAAGAGAAGCTGCCGCTGAAGAAGAAAAAGAAATGAGGCGGCGCGTGAATATGCCGTCTGAAATGTGGATATGAAAGCGAATATGGATATTGAAACCTTCCTTGCAGGGGAACGCGCCGCCGGCGGATACCGTTGCGGCTTCGTGGCGATTGTCGGTCGTCCGAACGTGGGCAAATCAACGCTGATGAACCATCTCATCGGTCAGAAAATCAGTATTACCAGCAAAAAGGCGCAGACGACGCGCAACCGCGTAACGGGGATTTATACCGACGATACCGCGCAGTTCGTGTTTGTCGATACGCCGGGCTTTCAAACCGACCACCGCAACGCGCTCAACGACAGGCTGAATCAAAATGTTACCGAGGCGCTCGGCGGTGTGGATGTGGTGGTTTTCGTCGTGGAGGCGATGCGCCTTACCGATGCCGACCGCGTCGTGTTGAAACAACTGCCCAAGCACACGCCGGTCATTTTAGTGATCAACAAAATCGACAAGGACAAGGCGAAAGACCGTTACGCGCTGGAGGCGTTTGTTGCCCAAGTGCGCGCCGAATTTGAATTTGCGGCGGCGGAGGCGGTCAGTGCGAAACACGGTTTGCGGATTGCCAACCTGTTGGAGCTGCTCAAGCCGTATCTGCCCGAAAGCGTACCGATGTATCCCGAAGACATGGTTACGGACAAATCGGCGCGTTTTTTGGCGATGGAAATCGTGCGTGAAAAACTCTTCCGCTATTTGGGCGAGGAGCTGCCTTATGCGATGAACGTCGAAGTGGAGCAGTTTGAAGAGGGAGACGGTTTGAACCGCATCTACATCGCCGTTTTGGTCGACAAAGAAAGCCAAAAGGCGATTTTGATCGGTAAAGGCGGGGAGCGTTTGAAAAAAATTTCCACCGAAGCGCGGCTGGATATGGAAAAACTGTTTGATAACAAAGTATTTTTGAAGGTCTGGGTCAAAGTCAAATCCGGTTGGGCAGACGACATTCGCTTCCTGCGCGAGCTGGGTTTGTAGTTTTTCTTGCTGAACTTTACGCAAATGCCGTCTGAACAGGTTTCGGACGGCATTTTGTTTCAATCGGGAATATCTTTGTTAAAAACGGGTTGATATTATCTGTGCATATTA

>32 |ref|NC_017511.1| Neisseria gonorrhoeae TCDC-NG08107 | Coordinates: 310483,390123 | Forward
[truncated: 1,833,171 more chars]
