# Supplementary material for: Unexpected cross-species contamination in genome sequencing projects
Source: PeerJ. 2014 Nov 20;2:e675. doi: 10.7717/peerj.675 (PMC4243333; doi:10.7717/peerj.675)
Supplement: Supplemental Information 3 [file peerj-02-675-s003.docx]

>1 |ref|NC_017511.1| Neisseria gonorrhoeae TCDC-NG08107 | Coordinates: 499351 - 499709

AATTATTGATAAGTATGATCCCGTTGCCATTTACTTTATTGTTTTGGGTTCGAGTTTATACACCCTTTTCGTGTTTCCTGTCTAGAGGATATCCTTTAGAATTTGTTGGAGAGCTGGTTTGGTGGTGCTGAATTCTCTCAGCTTTTGCTTGTCTGTAAAGCTTTTGATTTCTCCTTCATATTTGAATGAGATCCTTGCTGGGTACAGTAATCTGGGCTGTAGGTTATTGTCTTTCATCACTTTAAGTATGTCTTGCCATTCCCTCCTGGCCTGAAGAGTTTCTATTGAAAGATCAGCTGTTATCCTTATGGGAATCCCCTTGTGTGTTATTTGTTGTTTTTCCCTTGCTGCTTTTAAT

>2 |ref|NC_017511.1| Neisseria gonorrhoeae TCDC-NG08107 | Coordinates: 1267185 - 1267393

CTTGCTCTCTCCTCTCTTGATCTCACCGCATCTCATTCCAGTTACCTCTAACTACCCCCTCCATCTTCTCTTCTCCTTGTAACTCAGTGAACCTCTCTGAGTGTCCCTCAATGTGGAGAAACTTTTCATCTTTAACCTAGATGTTTTATCATCGGTGCTGTATAGATGGAGAAGTCTAGAGGCTACTGTAAAAATAAAACTGAAAACC

>3 |ref|NC_017511.1| Neisseria gonorrhoeae TCDC-NG08107 | Coordinates: 1371560 - 1371932

CCTGAGCTTCAATATACAGGCAGCTCAAAGTTACTCCAAAACCTTTGATGTCTCATAACCCATTACGGGTCACTCCACTGCACTCCAGAGAGAAGAAACCCAGCTCCACCCACCAAAACTCCAACACAAGCCTCCCTAACCAGGAAACCTTGACAAGCCACTGATAGAACCCCACCCAAAGTGAGGAAGCTCCATAATAAAGAGAACTCCACAAATTACCAGAATATAAAAAGGCCACCCCAAACGCAGCAATATAACCAAGATGAAGAGACAGAGGAATACTCAGCAGGTAAAGGAACAGGAGAAATGCCCACCAAACCAAACAAAAGAGGAAGAGATAGGGAATCTACCTGAGAAAGAATTCCGAATAAT

>4 |ref|NC_017511.1| Neisseria gonorrhoeae TCDC-NG08107 | Coordinates: 1635755 - 1635954

CTACACCTAAAGCAACTAGAAAAGGAAGAAATGGAGAACCCCAGAGTTAGTAGAAGGAAAGAAATCTTAAAAATTAGGGCAGAAATAAATGCAAAAGAAACAAAAGAGACCATAGCAAAAATCAACAAAGCCAAAAGCTGGTTCTTTGAAAGGATAAATAAAATTGACAAACCATTAGCCAGACTCATCAAGAAACAAA

>5 |ref|NC_017511.1| Neisseria gonorrhoeae TCDC-NG08107 | Coordinates: 2118014 - 2118647

TGAGGCAAGTCTGGGCAAGGCTCGCCCAGGGCAGAGCAGCCTCGCGCCAGCAACTCTGCCAGATCGGGCCTCTGAGCAGAGCTGGAGCTCTGCCGTCCTGGAGGCGCACTCGGCTCCAGCCAAGTAGGGCTTGAGCCAAACGAGGCTGGGCTGCCTCTTCTTCCCTCGAGGAGGCTCGCCTCCCCGCCAGCCGTTCACTCGGCTTCCAGGTGGGGGCGCTCTGCGCCTCCTGCAGGTCTTGGCTCAGGGGCTGCTTGCCCTGAGCTGGGGCTGTGGGCGGGAGCCCAGGCACGCAGGGGCAGGCCCCAACCAGCTGCACACGTCTCTGCTGGGGAGGAGAGCGCTAGGGAAGCTACGTGTGTGAGGCAGCTGCTGGGCGCCCAGGGAGTCAGCCTCCGGGTGTGTGGGGCTGCAGACCCCAGCCAGGGACCCTGGGAGCCTGGTGCACTCCGTGCAGCGCTGAGTCTGGGCGGCCAAGCAACGGCTGGAGGAGCCCTGCACTAGCCCTTTCCTTCGCTCACACAGAGCTCTTCCTGCTGGAGCCCTTGGCAGTGTGCAAGCCACCGTCCCCTAAGCCCTCCCCAAGGACGCCTGGGGCCTAGGTGCAAAGGCAAGGCAGGAGCGGAGCTGAGG
